# Polymers without Petrochemicals: Sustainable Routes to Conventional Monomers

**DOI:** 10.1021/acs.chemrev.2c00354

**Published:** 2022-10-13

**Authors:** Graham Hayes, Matthew Laurel, Dan MacKinnon, Tieshuai Zhao, Hannes A. Houck, C. Remzi Becer

**Affiliations:** † Department of Chemistry, University of Warwick, CV4 7ALCoventry, United Kingdom; ‡ Institute of Advanced Study, University of Warwick, CV4 7ALCoventry, United Kingdom

## Abstract

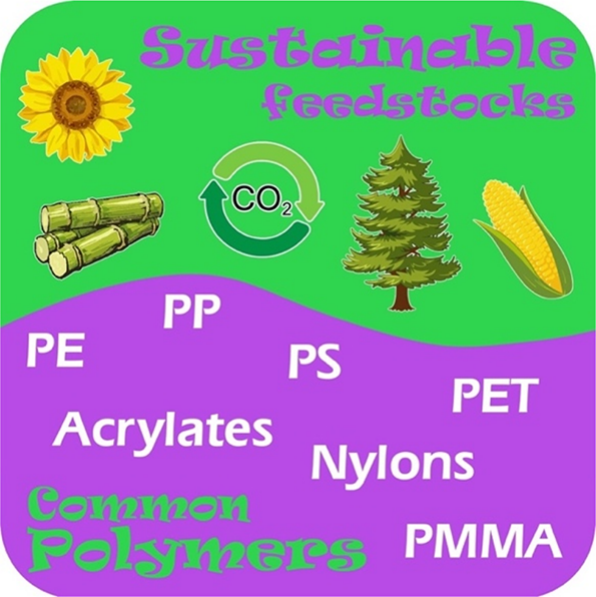

Access to a wide range of plastic materials has been rationalized by the increased demand from growing populations and the development of high-throughput production systems. Plastic materials at low costs with reliable properties have been utilized in many everyday products. Multibillion-dollar companies are established around these plastic materials, and each polymer takes years to optimize, secure intellectual property, comply with the regulatory bodies such as the Registration, Evaluation, Authorisation and Restriction of Chemicals and the Environmental Protection Agency and develop consumer confidence. Therefore, developing a fully sustainable new plastic material with even a slightly different chemical structure is a costly and long process. Hence, the production of the common plastic materials with exactly the same chemical structures that does not require any new registration processes better reflects the reality of how to address the critical future of sustainable plastics. In this review, we have highlighted the very recent examples on the synthesis of common monomers using chemicals from sustainable feedstocks that can be used as a like-for-like substitute to prepare conventional petrochemical-free thermoplastics.

## 1 Introduction

The amount of plastics produced ever worldwide has exceeded 8.3 billion metric tons and a large proportion of these materials has ended up in a landfill.^1^ Owing to the excellent chemical stability of plastic materials and their low degradation rates in the environment, the accumulated plastic waste in landfills and marine environments has become more and more noticeable over the last decade.^2^ Thus, degradation analysis over prolonged periods has become possible and the influence of key parameters such as crystallinity and hydrophobicity can be studied alongside the chemical structures and toxicity of resulting plastic degradation products.^3,4^ Bio-based polymers that are degradable are considered to be a solution for the landfill waste and have been reviewed widely in the literature.^2,5−9^ However, the process of creating new plastics and establishing their life cycle analysis is long, costly and risk-taking for the chemical industry.^10^ Moreover, material properties of newly invented plastics are often inferior to those of established thermoplastics, making it difficult to compete with conventional technologies. Therefore, the chemical companies and the plastic industry remain reluctant toward the introduction of completely new plastic materials and typically maintain a more conservative approach in marketing plastics with completely new chemical structures even if they can be fully obtained from sustainable resources.^11^

Renewable resources such as lignocellulose, vegetable oils, terpenes and carbohydrates are used as feedstocks for the synthesis of sustainable polymers.^12−14^ Biomass has been used to produce hundreds of value-added compounds that are further used in the synthesis of polymers.^15−18^ However, most of these polymeric structures are novel polymers with new chemical compositions in comparison to common plastics. Therefore, it would require a significant capital investment to commercialize any of these novel polymer structures as sustainable polymers. On the other hand, using cost-competitive sustainably sourced monomers in cemented manufacturing processes would enable the synthesis of known polymeric structures and hence sustainable plastics in a timely manner. As this implies creating the same plastic with the same chemical composition, there is no requirement of registering such conventional polymers or applying for safety certificates from regulatory authorities such as the Food and Drug Administration (FDA) and Environmental Protection Agency (EPA).^4,19,20^

**Scheme 1:**
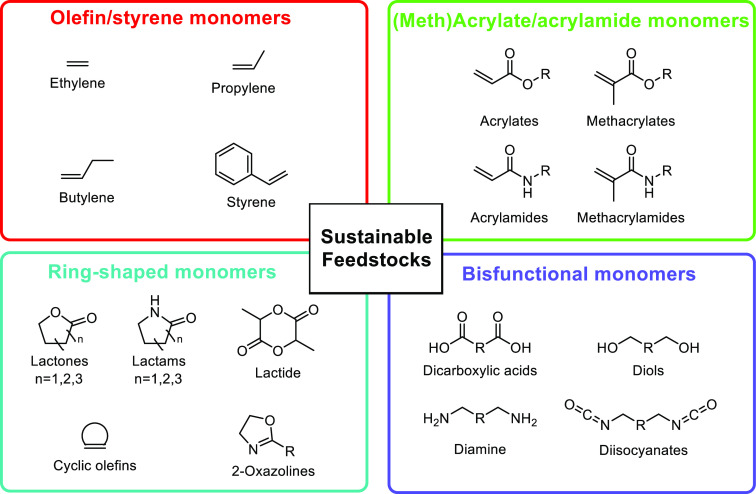
Overview of the Four Sections in This Review Article That Are Categorized Based on the Types of Monomers That Are Obtained from Sustainable Feedstocks and Used in the Synthesis of Commodity Thermoplastics

With the increasing fuel prices and geopolitical dependence, the research on finding new chemical resources to create commodity plastics without using petrochemical derived chemicals is expanding on an exponential scale. Most reviews on sustainable polymers are focused on a particular bio-based feedstock from which an array of monomers and materials can be prepared. Instead, this review is centered around the specific monomer classes that are used in conventional polymerizations and summarizes routes to access these from renewable resources. We have categorized the sustainable building blocks in four groups, i.e., olefins/styrenics, (meth)acrylics, cyclic monomers and commonly used bifunctional monomers that are used in step-growth polymerization (Scheme 1). The main aim of this review is to present a roadmap toward bio-sourced monomers that could be considered in order to replace petrochemically derived bulk monomers that are currently used in the production of commodity plastics, on a like-for-like basis. The first section is describing the synthetic routes to obtain olefins and styrenics used as building blocks in the production of polyethylene (PE), polypropylene, polybutylene and polystyrene. The main chemical that is used to create PE is bio-sourced ethanol and sustainable bioresources and its chemical dehydration process to ethylene have been highlighted. The following section is focusing on the (meth)acrylics and (meth)arylamides monomer classes. There is a large variety of acrylic polymers, of which the most widely used and commercially available polymeric structures have been discussed. The next section is based on ring shaped cyclic monomers mainly to create polylactones, polylactams and polylactides (PLA). This category is specifically important as PLA has become one of the most popular plastics materials for single use applications. In the final part, we have focused on the range of bisfunctional monomers that are used in the synthesis of some important step growth polymers such as polyesters and polyamides. While there are many other functional groups (e.g. epoxides) or multifunctional (f ≥ 3) derivatives that are receiving increased attention for the formation of thermosets, we have directed our focus to those most widely used in the synthesis of commodity thermoplastics.

## 2 Olefin and Styrenic Monomers

Polyolefins derived from olefin monomers have a broad range of industrial applications as a result of their outstanding properties such as transparency, gas permeability, heat and chemical resistance.^21−24^ Hence, polyolefins are commonly used in plastics for food storage, packaging, piping and clothing.^25^ In 2015, polyolefin materials constituted 45% of the consumer plastics and half of those produced,^21^ while it was reported in 2018 half of the global plastic market consisted of polyolefins.^26^ The two most used polyolefins are polyethylene, which includes both low and high density polyethylene, and polypropylene that dominates in the dairy packaging and plastic hinge production.^25^ However, this continuous cycle of production and consumption has led to plastic waste becoming a significant issue.^21^ The stability of polyolefin materials causes extremely slow degradation of these materials, and combustion of polyolefins leads to the problem of releasing additional volitale organic compounds (VOCs) that can contribute to climate change. It was estimated that only around 20% of plastics used since 1950 are recycled while the rest either end up in landfill or remain unaccounted for, which leads to terrestrial and aquatic “white pollution”.^21,26^ The consumption of non-renewable petrochemical resources to make olefin monomers for the preparation of polymers is also massive, constituting 5% of petroleum and natural gas resource consumption. Stemming from growing concerns regarding global climate change, the use of fossil-fuel resources is being thoroughly revised. As commodity polyolefin plastics remain undervalued in our society and hence facilitate plastic pollution, the development of methods to prepare “bio-labelled” polyolefins in sustainable ways from renewable resources has attracted significant research attention.^21,23,24,27−29^

While preparing bio-based polyolefins, several factors should be considered to maximize their sustainability. As highlighted by Siracusa and Blanco,^21^ there are three standards for bio-plastics. First, the monomer used in the polymerization should be fully or partially derived from biological sources. This is to prevent any competition with the food chain and ensure food security for animals and humans. Second, the preparation strategy should be energy efficient, non-hazardous and produce minimal waste. Finally, the resulting polymeric material should be, ideally, chemically or mechanically recyclable or degradable under biological or chemical conditions, ideally mild ones, for example by being compostable with low or no hazardous residue. One good example of the chemical or mechanical recycling of bio-plastics is a recent report on polyethylene-like material prepared by Häussler et al, where the bio-plastic reported was chemically degradable under 120 °C in basic ethanol.^30^ However, the degradation behavior depends on the stability of the chemical structure of the polymer, which also largely determines their mechanical and thermal properties. Thus, high degradability for hydrocarbon polymeric materials with stable structures such as polyethylene may prove unattainable. In reality, it is difficult to achieve all these standards simultaneously, and as long as polymers fulfill one of these criteria, they are typically considered bio-plastics.^21,22,25^ For bio-polyolefins, the first criteria regarding the bio-based monomer feedstock is the most commonly fulfilled. This section will focus on existing work on the synthesis of bio-based olefin monomers, as well as their use of feedstock for the preparation of its corresponding commodity plastic materials.

### 2.1 Ethylene

**Figure 1:**
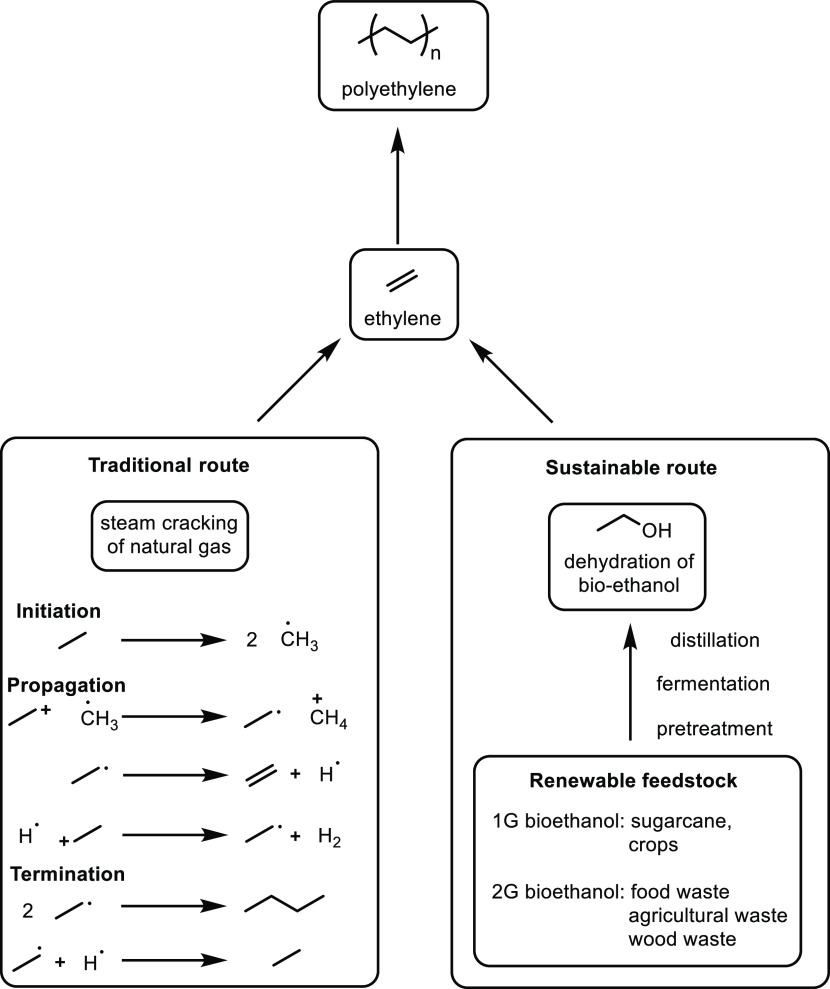
Polyethylene from the traditional way and the sustainable way.

Polyethylene, the most used polymer in the world, represents more than 30% of the global plastic market and is produced in several forms including low-density polyethylene (LDPE), linear low-density polyethylene (LLDPE) and high-density polyethylene (HDPE).^21^ PE is a homopolymer of the monomer ethylene, which is mostly obtained as a distillation product of petroleum. Conventionally, ethylene is prepared via the thermal cracking of hydrocarbons under 750–1000 °C in catalytic reactors.^21,24,31^ As PE is widely produced and consumed, the amount of petroleum sources used for the polymer production has raised concerns on non-renewable energy sources. Hence, the approach of deriving renewable ethylene monomers from bio-ethanol has been widely employed as the current solution to the bio-production of ethylene in order to reduce the consumption of petroleum sources. Several biomasses such as sugarcane,^32−36^ corn,^37−39^ food waste^40−43^ and wood waste^44−46^ have been reported to be converted into bio-ethanol via procedures including pre-treatment, saccharification (or hydrolysis) and fermentation. The core actions in these processes are the extraction of polysaccharides and oligosaccharides from the feedstock and fermentation of the obtained sugars into ethanol. There are two classes of bio-ethanol (bio-EtOH): 1G bio-EtOH and 2G bio-EtOH.^32,47,48^ 1G bio-ethanol is commonly used to refer to bio-EtOH derived from sugarcane or crop grains,^33,39,47,48^ while 2G bio-EtOH is derived from lignocellulosic biomass.^49−51^ As lignocellulosic biomass waste is produced in vast amounts and does not compete with food production, investigations into 2G bio-EtOH has become the main focus of new bio-ethylene production research. A comparison of ethylene preparation procedures via petroleum and biomass resources is shown in Figure 1. There are several general steps in the bio-production of ethylene, which are the pre-treatment of the bio-based feedstock, fermentation, distillation and catalytical dehydration, and these steps are discussed in detail in the following section.

#### 2.1.1 Renewable Feedstock for Bioethanol Production

Bio-EtOH, the bio-based starting material for the bio-production of ethylene, can be obtained from various sources. Several reviews and reports have been published on different feedstocks used to produce 1G and 2G bio-EtOH, which can be employed for bio-ethylene production.^34,42,50,52−56^ A summary of reported feedstocks, along with their conversion, is shown in Table 1.

**Table 1:** Examples of Reported Renewable Feedstocks for Bioethanol Production

Feedstock		Yield to sugar(g/g)	Conversion to sugar (%)	Productivity(g/L h)	Max. concentration(g/L)	Ref.
Agricultural products	Bamboo shoots	0.169	98			(57)
	Coconuts and cactus		90.09	0.21		(58)
	Cassava				35.01	(59, 60)
	Corn^a^					(37−39, 61)
	Potato^b^					(62)
	Sugarcane^c^					(32−34, 36, 48)
	Sugar beet				132.39	(63)
	Sweet sorghum	0.47			82	(56, 64−66)
	Wheat				132.1	(38, 67)
	Sweet potato				38 to 100	(41, 55)
	Paper mulberry fruit juice	0.48		4.7	73.69	(68)
Food wastes	Banana peel^d^					(69)
	Cashew apple pulp	5	46			(70)
	Coffee pulp	0.46	40			(70)
	Pineapple waste		96			(40)
	Potato waste	0.32			22.54	(71−73)
	Sugarcane juice				42.1	
	Sweet potato waste	0.46	47		79.00	(41, 55, 71)
	Brewery waste				219	(74)
	Coconut	20% v/v				(75)
	Mandarin peels	50–60 L/t				(43, 76)
	Citrus wastes	93.1%			14.4 to 29.5	(77)
	Olive mill solid		3			(78)
	Seaweed	84.9%			25.7	(79, 80)
	Waste hamburger	0.271			27.4	(81)
	Pomegranate peel	0.50		0.99	14.35	(82)
Agricultural wastes	Banana pseudo stem					(83)
	Sugarcane bagasse	102 L/t	84	0.024	17.1	(49, 51, 84, 85)
	Sweet sorghum bagasse	0.48	81	0.59		(64)
	Vineyard waste		13.1			(86)
	Grape pomace	0.419	82.1			(87)
	Corn Stover	330 L/t				(61, 88−93)
	Cotton stalk	52%			47.0	(90, 94, 95)
	Sesame plant				1.90	(96)
	Rice straw	61.3%			29.1	(97−99)
	Wheat straw	0.39–0.42	68–76			(100, 101)
	Grass biomass	32.7%–95%			4.72–32.1	(102, 103)
	Banana frond juice	0.33			45.75	(104)
Wood wastes	Acacia wood		90			(105, 106)
	Apple wood		70			(107)
	Eucalyptus globulus wood				93	(108, 109)
	Fir wood				43.96	(45)
	Hornbeam wood	251 L/t				(110)
	Japanese cedar wood		1.7–30			(111)
	Pine wood		44			(105, 112)
	Sengon wood		18			(44)

a Over 4.38 billion bushels of corn was used to produce over 68 billion L of ethanol in the US in 2016.

b Low concentration of bio-EtOH produced.

c Commercialized 1G bioethanol production.

d Different pretreatment techniques have been studied using same acidic hydrolysis procedure. It was shown that acid pre-treatment give rise to highest reducing sugar concentrations but did not guarantee a highest bioethanol concentration.

Agricultural products like sugarcane are used to produce 1G bio-EtOH, and a range of other crops like potato and wheat have also been used.^32,34−39,48,55,62,67^ Two main sources for 1G bio-EtOH production are sugarcane juice and molasses (as well as a third, corn); these two renewable feedstocks have been commercialized mainly in Brazil and the US.^33,36−39,47,52,61^ Various sugars are obtained from the first two feedstocks *via* crushing and extraction with water, while glucose is obtained from corn feedstocks *via* the hydrolysis of starch in milled corn grains. Bio-EtOH production costs are low due to high yields of sugar obtained from the feedstocks and the high conversion of sugar to bio-EtOH. 1G bio-EtOH therefore is cost-efficient enough to be competitive with petroleum fuel.^35,51^ Additionally, bio-EtOH production from sugarcane is reported to consume far less energy than from corn, explaining why ca. 25% of global bio-EtOH production is from sugarcane in Brazil.^34−36,113^ The production of bio-EtOH from sugar has been widely investigated. For example, Morschbacker briefly reviewed the utilization of sugar as the feedstock for bio-EtOH production in Brazil in 2009, introduced the general conversion procedures, and highlighted the equipment used in the process.^114^ In 2010, Dias *et al*. reported the improvement seen when using distillation and co-generation systems and performed simulated analyses producing 1G and 2G bio-EtOH from sugarcane with different pre-treatment methods.^48^ Carvalho *et al*. analyzed the economic-energy-environmental factors of several bio-EtOH production programs from sugarcane, the illustrative results of which suggested the energy balance and gas emissions could be improved by using a combination of optimized production methods, including 2G bio-EtOH production.^34^ The life cycle evaluation study by Luo *et al*. confirmed that a reduction in greenhouse-gas emissions was possible through producing bio-EtOH from sugarcane, when compared to conventional petrochemical feedstocks.^36^ These works have highlighted the sustainability and promising potential of a sugarcane-based bio-EtOH market.

Corn is another renewable feedstock that has been commercialized for 1G bio-EtOH production, particularly in the US. More than 4.38 billion bushels of corn were used to produce over 68 billion liters of bio-EtOH in the US in 2016.^37,38^ The life cycles of the bio-EtOH production systems of Brazilian sugarcane and US corn^24,34,36,37,39,47,51,52^ were studied with different parameters, including the renewable energy ratio (RER), which was defined as the total renewable energy produced per unit of fossil energy consumed, and the greenhouse gas (GHG) emissions reductions. In both systems, high GHG emission reductions and high RER values were determined, supporting their sustainability aspect. Various research groups have reviewed the production of bio-EtOH from corn in detail.^24,37,38^ In 2019, Kumar and Singh summarized the current status of bio-EtOH production from corn, with discussions on several factors like corn composition and structure, the bio-EtOH production from corn grains, corn stover and corn fiber, and technology advances in the corn-to-ethanol system.^37^ Mohanty and Swain have also discussed the technological aspects of using corn as a renewable feedstock for bio-EtOH production and stated several advantages of the corn bio-ethanol system in a 2019 report.^38^ They introduced the corn bio-EtOH production process from farm to fermentation, compared the dry-milling and wet-milling pre-treatment processes, and discussed several microorganisms used in the fermentation process. They also compared the corn bio-EtOH system with a possible wheat bio-EtOH production system from a technological perspective.

Apart from sugarcane and corn grains, other reported crops include wheat,^38^ potato,^62^ sweet potato,^55^ cassava,^60^ bamboo shoot,^57^ sugar beet,^63,87,115^ grape and cactus,^87^ for which a range of pre-treatment processes and fermentation systems were employed. Fruit juices were also reported as potential bio-EtOH production systems with high EtOH concentrations reported.^68,75,104^ For example, paper mulberry fruit juice was studied as a bio-EtOH feedstock by Ajayo *et al.*, where a high bio-EtOH concentration of 73.69 g L^–1^, a high yield of 0.48 g g^–1^ to sugar and high productivity of 4.7 g L^–1^ h^–1^ were recorded. The advantage of using food crops for bio-EtOH production is their usual abundance and low-prices in certain areas. Additionally, the by-product in the bio-EtOH production process can be recycled for crop fertigation, improving the sustainability of the route.^38,50,52,54^ However, the main drawback of producing bio-EtOH with agricultural products is the possibility for competition with food crops, which triggers debate on the topic of food-versus-fuel and increases food prices and security concerns. Moreover, the production of bio-EtOH from crops is only competitive with petrochemical feedstocks in certain areas, which means the production would be localized and may not become a strong force in bio-EtOH production. Thus, a more generalized solution which avoids competition with food crops would be more advantageous for the future of the bio-EtOH industry.

During the production of bioethanol with sugarcane, a common side product is sugarcane bagasse, which is normally burnt to provide energy.^49,84,85^ However, sugarcane bagasse also includes polysaccharides and has potential for utilization in bio-EtOH production.^85^ It was first used to co-produce bio-EtOH to reduce the overall cost in the process of producing bio-EtOH with sugarcane.^48,51^ To avoid competition with food crops, reduce the cost value of starting materials, and solve the environmental problem of agricultural waste, using polysaccharide-rich agricultural waste for bio-EtOH production was suggested as a practical solution. Crop residues like bagasse, stalk, straw and leaf wastes are the most common agricultural wastes and a variety of these have been reported as potential bioethanol production systems.^64,84,85,90,94,97−99,116^ For example, sugarcane bagasse, the most common by-product of sugarcane harvesting and processing, has been subjected to techno-economic evaluation of its impact on biorefinery processes in different reports, and both integrated 1G and 2G bio-EtOH production systems and stand-alone 2G bio-EtOH production system have been studied.^48,49,51^ For instance, Macrelli *et al*. has reported the study on 2G bio-EtOH production from sugarcane bagasse and leaves and integrated the production system with traditional 1G bio-EtOH production from sugarcane.^51^ It was shown that with a yield of 102 L ton^–1^ dry sugarcane with 50% leaves, the cost of bio-EtOH production could be reduced by about 50%. Other agricultural wastes have also been studied. Kadam and McMillan investigated the feasibility of bio-EtOH production with corn stover as a renewable feedstock in 2002 and pointed out about 80 million tons of dry corn stover could be available for bioethanol production.^88^ Several reports have studied the production of bioethanol with only corn stover or with a mixed corn/corn stover system since then, with variations in several factors including pre-treatment method, corn stover varieties and solid loading.^61,88,90,93^ Wheat straw and rice straw are another family of food crops residues and studies have shown the feasibility of producing bio-EtOH with these agricultural wastes.^100,101^ Belal has reported the utilization of rotted rice straw residues for bio-EtOH production with optimization of pH and temperature for the microorganism mediated saccharification process.^98^ The highest bio-EtOH concentration was reported after 7 days of fermentation as 11 g L^–1^. Yoswathana and Phuriphipat studied bio-EtOH production from rice straw with different pre-treatment methods including acid treatment, alkali treatment and physical treatment, as well as enzymatic saccharification methods.^97^ A combination pre-treatment method of acid and ultrasound followed by enzyme saccharification give rise to the highest bio-EtOH concentration, which was still lower than 15 g L^–1^. Increasing rice straw loading for the bio-EtOH production may increase the yield and make the process more practical. Georgieva *et al*. reported the production of bio-EtOH with wet-exploded wheat straw in a continuous immobilized reactor system,^100^ and an ethanol yield of 0.39 to 0.42 g g^–1^ sugar was reported with conversion in a range of 68-76% from sugar. Another study by Tamburini *et al*. also achieved similar ethanol yield from sugar of 68%.^101^ Comparison with other systems, the low yield of this system might prevent large-scale application. Other agricultural wastes including grass biomass,^102,103^ sesame plant,^96^ banana pseudo stem,^83^ sweet sorghum bagasse,^64^ vineyard waste,^86^ cotton stalk^90,94^ and coffee crop residue^117^ were also reported to produce bio-EtOH with yields from sugar ranging from 6% to 95%.

Apart from agricultural wastes, food wastes also constitute a lignocellulosic content-rich feedstock with potential for bio-EtOH production.^42^ It was reported that 1.3 billion tons of food wastes were produced each year, with landfill disposal both environmentally problematic and a waste of carbohydrate materials with huge potential.^42^ Examples of studied food wastes for bio-ethanol production include pulp wastes from coffee,^117^ apple pulp wastes,^70^ banana^69,104,118^ and peel wastes from mandarin,^43,76^ banana^69^ and potato.^72,73^ For example, Chohan *et al*. reported bio-EtOH production from potato peel wastes utilizing the simultaneous saccharification and fermentation (SSF) method, with optimization of temperature, pH and solid loading.^73^ With optimized conditions, the highest bio-EtOH concentration reached 22.54 g L^–1^ with a yield to sugar of 0.32 g/g. Choi *et al*. studied bio-EtOH production from soybean waste (okara) and compared the bio-EtOH conversion from sugar of raw okara and pre-treated okara.^119^ With the assistance of microorganisms, the concentration of bio-EtOH obtained could reach 59.1 g L^–1^ with 96.2% conversion to sugar, which indicates okara might be a candidate for the high-yield production of bio-EtOH. A recent study from Han *et al*. investigated the usage of a waste hamburger as a bio-EtOH feedstock.^81^ Enzymatic saccharification and fermentation methods were employed, with the hydrolysis rate shown to remain steady under increasing enzyme loading volumes, although higher bio-EtOH concentration could be achieved through increased enzyme loadings. Maximum bio-EtOH concentration was reported as 27.4 g L^–1^, corresponding to 0.27 g/g waste hamburger. These studies illustrate the potential of using food wastes as bio-EtOH production systems. Due to the benefits of low cost and reducing the environmental impact of food waste, the sustainability of a food waste bio-EtOH system should appear promising.

Wood waste was recently developed as a renewable source for bio-EtOH production.^45,46^ Large amount of carbohydrates including cellulose, lignin and hemicellulose are contained in wood wastes. Compared to agricultural wastes and food wastes, the higher density of wood waste results in lower transport cost, and the cultivation and harvesting of wood avoids competition with food production. Thus, wood and wood wastes such as sawdust, leaves and felled trunks were explored for their value in 2G bio-EtOH production. For example, acacia wood was studied by Lee *et al*. for the bio-EtOH production.^106^ Alkaline pre-treated acacia wood was used for the production of bio-EtOH, and the yield of bio-EtOH to sugar was reported to be above 90%. Romani *et al*. reported the production of 2G bio-EtOH from steam-exploded *Eucalyptus globulus* wood.^109^ A high bio-EtOH concentration of 51 g L^–1^, which corresponds to 91% conversion from sugar, was reported. Although the autohydrolysis process used was high-temperature and energy-intensive, the high bio-EtOH concentration and conversion still indicate the potential of this system. Decent summarizing work has also been published in the field of bioethanol production from wood wastes.^45,46^

To summarize, various renewable feedstocks have been reported for both 1G and 2G bio-EtOH production. While considering the benefits of low-cost feedstocks, reductions in environment issues, and the maximum utilization of waste materials, bio-EtOH production from lignocellulosic wastes is attracting intense research interest and should have a promising future as novel solutions to bio-EtOH production.

#### 2.1.2 Monomer Preparation

**Figure 2:**
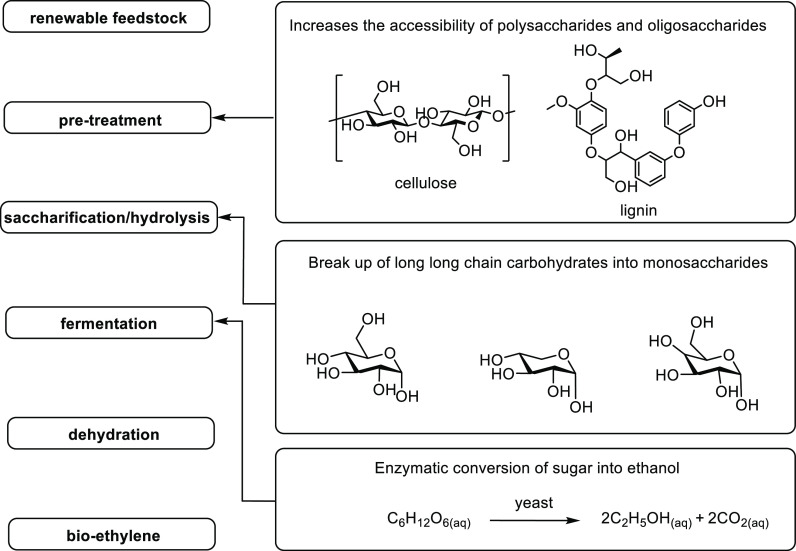
Steps of converting renewable feedstock into bio-ethylene.

With a range of renewable feedstocks available for bio-EtOH production, a reliable procedure for the subsequent conversion of bio-EtOH to bio-ethylene is required for polymerization. In established studies, the procedure of producing bio-ethylene typically includes the pre-treatment of feedstock, saccharification (or hydrolysis), fermentation, distillation and catalytic dehydration (Figure 2). These steps will be herein introduced.

##### 2.1.2.1 Pre-treatment

Pre-treatment is an important step in the production of bio-EtOH, as it can help break down lignin and glycosidic chains and increase lignocellulose digestibility.^67,102^ The major objectives of the pre-treatment process are to reduce the physical size of the starting materials, to increase the accessibility of carbohydrates (hemicellulose, cellulose, lignin) to chemicals or enzymes in the hydrolysis step, to yield improved fermentable sugars, and to reduce the crystallinity of the cellulose matrix. Pre-treatment processes are highly recommended as they increase subsequent yields of the fermentable sugars, prevent premature degradation of the yielded sugars, and prevent the formation of inhibitors prior to hydrolysis and fermentation.^67,95,102,116^ It was also reported that it lowers the demand of conventional energy in general. For example, in bulky renewable feedstocks like corn stover or wood, a pre-treatment step for physical size reduction is important to increase the total surface area of the material, allowing better accessibility for subsequent processing.^46,89,91,106,109^ Simple mechanical pre-treatment is adequate for starch-based materials while chemical pre-treatment might be required for starting materials like wood, to help breaking the tough fiber structure and cell walls.^109,111,112,120^ With smaller starting materials, easy hydrolysis methods like heating with water can be employed, which reduces the economic and energy cost of the overall procedure.^60^ However, the bio-EtOH yield may vary with different methods of pre-treatment, influencing the balance of cost-efficiency versus maximum yield. Physical pre-treatment was predominantly reported for lignocellulosic starting material.^85,99^ With additional pre-treatment methods, better hydrolysis conditions can be used in the following stage, and a better yield of sugars hydrolysis can be obtained.

Studies have also shown that bio-EtOH production is significantly impacted by the choice of pre-treatment.^76,82,92,94,102,111,116^ Pre-treatment methods can be subdivided into biological, chemical, physical and physicochemical pre-treatment. Biological pre-treatment employs microorganisms to perform the pre-treatment task, which degrade the polysaccharide and oligosaccharide components of the feedstock to amorphous forms. Some typical microorganisms used for degradation of polysaccharides include brown rot, soft rot and white rot fungi.^111^ While brown rot was able to degrade cellulose, soft and white rot were able to degrade both cellulose and lignin. Of these fungi, white rot fungi is considered to be the most favorable biological pre-treatment agent due to its ability to degrade polysaccharide and break the structure of lignin.^52^ Biological pre-treatment is favorable due to its high sustainability, as only mild conditions are required during the process. Moreover, the innate ability of the fungi to degrade the lignocellulosic wall makes this process achievable without additional chemicals. Therefore, this can be considered the most sustainable approach. However, pre-treating the renewable feedstock by this method results in a slower production rate and reduced applicability to industrialized large-scale production. Combining biological pre-treatment methods with another efficient pre-treatment method may present a solution for the sustainable pre-treatment of lignocellulosic feedstocks for large scale bio-EtOH production.

Chemical pre-treatment is the process of adding supporting chemicals to break tough crystalline regions or cell walls, making the material more suitable for subsequent saccharification. It was believed to be the most suitable method for commercial scale production.^46,52,92,116,121^ The advantages of using chemical pre-treatment include better availability and durability of chemical substances, lower cost and resilience toward technological developments (when compared to the enzymatic method, where engineered enzymes cost more than traditional enzymes). The chemical substances degrade the lignocellulosic walls and long-chain polysaccharides with chemical reactions that demand energy. Common chemicals used in the pre-treatment include acids and alkalis.^24,42,46,52,72,82,96,122^ Several commercially available acids including hydrochloric acid, phosphoric acid, nitric acid, and sulfuric acid were used in acid pre-treatment, as well as several organic acids such as (per)acetic acid, maleic acid and lactic acid.^50,82,96^ Acid pre-treatment can be performed either in a concentrated or diluted method. Concentrated acid pre-treatment requires short time-periods and mild temperatures, but often also results in the production of inhibitors and a reduction in bio-EtOH yield. Dilute acid pre-treatment leads to the same outcome as the concentrated acid pre-treatment, while dilute acid pretreatment does not require acid recovery with negligible acid loss. When compared to acid pre-treatment, alkali pre-treatment breaks more lignin component into a better accessible form for subsequent hydrolysis.^50,52,92,116^ Sodium and potassium hydroxide are the most frequent alkaline solutions used in alkali pre-treatment.^92,106,116^ Ammonia is also commonly employed as a pre-treatment agent in an attempt to produce 2G bio-EtOH.^89^ Pre-treatment methods using ionic liquid fracture the non-covalent structure between hemicellulose, lignin and cellulose, caused by the strong hydrogen bond acceptors of the ionic liquid contained in chloride.^50^ Although the suitability of ionic liquids with enzymes and fermentation microorganisms is still being investigated, ionic liquid pre-treatment is considered as a green, sustainable method.^42,54,114,123^

Another way to pre-treating the biomass is mechanically, where the size of biomass is physically reduced through cutting, chopping or other physical means. The aim is to decrease the biomass crystallinity, enhancing the remaining bio-EtOH production processes. For example, smaller-sized corn stover gave 1.5 times higher yield than a larger size corn stover substrate.^88,91,93^ The most significant challenge for mechanical pre-treatment, however, is the significant power consumption.^50,92^ Power consumption for mechanical comminution can be controlled by adjusting the sizes of the initial input and desired final substrate, but will nonetheless impact the sustainability aspect of the overall process. In addition, the material’s woody characteristics and moisture content also influence the required power input for this pre-treatment method.^46,109,112^ Microwave irradiation applying electromagnetic fields for internal heating has also been investigated.^99,116^ In bio-EtOH production, this approach is useful to selectively heat the polar bonds of the starting materials by vibrating the structure until the material is internally heated. As a result, complex lignocellulosic structures are fractured and made available for subsequent hydrolysis and fermentation.

Physiochemical methods, such as steam explosion, is another category of frequently employed pre-treatment process.^43,109,110,112^ Steam explosion applies a combination of hydrothermal and sudden pressure changes to convert the biomass. This method is categorized under physicochemical pre-treatment for its fused mechanical- and chemical-driven characteristics. The steam strikes the biomass causing fiber separation and physically shortening of the fibers. In terms of chemical changes, auto-hydrolysis of acetyl groups that exist in hemicellulose takes place under high temperature, forming acetic acid, and an acidic environment can be initiated when the water is treated at high temperature. For this nature, steam explosion is more beneficial on hemicellulose-rich biomass. However, formation of inhibitors as the by-product of the steam explosion method is one major challenge that may disturb the microbial activity during the subsequent fermentation step.

##### 2.1.2.2 Saccharification through Hydrolysis

After the pre-treatment of lignocellulosic starting materials, a hydrolysis process is usually required for the separation of long carbohydrate chains from cellulose or starch. This saccharification through hydrolysis is typically catalyzed by an enzyme or an acid. This stage is critical in bio-EtOH production since the quality of the hydrolysate will affect the subsequent fermentation process,^50,67^ as yeast used in the fermentation process cannot utilize polysaccharides as a substrate for bio-EtOH production.^75,82,124^ Acid hydrolysis benefits from the low cost of chemicals and the efficient process of chemical reactions.^47,57,82^ However, the production of undesirable by-products that inhibit yeast growth in the following fermentation process and the requirement for acid recovery makes this method less favorable. Enzymatic hydrolysis is typically economically challenging due to the high cost of enzymes and is therefore often considered impractical for commercial purposes.^57,72,81,82,116^ However, in comparison with acid hydrolysis, enzymes work under milder conditions with simple processes, which requires less equipment maintenance cost. Moreover, enzymatic hydrolysis essentially is a biological process, thus implying the sustainability of this process. The use of different enzymes in the hydrolysis process has been thoroughly reviewed in existing works.^50,57,72,81,82,116^

##### 2.1.2.3 Fermentation

During the fermentation process, sugars are converted into bio-EtOH *via* the metabolic processes of microorganisms, such as bacteria and yeasts.^55,81,102,104,124^ Monosaccharides (e.g., glucose and fructose) or disaccharides (e.g., maltose and sucrose) can be metabolized without oxygen and produce bio-EtOH and CO_2_. For starchy hydrolysates, only glucose is present for the fermentation, presenting a simple substrate. However, different pentose and hexose sugars are present in hydrolysates of biomass and natural microorganisms cannot typically ferment pentose sugars, although different strains of yeast have been reported to ferment xylose and arabinose.^50,75,85,102^ Thus, a co-culture system is often employed in the fermentation of hydrolysates from lignocellulosic biomass.

Generally, five fermentation strategies can be applied for bioethanol production.^50^ A first strategy is cell recycling batch fermentation, a process in which the main objective is to adapt yeast to become inhibitors. This process increases yeast performance as it can shorten reaction time and decrease minor carbon deviation for cell production. Another strategy is the SSF method.^85,99,102^ In this process enzymatic hydrolysis and fermentation steps are applied together to obtain fermentable sugar to produce bio-EtOH. SSF has often been reported to increase the yield of bio-EtOH production, although the different ideal temperatures of the enzyme and the yeast can result in poor performance. A third strategy is separated hydrolysis fermentation (SHF), where hydrolysis and fermentation are conducted separately to produce bio-EtOH.^48,61,85,95,99,102^ Semi-simultaneous saccharification and fermentation (SScF),^95,99,102^ in which a short pre-hydrolytic step is applied before the SSF process presents a fourth fermentation strategy. Here, the bio-EtOH yield is typically slightly higher than with conventional SSF. Finally, consolidated bioprocessing (CBP) can be used for sugar fermentation, in which the decomposition of resistive biomass substrates into solubilized sugars is conducted alongside a metabolic intervention to guide the metabolic flow toward specific products with high yield.

The product of fermentation often is an azeotropic mixture of bio-EtOH and vinasse, making the separation of bio-EtOH another crucial part for obtaining high-purity bio-EtOH. Typically, this separation is done by distillation. The bio-EtOH obtained was typically distilled ethanol (93%), which is insufficient for subsequent processes that require high purity standards and may hence need to be subjected to additional purification.

##### 2.1.2.4 Dehydration

Many different solid-catalysts have been reported as efficient for this process, including metal oxides, acidic zeolites, phosphoric acids, and heteropoly acids, as well as bio-catalysts,^125−129^ as is summarized in Table 2.

**Table 2:** Examples of Catalyst for the Dehydration of Bioethanol to Ethylene

	Catalyst	Ethanol conversion (%)	Reaction temperature (°C)	Catalyst stability	Ref.
Acid catalysts	Phosphoric acid	61–100	50–275	Low	(128)
	Phosphotungsic acids	75	180–150	Low	(129)
	Silicoutungsic acids	52		Stable	
	Phosphomolybdic acids	11		Low	
	Phosphotungsic acids modified with cations	100	190–260	High	(128, 129)
	Silica supported heteropoly acids	99.8	60–100	High	(127, 131)
Molecular-sieve based catalysts	HZSM-5 modified with La and P	100	240–280	High	(132)
	Spherical catalyst with γ-Al_2_O_3_	99.5–100	350–450	High	(133)
	ZSM-5 modified with Zn and Mg	99.0	400	Stable	(134)
	Zeolite ZSM-5	99.0	400	Low	(125, 130)
	Zeolite HZSM-5	96.0	400	Low	(135, 136)
	Silicoaluminophosphate (SAPO)-34	90	220–320	High	(137, 138)
	H-ZSM-5/H_3_PO_4_	99	400–450	Stable	(139)
	H-ZSM-5/SiC	40–75	400	High	(135)
Oxide catalysts	γ-Al_2_O_3_	99.0	350–450	Stable	(140−142)
	Al_2_O_3_-MgO/syndol	97.0–99.0	450	High	(143)
	Transition metal oxide	42.0–99.6	200-500	High	(126)
	Na_2_O-Mn_2_O_3_/Al_2_O_3_	92	300	High	(144)

The catalytical endothermic dehydration of bio-ethanol to bio-ethylene is the crucial step to eventually obtain the renewable polyolefin derivative. A representative catalytical synthesis of bio-ethylene from bio-ethanol was reported by Mao and co-workers in 1989,^130^ where acidic ZSM-5 zeolite was employed as the catalyst. Only a dilute ethanol mixture, as low as 2%, could be used to produce ethylene.

The phosphoric acid catalyst was among the first catalysts to be used in the industrial synthesis of ethylene *via* catalytic ethanol dehydration.^128^ The catalyst was prepared by impregnating clay or carbon with phosphoric acid. Ethylene obtained with this catalyst was of high purity, but the catalyst underwent rapid coking.^134,137^ Hetero-polyacid catalysts are another kind of acid catalyst, which consist of strong, polybasic anionic complexes.^127,131^ The possibility of employing various hetero-polyacids and their salts possessing Brønsted acidity in alcohol dehydration has been investigated in a number of studies.^129,131,134,137^

Catalysts based on molecular sieves have also been employed in the catalytical dehydration of bio-EtOH. Molecular sieves have a porous structure, unique acid–base properties, and a large specific surface area, making them widely employed as adsorbents and catalysts. Various types of zeolites as catalytic systems in the dehydration of alcohols to olefins have been reported,^23,125,136,140,145^ including the use of natural zeolites and clays,^134^ as well as bulk or supported synthetic zeolites such as NaX,^140^ SAPO-34,^138^ ZSM-5,^125^ HZSM-5^146^ and supported HZSM-5s.^135,136,139,145,147^

The molecular sieves that have been intensively studied in the hydrogenation of ethanol to ethylene are those based on silicoaluminophosphate (SAPO) and ZSM-5. Pure zeolite ZSM-5 was reported in the dehydration of a 20% ethanol solution to ethylene at 400 °C with a 99% ethanol conversion and ethylene selectivity up to 80%. On reducing the reaction temperature, the conversion and ethylene selectivity significantly decreased to 42% and 72%, respectively.^134^ H-ZSM-5 zeolites were also studied for the dehydration of ethanol into ethylene, suggesting a direct single step method for ethanol conversion without a purification stage for obtaining high purity ethanol.^125,130^ At an ethanol concentration of 15% and a temperature of 400 °C, the ethanol conversion and ethylene selectivity were 96% and 49%, respectively. Introduction of zinc and magnesium cations into the H-ZSM-5 catalyst was shown to enhance olefin selectivity, while similar effects are reported on H-ZSM-5 catalysts modified with phosphoric acid.^139^

Several different parameters have been studied regarding the stability and efficiency of HZSM-5 catalysts. For example, the addition of water to ethanol was reported to enhance the stability of the H-ZSM-5 catalyst and its ethylene selectivity.^146^ Possible reasons for this include the reduced acidity of the catalytic sites in the presence of water and deactivation of the catalyst.^136,146^ Thus, although considerable catalyst deactivation is observed in the presence of water, ethanol dehydration with zeolite catalysts is mainly performed with a large excess of water while it is recommended that the process should be conducted at elevated temperatures. Other modification methods such as changing crystallite size,^136^ treating HZSM-5 with ion exchange to obtain the NKC-03A catalyst, and modifying ZSM-5 with lanthanum have also been reported.^132^ Composite systems of zeolite catalysts were also reported with objectives to decrease the required reaction temperature and enhance the catalyst stability.^23,136,145^ For example, a system employing titanium dioxide nanotubes as a deposition substrate of ZSM-5 catalysts has been reported,^134^ whereby increasing the effective concentration of acid sites, ethanol conversion and ethylene yield were improved. Some drawbacks of zeolite catalysts include the rapid deactivation, their structural complexity and their high costs.^23,125,136,145^

Molecular sieves based on Si–Al phosphates, namely SAPOs, have been developed since 1984.^138^ The ethylene dehydration properties of SAPO catalysts have been studied^138^ and their stability has been screened relative to HZSM-5 and alumina catalysts, identifying SAPO-34 to have reasonable stability over 100 h of reaction. Under certain conditions, the activity of SAPO-34 in ethanol dehydration was reported to be higher than that of zeolite ZSM-5. The use of SAPO- and HZSM-based composite catalysts has also been studied,^134^ with the composite catalysts system having increased acidity and catalytic activity at lower temperatures. The disadvantage of SAPO-type catalysts includes being quite expensive, considering the minor improvement in performance.

Metal oxides are another group of catalysts used frequently in the study of ethanol dehydration to ethylene. Examples of oxide catalysts include alumina,^141^ magnesium oxide,^148^ cobalt oxide,^148^ chromium oxides^149^ and transitional metal oxide catalysts.^126^ Other mixed or supported systems have also been reported.^144,148,150,151^ One representative example is aluminum oxides (alumina), which can serve as both a catalyst and support. This kind of catalysts was mainly used in commercial scale ethanol dehydration to ethylene.^133,134,141,152^ The advantage of these catalysts is their improved stability compared to clay-based catalysts, but they originally suffered from producing low purity ethylene. The development of γ-Al_2_O_3_ catalyst has resolved this issue, showing a high ethanol conversion over 95% with ethylene selectivity over 96%.^134^ Composite systems of alumina combined with silicon or silicon dioxide can enhance the stability, activity and selectivity of the catalyst.^145^ Other modified systems such as syndol,^134^ iron oxide^153^ or zirconium oxide^134^ composites were also reported as more active systems in ethanol dehydration compared to individual alumina. However, excessive acid sites in composite alumina catalyst systems result in undesired catalytic properties. It was also demonstrated that modifying alumina with chlorine ions can result in a considerable increase in the number of weak electron-acceptor sites, which enhances the catalyst activity. Apart from doping the catalyst system with other elements, increasing the specific surface area can also improve the performance of alumina catalysts. For example, mesoporous alumina catalysts with 1-20 nm pore sizes can exhibit an ethanol conversion of 99% and an ethylene selectivity of 98%.^152^ Additionally, the granule shape and pore structure of alumina catalysts has also been demonstrated to affect the catalytic properties.^133^

In industrial ethanol dehydration units, individual alumina catalysts have been most employed due to the high-performance stability of the catalyst along with the high ethanol feed concentration that can be tolerated. More recently, modified oxide catalysts or composite catalytic systems have enabled improvement in outputs like ethylene selectivity and ethanol conversion.^23,143,148−150^ However, for the sustainable production of bio-ethylene, the energy and economic cost of the preparation of catalysts also needs to be taken into account. Thus, only relatively straightforward techniques such as thermal activation of alumina catalysts seems promising for industrial use at this stage^134^ and the development of cost-efficient methods of modifying alumina catalysts or designing composite catalytic system could be rewarding in the field of catalytic ethanol dehydration.

#### 2.1.3 Polymerization of Bio-ethylene

Although numerous examples have been reported on the production of bio-ethanol from renewable feedstock and its catalytical dehydration into ethylene,^32−34,36,48^ very few specifically demonstrated the subsequent polymerization of bio-ethylene into bio-polyethylene.^125,130^ Most studies focused on the application of bio-polyethylene as the matrix to produce renewable composites with fillers such as natural fibers^154,155^ and clays.^156^ For example, bio-based composites of bio-ethylene and thermo-mechanical pulp corn stover fibers have been reported.^155^ After the creation of a fiber-matrix interface *via* the addition of a coupling agent, the tensile strength of the composites increased over 100% for a 40 wt % reinforcement, when compared to unreinforced bio-polyethylene. The composites with other renewable polymers or fillers are often referred to as bio-composites.

Although commercialized renewable polyethylene has been used in markets,^157−159^ the cost-efficiency of bio-polyethylene is still low compared to petrochemically derived polyethylene. Thus, the improvement of the cost-efficiency and the development of novel treatment procedures to produce bio-ethylene from bio-ethanol is deemed crucial for renewable polyethylene-based commodity plastics production. Alternatively, instead of preparing bio-polyethylene from previously described methods, the development of polyethylene-like materials whereby degradable linkers are introduced into the polyolefin backbone has received significant attention.^29,30,160,161^ While such materials currently possess promising degradability, and hence chemical recyclability, their properties often differentiate too much from those of polyethylene in order to present a scalable alternative.

### 2.2 Propylene

**Figure 3:**
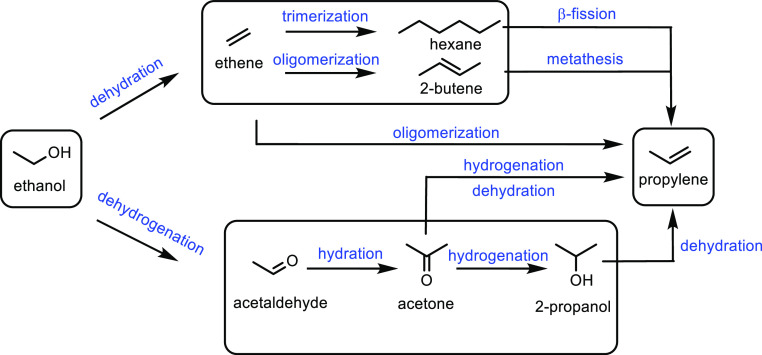
Possible pathways of production of propylene from ethanol.

Compared to the reported production of renewable polyethylene, the synthesis of bio-polypropylene remains rather limited.^21,162−165^ While propylene is conventionally obtained from the steam cracking of crude oil, a very recent review^162^ addressed the potential approaches for producing bio-polypropylene through the catalytic conversion of methanol or ethanol to propylene. Considering the wealth of research on bio-EtOH production previously mentioned and existing reports on bio-methanol (bio-MeOH) production, methanol-to-propylene (MTP) and ethanol-to-propylene (ETP) conversions have potential to serve as sustainable routes of bio-propylene.^162,163,166^ In the MTP process, propylene is mostly produced as a by-product, with over 80% of ethylene as the major product.^162,165^ However, as Koempel *et al*. reported, the production of olefin mixtures with predominantly propylene could be achieved *via* a catalytical MTP process.^163^ Catalytic pyrolysis of biomass presents another potential route for producing bio-propylene, as the pyrolysis of biomasses results in a complex mixture that contains propylene.^23,28,167^ The pyrolysis of biomass is a two-step process which contains fast pyrolysis and pyrolysis vapor conversion, for which catalysts such as ZSM-5 and calcium oxide have been studied.^28,167^ The bio-ethanol dehydration, dimerization and metathesis procedure towards bio-propylene is the most researched approach, but additional pathways have been investigated (Figure 3). Different catalysts have been utilized in the ethanol dehydration process, with reported propylene yields ranging from 2% to 32%.^162^ The use of a vast amount (>120 examples) catalysts for bio-propylene production has been reviewed in an excellent report elsewhere,^162^ where factors impacting the propylene yield, such as the addition of metals and non-metals, are discussed in detail. Reaction conditions influencing the reaction efficiency include temperature, pH, water content present in the ethanol mixture, pressure, and the modification of catalysts with metal additives, non-metal additives, varied particle size, and varied component ratio. As mentioned earlier, the high cost of zeolite catalysts forms a concern in view of the commercial valorization of the ETP process. Considering the low yield of propylene as a major product, other procedures or catalysts that increases propylene selectivity could of interest to promote the renewable production of bio-propylene. Furthermore, no reports on the use of bio-propylene as monomer feed to synthesize polypropylene appear to be available to date.

### 2.3 Butylene

**Figure 4:**
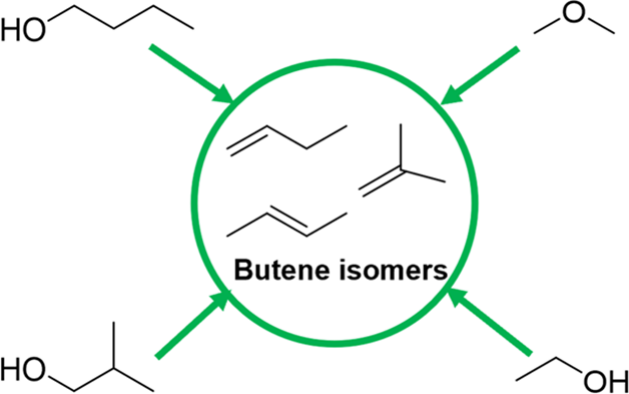
Various reported starting materials to produce butene isomers.

The renewable production of other olefins such as butylene is also rather undeveloped as compared to the production of bio-ethylene. Nonetheless, few reports are available on the production of bio-butylene *via* different methods, including the catalytical dehydration of butanol, the by-production from biomass and the conversion of ethanol (Figure 4).^103,147,168−172^ However, butene is mostly obtained as a mixture of different isomers in these examples. Perhaps most notable is the highest selective (i.e., 99%) synthesis toward butene through the catalytic conversion of ethanol using designated metal doped oxide catalysts.^172^ Different zeolite and zirconia-based catalysts have also been investigated, giving mixtures of 1-butene, *cis*/*trans*-2-butene and dibutyl ether from the catalytical dehydration of 1-butanol, while the highest selectivity toward 1-butene was reported to be 40%.^147^ Evidently, the purification of butene isomer mixtures prior to its subsequent polymerization is an important challenge that needs to be addressed for the large scale production of butene monomers from renewable sources.

Aside from the plain olefin polymers, the sustainable preparation of other important polyolefins from bio-based sources is still in its infancy. For example, poly(vinyl chloride) (PVC) is the third most widely produced synthetic polymer, though a sustainable preparation of the vinyl chloride monomer is yet to be reported.

### 2.4 Styrene

First isolated in the 1830s, styrene is an aromatic monomer that is widely used in the production of polystyrene, polyesters, protective coatings, resins and rubbers.^173^ The global production of styrene in 2017 was 15 million tons, with a market value of $43.1 billion.^173^ Styrene is produced industrially through the dehydrogenation of ethylbenzene, which in turn is derived from crude oil.^173,174^ Furthermore, dehydrogenation of ethylbenzene is a very energy intensive process, often requiring temperatures of 500–600 °C. In addition, the oxidative dehydrogenation of ethylbenzene is less intensive than simple dehydrogenation, and the various catalysts used in the oxidative dehydrogenation of bio-based ethylbenzene will be described. Hence, interests in a more sustainable production of ethylbenzene are highly desired.

Apart from bio-sourced ethylbenzene, a process focusing on the metathesis of cinnamic acid and cinnamate esters to produce styrene and acrylic acid/alkyl acrylates, called ethenolysis, will be highlighted as a different method for production of styrene. Also, the production of functional styrenics from bio-based chemicals such as cinnamic acids, ferulic acid, sinapic acid, coumaric acid and vanillin will be explored in this section. Apart from chemically alternative routes, styrene production through biochemical processes from phenylalanine using *Escherichia coli* will also be discussed.

#### 2.4.1 Styrene from Biobased Ethylbenzene

##### 2.4.1.1 Formation of Ethylbenzene from Lignin

One way to increase the sustainability of styrene is to obtain the ethylbenzene starting material from biomass instead of petrochemicals. Lignin is an abundant and renewable source of biomass and the highly aromatic nature of lignin gives rise to its potential use in the production of aromatic chemicals.^175,176^ Furthermore, ethylbenzene can be produced from the catalytic thermal degradation of lignin.^177^ Li and co-workers produced ethylbenzene from lignin *via* a two-step process.^178^ The first step was the depolymerization of lignin over a Re-Y/HZSM-5(25) catalyst. The major product of the depolymerization was benzene, and while at lower temperatures (i.e., 500 °C) the selectivity of benzene (measured in carbon-mol %) was poor (i.e., 26 C-mol %), it increased significantly to 90 C-mol % when the reaction temperature was increased to 650 °C. The resulting aromatic products were eventually reacted with ethanol over the catalyst HZSM-5(25) to form ethylbenzene. This reaction is reported to have occurred in two stages, the first being the dehydration of ethanol, producing ethylene and water, while the second step is the ethylation of benzene. While increasing the ratio of ethanol in the reaction increased benzene conversion, it also decreased the product selectivity. For example, when the feed to ethanol ratio was 2:1, the benzene conversion was 41% and the ethybenzene selectivity was 72 C-mol %. When the ratio was 1:2, the conversion of benzene increased to 68% but the product selectivity was only 43%. Production of ethylbenzene *via* this method has the advantage of using bio-based lignin and ethanol, which can be derived from sustainable sources. However, high temperatures are required for the depolymerization of lignin to proceed with high selectivity and there are also selectivity issues for the ethylation of benzene, which currently limit its sustainability.

Other studies have focused on screening different catalysts for the production of aromatics from lignin.^179−181^ Wang and co-workers investigated Zn and Ga loaded catalysts, and obtained an optimum hydrocarbon aromatic yield of 37 wt % and a BTEX (benzene, toluene, ethylbenzene and xylene) selectivity of 62 wt %.^181^ A different approach was explored by Luo and co-workers, investigating the formation of ethylbenzene from lignin.^180^ The first step was the hydrodeoxygenation of lignin to form C3–C7 alkanes (20%), C8 ethylcyclohexane (42%) and cyclic C9–C17 alkanes (38%). A nickel/silicalite-1 catalyst was used with a reaction temperature of 300 °C and a hydrogen pressure of 6 MPa. The ethylcyclohexane was separated *via* distillation before dehydrogenation over a Pt-Sn/Al_2_O_3_ catalyst at 500 °C and 0.5 MPa H_2_ resulted in a 99% yield of ethylbenzene. Separately, a modified FeO_*x*_ Ru/Nb_2_O_5_ catalyst has also been used for the selective production of ethylbenzene from birch oil lignin through a dehydrogenative decarbonylation and hydrogenation process, obtaining a 63% selectivity.^179^ While these methods require lower temperatures than for the depolymerization of lignin, harsh temperatures and metal catalysts are still required to achieve high yields, thus limiting the sustainability of these processes.

##### 2.4.1.2 Conversion of Ethylbenzene into Styrene

The ethylbenzene obtained from biomass can then be dehydrogenated to produce styrene. The dehydrogenation of ethylbenzene requires high operating temperatures of 500–600 °C, which decreases the selectivity of the reaction.^182^ In comparison, the oxidative dehydrogenation is exothermic and has a more favorable equilibrium. The reaction has been carried out previously using mild oxidizing agents such as O_2_, SO_2_ and CO_2_.^173,183,184^ Sugino and co-workers used CO_2_ as a mild oxidizing agent, having the advantage of consuming a greenhouse gas, which increases the overall environmentally friendly aspect of this process.^173,183,184^ Using CO_2_ also has several other advantages, as it is cheap and abundant, increases catalyst selectivity by poisoning non-selective sites in the catalyst, and improves the rate of reaction.^184−186^ Oxidative dehydrogenation proceeds through two steps whereby H_2_ is produced in the first dehydrogenation step and then reacts with CO_2_ to form water and carbon monoxide in a reverse water gas shift reaction.^173,187,188^ The activation of CO_2_ onto catalyst surfaces is an important step in the oxidative dehydrogenation reaction. While catalysts can activate CO_2_ themselves, activators such as alkali or alkaline metals can be used.^183,189,190^ Many different catalysts have been explored, including catalysts based on vanadium, mixed metal oxides and nanocarbon structures (Table 3).^173^

**Table 3:** Different Catalytic Systems for the Formation Styrene from Biobased Ethylbenzene

Entry	Catalyst	Temperature (°C)	Conversion (%)	Selectivity (%)	Yield (%)	Ref.
**1**	Al_2_O_3_	600	62	92	57	(191)
**2**	0.8 V/OMA	550	52	>97	50	(192)
**3**	V/TiO_2_-Al_2_O_3_	550	65	96	62	(193)
**4**	V_2_O_5_/HMS	600	73	99	72	(194)
**5**	MnO_2_-ZrO_2_	650	74	98	72	(195)
**6**	Na/TiO_2_-ZrO_2_	600	72	97	70	(186)
**7**	K_2_O/TiO_2_-ZrO_2_	660	72	98	71	(196)
**8**	MoO_3_/TiO_2_-Al_2_O_3_	650	77	97	75	(197)
**9**	Co_3_O_4_/MgAl_2_O_4_	600	82	98	80	(198)
**10**	CeO_2_/ZrO_2_/SBA-15	650	67	93	62	(199)
**11**	Carbon molecular sieve	330	80	90	72	(200)
**12**	Carbon nanotube 3	450	28	68	19	(201)
**13**	Carbon multiwalled nanotube	400	47	91	43	(202)
**14**	Nanodiamond	450	40	92	37	(203)

Vanadium catalysts have been heavily investigated for use in oxidative dehydrogenation reactions.^192,204^ Vanadium catalysts were of particular significance because the V–O bond is effective at interacting with the alkyl protons, from the ethyl group of ethylbenzene, assisting in their abstraction. While vanadium and chromium oxides supported on alumina showed similar results to bare alumina (Table 3, entry 1), vanadium oxides on other supports have shown increased catalytic performance. For example, Li and co-workers compared vanadium catalysts supported on ordered mesoporous alumina (OMA) (Table 3, entry 2) and γ-alumina.^192^ The OMA support showed a styrene selectivity of >97% and a stable conversion of 52% from ethylbenzene was obtained for 12 h. In contrast, a catalyst with the same loading of vanadium on a γ-alumina support showed a high ethylbenzene conversion in the first 2 h before significant deactivation took place. The results suggested that the support plays an important part in stabilizing the vanadium catalyst. Furthermore, OMA is more effective than y-alumina at stabilizing the active V^5+^, while also suppressing its reduction to the less active V^3+^ form due to the stronger interaction between OMA and the catalyst compared to γ-alumina. A hybrid TiO_2_–Al_2_O_3_ support was used to obtain a styrene yield of 62% (Table 3, entry 3).^193^ The hybrid support was effective at overcoming the limiting surface area of the active TiO_2_ by combining it with the higher surface area Al_2_O_3_. The best support was shown to be hexagonal mesoporous silica (HMS).^194^ At an optimum catalyst loading of 20 wt %, a maximum conversion of 73% and selectivity of 99% were achieved (Table 3, entry 4). The V_2_O_5_ catalysts supported on HMS were the most selective vanadium-based catalyst, and this can be attributed to the high surface area and the unique sponge-like particle texture of the support, which favors its application in heterogenous catalysis.^205^ However, it is noteworthy that the synthesis of HMS supports has been reported to be energy intensive and time consuming, potentially limiting the potential of this catalytic system.^194^

Mixed metal oxide catalysts have also been effective for the oxidative dehydrogenation reaction of ethylbenzene, offering several advantages including stabilized conversions, selectivity, and reduced coke formation.^206^ There are many examples of different mixed metal oxide catalysts that have been used to obtain high conversions and styrene selectivity (Table 3, entries 5–10).^186,195−199^ Of those, molybdenum oxide- and cobalt oxide-based catalysts resulted in the best styrene yields of 75% and 80%, respectively.^197,198^ Nagaraja and co-workers developed a MoO_3_/TiO_2_-Al_2_O_3_ catalyst that achieved a conversion of 77% and styrene selectivity of 97% after 1 h of reaction at 650 °C, with an optimum molybdenum loading of 7.5 wt % (Table 3, entry 8).^197^ Further doping with molybdenum decreased the ethylbenzene conversion and the catalyst deactivated over time, with the conversion and selectivity falling to 50% and 85% after 40 h, respectively. The performance of this catalyst was attributed to the high surface area and the active Mo^6+^ species being effectively stabilized by the TiO_2_–Al_2_O_3_ support. Rao and co-workers developed a Co_3_O_4_/MgAl_2_O_4_ catalyst that achieved an even higher styrene yield of 80% (Table 3, entry 9).^198^ The higher yield was attributed to the synergistic interface between the active catalyst and support leading to an increased active surface. The importance of the interaction with the support was shown by a significant decrease in the conversion when MgO and γ-Al_2_O_3_ supports were doped with Co_3_O_4_.

One of the major disadvantages of the reported catalytic systems is their deactivation due to coke formation or catalyst reduction. Coke formation has been attributed to several side reactions, including ethylbenzene cracking and styrene oligomerization reactions.^207^ When a γ-alumina support was used, deactivation of the catalyst was observed due to coke formation.^191^ This deactivation was limited by adding a thin carbon layer, with an optimum loading of 18.6 wt %.^208^ A higher wt % reduced the styrene selectivity and ethylbenzene conversion, as it changed the textural properties of the catalysts. Again, the nature of the support was shown to have an impact on catalyst deactivation. For example, deactivation is more severe for γ-alumina because of its smaller surface area, pore volume and pore diameter causing them to become blocked by coke deposition more easily, when compared to the ordered mesoporous alumina.^192^ Reduction of the catalyst also leads to catalytic deactivation. In the oxidative dehydrogenation process, the released hydrogen from ethylbenzene reacts with lattice oxygen to release water, leading to a reduction of the catalyst activity. Carbon dioxide plays an important role in removing the hydrogen through the reverse water gas shift reaction. It has been reported that CeO_2_ is better at protecting the lattice oxygen than Fe_2_O_3_ and V_2_O_5_, consequently reduction of the catalyst is limited.^209^ The CeO_2_ doped catalyst was found to improve the acid–base properties of the catalyst, which are important for catalysis. Burri *et al*. developed a 25/25CZS catalyst (containing CeO_2_, ZrO_2_ and 75 wt % of a SBA-15 support) for the oxidative dehydrogenation of ethylbenzene, obtaining a styrene yield of 62% after 5 h (Table 3, entry 10).^199^ The high activity of the catalyst was attributed to the active CeO_2_–ZrO_2_ catalyst being dispersed on a high-surface-area support. The styrene selectivity remained around 93% during the 10 h reaction time, while the conversion fell slightly to 64%, indicating good catalyst stability.

Cheaper alternatives to metal oxide catalysts have been sought for styrene production, such as graphene, carbon nanotubes or nano-diamond (Table 3, entries 11–14).^173,189,210,211^ As well as being less expensive, these carbon-based catalysts also offer other advantages when compared to metal-based catalysts. For example, ultra-high-surface-area molecular sieves have been used as catalysts (AX-21) for the oxidative dehydrogenation of ethylbenzene, obtaining a conversion of 80% and a styrene selectivity of 90% (Table 3, entry 11).^200^ While the selectivity of this catalyst is lower than the majority of metal-based catalysts, there are two advantages to AX-21. First, the conversion of ethylbenzene is very high (i.e., 80%) compared to when metal-based catalysts were used. Second, a significantly lower reaction temperature had to be used when using AX-21 (i.e., 330 °C) compared to temperatures >550 °C that were required when using metal-based catalysts. The high activity of AX-21 was accredited to the high surface area of the catalyst, which was reported to be 3000 m^2^ g^–1^. As well as molecular sieves, carbon nanotubes have also been used as non-metallic catalysts for styrene production from ethylbenzene.^201^ Nonetheless, carbon nanotubes require an oxidative pre-treatment which improves their conversion potential from 20% to 28%. This highlights the importance of oxygenated groups on the catalyst surface. However, carbon nanotubes proved not as effective as activated carbon (42% styrene yield) and metal-based catalysts (50–80% styrene yield).

Oxidative treatments are used to introduce oxygenated functional groups, for example, carboxylic, carbonyl and hydroxyl groups, at the surface of carbon nanotubes.^212,213^ These treatments can include gas phase oxidation, using a range of oxidants including air, steam or a mixture of Cl_2_, HCl and H_2_O.^212,214−216^ Liquid phase oxidation involves the use of oxidizing agents such as HNO_3_, H_2_O_2_, KMnO_4_ or a mixture of H_2_SO_4_, HNO_3_, KMnO_4_ and NaOH.^212,217−219^ However, using strong acids and oxidants can destroy the carbon nanotube structure, hence why the milder nitric acid is the most common oxidizing agent used in these treatments.^212^ When multiwalled carbon nanotubes were subjected to an oxidative pre-treatment utilizing UV light and H_2_O_2_, an ethylbenzene conversion of 47% and a styrene selectivity of 91% was obtained (Table 3, entry 13). Temperature was also shown to effect catalytic performance due to a decrease in the number of oxygenated groups as the temperature was increased from 700 to 3000 °C.^220^ This correlated with a decrease in catalytic activity as shown by a decrease in conversion from 50% to less than 3% for temperatures of 700 and 3000 °C, respectively.

Nanodiamonds are a less frequently studied class of carbon nanomaterial and have unique characteristics and properties, including a unique sp^3^/sp^2^ structure, high thermal and chemical stability and a high surface area.^221^ Nanodiamond catalysts were reported to be more active and selective than other forms of carbon catalysts including carbon nanotubes and activated carbon.^222^ The increase in activity originates from the sp^3^/sp^2^ structure giving rise to a variety of different defects and surface functional groups. These carbon nanodiamonds have been combined with carbon nitride to obtain catalysts that demonstrated 99% styrene selectivity.^223^ Nanodiamonds can be used to combine both dehydrogenation and oxidative dehydrogenation, demonstrating an ethylbenzene conversion of 40% and 92% styrene selectivity in oxygen-lean conditions (Table 3, entry 14).^203^ Furthermore, the sp^3^/sp^2^ structure remained intact and active ketone functionalities were regenerated *in situ*. The oxygen-lean conditions gave a superior ethylbenzene conversion than direct dehydrogenation (5.5%), while the selectivity was slightly lower than for the direct dehydrogenation (i.e., 98%). Adding more oxygen increased the activity of the catalyst, as more oxygenated functional surface active groups were present, but this also led to a decrease in selectivity and catalyst stability. Finally, carbon nanotubes and nanodiamonds have been combined into a single catalytic system, demonstrating high styrene selectivity (i.e., 98%) but low ethylbenzene conversion (i.e., 18%).^224,225^

#### 2.4.2 Biochemical Routes to Styrene

Biochemical routes are important when considering sustainable reactions as they remove the need for metal catalysts and high operating temperatures.^226^ Styrene has been produced by the fermentation of glucose using *E. coli*; however, the yield is limited by the toxicity of styrene toward *E. coli*, which is predicted to be around 300 mg L^–1^.^227,228^ The solubility of styrene in water is only 320 mg L^–1^ at 32 °C, which is similar to fermentation conditions. Thus, optimizing conditions such that styrene can phase separate from the cultures would help to alleviate the associated toxicity problem.^227^ Furthermore, if spontaneous phase separation could be achieved, the produced styrene would be high purity due to water being very insoluble in styrene.

**Figure 5:**
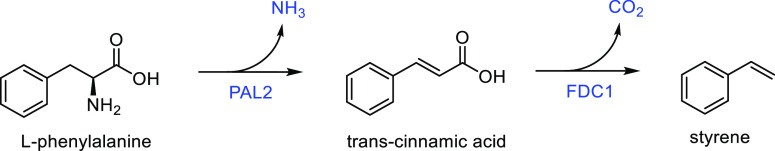
Synthesis of styrene from l-phenylalanine *via* a biochemical approach.

McKenna and co-workers were the first to synthesize styrene from biomass using an engineered microbial platform in 2011.^229^ The engineered *E. coli* was able to generate styrene with a titer of 0.26 g L^–1^ by converting glucose into l-phenylalanine, *trans*-cinnamic acid and then styrene. Nevertheless, problems such as low enzyme activity and styrene toxicity were encountered. The titer could be improved to 0.35 g L^–1^ when an *in situ* product removal approach was taken using isopropyl myristate as a solvent.^230^ Using a solvent extraction method to reduce the impact of styrene toxicity with bis(2-ethylhexyl)phthalate generated a titer of 0.84 g L^–1^.^231^ The titer was increased further by Lee *et al*. who optimized the production of styrene by using *E. coli* YHP05 and the coenzyme ScFDC along with an *in situ* product recovery.^232^ An optimum titer of 1.7 g L^–1^ was obtained for the shake flask production, while when in a liter scale fed-batch reactor, a titer of 5.3 g L^–1^ was achieved. Grubbe and co-workers produced two enzymes *in situ via* cell-free protein synthesis.^233^ These two enzymes, phenylalanine ammonia lyase 2 (PAL2) and ferulic acid decarboxylase 1 (FDC1) were used to convert l-phenylalanine into styrene in a two-step synthesis with a titer of 4.2 g L^–1^ (Figure 5). In the first step, PAL2 catalyzed the transformation of l-phenylaniline to *trans*-cinnamic acid through the loss of ammonia.^234−236^ The *trans*-cinnamic acid then undergoes decarboxylation to form styrene, catalyzed by the FDC1 enzyme.

The biochemical production of styrene has many benefits including being less energy intensive, producing fewer toxic products and reducing greenhouse gas emissions. Nonetheless, the biochemical pathways to styrene cannot produce styrene currently on a large enough scale to meet demand, meaning further research is required to become industrially viable.^237^

**Figure 6:**
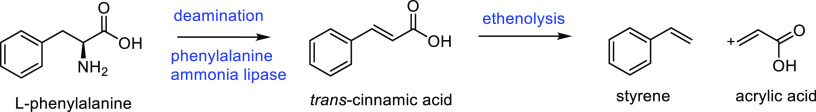
Two-step synthesis of styrene and acrylic acid from phenylalanine *via* deamination and ethenolysis.

The *trans*-cinnamic acid produced *via* the biochemical reaction of l-phenylalanine with PAL2 can eventually be reacted in a metathesis reaction, called ethenolysis, with ethene to produce styrene. The products of this reaction are styrene and acrylic acid, two valuable vinyl monomers (Figure 6). Alkyl acrylates could also be produced when cinnamate esters were used instead of cinnamic acid.^236^ For the ethenolysis of all substrates tested, the best catalyst was the Hoveyda–Grubbs second generation catalyst. This was attributed to the electron donating carbene ligand stabilizing the electron deficient double bond in the substrate. However, this reaction proceeded with limited yields. The styrene yield when cinnamic acid was used was 29%, while all the cinnamate esters tested obtained lower yields between 12 and 23%. This ethenolysis was more challenging than previous examples reported because of the double bond in the substrate being electron deficient.^236,238^ In previous work, increasing the pressure of ethene generally led to an increase in the conversion; however, the reverse was true for this ethenolysis.^236,238,239^ It was suggested that this was due to the double bond being electron deficient, and at high pressures ethene would predominantly undergo self-metathesis.^236^ At lower pressures, there is less ethene in the system for this side reaction to take place, the cross metathesis with cinnamic acid/esters was favored.^236^ However, the disadvantage of using lower ethene pressures is that the self-metathesis of styrene to form stilbene occurs at a faster rate. In an ethenolysis of styrene with 20 bar ethene the ratio of styrene to stilbene was 83:17 while at 1 bar pressure the selectivity fell to a ratio of 57:43.^236^ This trend was attributed to a higher selectivity being achieved when a more reactive metathesis substrate is used in excess.^236,240^ While ethenolysis has the advantage of producing two important monomers for the polymer industry, low yields and selectivity problems hinder its feasibility, while the use of ethene limits the sustainability aspect of this process unless it is also sustainably sourced.

#### 2.4.3 Functional Styrene Derivatives

**Figure 7:**
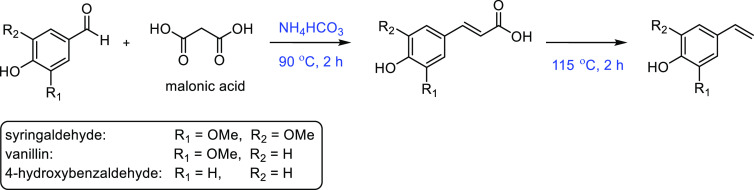
Synthetic route to produce 4-vinylphenol derivatives from syringaldehyde, vanillin and 4-hydroxybenzaldehyde.

Biobased alternatives to styrene are of interest and a series of functional styrenics featuring phenolic and methoxy groups have been synthesized. While the synthesis of styrene requires very high operating temperatures, the synthesis of functional styrenics operates at significantly lower temperatures. Functional styrenics possess a similar structure to styrene and can thus mimic the properties of styrene. The structures of some of the bio-based starting chemicals, derived from lignin, used are displayed in Figure 7 and include syringaldehyde (**1a**, Table 4), vanillin (**1b**, Table 4) and 4-hydroxybenzaldehyde (**1c**, Table 4). Meuldijk and co-workers produced functionalized styrenics through a two-step synthesis as shown in Figure 7, while the key results are summarized in Table 4.^241^ The first step of the synthesis was a green Knoevenagel reaction between the aldehyde functionality of the bio-reactants with malonic acid to form cinnamic acids, using ammonium hydrogen carbonate as a catalyst and a temperature of 90 °C. The next stage of the synthesis involved the decarboxylation of the α,β-unsaturated carboxylic acids to form the 4-vinylphenol derivatives. High overall yields of 91%, 92% and 90% were obtained for substituted styrenics produced from syringaldehyde, vanillin and 4-hydroxybenzaldehyde, respectively. Acetylated versions of the monomers were also formed *via* reactions between the 4-vinylphenol monomers, acetic anhydride, and sodium acetate at 90 °C for 30 min. The acetylation proceeded with high yields (>98%) for all monomers. This process has the advantage of using lignin derived starting materials as well as the naturally occurring malonic acid. It is also noteworthy that the temperatures required for this reaction are significantly lower than to produce styrene from the dehydrogenation of ethylbenzene.

**Table 4:** Reaction Conditions and Results for the Two-Step Synthesis of 4-Vinylphenol Derivatives from Syringaldehyde, Vanillin and 4-Hydroxybenzaldehyde

	Step 1			Step 2				
Substrate	Temperature (°C)	Reaction time (h)	Yield (%)	Temperature (°C)	Reaction time (h)	Yield (%)	Overall yield (%)	Ref.
**1a**	90	2	93	115	2	98	91	(241)
**1b**	90	2	95	115	2	97	92	(241)
**1c**	90	2	94	115	2	96	90	(241)

**Figure 8:**
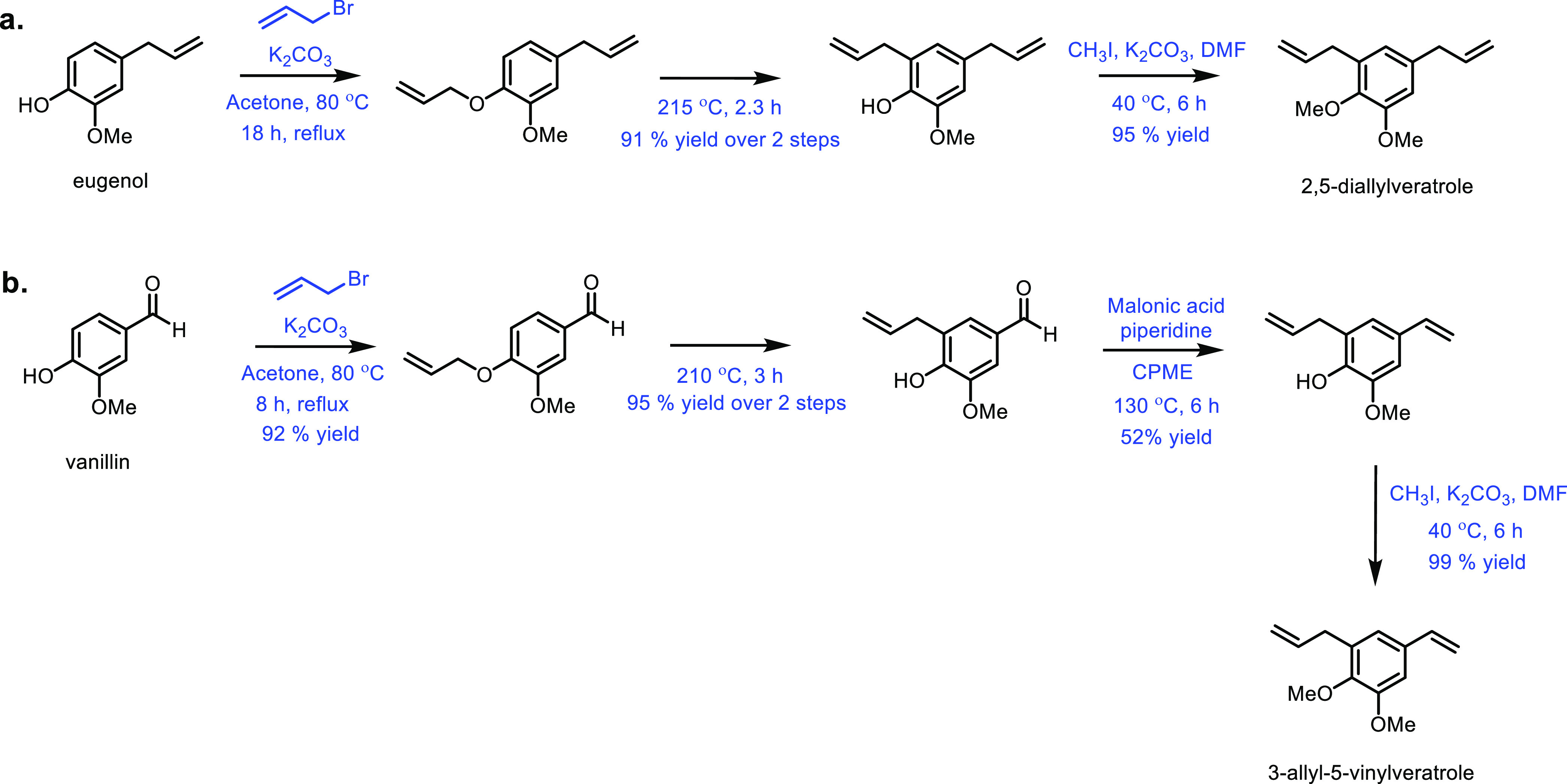
Multi-step synthesis of DV and AVV from eugenol and vanillin, respectively.

Eugenol is another bio-based aromatic chemical derived from lignin that can be used to synthesize functional styrene. For instance, Yu and co-workers synthesized two aromatic monomers featuring two double bonds, i.e., 2,5-diallylveratrole (DV) and 3-allyl-5-vinylveratrole (AVV).^242^ The synthetic routes for both monomers from eugenol and vanillin respectively are illustrated in Figure 8. A three-step synthesis from eugenol resulted in the formation of 2,5-diallylveratrole. The first step was the allylation of eugenol with allyl bromide and potassium carbonate in acetone, followed by a distillation which resulted in an aromatic Claisen rearrangement. The yield of these two steps was 91%, with the final step being a methylation with potassium hydroxide and iodomethane in a yield of 95%. The second monomer, 3-allyl-5-vinylveratrole, was synthesized in a slightly adjusted synthetic route, using vanillin as the starting material instead of eugenol. The first two steps were the same as for the previous monomer and achieved an overall yield of 95%. An extra step compared to the eugenol synthesis was required to convert the aldehyde group of vanillin to a vinyl group and was completed using a Knoevenagal condensation with malonic acid and piperidine in cyclopentylmethyl ether. A lower yield of 52% was reported for this additional step before the final methylation step proceeded with a 99% yield. These monomers have been shown to successfully replace styrene as the reactive diluent in vinyl ester thermoset networks, hence producing (partially) bio-based thermosets.^242^ The most promising result was replacing styrene with 3-allyl-5-vinylveratrole (>90% bio-based from this synthetic method), which matched the performance of styrene when formulated at 30 wt % of the diluents.

**Figure 9:**
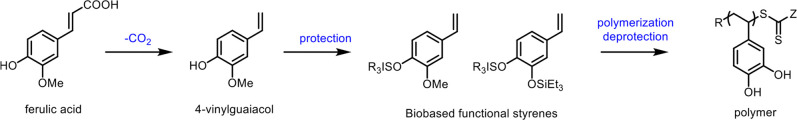
Synthesis, protection and polymerization of bio-based styrenics derived from ferulic acid.

Youngblood and co-workers synthesized derivatives from 4-vinylguaiacol including 2-methoxy-4-vinylphenyl acetate and 2-methoxy-4-vinylphenyl oleate.^243^ These resulting monomers were tested as potential styrene replacements in polyester thermoset networks. The protection of 4-vinylguaiacol was necessary, however, as phenolic compounds act as inhibitors in radical reactions.^243,244^ Moreover, a general trend was observed in which increasing the fraction of the bio-based styrene incorporated into the polymer led to a detrimental impact on at least one of the properties investigated (i.e., mechanical, thermal and water-uptake properties). Another bio-monomer using different protecting groups, 2,3-dimethoxystyrene, outperformed the others; however, it did lead to problems such as fast gelation and low polyester solubility. Nevertheless, a 50:50 mix of bio-styrene to styrene resulted in polymers with equivalent physical properties to the plain styrene polymers. The use of silyl protecting groups further allowed the bio-styrene to be polymerized with conversions of >95% (Figure 9).^244^ Similar polymerizations have been carried out for protected vinyl catechols, which were formed *via* a decarboxylation of caffeic acid followed by protection, with yields of 74–98% being achieved.^245,246^ The protected monomers were shown to be successfully polymerized *via* anionic and several controlled radical polymerization techniques. The anionic polymerization of a TBDMS protected vinyl catechol achieved the greatest success with near-quantitative conversion (i.e., 99%), molecular weight of 14 100 Da and a polydispersity index of 1.04.^245^

Cinnamic acids are another class of substrate that can be conveniently converted into functional styrenics *via* both biochemical and chemical routes. Cinnamic acid can be converted into 4-hydroxystyrenes using enzymatic decarboxylation. Previous work demonstrated that steric hindrance is an important factor that can prevent binding to the enzyme.^247^ This was shown, for instance, through *p*-coumaric acid having the highest rate of CO_2_ evolution (91 μL in 30 min) compared to the more sterically hindered substrates, e.g., caffeic acid (68 μL in 30 min) and ferulic acid (26 μL in 30 min). More sterically hindered substrates such as sinapic acid and substrates without a 4-hydroxy group (e.g., cinnamic acid, *o*- and *m*-coumaric acids) showed no CO_2_ evolution after 30 min.^247^ The microwave heated base-catalyzed decarboxylation of different cinnamic acids has also been investigated.^248^ As with the enzymatic route explored by Finkle, the 4-hydroxy position was vital for the reaction to proceed. In this case, there were two competing reactions, being the formation of bio-styrene and the formation of an amide. The optimized yields of styrene obtained from ferulic acid and *p*-coumaric acid were both 63% and proceeded with selectivity for only the bio-styrene. Other hydroxy-substitution patterns gave either lower yields of bio-styrene, or poor selectivity favoring the formation of the amide. Liu and co-workers used *N*-heterocyclic carbene precursors to catalyze the decarboxylation of cinnamic acids.^249^ Once again, the substitution pattern was an important parameter in determining the success of the reaction with the highest yields being obtained for ferulic acid (100%), sinapic acid (100%) and caffeic acid (97%).

Cadot *et al*. synthesized bio-styrene in a one pot synthesis by heating different cinnamic acids with a Cu(OH)_2_ catalyst and 1,10-phenanthroline ligand in a green solvent (i.e., PEG-6000).^250^ A yield of 96% was obtained when ferulic acid was used while yields of between 31 and 73% were obtained for cinnamic acid, sinapic acid and *m*-coumaric acid. It was noted that decarboxylation of caffeic acid and *p*-coumaric acid were observed to be successful through observation of CO_2_ evolution, but yields were unable to be obtained due to the immediate polymerization of the monomers. From a sustainable chemistry perspective, it is noteworthy that the copper catalyst was shown to be effective for four runs in the decarboxylation of ferulic acid, with a yield of 96% obtained on the fourth run.^246,250^

In summary, various routes from bio-based sources are available to produce bio-styrene, including chemical and biological pathways. Compared to the oxidative dehydrogenation of ethylbenzene, these routes have the advantage that they require significantly lower temperatures and do not require the same expensive heterogeneous catalysts. While each step may proceed with high yields, these synthetic routes often require multiple steps and this limits both the overall yield and the sustainability. However, these functionalized bio-styrene monomers show comparable properties and performance to standard styrene.

## 3 Acrylics

### 3.1 (Meth)acrylates

Acrylic and methacrylic monomers are widely used throughout the polymer industry. Acrylic acid and its esters were listed as the 25^th^ top organic chemical product, and the market for acrylic acid grew from $11 billion in 2013 to nearly $19 billion in 2020.^173^ Similarly, methacrylic acid and alkyl methacrylates are important monomers to form polymers such as PMMA, which are used in many applications such as coatings, paints and electronics.^173^ The PMMA market was estimated at $7 billion in 2018 and is anticipated to grow by 17% to ca. $12 billion in 2022.^173^ Currently, acrylic acid is produced through the oxidation of propane which is obtained from crude oil. Alkyl acrylates are then produced by the esterification of the acrylic acid with their corresponding alcohol. For methyl methacrylate, the majority of industrial production is carried out through the acetone-cyanohydrin process.^173,251^ As well as having the problem of using petrochemical feedstocks, this process also produces large amounts of emissions of greenhouse gases, has poor selectivity and a poor atom economy.^252^ There have been a vast number of studies into the production of acrylic and methacrylic monomers from biomass. For instance, acrylic acid has been derived from glycerol, lactic acid, 3-hydroxypropanoic acid and acetic acid.^173^ The main focus of this section will be reviewing the different catalytic systems used to convert each of these bio-derived platform chemicals to acrylic acid. It is noteworthy, that acrylic acid and alkyl acrylates can be produced, as well as styrene, in ethenolysis (refer to section 2.4.2).^236^

For methacrylic acid, one potential approach is to replace petroleum-derived chemicals with their biomass-derived equivalents and use these in the current production methods. For example, for the acetone-cyanohydrin process, the fermentation of plants gives ethanol and acetone, which can be used in the process to form methacrylic acid.^252,253^ However, producing the feedstock from biomass does not overcome other disadvantages of this process, such as low selectivity, low atom economy and the formation of toxic intermediates, including acetone cyanohydrin.^252^ Hence, the main focus of this section is to explore the different catalytic systems which can convert bio-derived chemicals into methacrylic acid. The bio-sources discussed here are isobutane, produced through the thermo-catalytic process and fermentation of sugars, and bio-based carboxylic acids, such as itaconic and citric acids. Thus, bio-based approaches to form alkyl, cyclic and aromatic (meth)acrylates will be discussed.

For alkyl (meth)acrylates, the main focus herein is to highlight different production methods that involve glycerol, acrolein, acetic acid, alkyl lactates and vegetable oils/lipids. For the cyclic (meth)acrylates, terpenes, isosorbide, lactones and levoglucosenone are identified as key platform chemicals. Finally, aromatic (meth)acrylates will be evaluated where the majority of studies use lignin as the biomass. It is noteworthy that many of the thus far reported approaches are not fully bio-based as it involves coupling a bio-sourced platform chemical with a non-bio-derived reagent, such as methacrylic anhydride or (meth)acryloyl chloride. However, these approaches have been evaluated due to one of the major components of the reaction being sourced from biomass. Where more sustainable methods have been investigated, these have been discussed and their advantages and limitations have been highlighted.

#### 3.1.1 Acrylic Acid

**Figure 10:**
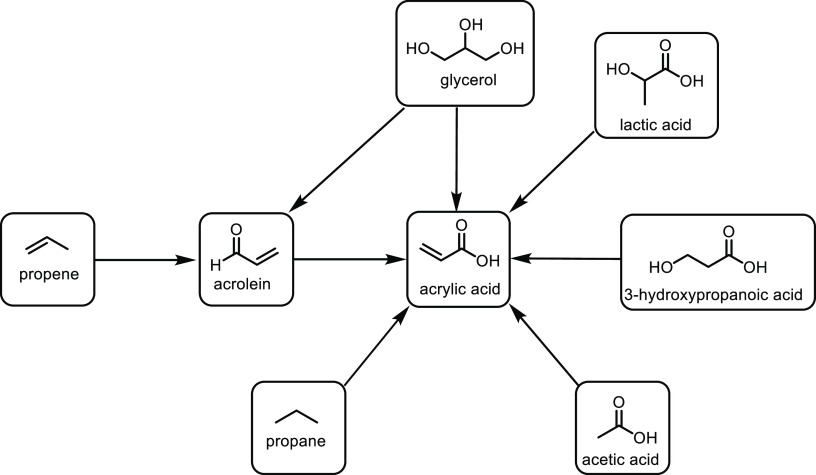
Synthetic routes to produce acrylic acid from various bio-sources including glycerol, lactic acid, 3-HPA and acetic acid.

Acrylic acid is one of the most important industrial chemicals and its alkyl esters are widely used in products such as paints, coatings and adhesives.^254−256^ Acrylic acid is produced from petrochemicals through the oxidation of propene.^254,256−258^ Nonetheless, acrylic acid can be produced sustainably from bio-sources including glycerol, lactic acid, 3-HPA and acetic acid, as illustrated in Figure 10.^173,259−262^

##### 3.1.1.1 From Glycerol

**Figure 11:**
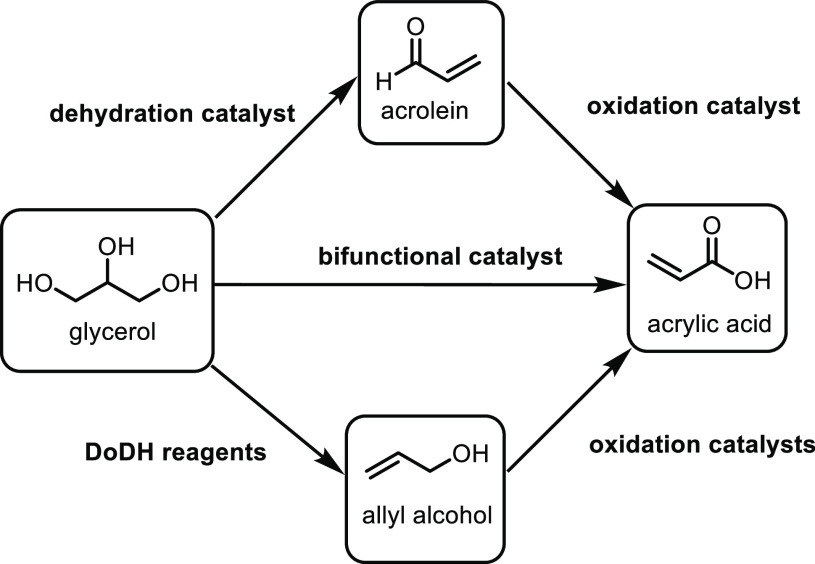
One-step and two-step routes to produce acrylic acid from glycerol.

The value of using glycerol has increased as it is a readily available bio-source due to its formation as a by-product in biodiesel production.^173,259,262^ Acrylic acid can be synthesized from glycerol by either a two- or one-step process (Figure 11).^173,263^ In one reported two-step synthesis, a double dehydration forms the intermediate acrolein, which then undergoes an oxidation to form acrylic acid. Another two-step pathway involved the dehydration of glycerol to allyl alcohol before an oxidation to acrylic acid. Alternatively, a single-step route can be applied, involving the dehydration to acrolein and oxidation of acrylic acid over a multifunctional catalyst.

There have been studies into many different catalysts for the production of acrylic acid from glycerol, including Keggin-type catalysts, metal mixed oxide-based catalysts and zeolite-based catalysts.^173,259^ Catalysts containing vanadium have also been thoroughly studied as multifunctional catalysts for the one-step synthesis of acrylic acid from glycerol, with examples summarized in Table 5. The multifunctional catalyst allows for the dehydration of glycerol to acrolein and the subsequent oxidation into acrylic acid to proceed in one step. The vanadium presence in the catalysts enables the oxidation of acrolein into acrylic acid (Table 5, entry 1), while doping it with other metals (Table 5, entries 2–6) enables acrylic acid formation below 310 °C.^263−268^ These catalysts could reach a conversion of >99%; however, acrylic acid selectivity is an issue, and this limits the yields (cf. Table 5). While the doped catalysts were able to function at lower temperatures, the highest yield of 57% was for the undoped vanadium Keggin-type catalyst, which operated at a temperature of 340 °C.^263^ Doping with niobium proved to be more effective than doping with iron, molybdenum and tungsten, with a yield of 51% being achieved.^268^ For the doped catalysts, increasing the temperature resulted in a decrease in selectivity, as the formation of heavy products was favored.^265^

**Table 5:** Different Catalytic Systems for the Formation of Acrylic Acid from Glycerol

Entry	Catalyst	Temperature (°C)	Reaction time (h)	Conversion (%)	Selectivity (%)	Yield (%)	Ref.
**1**	Cs(VO)_0.2_(PMo)_0.5_(PW)_0.5_	340	1	100	57	57	(263)
**2**	W-V-Nb-O	290	nr	100	34	34	(265)
**3**	VWO	<310	2	>99	26	26	(264)
**4**	MoVW-5	250	14	100	31	31	(266)
**5**	WMoV-3	290	70	100	40	40	(267)
**6**	NbVWO	265	37	100	51	51	(268)
**7**	PO_4_/W_2.2_V_0.4_Nb_2.4_O_14_	285	nr	100	59	59	(269)
**8**	CuNC/SiO_2_-MnO_2_	70	30	77	75	58	(270)
**9**	HZSM-5/MoVO	250	2	100	47	47	(266)
**10**	V6-SiW/HZSM-5	90	4	100	36	36	(271)
**11**	H,Fe-MCM-22	320	10	93	57	53	(272)

The introduction of phosphoric acid into catalysts increased the acidity and improved the oxidation, while hindering the oxidation of acrylic acid to form CO_*x*_ side products.^269^ When doped with phosphoric acid, an acrylic acid selectivity of 59% was obtained, which was notably higher than for the metal doped catalysts (Table 5, entry 7).^269^ A different type of catalyst consisting of copper nanocrystals supported on a SiO_2_–MnO_2_ support has also been developed.^270^ This catalyst was the most selective for acrylic acid, with an optimum selectivity of 75%, but had a significantly lower glycerol conversion of only 77% (Table 5, entry 8). However, even with low conversion, an acrylic acid yield of 58% was comparable to the phosphoric acid doped catalyst and was significantly higher than most of the metal doped mixed metal oxide catalysts explored. Another advantage of using this catalyst was that a significantly lower temperature of 70 °C could be used. The reusability of the catalyst was also explored and after 4 cycles the glycerol conversion and acrylic acid selectivity had only fallen slightly to 73% and 72%, respectively.

Mixed metal oxides have been combined with zeolites to form catalysts for the oxidative dehydration of glycerol (Table 5, entries 9–11).^266,271,272^ When the catalyst HZSM-5/MoVO was used, an acrylic acid selectivity of 47% was observed at full conversion.^266^ In this case, a two-bed system was utilized by having a layer of an acid catalyst (i.e., HZSM-5) on a layer of MoVW-5. This system overcame the problem of glycerol oxidation by ensuring that all the glycerol had been converted on the first bed, before the subsequent oxidation of acrolein on the second bed takes place. A vanadium doped SiW/HZSM-5 catalyst achieved a lower acrylic acid yield of 36%.^271^ However, an advantage of this catalyst was that it required a significantly lower temperature than the other mixed metal oxide/zeolite hybrid catalysts. It was noted that the textural properties of the catalysts are equally as important as the surface properties when considering the catalytic activity. A bi-functional H,Fe-MCM-22 catalytic system has also been developed with an optimum glycerol conversion and acrylic acid selectivity of 93% and 57% being obtained, respectively.^272^ This catalyst had a much higher acrylic acid selectivity than any previously reported zeolite-based catalysts for oxidative dehydration.^272,273^ Iron was selected as the metal for this catalyst due to its Fe^2+^/Fe^3+^ redox couple as well as due to iron being a more readily available, cheaper and less toxic metal than vanadium, tungsten or molybdenum, hence improving the sustainability aspect of this catalyst.

A different method as to attempt to improve acrylic acid yields involves using two separate catalytic systems for the two steps of the transformation, rather than using a bifunctional catalyst. In this case, the glycerol to acrolein and acrolein to acrylic acid reactions take place in different reactors with various catalysts under different optimized conditions.^259^ Several catalysts for the first step have been reported to give good glycerol conversions and acrolein selectivity including cesium doped silicotungstic acid/Al_2_O_3_ (90% acrolein selectivity, 100% conversion), HY zeolite (>99% acrolein selectivity, 89% conversion) and FePO_4_ (92% acrolein selectivity, 100% conversion).^259,274−276^ The second step of the reaction is the oxidation of acrolein, which is typically performed by molybdenum vanadium oxide catalysts. A good example is a vanadium molybdenum mixed oxide supported on silicon carbide synthesized by Liu and co-workers, with acrylic acid yields of up to 96% being obtained.^277^ An alternative approach is to use two separate catalysts on a double bed system where there are two separate fixed layers of the catalyst in one reactor. However, the optimum acrylic acid yields for this approach have only been reported to reach 75%.^259,278^

It is noteworthy that acrylic acid yields are higher for these two-step approaches compared to when a multifunctional catalyst is used.^269,270,274−276^ However, these approaches have disadvantages such as the need to operate two different reactors and to have to use two valuable catalysts rather than one. Furthermore, the catalytic performance and acrylic acid yields are heavily impacted by the purity of the glycerol.^173,259^ This is one of the major challenges in using crude glycerol from the production of biodiesel, as the necessary purifications are an expensive and time-consuming process. These processes are less appealing to commercialize if the large amounts of glycerol produced as a by-product cannot be used.

##### 3.1.1.2 From Lactic Acid

Lactic acid is the most common naturally occurring carboxylic acid and is produced *via* the fermentation of sugars, such as glucose and sucrose.^173^ In 2020, the global production was reported to be around 370 000 tons per year, with this value being predicted to grow substantially over the next decade.^173,259^ The dehydration of lactic acid to produce acrylic acid is challenging because there are several competing reactions such as decarbonylation, decarboxylation, and hydrogenation.^173,254,256,257^ If lactic acid is reacted with a Lewis acid or base, acrylic acid will be formed as the major product. However, if lactic acid is reacted in the presence of a Bronsted acid or base, acetaldehyde will be formed as a major side product, while it is also possible in basic conditions for 2,3-pentadione to be formed.^279^ Biochemical approaches have been explored in which acrylic acid has been formed in a reaction catalyzed by CoA transferase and lactyl-CoA-dehyratase, which were obtained from *Clostridium propionicum*.^254,256,257^ However, this anaerobic process gives a low yield of acrylic acid. Many catalysts have been studied for the production of acrylic acid from lactic acid, with zeolites having been the most widely used due to their high surface area.^173^ NaY, β and ZSM-5 zeolites have been the most vastly studied classes of zeolite-based catalysts for the transformation of lactic acid into acrylic acid (Table 6).^173^ Of these, NaY zeolites have been the most explored catalysts for the dehydration of lactic acid, especially when modified with potassium, barium or rare earth metals (including lanthanum, cerium, europium and samarium).^256,280−284^

**Table 6:** Different Zeolite-Based Catalysts for the Formation Acrylic Acid from Lactic Acid

Entry	Catalyst	Temperature (°C)	Conversion (%)	Selectivity (%)	Yield (%)	Ref.
**1**	NaY	350	100	35	35	(282)
**2**	La-NaY	350	100	56	56	(282)
**3**	Na_2_HPO_4_/NaY	350	78	72	56	(285)
**4**	2.8K/NaY	325	99	50	50	(281)
**5**	KI/NaY	325	98	68	67	(280)
**6**	KOH-Ca-NaY	350	100	84	84	(286)
**7**	K_0.94_Na_0.06_β	360	94	65	61	(287)
**8**	K/ZSM-5	365	99	77	76	(288)
**9**	0.5P/ZSM-505AT	350	97	78	76	(289)
**10**	NaH_2_PO_4_/ZSM-5	375	100	84	84	(290, 291)
**11**	K/ZSM-5/NaY	375	100	71	71	(292)
**12**	KH_2_PO_4_/NaZSM-5	330	98	86	84	(293)

The effect of the Si/Al ratio in NaY zeolites has also been investigated, with higher Si to Al favoring the formation of acetaldehyde, thus decreasing the selectivity toward acrylic acid.^279^ Attempts to modify NaY catalysts include doping with rare earth metals (Table 6, entries 1 and 2).^282^ Doping with 2 wt % lanthanum, cerium and europium increased the acrylic acid selectivity from 35% to 56%, 46% and 40%, respectively.^282^ Modifying NaY zeolites with alkali phosphates also had a positive impact on the reaction by decreasing surface acidity, thus reducing side reactions. The acrylic acid selectivity further increased up to 72% for the 14 wt % Na_2_HPO_4_/NaY catalyst at 350 °C (Table 6, entry 3).^285^ Doping with potassium led to an increase in both conversion and acrylic acid selectivity from 96% and 15% to 99% and 50%, respectively for the catalyst 2.8K/NaY after a 360 min reaction time at 325 °C (Table 6, entry 4).^281^ In a different study, the NaY zeolites were doped with potassium salts which led to an even greater increase in acrylic acid selectivity.^280^ The optimum value for acrylic acid selectivity that was achieved to be 68% when a KI/NaY catalyst was used while the lactic acid conversion was 98% (Table 6, entry 5). As for the alkali phosphates, potassium doping decreased the acidity of the catalysts, hence reducing unwanted side reactions.

Zhang *et al*. achieved even higher acrylic acid selectivity by doping NaY zeolites with alkali and alkaline earth metals.^286^ An optimum acrylic acid selectivity of 84% was obtained for the KOH-Ca-NaY catalyst (Table 6, entry 6). This was a significantly larger value than those of the NaY, KOH-NaY or Ca-NaY catalysts, which had selectivity values of 43%, 63% and 70%, respectively. The stability of the catalyst was further demonstrated, as after 4 long reaction cycles, an acrylic acid selectivity of 82% was obtained. While modified NaY zeolites show promising increases in the reaction selectivity, the problem of catalyst deactivation remains a challenge. Näfe *et al*. reported that the buildup of acidic hydrocarbons, such as lactic acid and acrylic acid commonly leads to catalyst deactivation. It was reported that substituting the Na^+^ ions for K^+^ or Cs^+^ can reduce such deactivation due to steric hindrance.^294^

Other types of zeolites have been explored for catalysis in the formation of acrylic acid including β-zeolites and ZSM zeolites.^173^ Alkali metal doped β-zeolites reached acrylic acid selectivity and conversion values of up to 65% and 94%, respectively (Table 6, entry 7).^287^ A general trend was observed where potassium and cesium doped zeolites had higher acrylic acid selectivity when compared to lithium and sodium doped zeolites. Yuan *et al*. studied alkali metal doped ZSM-5 zeolites, whereby the potassium doped catalyst gave an optimum acrylic acid selectivity of 77% at 99% conversion (Table 6, entry 8).^288^ All of the alkali metal doped catalysts gave significantly higher acrylic acid selectivity values than the HZSM-5 catalyst which, while having a high lactic acid conversion of 99.9%, had a selectivity of only 1.4%. Furthermore, ZSM-5 doped with NaOH and Na_2_HPO_4_ achieved similar results with an optimized conversion and acrylic acid selectivity of 97% and 78%, respectively for a 9 h reaction at 350 °C (Table 6, entry 9).^289^ When the ZSM-5 catalyst was treated with NaOH and doped with 7% NaH_2_PO_4_ an improved selectivity of 84% at full conversion was obtained (Table 6, entry 10).^290^ The improvement in performance was attributed to the phosphate doping changing the number of acid sites and increasing the basicity. The use of mixed zeolite catalysts has also been investigated. A K/ZSM-5/NaY catalyst achieved a 71% acrylic acid selectivity at full conversion in a reaction run at 375 °C (Table 6, entry 11).^292^ A final example of a zeolite catalyst investigated a NaZSM-5 catalyst, which managed to achieve a high acrylic acid selectivity of 86% with a 98% lactic acid conversion when doped with 4 mmol KH_2_PO_4_ (Table 6, entry 12).^293^

Phosphate catalysts (e.g., calcium hydroxyapatite, HAP) have also been used in the production of acrylic acid from lactic acid.^259^ The most important property in determining catalytic performance is the balance of acidic and basic sites. Strongly acidic sites favored unwanted side reactions and so moderate or weakly acidic sites were important for optimal catalytic performance.^259^ HAP catalysts have both weakly acidic and weakly basic sites which favored dehydration and the acid/base properties can be tuned by altering the Ca/P ratio.^259,295^ For the catalyst HAP-3, which has a Ca/P ratio of 1.3, an optimized acrylic acid selectivity of 60% was obtained for a reaction at 375 °C (Table 7, entry 1).^295^ Yan *et al*. found that increasing the Ca/P ratio led to an increase in the acrylic acid selectivity, with a catalyst with a Ca/P ratio of 1.62 achieving an optimum acrylic acid selectivity of 74% (Table 7, entry 2).^296^ However, a lower lactic acid conversion of 84% was observed. The correlation between the Ca/P ratio and acrylic acid selectivity was attributed to the ratio of acid/base sites. Matsuura *et al*. optimized conditions for the use of a Ca-HAP(1.55)_NaOH_ catalyst and achieved an optimum acrylic acid yield of 78% and a lactic acid conversion of 90% (Table 7, entry 3).^297^ This catalyst had a Ca/P ratio of 1.55 and was doped with 1.4 wt % of sodium. The Na^+^ ions played an important role in replacing deficient Ca^2+^ ions and this led to increased catalytic performance.

**Table 7:** Different Phosphate-Based Catalysts for the Formation Acrylic Acid from Lactic Acid

Entry	Catalyst	Temperature (°C)	Conversion (%)	Selectivity (%)	Yield (%)	Ref.
**1**	HAP-3	375	100	60	60	(295)
**2**	HAP_1.62_-360	360	84	74	62	(296)
**3**	Ca-HAP_(1.55)_NaOH	350	90	87	78	(297)
**4**	CP-3	375	100	78	78	(298)
**5**	K_2_HPO_4_/CaOP	nr	91	93	85	(173)
**6**	K_2_HPO_4_/BaOP	350	91	93	85	(299, 300)
**7**	K_2_HPO_4_/SrOP	380	100	72	72	(301)
**8**	BaPP	400	100	76	76	(302)
**9**	CaSO_4_	400	>99	69	69	(303)
**10**	BaSO_4_	400	>99	66	66	(303)

As well as HAPs, alkaline earth metal pyrophosphates (MPP) and orthophosphates (MOP) have been reported as catalysts in the production of acrylic acid from lactic acid.^173^ Ghantani and co-workers showed an optimum acrylic acid selectivity of 78% for a reaction at 375 °C with the catalyst CP-3 (an MPP), which had a low Ca/P ratio of 0.76 (Table 7, entry 4).^298^ The increased selectivity was attributed to the acid/base balance at low Ca/P ratios which resulted in the increased formation of a calcium lactate intermediate. MPPs and MOPs can be doped to improve their catalytic activity. For example, calcium orthophosphate has been doped with K_2_HPO_4_ which resulted in a very high acrylic acid selectivity of 93% along with a lactic acid conversion of 91% (Table 7, entry 5).^173^ Metals other than calcium can be used in MPPs and MOPs to obtain acrylic acid in comparable yields. In one example, a K_2_HPO_4_ doped BaOP catalyst was used in a one-pot conversion of lactic acid to acrylic acid, with a conversion and selectivity of 91% and 93%, respectively (Table 7, entry 6).^299,300^ Tang and co-workers obtained an optimal acrylic acid selectivity of 72% when a SrPP catalyst doped with 0.1 wt % H_3_PO_4_ was used at 380 °C (Table 7, entry 7).^210,301^ It was also shown that BaPP achieved an acrylic acid selectivity of 76% at 400 °C (Table 7, entry 8).^302^

Other phosphate catalysts that have been explored include lanthanum and cerium phosphates. However, acrylic acid selectivity values were relatively low at 50% and 64%, respectively.^173,304,305^ As well as phosphates, sulfate salts and doped sulfate salts have been explored as catalysts. Peng and co-workers investigated the use of metal sulfates as catalysts for the production of acrylic acid from lactic acid.^303^ Magnesium, barium and calcium sulfates had higher catalytic performances than aluminum, nickel or zinc sulfates. This was attributed to the calcium, magnesium and barium salts being weaker acids, while the other sulfates tested were more acidic and so favored unwanted side reactions. Calcium sulfate achieved slightly better results than barium sulfate (i.e., 69% vs 66% selectivity) but the barium salt was found to be the most stable catalyst (Table 7, entries 9 and 10).

##### 3.1.1.3 From 3-Hydroxypropanoic Acid

A structural isomer of lactic acid, 3-hydroxypropionic acid (3-HPA), is also produced through glucose fermentation or from glycerol.^257^ Furthermore, acrylic acid can be prepared *via* the dehydration of 3-HPA. Different classes of catalysts have been used in the dehydration of 3-HPA to acrylic acid, including Bronsted acids, metal oxides or zeolite catalysts.^173^ The thermal dehydration of 3-HPA produced an 80% yield of acrylic acid, using reduced pressure, a sulfuric acid (or phosphoric acid) catalyst and copper powder as an inhibitor, at elevated temperatures between 140 and 160 °C.^306^ If an alcohol is added into this reaction, the alkyl acrylate can be formed directly.^307^ Heterogeneous catalysts have been reported to increase the yield of production, for example, a yield of 96% was achieved when a NaH_2_PO_4_ on silica catalyst was used.^308^ Gas phase dehydration reactions have also been reported using metal oxide or zeolite catalysts, with conversions of 97% being achieved when Al_2_O_3_ was used as the catalyst.^309^ An example of a metal oxide catalyst was the use of Cu-Ba-CrO as a catalyst.^259,310^ Full acrylic acid selectivity was reported when used in a batch reactor, but a lower 3-HPA conversion of 63% was observed. Alumina and silica catalysts resulted in a 97% acrylic acid yield in a reaction at 250 °C.^311^ Other studies showed that TiO_2_ could also be an effective catalyst for this reaction, with a selectivity for acrylic acid of 99%.^312,313^ Different solid acid catalysts such as, HY, ZSM-5, β, MCM-41 and silica gel were explored by Li *et al*.^314^ The silica gel proved to be the best catalyst for this reaction, with an optimal acrylic acid yield of >99%. There was also very limited deactivation over 200 h, with excellent catalytic stability being shown.

The performance of the reaction is heavily influenced by the purity of the 3-HPA starting material.^315^ When pure synthesized 3-HPA was reacted over a bentonite catalyst, a high acrylic acid yield (i.e., 89%) was observed. However, when a less pure bio-3-HPA was used under the same conditions, a much lower acrylic acid yield of 68% was reached. When bio-3-HPA was purified with a cation exchange resin before the reaction, 3-HPA conversion and acrylic acid selectivity were 95% and 98%, respectively. While high catalytic performance was achieved, it should be noted that the purification has significant disadvantages including added cost and increased time consumption.^316^ While there have been promising results in terms of acrylic acid yields from 3-HPA, another problem is that the formation of the starting material *via* fermentation is challenging and has deemed the route unviable to industrially produce acrylic acid from 3-HPA.^259^

##### 3.1.1.4 From Allyl Alcohol

Acrylic acid can also be formed from allyl alcohol, which is an intermediate in the production of acrylic acid from glycerol.^173^ Molybdenum vanadium mixed oxides can catalyze this reaction, with the heptagonal channels present in the catalyst structure providing active sites for the oxidation of both allyl alcohol and acrolein to acrylic acid.^317^ The maximum acrylic acid yield was 73% when an orthorhombic MoVO_*x*_ catalyst was used at 350 °C. However, the main problem with using molybdenum vanadium mixed oxide catalysts is that they are unstable and deactivate over time. As for the oxidative dehydration of glycerol to form acrylic acid, the addition of a third metal, such as tungsten, copper and iron, has been shown to improve the catalytic activity and stability.^173,263^ The effects of doping MoVO_*x*_ with copper, iron and tungsten on the catalytic performance were investigated.^318^ The best catalyst for the oxidation of allyl alcohol was shown to be the iron doped catalyst, MoVFeO, which achieved an acrylic acid yield of 83% at 350 °C. The high activity of this catalyst was attributed to the prevention of the isomerization of the allyl alcohol, which would result in undesired side reactions.

An alternative to the mixed metal oxide catalysts is liquid oxidation using noble metal nanoparticles supported on metal oxides.^319^ Gold nanoparticles supported on CeO_2_ showed the best acrylic acid selectivity of 51% at full conversion. TiO_2_, ZnO, Fe_2_O_3_ and carbon-based supports all achieved significantly lower acrylic acid yields, thus highlighting the importance of the support on the catalytic activity. One advantage of this procedure was the significantly lower operating temperature of 50 °C, compared to the temperatures >300 °C that were required for the metal mixed oxide and zeolite catalysts. Kim and co-workers improved on the acrylic acid selectivity of the Au/CeO_2_ catalyst by investigating the different morphologies of catalysts.^320^ The octahedral CeO_2_ support provided a higher catalytic activity compared to the cubic or rod-shaped geometries, with the optimum acrylic acid selectivity being 92% at full conversion. The octahedral geometry has the highest surface area, which led to a greater catalytic performance. Once again, this procedure had the advantage of a low operating temperature of 25 °C. One of the issues with the Au/CeO_2_ catalyst, however, is that it requires 3M NaOH. Different catalysts that operate in base free conditions have been investigated, which has two main advantages. First, the amount of waste is reduced and second corrosion in the reactor is limited.^321^ However, the catalytic performance was significantly lower with the highest acrylic acid yield being 43% when 4 wt % Pd NP/C(sol) catalyst was used in a 6 h reaction at 100 °C.

##### 3.1.1.5 From Acetic Acid

Acetic acid can be used to synthesize acrylic acid through an aldol reaction with formaldehyde. Both of these reactants can be produced sustainably through the fermentation of biomass.^322,323^ The main class of catalysts for this reaction are vanadium phosphorous oxides. Hu and co-workers achieved an optimum acrylic acid selectivity of 93% for a vanadium phosphate catalyst on a SBA-15 support, which had a P/V ratio of 2.4, at 330 °C.^324^ However, the conversions of acetic acid were very low. By increasing the reaction temperature to 370 °C the conversion increased to 57% but the acrylic acid selectivity fell to 78%. The catalytic performance of different catalysts was attributed to the affect the different P/V ratios had on the acid/base characteristics of the catalysts. By doping a vanadium phosphate catalyst with cerium, Wang and co-workers achieved an acrylic acid yield of 74% at 380 °C.^325^ The addition of metal cations increased the V^5+^/V^4+^ ratio which promoted a higher catalytic performance. Liu and co-workers investigated a VPO catalyst supported on a siliceous mesostructured cellular foam (MCF).^326^ The V^5+^/V^4+^ ratio of the catalyst was tunable by the VPO loading, while during the catalyst synthesis, ammonia was used to induce a partial reduction of V^5+^. Using this catalyst, the combined yield of acrylic acid and methyl acrylate was 84% at a reaction temperature of 360 °C.

#### 3.1.2 Methacrylic Acid

##### 3.1.2.1 From Isobutane

Isobutane can be produced from biomass through a thermo-catalytic process as well as through the fermentation of sugars.^327^ To produce MAA from isobutane, first the oxidation of isobutane to the intermediate methacrolein occurs, before further oxidation resulting in the formation of methacrylic acid.^173^ The most frequently used class of catalysts used for the oxidation of isobutane are Keggin-type catalysts, which are based on a H_4_Pmo_12_O_40_ structure. The structure consists of a phosphorus atom at the center of a tetrahedron that is neighbored by MoO_6_ units. The acidity/basicity of the catalyst can be tuned through the addition of different metals.^173^ For example, Mizuno and co-workers showed that an unmodified H_3_Pmo_12_O_40_ catalyst showed very low activity and selectivity, with a conversion and MAA selectivity of just 7% and 4%, respectively, being obtained (Table 8, entry 1).^328^ The catalyst selectivity was significantly improved by using a pyridinium 12-molybdophosphate (H_3_Pmo_12_O_40_-Py).^329^ An optimum MAA selectivity of 58% was obtained at 12% isobutane conversion at 300 °C (Table 8, entry 2). An increased temperature of 340 °C showed an increased conversion of 47% but with a significant drop in selectivity to 18%. The better performance of this catalyst was attributed to the reduced molybdophosphate and oxygen deficient anions providing suitable activating sites for both oxygen and isobutane.

**Table 8:** Different Keggin-Based Catalysts for the Transformation of Isobutane to Methacrylic Acid

Entry	Catalyst	Temperature (°C)	Conversion (%)	Selectivity (%)	Yield (%)	Ref.
**1**	H_3_PMo_12_O_40_	340	7	4	0.28	(328)
**2**	H_3_PMo_12_O_40_-Py	300	12	58	7	(329)
**3**	Cs_2.5_H_0.5_PMo_12_O_40_	340	16	24	4	(330)
**4**	Cs_2_HPMo_12_O_40_	340	11	34	4	(330)
**5**	Cs_2.5_Ni_0.08_H_0.34_PMo_12_O_40_	340	24	27	7	(330)
**6**	(NH_4_)_3_PMo_12_O_40_	352	5	42	2	(331)
**7**	H_4_PVMo_11_O_40_	350	11	30	3	(332)
**8**	Cs_0.75_H_0.25_VO[PMo_12_O_40_]	350	nr	76	nr	(333)
**9**	Cs_2_V_0.3_PMo_11_O_40_	330	6	55	3	(334)
**10**	Cs_1.7_(NH_4_)_1.3_HPMo_11_VO_40_	340	10	43	4	(335)
**11**	(NH_4_)_3_HPMo_11_VO_40_/Cs_3_PMo_12_O_40_	340	14	47	6	(336)
**12**	Cs_2_V_1.3_Cu_0.2_PMo_12_O_40_	350	13	48	6	(337)
**13**	Cs_2_HPMo_6_O_12_	350	9	44	4	(337)

The selectivity and activity of the catalysts can also be improved through doping with other metals, for example, cesium, vanadium, and copper.^330^ Keggin-type catalysts can be doped with cesium to improve catalytic activity. The catalyst Cs_2.5_H_0.5_PMo_12_O_40_ showed a MAA selectivity of 24% (at 16% conversion), while the catalyst with slightly less cesium, Cs_2_HPMo_12_O_40_, had an increased MAA selectivity of 34% but at lower conversion (i.e., 11%, Table 8, entries 3-4). Further modification by addition of a nickel dopant showed an increase in activity without too much change to the MAA selectivity, with a selectivity of 27% being obtained at 24% conversion (Table 8, entry 5).^330^ It was shown that doping the catalyst with nickel improved the catalyst performance more significantly than when Mn^2+^, Fe^3+^, Cu^2+^ or Co^2+^ were used.^338^ Replacing the protons with ammonium cations significantly increased the selectivity (42%) compared to the unmodified catalyst, while not improving the activity (5% conversion) under isobutane rich conditions (Table 8, entry 6).^331^ However, under isobutane deficient conditions, the selectivity fell back to a value comparable with the unmodified catalyst (4%). The difference in selectivity for the two different conditions was attributed to the redox reaction between ammonia and Mo^6+^ occurring at high isobutane concentrations, which positively impacted the catalytic performance.

Partially substituting molybdenum with vanadium led to an increase in selectivity of the catalyst.^332^ The vanadium occupies a cationic site and becomes an active site for the oxidation of isobutane. For the catalyst H_4_PVMo_11_O_40_, a conversion and MAA selectivity of 11% and 30% were obtained respectively at 350 °C (Table 8, entry 7). Further doping with cesium led to an improved selectivity of 76% (Table 8, entry 8).^333^ He and co-workers investigated various catalysts containing both cesium and vanadium, and obtained an optimum selectivity of 55% with a conversion of 6% at 330 °C using a Cs_2.0_V_0.3_PMo_11_VO_40_ catalyst (Table 8, entry 9).^334^ The key finding of this study was that the precursors and methods used to synthesize the catalysts were important for good catalytic performance. The optimal catalyst was obtained from vanadyl sulfate and showed good V^4+^/V^5+^ and Mo^5+^/Mo^6+^ surface ratios, along with high acidity leading to good performance.

Catalysts containing both cesium and ammonium ions have also been studied. The best results were achieved with the catalyst Cs_1.7_(NH_4_)_1.3_HPMo_11_VO_40_ at 340 °C, with a MAA selectivity of 43% reached at 10% conversion (Table 8, entry 10).^335^ The key factor in catalytic performance was thought to be the balance between acidity and specific surface area. The catalytic loading for a (NH_4_)_3_HPMo_11_VO_40_ catalyst was investigated and shown to have a significant impact on catalytic activity.^336^ While at 10 wt %, the reaction did not occur, increasing the catalyst loading increased the selectivity of the oxidation. At 50 wt %, the optimum results of a MAA selectivity of 47% at 14% isobutane conversion was obtained (Table 8, entry 11). Catalysts doped with Cu^2+^ and VO^2+^ have also been developed and achieved an optimal MAA selectivity of 48% at 13% isobutane conversion using a Cs_2_V_0.3_Cu_0.2_PMo_12_O_40_ catalyst at 350 °C (Table 8, entry 12).^337^ This was a slight improvement on the undoped Cs_2_HPMo_6_O_12_ catalyst, for which a MAA selectivity of 44% at 9% isobutane conversion was observed (Table 8, entry 13). The improvement in catalytic activity was attributed to the Cu^2+^ and VO^2+^ ions in the secondary structure assisting the re-oxidation of Mo^5+^ to Mo^6+^. Doping with other metals was attempted, including iron, nickel and cerium, but none resulted in superior results to the copper doped catalyst.

A small number of mixed oxide catalysts have also been used in the oxidation of isobutane to methacrylic acid.^339,340^ The catalyst with the highest performance was a MoV_0.3_Te_0.23_Ce_0.2_ catalyst, which achieved a combined methacrylic acid and methacrolein selectivity of 20% when an isobutane conversion of 20% was reached.^339^ This showed a significant improvement on the catalyst without the cerium (12% selectivity at 15% conversion), which was attributed to cerium’s high oxygen storage potential hence improving oxygen mobility throughout the catalyst. It was also noted that cerium can participate in a redox reaction with molybdenum and help in the reoxidation of the catalyst.

While there have been many studies into different catalysts to produce methacrylic acid from isobutane, it is plagued by extremely low yields. While doping catalysts led to increases in selectivity, all examples show extremely low isobutane conversions (i.e., <24%). It is also noteworthy that moderately high temperatures are required to achieve these poor yields, thereby impeding the sustainability of this process. Until these techniques have been developed further to boost methacrylic acid yields, the production of methacrylic acid from isobutane remains rather unfeasible on an industrial scale.

##### 3.1.2.2 From Carboxylic Acids

Besides isobutane, bio-based MAA can also be produced from bio-based carboxylic acids, such as itaconic acid or citric acid (which can be produced through the fermentation of carbohydrates), through a dehydration and decarboxylation reaction.^173^ Both homogeneous and heterogeneous catalysts have been investigated for use in the production of MAA from carboxylic acids (Table 9 and Table 10).

**Table 9:** Different Homogeneous Catalysts That Were Used for the Transformation of Various Biobased Carboxylic Acids to Methacrylic Acid

Entry	Catalyst	Substrate	Temperature (°C)	Conversion (%)	Selectivity (%)	Yield (%)	Ref.
**1**	No catalyst	Itaconic acid	400	92	33	30	(341)
**2**	No catalyst	Citric acid	320	100	7	7	(341)
**3**	10 mM NaOH	Itaconic acid	360	100	72	72	(341)
**4**	0.5 M NaOH	Mesaconic acid	320	100	52	52	(343)
**5**	0.5 M NaOH	Citraconic acid	320	98	40	39	(343)
**6**	Ru carbonyl proprionate	Itaconic acid	225	34	100	34	(344)

**Table 10:** Different Heterogeneous Catalysts That Were Used for the Transformation of Various Biobased Carboxylic Acids to Methacrylic Acid

Entry	Catalyst	Substrate	Temperature (°C)	Conversion (%)	Selectivity (%)	Yield (%)	Ref.
**1**	Pd/C/0.1 M NaOH	Itaconic acid	250	89	47	42	(345)
**2**	Pt/Al_2_O_3_	Itaconic acid	250	80	84	67	(345)
**3**	Pd/Al_2_O_3_	Citric acid	250	100	41	41	(345)
**4**	Pt/Al_2_O_3_	Aconitic acid	250	100	48	48	(345)
**5**	BaAl_12_O_19_	Itaconic acid	250	100	50	50	(346)
**6**	BaAl_12_O_19_	Citric acid	250	100	50	50	(346)
**7**	BaAl_12_O_19_	Aconitic acid	250	85	51	43	(346)
**8**	Hydrotalcite	2-HIBA	275	80	89	72	(347)
**9**	LaHA	Itaconic acid	250	100	12	12	(348)
**10**	CaHA	Itaconic acid	250	100	38	38	(348)
**11**	MgHA	Itaconic acid	250	100	40	40	(348)
**12**	BaHA	Itaconic acid	250	100	50	50	(348)

In general, homogenous catalysts results in increased yields when compared to heterogenous catalysts but have the disadvantage of being more difficult to be separated from reaction products. A specific example of using homogeneous catalysts to produce MAA has been reported by Carlsson and co-workers using citric acid and itaconic acid as substrates.^341^ For itaconic acid, when no catalyst was used at 400 °C, a yield of 30% was obtained for MAA at 92% conversion (Table 9, entry 1). The reaction was carried out under supercritical water conditions and they reported that the decarboxylation of acids was assisted by the dissociation of water into hydronium and hydroxide ions. For the reaction of citric acid with no catalyst present at 320 °C, the reaction went to full conversion but the yield of MAA was only 7% (Table 9, entry 2). It was also found that the reaction strongly depended on the pH with both neutral and acidic conditions promoting unwanted side products, such as acetic acid and acetone, at high temperatures.^341,342^ Conditions that were too basic were also detrimental to the reaction by quenching the decarboxylation process.^341,342^ However, the addition of a small amount of base was found to improve results for the reaction with itaconic acid. When 10 mM NaOH was added, full conversion was obtained at 360 °C, with a 72% yield of MAA (Table 9, entry 3). In contrast, the addition of 10 mM NaOH to the citric acid reaction showed no improvement on the selectivity of MAA production.

Other bio-based carboxylic acids have been studied to produce MAA, and these include mesaconic acid and citraconic acid.^343^ When reacted in 0.5 M NaOH at 320 °C, the yields of MAA were 52% and 40% for mesaconic and citraconic acid, respectively (Table 9, entries 4 and 5). More recently, a ruthenium carbonyl propionate catalyst was used to further improve the selectivity for MAA in the reaction of itaconic acid.^344^ This system greatly improved the selectivity for MAA, with 100% selectivity being observed for a 1.5 h reaction at 225 °C, containing 0.1 wt % of catalyst and 0.5 mol % of PPh_3_. This resulted in an overall yield of MAA of 34% (Table 9, entry 6).

In contrast to homogenous catalysts, using heterogeneous catalysts enables the use of more expensive catalysts as they can often be recovered and recycled. Hence, the use of various platinum and palladium catalysts for the transformations of carboxylic acids to MAA has been investigated.^345^ When itaconic acid was used with a Pd/C catalyst and 0.1 M NaOH at 250 °C, a MAA selectivity of 47% was obtained at 89% conversion (Table 10, entry 1). By using a Pt/Al_2_O_3_ catalyst for the reaction of itaconic acid, the selectivity was significantly improved to 84% (at 80% conversion, Table 10, entry 8). Al_2_O_3_ supported Pd and Pt catalysts were also used to convert citric acid and aconitic acid, respectively. However, these resulted in lower methacrylic acid yields of 41% and 48% compared to the reaction of itaconic acid with Pt/Al_2_O_3_ (Table 10, entries 3 and 4). BaAl_12_O_19_ catalyst was also investigated as a catalyst for the conversion of itaconic acid, citric acid and aconitic acid into MAA (Table 10, entries 5–7).^346^ Itaconic acid and citric acid both reached full conversion with a 50% selectivity for MAA, while the reaction of aconitic acid obtained a 51% MAA selectivity at 85% conversion. Hydrotalcite was used as a catalyst in the production of MAA from itaconic acid, citric acid and 2-hydroxyisobutyric acid (2-HIBA).^347,348^ While a MAA yield of 72% was achieved at 80% conversion for the reaction of 2-HIBA (Table 10, entry 8), lower yields of 23% and 21% were recorded for itaconic and citric acid, respectively. Finally, various hexaluminate catalysts have been used for the conversion of itaconic acid into methacrylic acid (Table 10, entries 9-12).^348^ Barium hexaluminate showed the highest catalytic activity, achieving a 50% MAA selectivity at full conversion.

#### 3.1.3 Alkyl (Meth)acrylates

##### 3.1.3.1 From Acrylic Acid

Industrially, alkyl acrylates, such as methyl acrylate, are produced *via* an esterification reaction with acrylic acid and an excess of alcohol in the presence of a strong acid (e.g. sulfuric acid).^259^ To obtain high yields, an excess of alcohol is typically required, but this leads to problems with purification of the end product. Another challenge for the esterification of acrylic acid is the production of unwanted side products, such as dimers and trimers of acrylic acid formed *via* Michael addition and alkyl alkoxy esters.^259^

**Figure 12:**
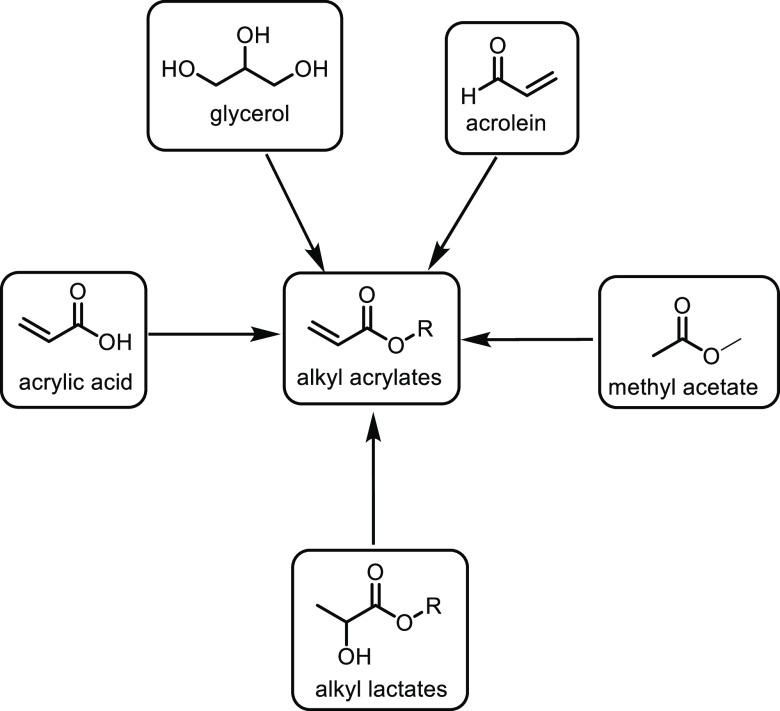
Various routes to produce alkyl acrylates from bio-derived feedstocks.

Alternative methods have been sought which reduce the need to use an alcohol in a large excess. One such method uses dimethyl carbonate instead of an alcohol. A high yield of 91% methyl acrylate was achieved when aluminum triflate was used as a catalyst.^259,349,350^ A different ZrOCl_2_·8H_2_O catalyst was used for the esterification of acrylic acid and methanol, producing methyl acrylate in a 71% yield.^351^ This catalyst offered the significant advantage over strong acid catalysts (e.g. sulfuric acid) in that it allowed acrylic acid and methanol to be used in an equimolar ratio, thus overcoming problems with purification of the product.^351^ While improved methods for the esterification of acrylic acid have been made, possible alternative routes from feedstock directly, without the need for an esterification of acrylic acid, have been highly sought after, with some of these being displayed in Figure 12. Examples of feedstock for the synthesis of alkyl acrylates include 3-HPA, glycerol, acetic acid and lactic acid.

##### 3.1.3.2 From Alkyl Lactates

The hydroxyl and carboxylic acid functional groups in lactic acid make it a useful precursor in the production of different chemicals such as acrylic acid, lactate esters and 1,2-propanediol.^256^ While the use of lactic acid in the synthesis of bio-acrylic acid has already been discussed (section 3.1.1.2), lactate esters can also be used in the synthesis of acrylate and methacrylate monomers. Lactate esters can be used to produce alkyl acrylates through two methods. First, the lactate esters can undergo a dehydration reaction, similar to the transformation of lactic acid into acrylic acid. Second, the hydroxyl group of the lactate ester can react with a coupling agent such as acryloyl chloride to form more complex acrylates.

Although lactic acid can be made through chemical synthesis, it is commercially produced from the fermentation of glucose.^257^ Whereas conventional production uses a large amount of sulfuric acid and produces large amounts of calcium sulfate, new technologies have been developed in membrane separation and purification methods to try and increase the sustainability of production.^257,352,353^ Alkyl lactates are typically synthesized from lactic acid *via* esterification. While the conversion of lactic acid to acrylic acid has been vastly studied, the conversion of alkyl lactates has been less explored. The same classes of catalysts that are used for the synthesis of acrylic acid from lactic acid, can be used to produce alkyl acrylates. For pure HAP catalysts, there is a significant decrease in conversion (99% vs 55%) and selectivity (68% vs 18%) when the feedstock was changed from lactic acid to ethyl lactate (Table 11, entry 1).^354,355^ Hong and co-workers observed a decrease in lactate conversion as the alkyl chain increased in length with the lactate conversions of methyl, ethyl and butyl lactates having conversions of 91%, 82% and 76% when using a mixed HAP/Na-CaPP (50/50) catalyst.^356^ However, both the alkyl acrylate and acrylic acid selectivity values remained similar at about 5% and 75% respectively (Table 11, entries 2–4). Hong also investigated CaOP-CaPP(50/50) composite catalysts at 390 °C for methyl lactate and ethyl lactate.^357^ Once again the methyl lactate proceeded with higher conversion (91%) compared to ethyl lactate (57%), but the difference was more pronounced with this catalyst. The same problems with selectivity were observed for both of the alkyl acrylates (only 5% selectivity) while the values for acrylic acid selectivity remained high at 75% and 79% for the reactions with methyl lactate and ethyl lactate, respectively (Table 11, entries 5 and 6). A CaOP/CaSO_4_ catalyst was shown to not have the same issues in terms of selectivity.^358,359^ For a reaction with methyl lactate at 400 °C, a MA selectivity of 53% was achieved, while no acrylic acid selectivity was reported. However, this reaction did proceed with a significantly lower methyl lactate conversion of 50% (Table 11, entry 7). Zhang *et al*. explored the use of CaSO_4_ catalysts with different promoters.^255^ The catalyst showing the highest performance was a CaSO_4_/CuSO_4_·5H_2_O/Na_2_HPO_4_/KH_2_PO_4_ system with a ratio of 150:13.78:2.5:1.2. In a reaction with methyl lactate, a conversion of 76% was achieved while selectivity values for acrylic acid and methyl acrylate of 54% and 26%, respectively were obtained (Table 11, entry 8).

**Table 11:** Different Catalysts Used to Convert Alkyl Lactates to Alkyl Acrylates

Entry	Catalyst	Substrate	Temperature (°C)	Conversion (%)	Selectivity (%)	Yield (%)	Ref.
**1**	HAP	Ethyl lactate	nr	55	18	10	(354, 355)
**2**	HAP/Na-CaPP (50:50)	Methyl lactate	390	91	5	5	(356)
**3**	HAP/Na-CaPP (50:50)	Ethyl lactate	390	82	5	4	(356)
**4**	HAP/Na-CaPP (50:50)	Butyl lactate	390	76	5	4	(356)
**5**	CaOP/CaPP (50:50)	Methyl lactate	390	91	5	5	(357)
**6**	CaOP/CaPP (50:50)	Ethyl lactate	390	57	5	3	(357)
**7**	CaOP/CaSO_4_	Methyl lactate	400	50	53	27	(358, 359)
**8**	CaSO_4_/CuSO_4_.5H_2_O/Na_2_HPO_4_/KH_2_PO_4_	Methyl lactate	400	76	26	20	(255)
**9**	NaX	Methyl lactate	240	99	93	92	(360, 361)
**10**	Cs-NaX	Methyl lactate	300	70	43	30	(362)
**11**	La-Montmorillonite/NaY	Methyl lactate	320	88	59	52	(363)

Zeolites have also been used to catalyze the dehydration of alkyl lactates. The best catalytic performance reported was obtained with a NaX catalyst at 240 °C when a methyl lactate conversion of 99% was achieved along with selectivity values for methyl acrylate and acrylic acid of 93% and 2%, respectively (Table 11, entry 9).^360,361^ This shows more promising results in terms of methyl acrylate selectivity than any of the Ca-based catalysts. However, since patented, these promising results have yet to be replicated by others.^259^ Other zeolites that result in a higher methyl acrylate selectivity compared to acrylic acid include Cs-NaX and La-montmorillonite/NaY (Table 11, entries 10 and 11).^362,363^

**Figure 13:**
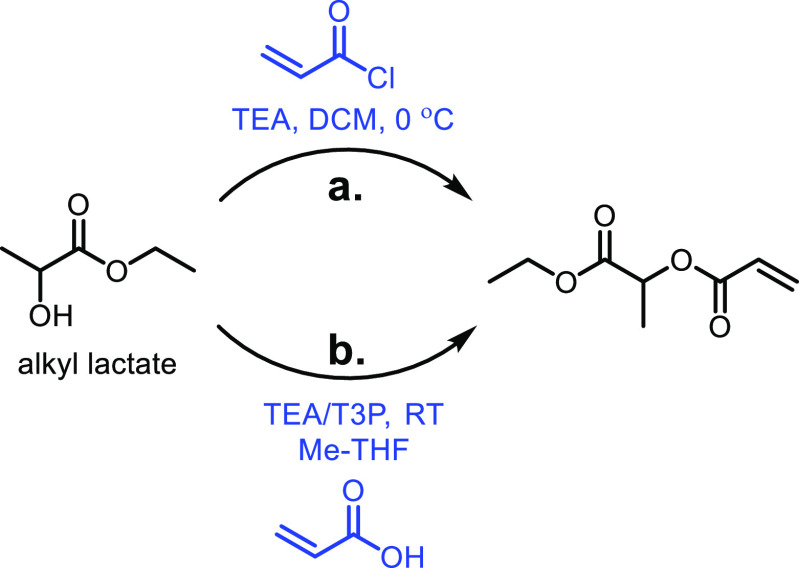
Two synthetic routes to synthesize acrylate monomers from alkyl lactates using acryloyl chloride (a) and a more sustainable route using acrylic acid (b).

An alternative method to form acrylates from lactate esters involves the reaction of the hydroxyl group with a coupling agent. Bensabeh *et al*. synthesized ethyl lactate acrylate *via* two different approaches, while also synthesizing methyl lactate acrylate and butyl lactate acrylate through the first approach.^364^ The starting material, ethyl lactate, can be synthesized from lactic acid through a reaction with ethanol catalyzed by Cal-B in dioxane with 85% yield.^365,366^ The two approaches for the synthesis of ethyl lactate acrylate are displayed in Figure 13. Methyl lactate acrylate, ethyl lactate acrylate and butyl lactate acrylate were synthesized by reacting the alkyl lactates with acryloyl chloride in the presence of triethylamine at 0 °C. A second synthetic route was attempted for ethyl lactate acrylate in which ethyl lactate was reacted with acrylic acid instead of acryloyl chloride in the presence of triethylamine and the coupling agent, propylphosphonic anhydride (T3P); however, this produced a lower yield of 60% compared to 70% for the reaction with acryloyl chloride.^364,367^ This second route was designed to be more sustainable by using bio-acrylic acid while T3P has reduced toxicity compared to other coupling agents. Interestingly, the thus obtained monomer was also investigated in terms of its polymerization potential. Living radical polymerization of ethyl lactate acrylate was optimized using ethanol as a green solvent with polymers ranging from DP 25 to 400 being achieved with >95% conversion and low dispersities (i.e., <1.23).^364^ Ethyl lactate acrylate was also co-polymerized with α-pinene acrylate and solketal acrylate to form soft-hard and amphiphilic (after deprotection) block copolymers, respectively.

**Figure 14:**
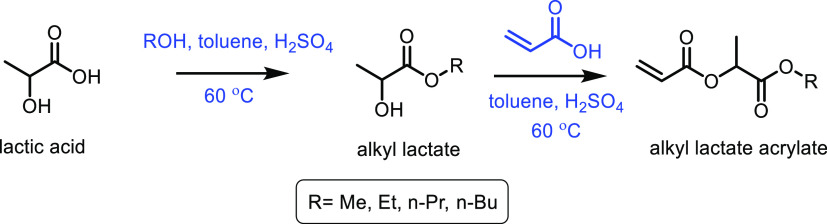
Synthesis of alkyl lactate acrylates from lactic acid *via* an alkyl lactate intermediate.

Another example that coupled lactate esters with acrylic acid was conducted by Nayak and co-workers, who synthesized a series of alkyl lactate acrylates through a two-step reaction, which is summarized in Figure 14.^368^ First, lactic acid was reacted with an alcohol, using sulfuric acid and toluene as the catalyst and solvent respectively to form the corresponding alkyl lactate. High yields of 94%, 92%, 91% and 95% were observed for methyl lactate, ethyl lactate, *n*-propyl lactate and *n*-butyl lactate, respectively. The second step was the reaction between the alkyl lactates and acrylic acid, again using sulfuric acid as catalyst, although the corresponding yields were slightly lower (72–93%). The resulting monomers were then successfully polymerized *via* free radical polymerization.

##### 3.1.3.3 From Glycerol

**Figure 15:**
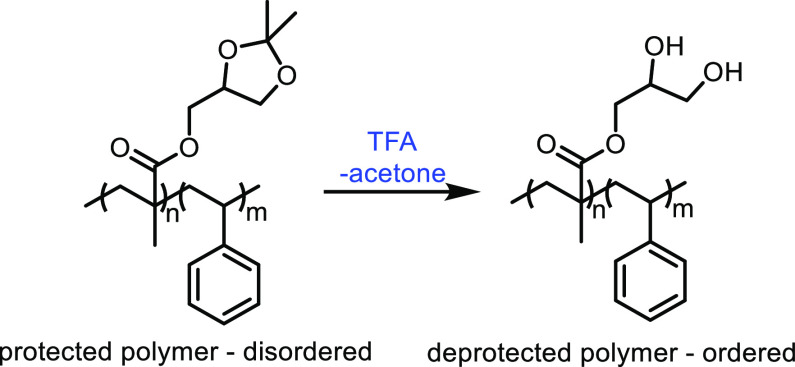
Structures of the protected and deprotected polymers and how it affects the structure of the polymer in solution.

Glycerol is a by-product of biodiesel production, and it is estimated 400 million tons of glycerol are produced each year. Thus, to increase the sustainability of the use of biodiesel and due to glycerol production exceeding the current demand, suitable applications need to be found for glycerol.^369^ As previously discussed, glycerol can be used as a feedstock for the synthesis of acrylic and methacrylic acid. The conversion of glycerol into ketals has been thoroughly reported and these can further be used in the synthesis of different acrylate and methacrylate monomers. Goyal and co-workers combined glycerol from biodiesel with products from the ketonization of pyrolysis oil from biomass, with *p*-toluene sulfonic acid as the catalyst, to form glycerol ketals.^370^ The second step was to form the methacrylate monomers which involved a transesterification of the ketal pre-monomers and methyl methacrylate, catalyzed by lipase enzymes. The resulting monomers were polymerized *via* living radical polymerization.^370^ The ketal group also acts as an important protecting group during the polymerization and can be removed afterward. Yu *et al*. copolymerized solketal methacrylate, a monomer formed from the condensation of glycerol into acetone and a subsequent acrylation, with styrene.^371^ It was shown that the protecting group had an effect on the polymer solution behavior as when protected, the polymer was disordered, while deprotection through hydrolysis resulted in an ordered polymer structure, as shown in Figure 15.

Glycerol can be protected through a reaction with acetone and *p*-toluenesulfonic acid catalyst at 80 °C to form isopropylidene glycerol in a 73% yield.^372^ This can in turn be coupled with acryloyl chloride in the presence of triethylamine.^372−377^ There have been several examples of this reaction reported, with a 90% yield being the highest obtained.^376,377^ The resulting monomer was also shown to be polymerized *via* copper-mediated controlled radical polymerization using a macroinitiator.^372^ Polymers containing solketal acrylate (SKA) are acid-degradable and temperature responsive, while the SKA groups significantly impact the self-assembly behavior.^378−380^ A hydrophobic–hydrophilic PMMA-b-PSKA block copolymer was formed by sequential addition before the hydrolysis of the solketal groups. Self-assembly of the amphiphilic polymers led to the formation of core–shell micelles with an average size of 30 nm.

##### 3.1.3.4 From Acrolein

As discussed earlier in the section on acrylic acid (section 3.1.1), acrolein is an intermediate in the production of acrylic acid from glycerol and can be used in a separate process to form alkyl acrylates. For instance, gold nanoparticles have been investigated as catalysts for the production of methyl acrylate from acrolein. Kenjo and co-workers found that when an Au(2h)/MA450 catalyst was used, the optimum selectivity of MA was 78%.^381^ Tsutsumi investigated the use of gold nanoparticles supported on CeO_2_ for both the production of methyl acrylate and methyl methacrylate.^382^ The morphology of the CeO_2_ support was seen to have a major impact on catalytic performance. For the reaction of acrolein with methanol to form MA, the Au/CeO_2_-nano performed the best, with the conversion and selectivity both >80% at low acrolein concentrations. The catalysts containing rod and cube shaped CeO_2_ performed significantly worse, while there was a trend of decreasing catalytic performance as the concentration of acrolein increased. For the reaction of methacrolein with methanol to form methyl methacrylate, all catalysts achieved 100% selectivity, but once again it was the Au/CeO_2_-nano that had the best catalytic performance with a conversion of 91% being obtained compared to values of 79% and 10% for the rod and cubic morphologies, respectively. In another example, Marsden and co-workers achieved a high conversion and methyl acrylate selectivity of 97% and 87% respectively for the aerobic oxidation of acrolein in methanol with a Au/ZnO catalyst at 25 °C.^383^

##### 3.1.3.5 From Methyl Acetate

Methyl acrylate can further be synthesized by an aldol condensation between methyl acetate and formaldehyde in a similar reaction to that of acetic acid with formaldehyde in the synthesis of acrylic acid.^259^ Methyl acetate has a lower reactivity than acetic acid and so the yields for methyl acrylate are lower than for the corresponding reaction forming acrylic acid.^384^ The conversion of methyl acetate into methyl acrylate has been investigated with vanadium phosphate catalysts, showing selectivity values up to 92% with formaldehyde conversions of up to 48%.^385^ The use of a cesium catalyst supported on a SBA-15 mesoporous support was explored and a methyl acetate conversion of 49% and methyl acrylate selectivity of 95% were achieved, in a reaction with a 1:2 ratio of methyl acetate to formaldehyde at 390 °C.^386^ HZSM-5 catalysts doped with phosphorus and cesium have also been investigated, showing a yield of 39% and selectivity of 98%.^387^

##### 3.1.3.6 From Vegetable Oils and Lipids

Vegetable oils have been used as raw materials for many different industries including in paints, coatings, biodiesel and lubricants.^388^ Vegetable oils are composed of fatty acids which contains carboxylic acid and double bonds functionalities that can be used for a variety of different reactions. Examples of vegetable oils that have been studied for acrylate synthesis include soybean oil, jatropha oil, linseed oil and castor oil.^389−394^ Using vegetable oils for monomer synthesis results in products containing long, and often unsaturated, hydrocarbon side chains being formed.

**Figure 16:**
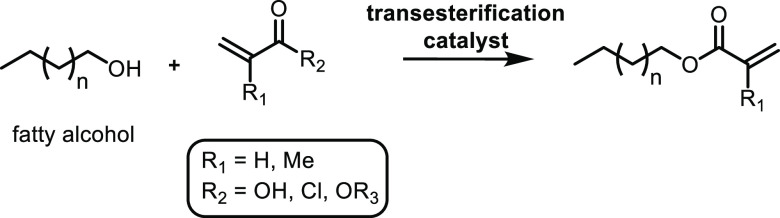
Conversion of fatty alcohols into acrylate monomers *via* an esterification reaction. R_1_ was either H or Me while R_2_ corresponds to either OH, Cl or OR_3_.

A particularly useful derivative for the synthesis of alkyl-substituted (meth)acrylates are fatty alcohols which can be obtained from fatty acids and esters through a catalytic hydrogenation at high temperatures and pressures. There have been a range of catalysts that have been investigated for this reaction, including Cu-based, Ru–Sn–B and Co–Sn catalysts.^395^ The hydroxyl group of the fatty alcohols can then participate in an esterification reactions to form acrylate and methacrylate monomers, as illustrated in Figure 16. Pratap and co-workers carried out an esterification of various *n*-alkyl alcohols (*n* = 12–18) with acrylic acid using *p*-toluenesulfonic acid as a catalyst while Soldi and co-workers synthesized octadecyl methacrylate, tetradecyl methacrylate and hexadecyl methacrylate in 85%, 70% and 80% yield, respectively.^396,397^ The enzyme Novozym 435 lipase has also been shown to work for the esterification of stearyl alcohol and oleyl alcohol.^398^ The acrylate of both alcohols was formed with 65% yield, while for the methacrylate derivative, the stearyl alcohol achieved a higher yield of 94% compared to oleyl alcohol (86%). Linolenyl acrylate was formed from linolenyl alcohol through a reaction with acryloyl chloride and a yield of 65% was obtained.^399^ Acrylates have also been formed from linoleyl alcohol, oleyl alcohol and lauryl alcohol, while similar approaches have been taken to form methacrylates through reactions with methacryloyl chloride.^400^ However, the disadvantage of many of these procedures is that toxic acryloyl chloride and methacryloyl chloride coupling agents are required thus limiting their sustainability.

**Figure 17:**
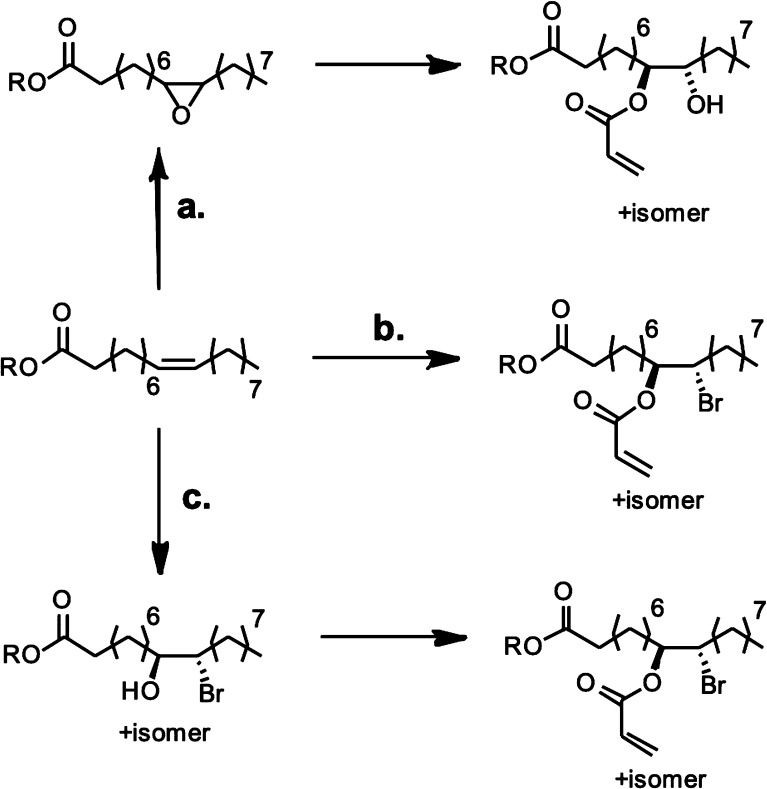
Different methods to form acrylate monomers by functionalizing the C=C bond in vegetable oils. Methods include expoxidation-acrylation (a), bromoacetoxylation (b) and hydroxybromination-acrylation (c).

**Figure 18:**
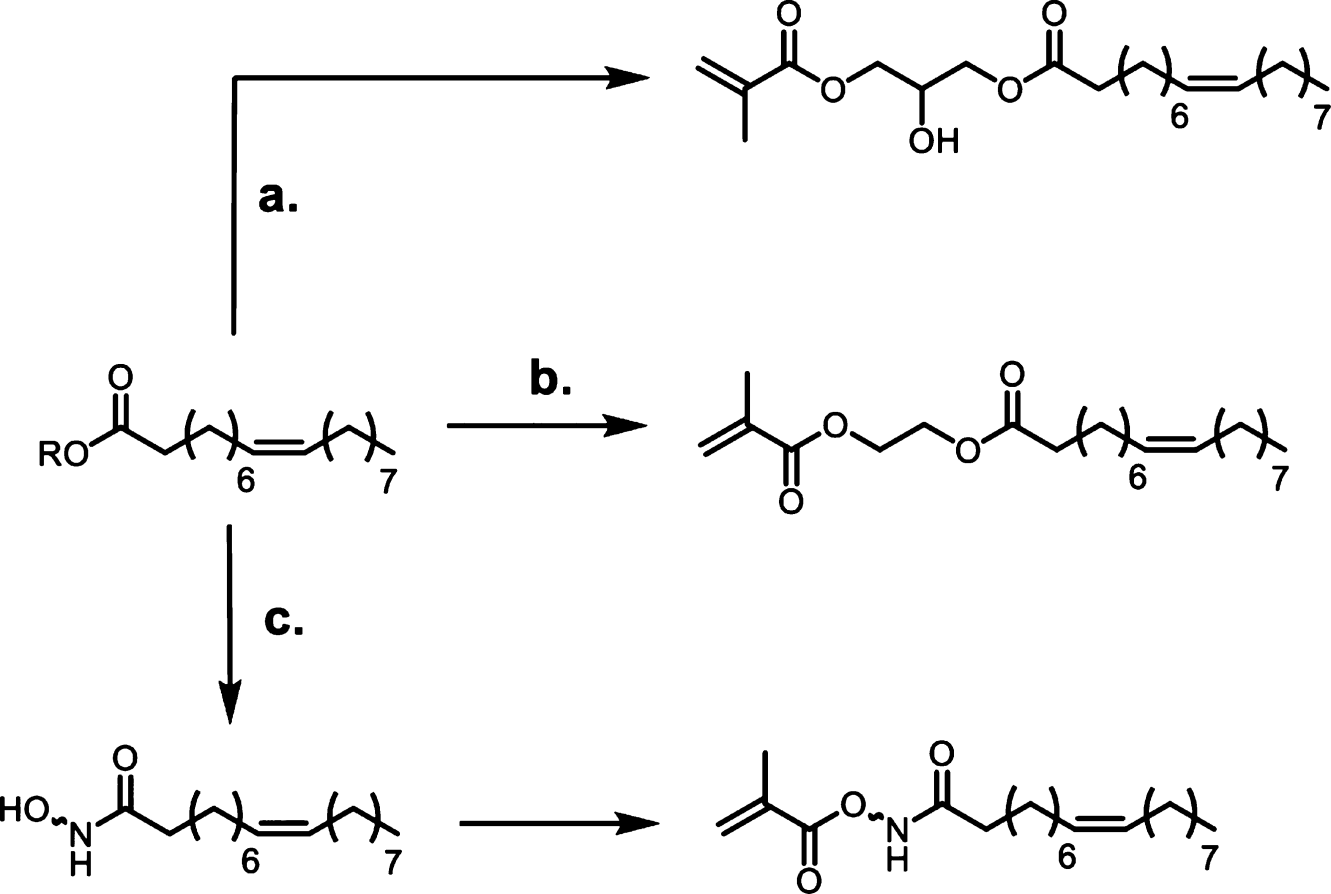
Different methods to form acrylate monomers by functionalizing the carboxylic functionality in vegetable oils. Methods include glycidyl methacrylate ring opening (a), (trans)esterification (b) and amidation-(meth)acrylation (c).

There have been many previously reported approaches to synthesize acrylate and methacrylate monomers from vegetable oils which either aim to target the carbon–carbon double bond or the carboxylic acid functional group (see Figure 17 and Figure 18, respectively).^388^ These methods include epoxidation-acrylation, hydroxybromination-acrylation, bromoacrylation, epoxy ring opening and transesterification.

As well as synthetic routes from fatty alcohols, other routes have been proposed to functionalize fatty acids directly. One method is through an epoxidation-acrylation approach.^391,401−406^ The general approach is to first convert double bonds in the fatty acid to the corresponding epoxide through reaction with peracids or peroxides in the presence of a carboxylic acid, such as formic acid.^391,401−406^ The epoxy group can then be ring-opened with acrylic acid resulting in the acrylate monomer. This procedure has been carried out on various different substrates including soybean oil and methyl oleate.^389,407−409^ Huang and co-workers investigated two separate systems for the epoxidation of fatty esters.^408^ The first was using formic acid and hydrogen peroxide. When using methyl oleate, methyl linoleate and methyl linolenate as the fatty esters, yields for the epoxidized products were 51%, 65% and 69%, respectively. The other system was based on a stronger acid catalyst, a mix of sulfuric acid and acetic acid. However, this setup resulted in significantly lower yields (i.e., 43%, 33% and 6% for the epoxidized products from methyl oleate, methyl linoleate and methyl linolenate, respectively). Walter *et al*. carried out the epoxidation of methyl oleate with formic acid and hydrogen peroxide at 0 °C and obtained a yield of 97%.^407^ The resulting product was heated with acrylic acid for 18 h at 95 °C, resulting in the acrylated product being obtained in 86% yield. An alternative chemo-enzymatic approach to epoxidation was reported through the reaction of soybean oil and hydrogen peroxide in the presence of the enzyme CAL-B.^403^ The resulting epoxidized soybean oil was acrylated through a reflux with acrylic acid.

A different approach of the carbon–carbon double bond functionalization was a hydroxybromination-acrylation method proposed by Eren and Küsefoglu.^410^ The hydroxybromination of both oleic acid and methyl oleate was conducted through a reaction with a mixture of *N*-bromosuccinimide, acetone and water at room temperature for 2 h. Hence, yields of 85% and 92% were obtained for the products from methyl oleate and oleic acid, respectively. The hydroxy group on the products was then further functionalized, as in many examples, through a reaction with acryloyl chloride.

In an attempt to simplify the two-step synthetic procedures, an approach based on a simultaneous bromination and acrylation was investigated.^411^ This bromoacrylation was achieved for methyl oleate by reacting it with acrylic acid and *N*-bromosuccinimide at room temperature for 1 day. A high yield of 90% was obtained, which was higher than reported for the two-step process outlined above. When repeated for soybean oil and sunflower oil, the bromoacrylation achieved yields of 75% and 55%, respectively.

**Figure 19:**
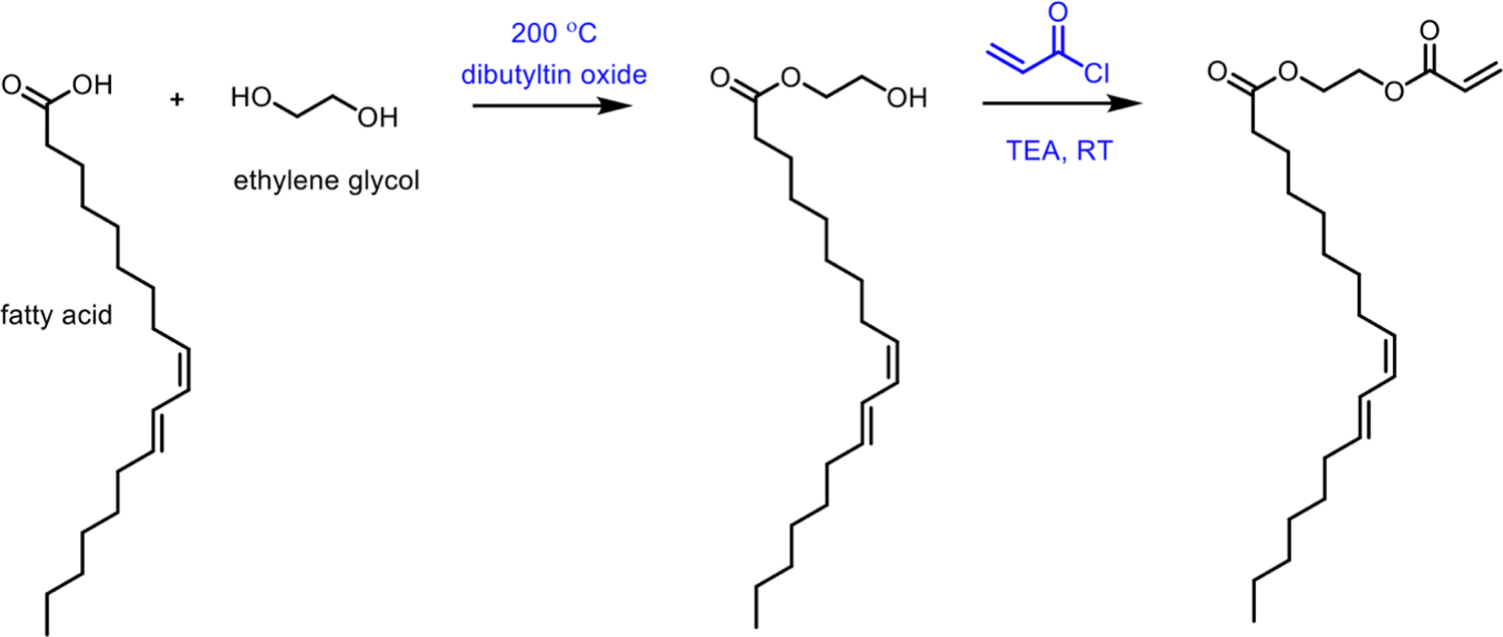
An example of an esterification between a fatty acid and ethylene glycol before a subsequent reaction with acryloyl chloride to form an acrylic monomer.

As well as being able to functionalize the double bond, the carboxylic acid functionality can also be utilized to synthesis acrylic monomers. Esterification reactions between fatty acids and allylic alcohols have previously been described, but usually require a significant excess of alcohol to proceed to high conversions.^388,412,413^ Therefore, alternative strategies have been investigated, including esterification. In this process, the fatty acids were heated to 55–60 °C with phosphorus trichloride for 1 h to form the acid chloride.^414^ Subsequently, the product was cooled to 10 °C and allylic alcohol was added. When palmitic acid was used as the fatty acid, a yield of 78% was obtained. However, this procedure has the disadvantage of using the toxic PCl_3_ and so an alternative route was developed, as illustrated in Figure 19. It should be noted that this procedure is mainly applicable to conjugated fatty acids. By first carrying out an efficient esterification with ethylene glycol (i.e., 92% yield) at 200 °C with a dibutyltin oxide catalyst, a hydroxyl terminated product was formed. A subsequent reaction with acryloyl chloride produced the acrylated product in 86% yield. Other synthetic routes based on esterification have been reported as well. Maiti *et al*. carried out an esterification between oleic acid and hydroxyethyl methacrylate with *N*,*N*′-dicyclohexylcarbodiimide (DCC) and 4-dimethylaminopyridine (DMAP), which resulted in a 75% yield of the methacrylate monomer.^415^ Similar reactions were carried out for caprylic, capric, lauric, mysritic, palmitic and stearic acid with yields between 74%–85%.^416^ The enzyme Novozym 435 has been shown to catalyze transesterification reactions of methyl methacrylate and was used to synthesize novel acrylate and methacrylate monomers from unsaturated fatty esters, with yields ranging from 65% to 94%.^388,398,417^ While these methods utilize bio-based feedstock, they do not necessarily present a sustainable pathway as the use of toxic chemicals and catalysts (e.g., PCl_3_, dibutyl tin oxide) must be considered.

Another approach to functionalize the acid group in vegetable oils, for the transformation into acrylic monomers is the amidation-methacrylation method.^418,419^ The first step is the reaction of a triglyceride with an amino alcohol, such as ethanolamine at 60 °C for 4 h. Hydroxy-terminated fatty amide chains were produced with yields between 95% and 97%. A subsequent reaction with methacrylic anhydride and DMAP at 60 °C resulted in the methacrylate monomer, with almost full conversion. The second step of the reaction has also been reported using acryloyl chloride to form an acrylic version of the monomer.^420^

A final approach to acrylic monomers derived from fatty acids is through an epoxy ring-opening reaction. The general approach is the reaction of a fatty acid with glycidyl methacrylate in the presence of a catalyst.^421,422^ Several catalysts have been investigated, including *N*-dimethyl benzyl amine, hexadecyl trimethylammonium chloride and benzyltriethylammonium chloride, which obtained high yields of 94%, 94% and 91%, respectively.^423^ Two catalysts were used for the reaction of oleic acid with glycidyl methacrylate, to form a methacrylate monomer.^424−426^ One approach used a chromium(III) based catalyst (AMC-2) at 80 °C while the second featured a zinc based catalyst (Nacure XC-9206) being used at 100 °C. The resulting monomer was polymerized through free radical mini-emulsion polymerization, while the double bond from the oleic acid can be used as a cross-linking unit in gel formation.^424^ The monomer was also copolymerized with α-methylene-γ-butyrolactone in a mini-emulsion polymerization, forming renewable polymer latexes with tunable properties.^425^

There have been many examples of acrylates and methacrylates derived from vegetable oil/fatty acids being polymerized. To investigate potential bio-based adhesives, Badia and co-workers for instance carried out an emulsion polymerization of 2-octyl acrylate, derived from castor oil, and isobornyl methacrylate, derived from pine resin.^394^ Soybean oil contains many unsaturated double bonds within the structure; however, they are usually unreactive and need to be converted into other functional groups, such as epoxides, to increase their reactivity.^427^ Epoxy groups can be incorporated into the structure of soybean oil through the reaction of double bonds with hydrogen peroxide in the presence of acetic or formic acid.

#### 3.1.4 Cyclic (Meth)acrylate

##### 3.1.4.1 From Terpenes

**Figure 20:**
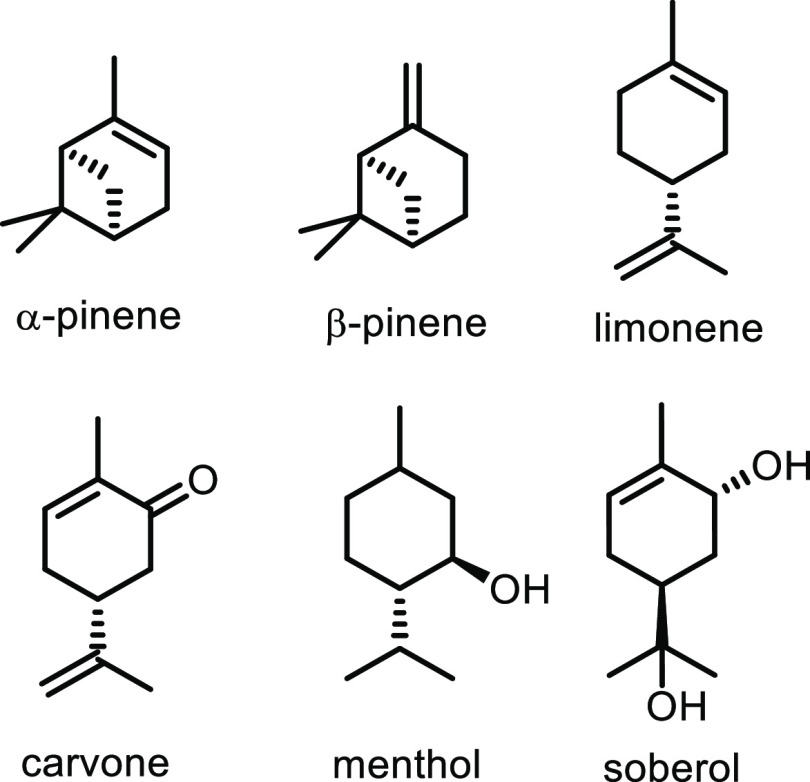
Structures of different terpenes and terpenoids that have been used to synthesize acrylate monomers.

Another potential bio-source for (meth)acrylate monomers are terpenes and terpenoids (Figure 20), which can be derived from biomass including wood (α-pinene and β-pinene) or citrus (limonene).^428^ While terpenes contain vinyl groups and are based on isoprene C_5_H_8_ units, terpenoids (e.g. menthol and carvone) are derived from terpenes and contain additional functional groups. The homopolymerization of the vinyl groups of terpenes proved unsuccessful through free radical polymerization,^429^ although when copolymerized with styrenics or acrylates, low molecular weight polymers were obtained.^430^

**Figure 21:**
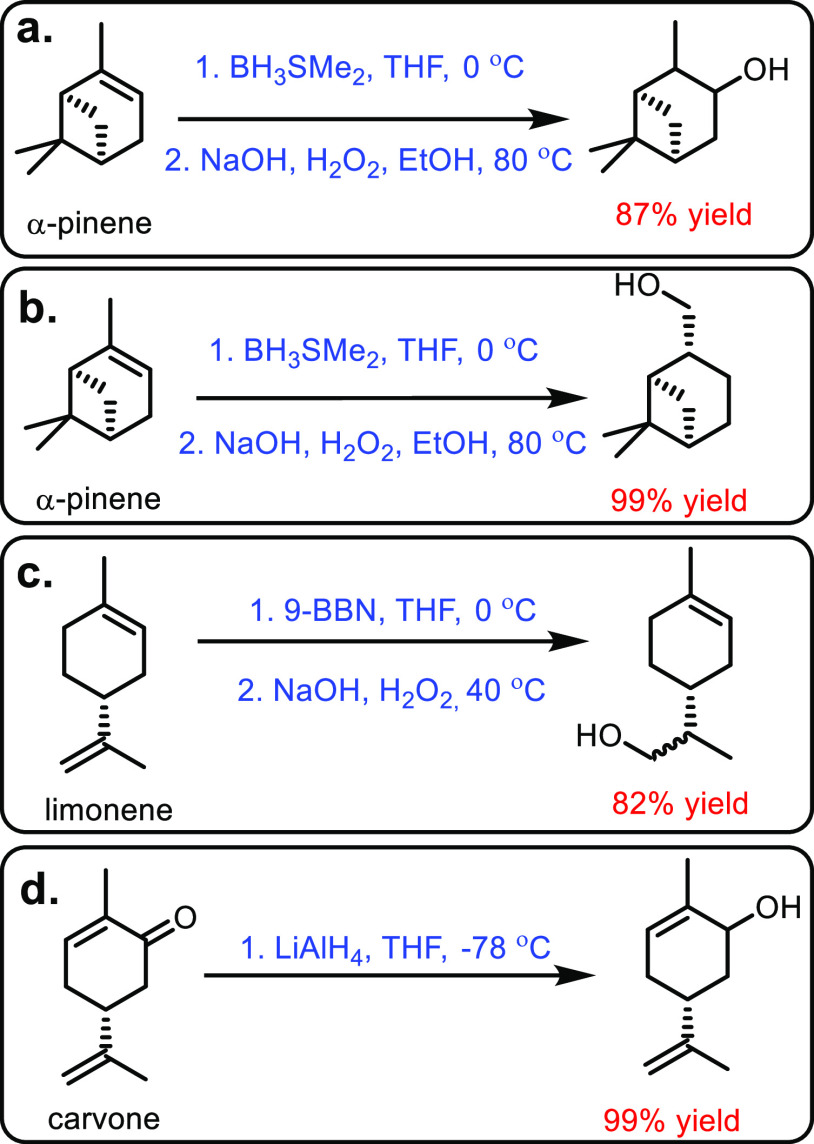
Reaction schemes to form hydroxy-functionalized terpenes/terpenoids, which were then reacted further to form acrylic monomers.

Sainz and co-workers transformed (+)-α-pinene, (−)-β-pinene, (*R*)-(+)-limonene and (*R*)-(−)-carvone into acrylates and methacrylates *via* a two-step approach.^428^ The first step was to convert the terpenes into alcohols before esterification with the relevant acryloyl or methacryloyl chloride. For α-pinene, β-pinene and limonene, the first step consisted of a hydroboration/oxidation step.^428,431,432^ For limonene, a hindered borane was used to selectively react with the less hindered vinyl group. The first step for the carvone synthesis was different due to the presence of a ketone group, which was reduced with LiAlH_4_ to form the corresponding alcohol.^428,433^ The formations of the alcohols proceeded with high yields (>82%, see Figure 21).

**Figure 22:**
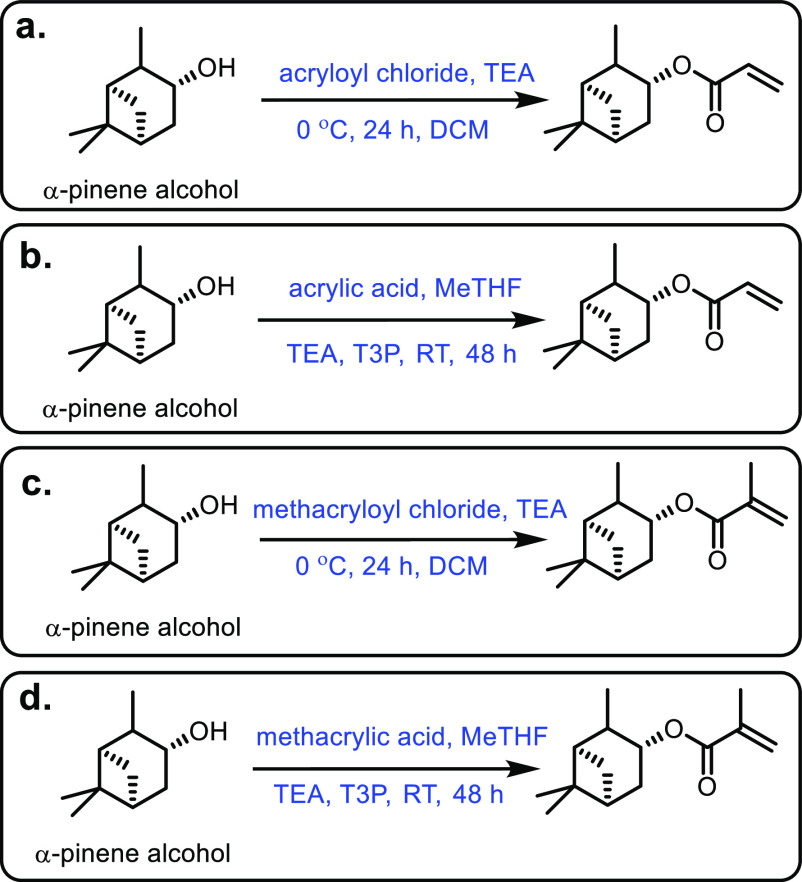
Different approaches to form acrylic and methacrylic monomers from α-pinene alcohol.

**Figure 23:**
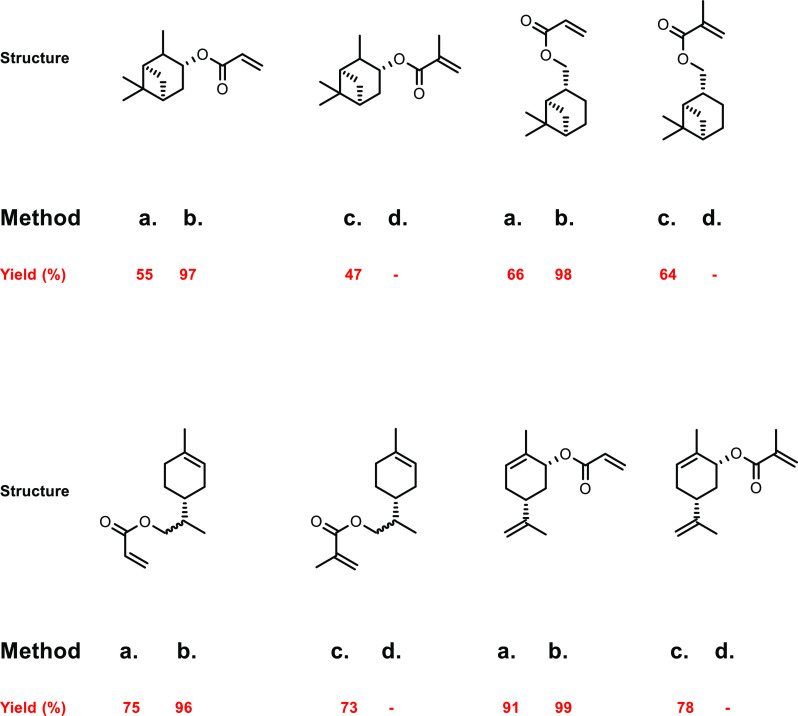
Reported yields for the acrylic and methacrylic monomers derived from terpenes *via* four different methods.

**Figure 24:**
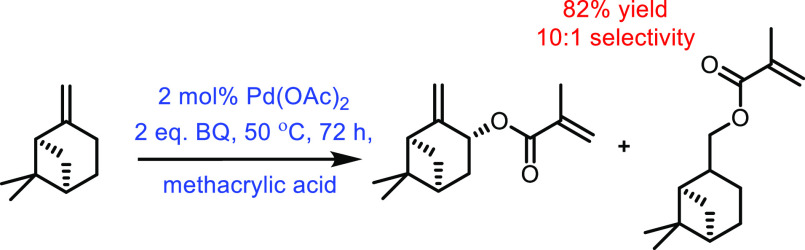
Scheme for the White’s allylic oxidation of β-pinene.

With the terpene-based alcohols available, several different methods were then employed to synthesize the actual acrylate/methacrylate monomers, as exemplified for (+)-α-pinene in Figure 22 and Figure 23.^428^ As previously described, the reaction of alcohols with acryloyl or methacryloyl chloride is typically used to synthesize acrylates and methacrylates. However, in a bid to remove the use of acryloyl or methacryloyl chloride so to produce a more sustainable method, esterification with acrylic and methacrylic acid presents a desirable alternative. This was achieved by using propyl phosphonic anhydride (T3P) as the coupling agent, with higher yields achieved for the acrylate monomers than for the synthesis with acryloyl chlorides.^367,428^ However, no yields were reported for the synthesis of methacrylates using T3P. Sainz et al. also developed a new one pot synthesis built on White’s catalytic allylic oxidation, which uses a Pd(OAc)_2_ catalyst to oxidize the allylic position of β-pinene (Figure 24).^428,434^ Conditions were screened and optimized, indicating the use of 2 mol % Pd(OAc)_2_, 2 equiv of benzoquinone at 50 °C for 72 h to yield two new monomers with 82% yield.

Another (meth)acrylate monomer, derived from the monoterpenoid menthol, that is receiving increased attention is menthyl acrylate.^364,435−440^ Unlike the aforementioned terpenes, menthol has a hydroxy functional group available on the scaffold and so the acrylic monomer can be formed in a one-step synthesis upon the addition of acryloyl or methacryloyl chloride. Indeed, Baek and co-workers synthesized menthyl acrylate by reacting menthol with acryloyl chloride with a yield of 73%.^439^ The monomer was shown to be co-polymerized with 2-ethylhexyl acrylate and 2-hydroxyethyl acrylate in a UV-initiated bulk process to form a pressure sensitive adhesive. Additionally, Argyros and co-workers synthesized menthyl methacrylate in 56% yield using methacryloyl chloride, which could be copolymerized with MMA *via* free radical polymerization, both in bulk and solution.^438^ Notably, the chirality of the menthol starting material was the focus of their work in exploring the feasibility in polymer optic fiber applications. As well as free radical polymerization, the living radical polymerization of menthyl acrylate and menthyl methacrylate has been heavily explored.^364,435,436,440^

**Figure 25:**
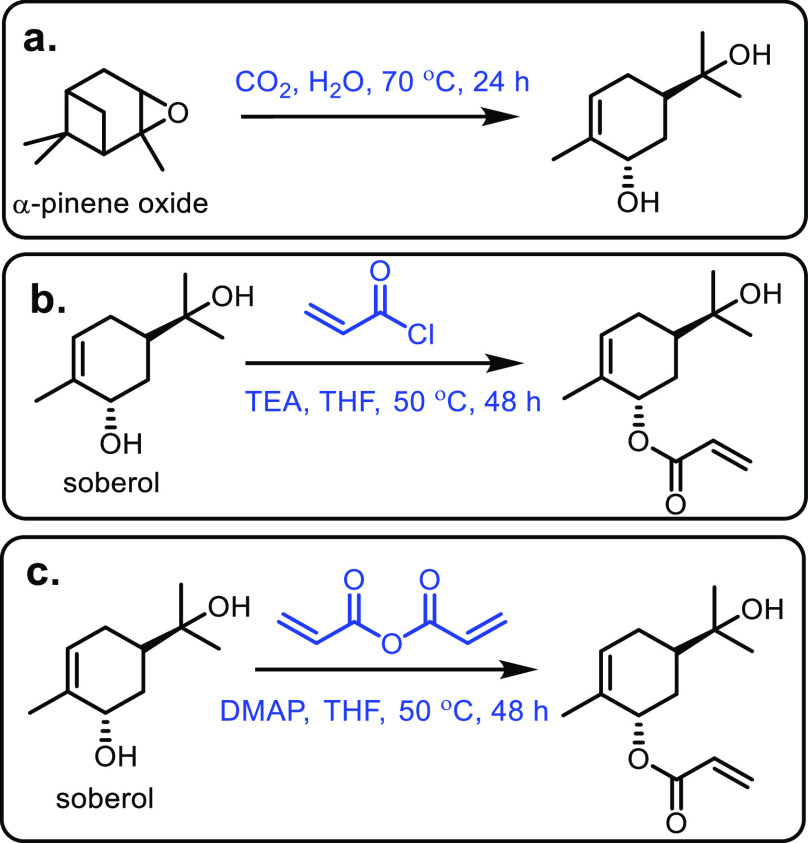
Schemes for the formation of sobrerol from α-pinene oxide (a), the acrylation of sobrerol with acryloyl chloride (b) and the methacrylation of soberol with methacrylic anhydride and DMAP (c).

**Figure 26:**
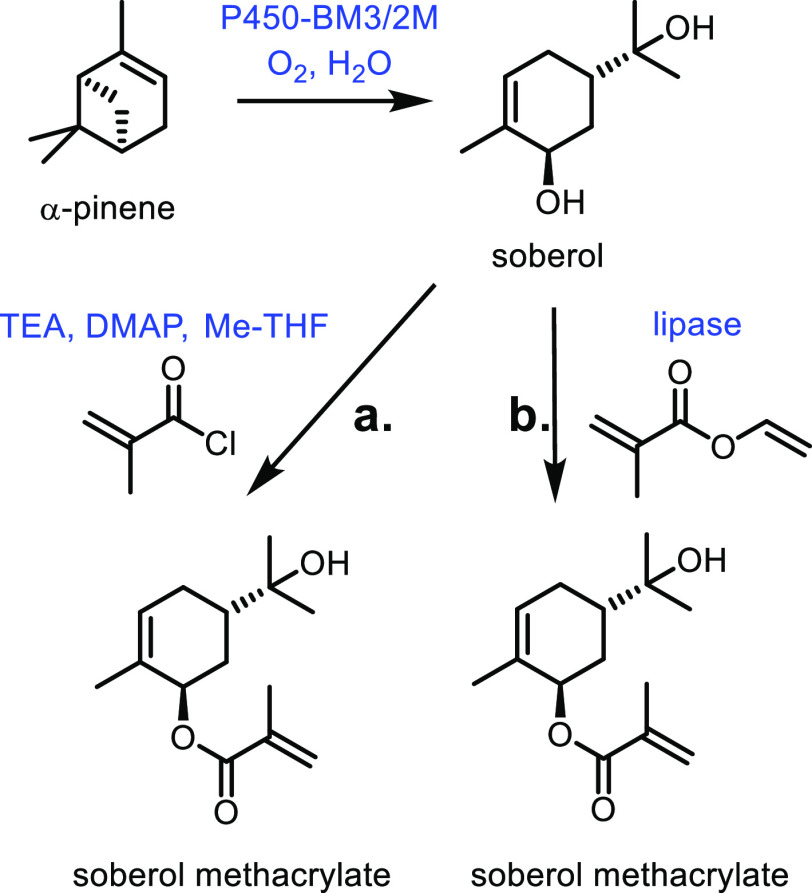
Two different routes to synthesize sobrerol methacrylate from α-pinene. The second step was conducted *via* a chemical route (a) and biochemical approach (b).

Another (meth)acrylate monomer that has been explored is derived from sobrerol, for which different synthetic approaches have been taken (Figure 25).^441,442^ Similar to menthol, sobrerol contains a hydroxy functional group and is synthesized through the hydration of α-pinene oxide.^442^ The synthesis of sobrerol acrylate was completed by reacting sobrerol with acryloyl chloride with a yield of 72% being obtained. The synthesis of the sobrerol methacrylate derivative achieved a yield of 84% and was carried out by treating sobrerol with methacrylic anhydride in the presence of DMAP at 50 °C. Both monomers have been investigated for their potential use to replace styrene in unsaturated polyester resins (UPRs).^442^ Additionally, Stamm and co-workers investigated other potential routes to synthesize sobrerol methacrylate as shown in Figure 26.^441^ First, α-pinene was converted into sobrerol, catalyzed by cytochrome P450-BM3/2M monooxygenase from *Bacillus megaterium*. *Trans*-sobrerol was also shown to be the main product from the biotransformation of (+)-α-pinene with *Armillariella mellea*.^443^ Sobrerol methacrylate was then derived from its corresponding alcohol *via* two routes.^441^ The first was a conventional esterification with methacryloyl chloride with a yield of 66% being reached. The second route was a greener approach that used vinyl methacrylate and the amino lipase enzyme originating from pseudomonas fluorescens, with the enzymatic approach giving a much higher conversion (i.e., 96%) than the chemical synthesis pathway. The resulting monomer was successfully used in conventional living radical polymerizations and also copolymerizations with MMA and BMA were conducted.

##### 3.1.4.2 From Isosorbide

**Figure 27:**
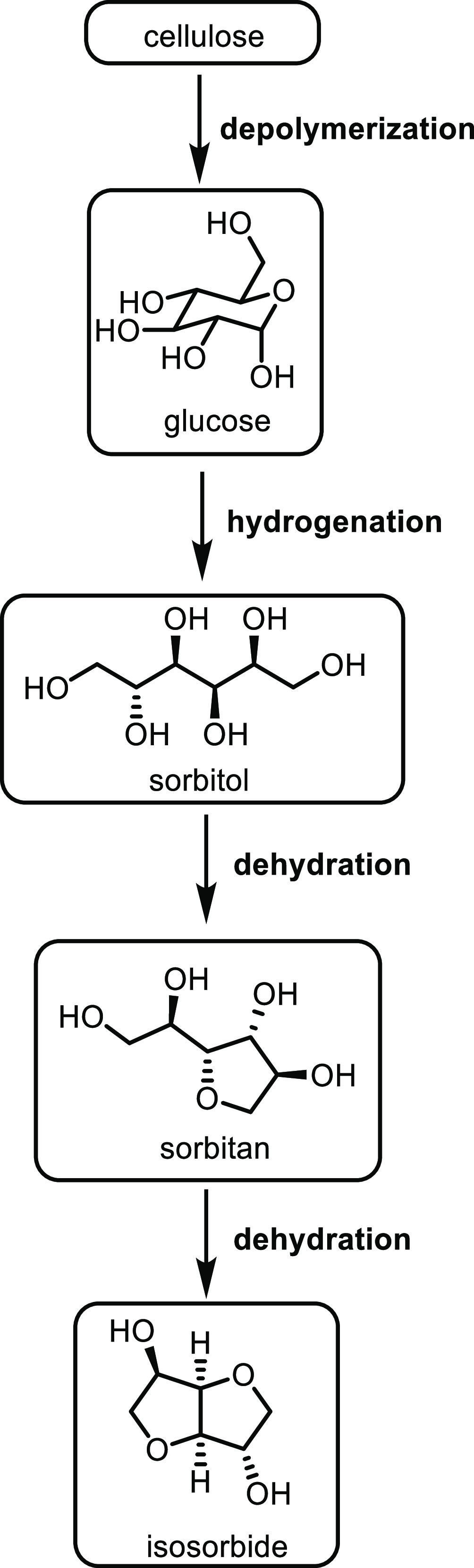
Synthetic route of isosorbide from glucose.

Isosorbide is considered one of the top 12 most renewable building blocks and is a chiral bicyclic diol, derived from glucose (Figure 27).^444^ There have been many potential applications of isosorbide which include monomer synthesis, being used as a bio-based organic solvent, or for use in the pharmaceutical/medical fields.^445^ As well as being a sustainable bio-source, isosorbide also has other advantageous properties as it brings rigidity, thermal stability, and is also considered to be non-toxic. As an example, the oxygen barrier and mechanical properties of poly(butylene succinate) could be improved when the rigid isosorbide was incorporated into the structure, while the non-toxicity of isosorbide allowed for it to be used as a substitute for bisphenol A in epoxy resins and polycarbonates.^445−447^

Isosorbide can be synthesized from starch through chemical-biological processes, which include enzymatic hydrolysis to form glucose followed by a hydrogenation to sorbitol before a final dehydration step.^445,446,448^ This hydrogenation to sorbitol can be performed through well-established synthetic procedures.^448−450^ The double dehydration of sorbitol is the major step in the synthesis of isosorbide, and problems including chemo- and regioselectivity and the formation of side products have to be overcome.^448^ There have been many different homogenous and heterogeneous catalysts that have been used for this crucial step in the synthesis. For instance, the dehydration step has been catalyzed by strong Bronsted acids (e.g., hydrochloric and sulfuric acid) in order to transform one of the hydroxy groups into a good leaving group.^448,451,452^ Lewis acids have also been used to catalyze this reaction with metal triflates (e.g., bismuth(III)triflate and gallium(III)triflate) proving better results than metal chlorides (e.g., AlCl_3_ and SnCl_4_) and sulfuric acid.^448,453−455^ Acid-free conditions have also been applied when water was used as a solvent at high temperatures, as the self-ionization of water promoted the dehydration reaction.^448,456,457^ Heterogeneous catalysts that have been used include acid resins zeolites, metal oxides, metal phosphates and tungsten phosphoric acids supported on metal oxides.^448,458−463^ A one-pot synthesis of isosorbide was investigated by developing a hydrogenation catalyst containing acid. In this case, a heterogeneous bifunctional catalyst was used that contained 0.2 wt % of ruthenium on a Bronsted acid support with an optimized yield of 85%.^464^ De Almeida *et al*. carried out the synthesis of isosorbide from cellulose in a molten ZnCl_2_ medium.^465^ The ZnCl_2_ was found important in dissolving and hydrolyzing the cellulose into glucose, before a ruthenium catalyst was used for the hydrogenation step. The dehydration step was found to proceed both in the plain molten ZnCl_2_ medium, or in the presence of CuCl_2_ or NiCl_2_ catalysts.

**Figure 28:**
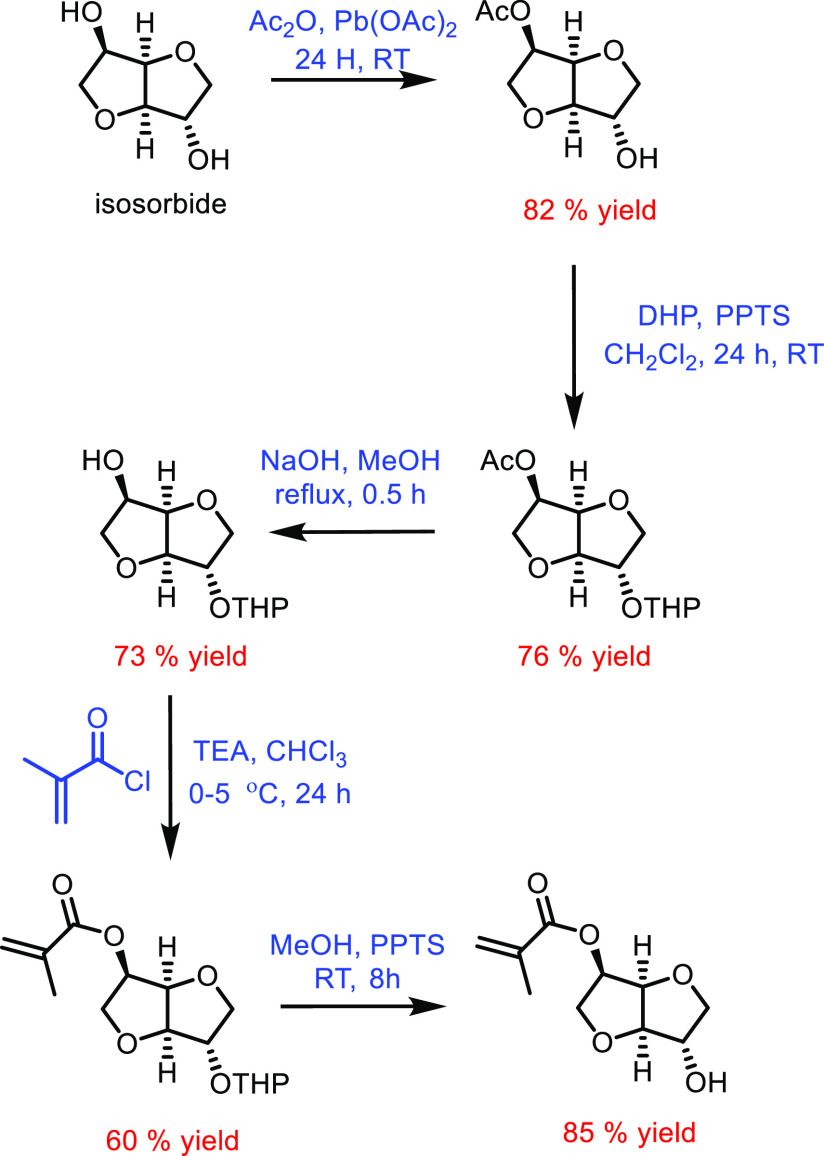
Synthesis of a mono-functional acrylate from isosorbide utilizing orthogonal protecting groups.

**Figure 29:**
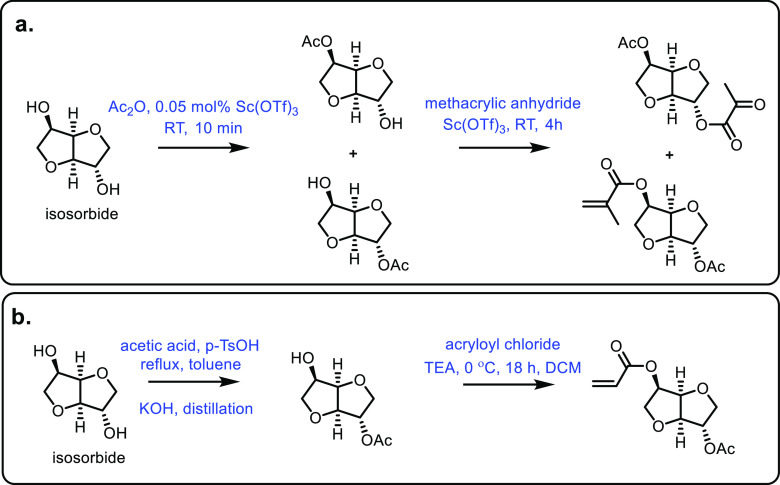
Synthesis of acetylated isosorbide methacrylate (a) and acetylated isosorbide acrylate (b).

Once synthesized, isosorbide can then be functionalized into monomers such as acrylates and methacrylates. Due to isosorbide containing two hydroxy functionalities, carefully planned synthetic procedures must be carried out to obtain a mono-functional monomer. Imanzadeh and co-workers synthesized a mono-functional isosorbide methacrylate by using orthogonal protecting groups.^466^ The synthesis is shown in Figure 28, and an overall yield of 23% was obtained. First, selective mono-acetylation of isosorbide was conducted before the remaining hydroxy group was protected with a tetrahydropyran group.^467^ The acetyl group was removed *via* basic hydrolysis before further reacting with methacryloyl chloride. The final step was to remove the tetrahydropyran protecting group using pyridinium *p*-toluenesulfonate in methanol. Gallagher and co-workers synthesized both acetylated isosorbide methacrylate (AMI) and acetylated isosorbide acrylate (AAI) (Figure 29).^468^ AMI was prepared through a mono-acetylation, followed by a further reaction with methacrylic anhydride and a scandium(III) triflate catalyst. The second step reached full conversion after 4 h with 1 mol % catalyst, while the overall yield for the synthesis was reported to be 44%. The resulting monomer was polymerized through free radical polymerization to form a high *T*_g_ polymer (*T*_g_ = 130 °C), while also a block copolymer of the acetylated isosorbide methacrylate and butyl acrylate has been successfully achieved. The AAI monomer was formed by reacting the acetylated intermediate with acryloyl chloride.^469^ The yield of the acetylation was recorded as 38%, while the reaction with acryloyl chloride achieved a 58% yield. The resulting monomer was used to form a PAAI–PBA-PAAI triblock, which showed good mechanical properties for potential use as pressure sensitive adhesives.

**Figure 30:**
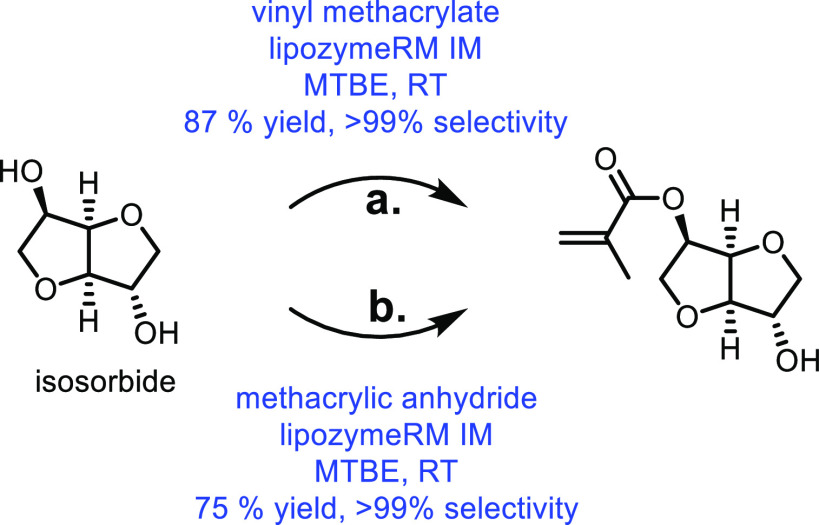
Biochemical approaches to synthesize a monofunctional isosorbide methacrylate.

As well as examples utilizing protecting groups, other routes to monofunctional (meth)acrylates synthesized from isosorbide make use of the difference in reactivities between the exo- and endo- hydroxy groups. Baek *et al*. synthesized a mono-functional isosorbide acrylate using acryloyl chloride, selectively producing the endo-isomer with 35% yield.^470^ The resulting monomer was co-polymerized with 2-ethylhexyl acrylate and 2-hydroxyethyl acrylate for potential use in pressure-sensitive adhesives. Biochemical routes to synthesize monomers derived from isosorbide have also been developed. For example, Matt and co-workers synthesized mono-functional isosorbide methacrylate *via* two methods, which both used a lipozyme RM IM (*Rhizomucor miehei* lipase) catalyst (Figure 30).^471^ One method, using vinyl methacrylate, achieved a yield of 87% and >99% selectivity for the endo-isomer. The other method, which used methacrylic anhydride achieved a lower yield of 75%, but showed a similar selectivity for the endo-isomer. The monomer was shown to be susceptible to free radical polymerization, obtaining a polymer with a high *T*_g_ of 167 °C. The advantages of these biochemical methods is its one-step process, rather than a multi-step synthesis that resulted in a higher overall yield, as well as eradicating the use of metal catalysts.

**Figure 31:**
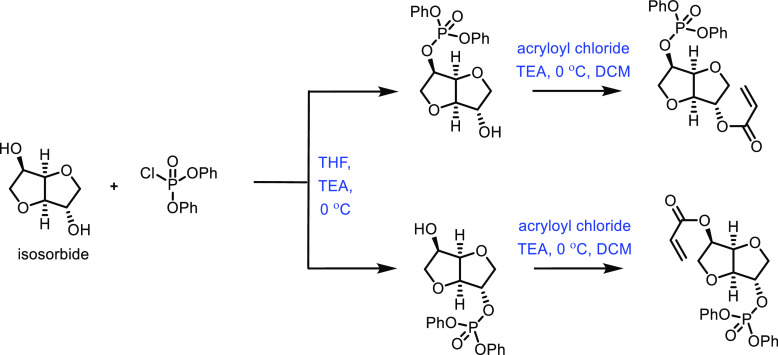
Conversion of isosorbide into two isomers of diphenylphosphato acrylates.

Due to isosorbide containing two hydroxy groups, there is also the potential to synthesize bifunctional monomers besides only mono-functional derivatives.^444,472−475^ Indeed, Liu *et al*. synthesized a rigid isosorbide methacrylate bifunctional monomer, using a solvent free approach, by reacting isosorbide with methacrylic anhydride at 60 °C using ultrasonication.^444^ The methacrylic anhydride to isosorbide ratio was optimized at 3:1, resulting in a high yield of 92%. The resulting monomer was copolymerized with acrylated epoxidized soybean oil to form a bio-based thermoset resin. It was shown that this bio-based resin had lower viscosity and curing temperature and higher polymerization rates, while the combination of the rigid isosorbide methacrylate and flexible soybean oil derivative resulted in enhanced mechanical and thermal properties. Badia and co-workers synthesized a mixture of mono- and bi-functional isosorbide methacrylate monomers by reacting isosorbide with methacrylic anhydride with a yield of 56% being obtained.^472,474^ The resulting mixture contained an 8:2 ratio of mono- to bi-functional monomer, from which the mono-functional methacrylate was separated *via* column chromatography (35% yield). In a final example, Howell and co-workers synthesized diphenylphosphato acrylates from isosorbide in a two-step reaction (Figure 31).^473^ Specifically, isosorbide was reacted with diphenylchlorophosphate and triethylamine at 0 °C, forming two isomers which were separated *via* column chromatography. Each isomer was then treated with acryloyl chloride to form the corresponding acrylate monomers. The endo- and exo-acrylate monomers were obtained in high yields of 98% and 93%, respectively.

##### 3.1.4.3 From Lactones

**Figure 32:**
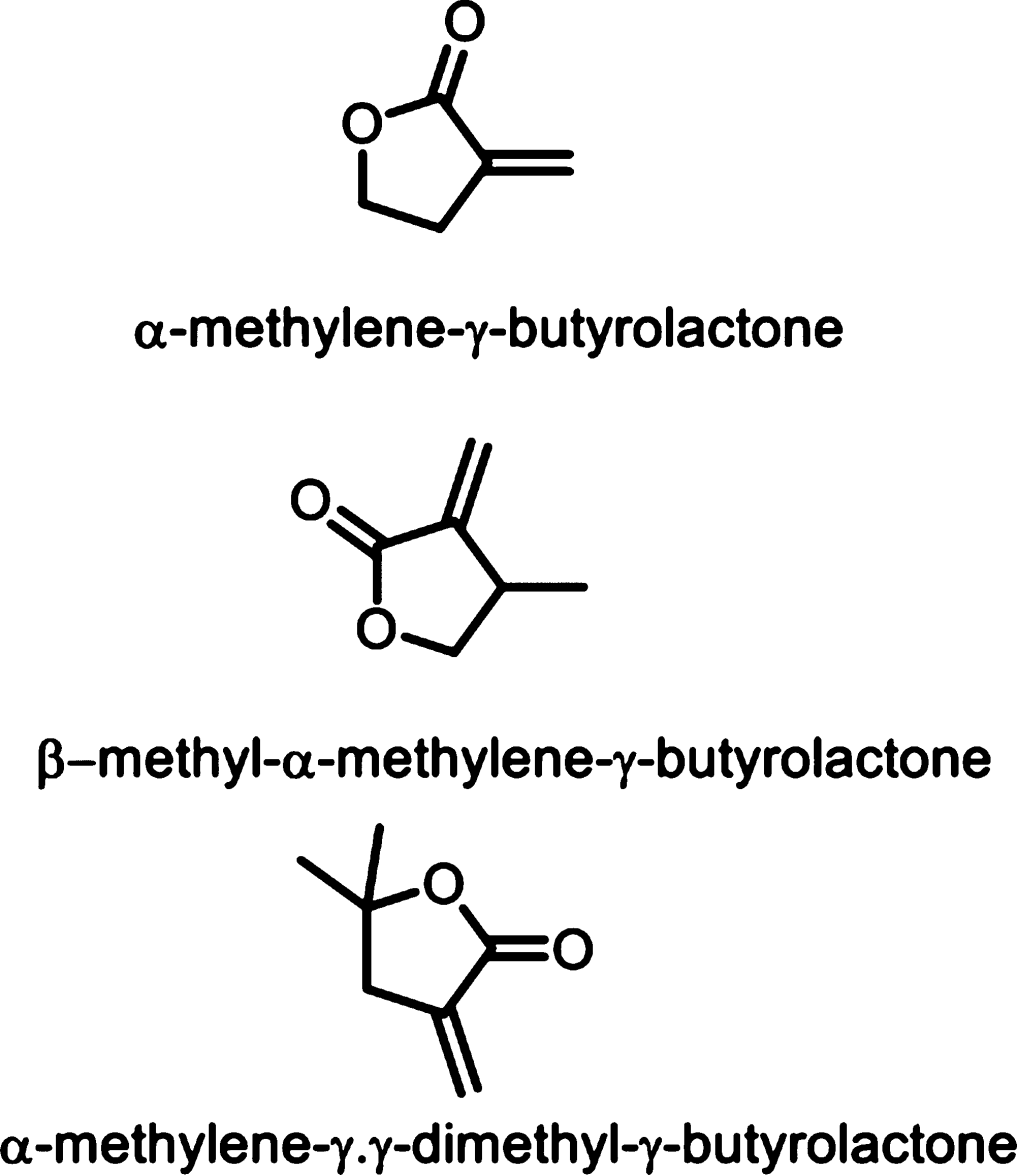
Butyrolactone derivatives also known as cyclic acrylates.

Lactones are cyclic esters that can be polymerized *via* ring-opening polymerization (cf. section 4.1). Some lactones possess a vinyl group and can be regarded as cyclic acrylates (Figure 32). An example is α-methylene-γ-butyrolactones (MBL), which is an important bio-source found in tulips, and is represented in about 10% of known natural products.^476−479^ Such a wide use originates from its biological, cytotoxic anti-cancer and antimicrobial properties.^478,480,481^ MBL is a more reactive cyclic analogue of methyl methacrylate, with the increased reactivity attributed to the relief of ring strain driving the polymerization reaction.^482^ Thus, MBL is an acrylate which can be polymerized *via* free radical polymerization, as illustrated by the formation of a homopolymer with a high *T*_g_ of 195 °C.^482^ As well as MBL, other lactones have been converted into acrylate monomers.

While MBL can be obtained naturally from tulips, this is an expensive process and so more viable sustainable routes have been investigated.^483^ One of these uses itaconic acid as the starting material to form a β-monomethyl itaconate ester, derived from an itaconic anhydride intermediate, which can be reduced to the alcohol using LiBH_4_.^483,484^ The latter then undergoes an acid catalyzed cyclization to form the 5-membered lactone (49% yield from β-monomethyl itaconate).^483,485^

**Figure 33:**
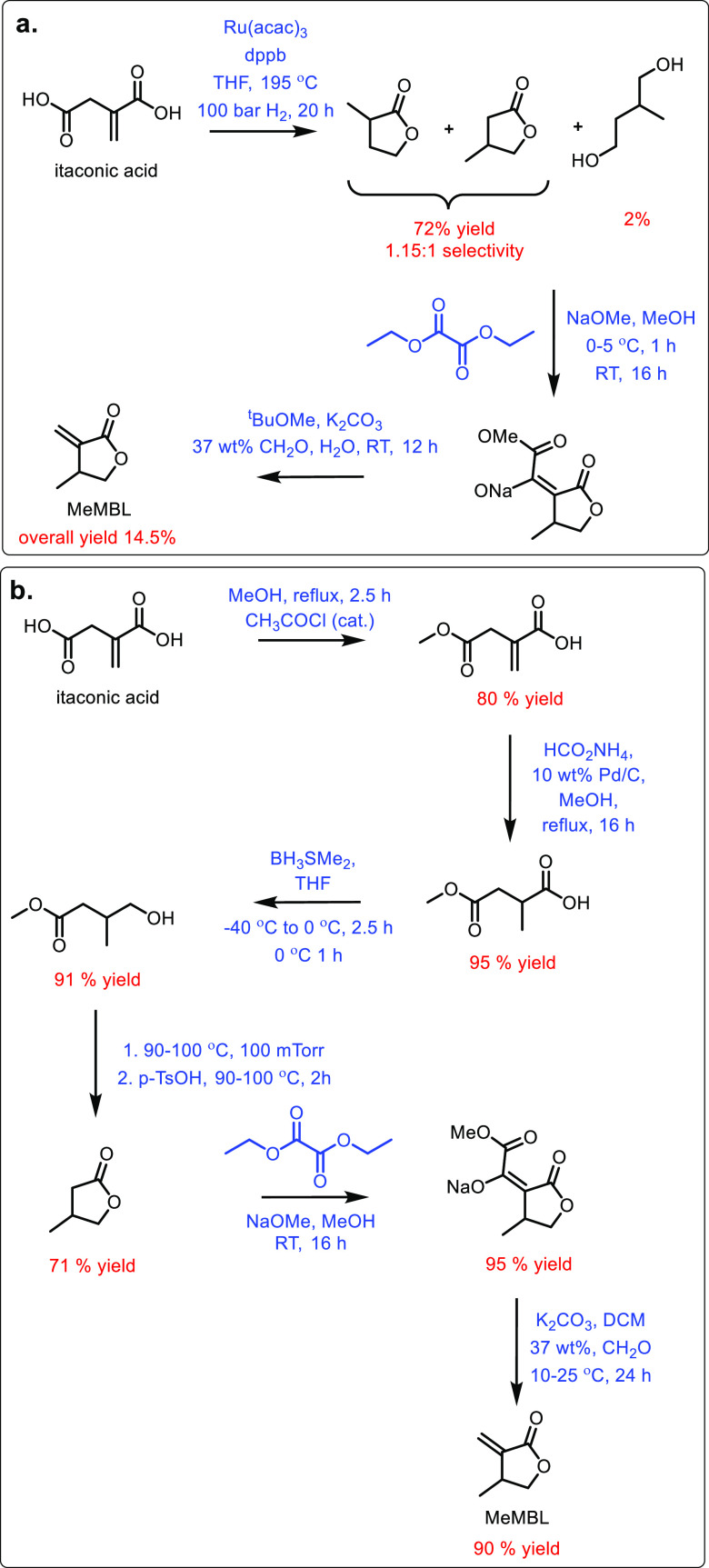
Two routes to form β-methyl-α-methylene-γ-butyrolactone (MMBL).

Two synthetic routes to β-methyl-α-methylene-γ-butyrolactone (MMBL) from the sugar-based itaconic acid were devised by Gowda and co-workers (Figure 33).^486^ The first route consisted of 3 steps (Figure 33a), with the first stage being a reduction of itaconic acid with 100 bar H_2_ at 195 °C using a ruthenium(III) acetylacetonate catalyst and diphosphine ligand. A pair of isomers and a side product were produced with the ratio of the desired to undesired isomer being 1:1.15. The mixture of isomers was reacted with diethyl oxalate and sodium methoxide before the resulting sodium salt was reacted with potassium carbonate and aqueous formaldehyde in *tert*-butylmethyl ether to give the desired product with an overall yield of 15%.^486−488^ The second, six-step, route was designed to be greener and to improve the overall yield of the synthesis (Figure 33b). Here, the first step was a partial esterification of itaconic acid with methanol using acetyl chloride as a catalyst to form a monomethyl ester.^486,489,490^ This was followed by a catalytic hydrogen transfer reduction with a Pd/C catalyst and ammonium formate as the hydrogen source.^486,491,492^ Then, the remaining carboxylic acid was reduced to the primary alcohol using BH_3_.SMe_2_ before a distillation containing a catalytic amount of *p*TsOH. It is noteworthy that this step selectively gives the desired isomer, which the reduction in the former synthetic route failed to achieve. The second and third steps from the first synthesis were then repeated to form the desired product with an increased overall yield of 53%. While this second route, despite being longer, is a significant improvement on the first, neither procedures can be considered highly sustainable, primarily due to the nature of the used chemicals. Nonetheless, the resulting monomer was successfully polymerized *via* organocatalytic conjugate addition polymerization, demonstrating its potential use in polymer science.^486,493,494^

Trotta and co-workers derived a single-step synthesis of another lactone, α-methylene-γ,γ-dimethyl-γ-butyrolactone (Me_2_MBL), which was successfully synthesized from β-monomethyl itaconate through a single-step reaction with the Grignard reagent MeMgCl, obtaining yields up to 55%.^485^ Another single step synthesis using the same starting material was derived using NaBH_4_ rather than the Grignard reagent, to form MBL in an optimized yield of 56%. The monomers were successfully converted into polymers with a range of molecular weights and narrow dispersity. Homopolymers of MBL and co-polymers containing MBL and MMA have also been prepared *via* Cu-mediated controlled radical polymerization techniques, obtaining polymers with molecular weights ranging from 31 800 to 62 400 g mol^–1^.^476,495^ Zhang *et al*. polymerized MBL using Al(C_6_F_5_)_3_ catalysts and obtained a 91% yield and a molecular weight of 44 800 g mol^–1^. When MMBL was polymerized using the same catalyst, the reaction proceeded to full conversion obtaining a 192 000 g mol^–1^ polymer.^479^

##### 3.1.4.4 From Levoglucosenone

Levoglucosenone is a sugar enone with a cyclic structure containing a double bond and can be easily modified.^496^ It is a valuable chiral molecule that is obtained from cellulose *via* flash pyrolysis.^497,498^ An advantage of using levoglucosenone over other bio-sources is that it can be produced from the pyrolysis of cellulose-containing industrial materials, for example waste paper.^499^ It is an important chiral precursor to many compounds including natural products, anticancer drugs, and green solvents. The production of levoglucosenone shows increased selectivity when an acid catalyst, such as phosphoric acid, solid superacids or zeolites, is used in the pyrolysis process.^499−502^ Cyrene is the reduced form of levoglucosenone and has the potential to be used as a green solvent.^497,503,504^

**Figure 34:**
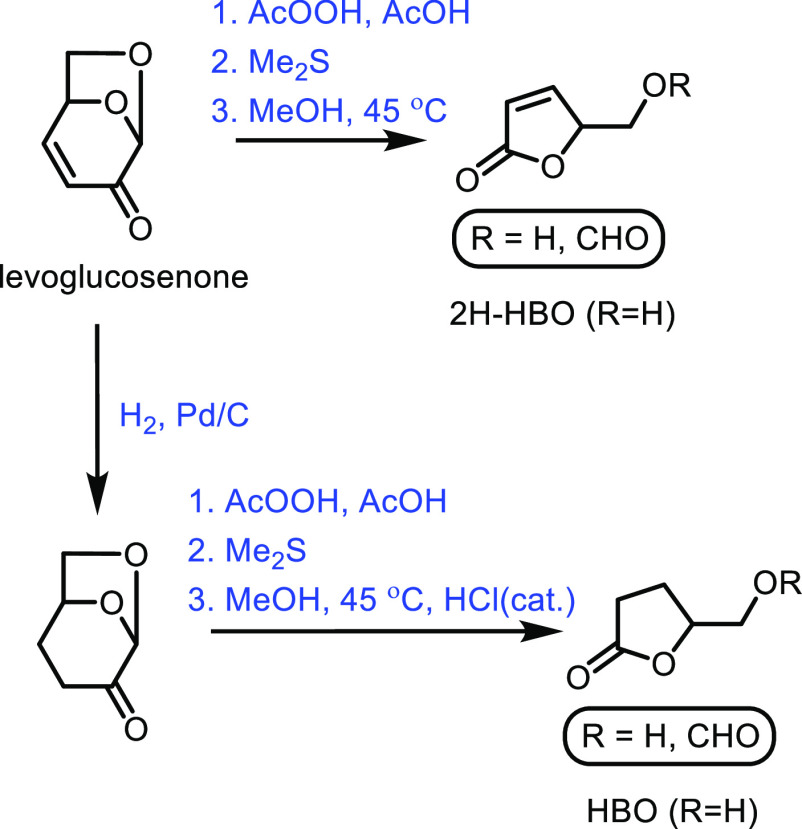
Synthesis of HBO and 2H-HBO, which were intermediates of the synthesis of acrylic monomers from levoglucosenone.

**Figure 35:**
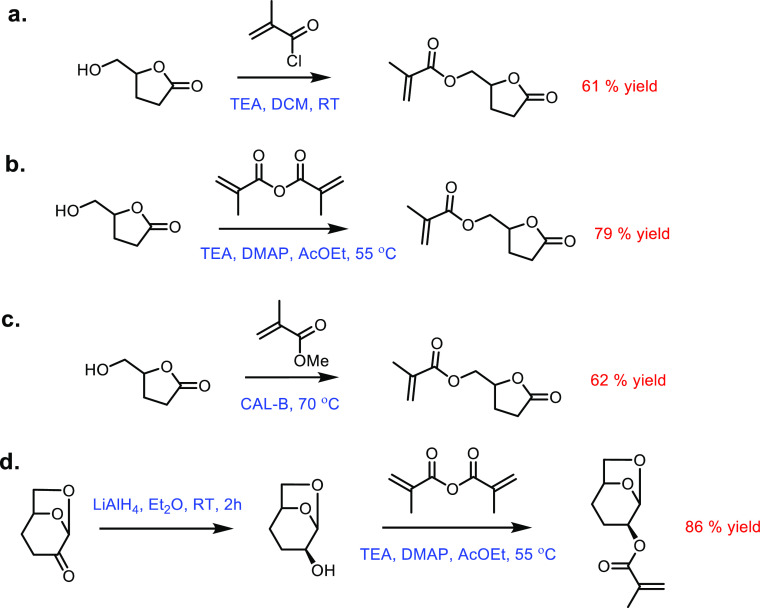
Different routes to form methacrylates from 2H-HBO and Cyrene.

The Baeyer–Villiger oxidation of levoglucosenone and Cyrene results into the formation of two lactones: 5-hydroxy-2-penten-4-olide (HBO) and dihydro-5-hydroxyl furan-2-one (2H-HBO) (Figure 34), which can then be used for acrylate monomer synthesis.^365,505^ The products from the above synthesis, HBO and 2H-HBO, both contain hydroxyl functional groups. As for previous examples, including those derived from terpenes and isosorbide, these hydroxyl groups can be readily converted into acrylate or methacrylate groups (Figure 35). The two main methods for the synthesis were again the reactions of the feedstock with either methacryloyl chloride or methacrylic anyhydride. Specifically, Zamzow *et al*. synthesized 2H-HBO methacrylate in 61% yield by reacting 2H-HBO with methacryloyl chloride (Figure 35a),^506^ while Ray and co-workers prepared the same monomer achieving a yield of 79% using methacrylic anhydride as a substitute for the more hazardous methacryloyl chloride (Figure 35b).^496^ Again, free radical polymerizations of the resulting monomer was successfully demonstrated. In another method, Allais and co-workers developed the synthesis of 2H-HBO methacrylate from 2H-HBO, which in turn can be derived from the levoglucosenone.^507^ Levoglucosenone was first converted to HBO using the lipase CAL-B and hydrogen peroxide, before the resulting product underwent hydrogenation into 2H-HBO. The conversion into the methacrylate monomer was carried out by the transesterification reaction of the resulting 2H-HBO with methyl methacrylate, using a catalytic amount of CAL-B, resulting in a yield of 62% (Figure 35c). The monomer was successfully polymerized, forming both homopolymers and co-polymers with methacrylamides. Previously, Warwel *et al*. showed that also an enzyme, Novozym-435, from the lipase *Candida antarctica*, could be used to catalyze the transesterification of methacrylates.^398^ A final approach to levoglucosenone-derived acrylics by-passed the synthesis of 2H-HBO from Cyrene and instead focused on the direct synthesis of methacrylated Cyrene *via* a two-step reaction (Figure 35d).^508^ The first step was the high yielding (i.e., 95%) reduction of the ketone functionality with LiAlH_4_, followed by the addition of methacrylic anhydride in the presence of a base to form the monomer with 86% yield. The resulting monomer was successfully polymerized in bulk, emulsion and solution.

#### 3.1.5 Aromatic (Meth)acrylates

**Figure 36:**
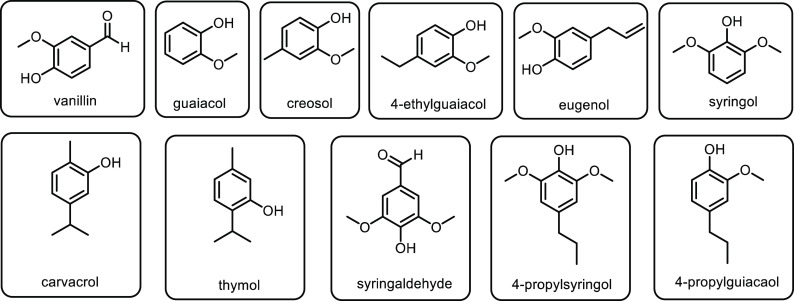
Structures of different platform chemicals derived from lignin.

Due to aromatic groups offering high stability, rigidity, and hydrophobicity for polymer chains and being predominantly petroleum based, interest in renewable aromatic polymers has greatly increased in recent years.^509^ The main source of aromatic biomass comes from lignin, representing 15-30% of lignocellulosic biomass.^365,510^ While lignin is difficult to process, (meth)acrylate monomers can be conveniently formed by reacting hydroxyl-containing bio-aromatic compounds derived from lignin.^22,365^ These monomers can then be polymerized to form high *T*_g_ polymers, which are of interest as potential styrene mimics. However, it is noteworthy that as these structures differ significantly from the structure of styrene, their ability to act as a direct replacement for styrene is limited. The structures of the key aromatic platform chemicals derived from lignin and used to synthesize bio-based acrylics are shown in Figure 36 and include vanillin, guaiacol, syringol and eugenol.^365,511^

**Figure 37:**
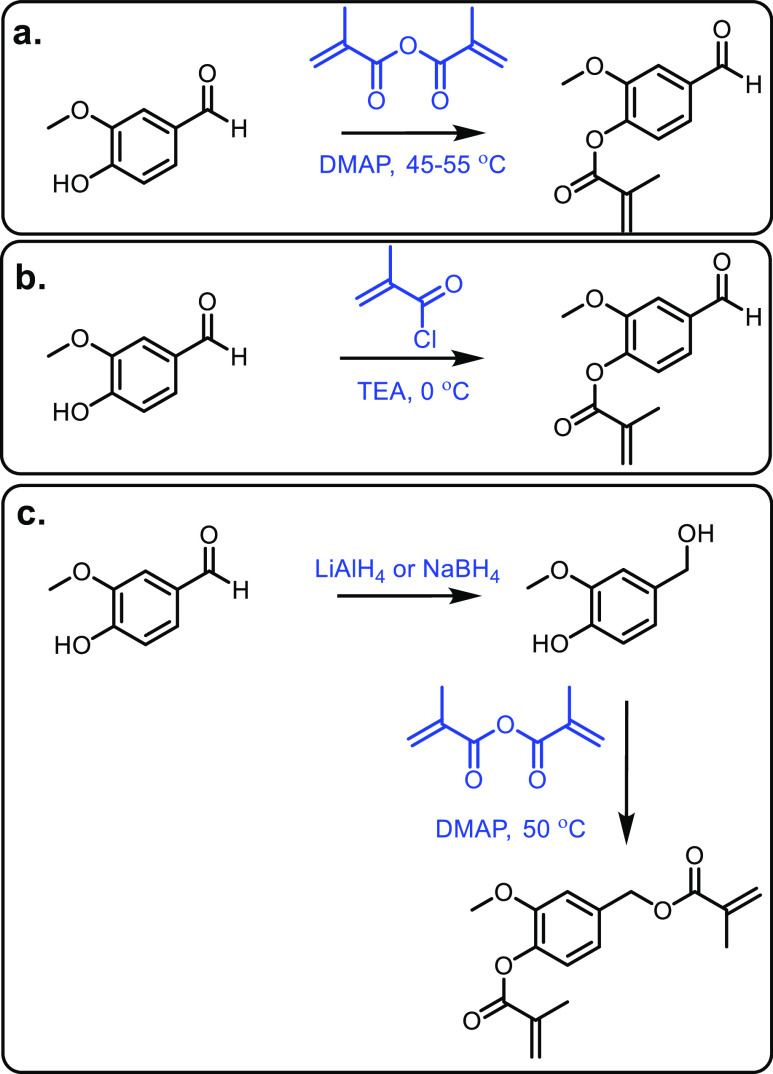
Synthetic routes to vanillin methacrylate by using methacrylic anhydride (a) and methacryloyl chloride (b). The synthesis of a bifunctional methacrylate is also shown (c).

One of the most widely used platform chemicals derived from lignin is vanillin. Vanillin can be formed from lignin by many different processes including the pulping of kraft lignin, lignin depolymerization, bisulfitation of lignin and lignin alkaline oxidation.^510,512−514^ Other phenolic compounds, such as guaiacol, syringol and their derivatives, can be derived from lignin through similar processing.^515,516^ Once these key feedstock chemicals have been derived from lignin, many examples have been reported for their transformation into methacrylate or acrylate-containing monomers. Many of these monomers have then been polymerized in an effort to replicate the properties of the non-bio-based alternatives (e.g., polystyrene). Two main methods have been reported for the synthesis of bio-based (meth)acrylates from lignin derivatives. Of reported examples, the methacrylating agent is either methacrylic anhydride or methacryloyl chloride, while the acylates were formed with acryloyl chloride. These two synthetic methods, as well as the synthesis of a bi-functional monomer, are illustrated in Figure 37 for vanillin and are more broadly applicable to other lignin-based platform chemicals.

Several different compounds originating from lignin have been converted into (meth)acrylates and then polymerized. In 2012, Wool and co-workers synthesized methacrylated vanillin by reacting vanillin with methacrylic anhydride with a DMAP catalyst at 50 °C for at least 24 h.^517^ This bio-monomer was then cured with a glycerol dimethacrylate cross-linker to form a thermoset resin. A glass transition temperature of 155 °C was recorded, which is comparable with commercially available non-bio-based vinyl ester resins. Holmberg and co-workers synthesized vanillin methacrylate using the same reaction conditions and utilized it in a co-polymerization with lauryl methacrylate.^518^ In addition to vanillin methacrylate, Epps and co-workers further synthesized guaiacol methacrylate, creosol methacrylate and 4-ethylguaiacol methacrylate *via* the same method, before the kinetics and reactivity ratios of their polymerizations were screened.^519^ Guaiacol methacrylate, creosol methacrylate, 4-ethylguaiacol methacrylate have also been obtained by reacting the corresponding lignin derived bio-precursors with methacrylic anhydride.^520^ The properties of the resulting homopolymers were also investigated, showing slightly higher *T*_g_ polymers were observed for poly(vanillin methacrylate) and poly(cresol methacrylate) (132–139 °C) compared to poly(guaiacol methacrylate) and poly(4-ethylguaiacol methacrylate) (116–120 °C). In a separate study, similar reactions were also conducted for the synthesis of methacrylated guaiacol and methacrylated eugenol.^521^ The homopolymers of methacrylated guaiacol and methacrylated eugenol had *T*_g_ values of 92 and 103 °C, respectively, and when blended with standard ester resins, the glass transition temperatures were comparable to styrene-ester resin blends. Syringyl methacrylate was also synthesized, although with a very poor yield of 7 wt %, albeit >99% in purity.^522^ Unlike the previous lignin-derived compounds, syringyl methacrylate, synthesized from syringol, is classified as originating from hardwood lignin. The resulting monomer has a remarkably high *T*_g_ for amorphous, linear, aliphatic polymers (i.e., 185–205 °C depending on molecular weight) and it has the potential to increase the *T*_g_ and mechanical resistance of copolymers, with only small amounts added.

Apart from monomers, a bi-functional vanillin methacryate has also been synthesized, by first carrying out a reduction of the aldehyde, before the reaction with methacrylic anhydride.^523^ The reduction can be carried out using reducing agents such as LiAlH_4_ or NaBH_4_, before the resulting vanillyl alcohol can undergo a reaction with methacrylic anhydride with DMAP at 50 °C for 18 h. The bifunctional methacrylated vanillyl alcohol was effectively used as a cross-linker in a bulk polymerization, with the resulting resin reported to show improved mechanical properties compared to the resin formed with the monofunctional vanillin methacrylate.

Aromatic chemicals derived from plant oils, such as thymol, can also be used to synthesize acrylic monomers to investigate their use as potential as styrene replacements.^524^ Due to these monomers sharing the same aromatic nature of styrene, there is potential to replace petroleum derived styrene with these bio-based monomers. Vergara *et al*. synthesized three monomers derived from carvacrol (oregano), thymol (thyme) and menthol (mint). The monomers derived from carvacrol and thymol were aromatic, while the monomer from menthol has a non-aromatic cyclic structure. The three monomers were synthesized by reacting the bio-source with methacrylic anhydride with a DMAP catalyst at room temperature for 24 h. Yields of 78%, 77% and 73% were obtained for caracryl methacrylate, thymyl methacrylate and menthyl methacrylate, respectively. The resulting monomers were shown to have comparable or enhanced performance compared to styrene in diglycidyl ether of bisphenol A-based vinyl esters, for use in thermoset resins.

The second synthetic route to produce aromatic bio-based monomers from lignin is through a reaction with methacryloyl chloride (or acryloyl chloride). Abdelaty and Kuckling synthesized vanillin acrylate in 85% yield by reacting vanillin with acryloyl chloride.^525^ A copolymer was then formed with NIPAAm through free radical polymerization, while post-polymerization modification were carried out by reacting the aldehyde on the vanillin moieties to form imines. Zhou and co-workers synthesized the acrylates and methacrylates of vanillin and syringaldehyde by reacting them with acryloyl chloride and methacryloyl chloride, respectively.^526^ Yields of 65%, 60%, 63% and 50% were achieved for vanillin methacrylate, vanillin acrylate, syringaldehyde methacrylate and syringaldehyde acrylate, respectively. The resulting monomers were successfully used in a free radical polymerization with the syringaldehyde based polymers having a significantly greater *T*_g_ (> 170 °C) than the vanillin-based (<110 °C) and petroleum-based polymers (<110 °C). In a final example, 4-propylsyringol and 4-propylguaiacol were reacted with acryloyl chloride to synthesize 4-propylsyringol acrylate and 4-propylguaiacol acrylate, respectively, with high purity and yields.^527^ Controlled radical polymerization of these monomers was investigated and high molecular weights and low dispersity values (<1.44) were obtained. A low dispersity triblock, containing one of the bio-based monomers and *n*-butyl acrylate was also prepared, which showed promising thermal and mechanical properties.

**Figure 38:**
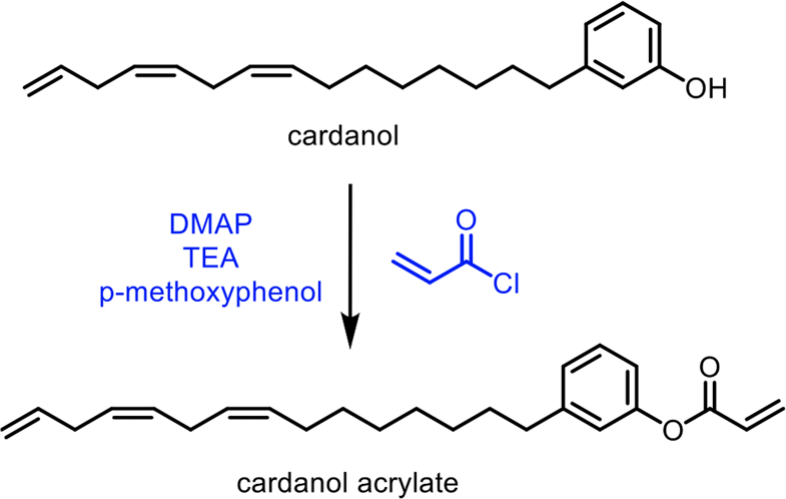
Synthesis of cardanol acrylate by coupling with acryloyl chloride.

Other aromatic platform chemicals, which are not derived from lignin, can be used to synthesis acrylate monomers. One example is piperonyl alcohol, which can be derived from piperine that is sourced from black pepper. There are several reported routes for the synthesis of piperonyl alcohol from piperine *via* the intermediate piperonal.^528^ The first step to form the aldehyde intermediate involved either an oxidation with KMnO_4_/THF or an ozonolysis with O_3_/H_2_O. The subsequent reduction to the alcohol product was carried out with either NaBH_4_ or Pt/H_2_. Once the alcohol was formed, the piperonyl methacrylate monomer was formed by a reaction with methacrylic anhydride, with a yield of 89% being obtained. The monomer was then investigated for potential use in water-based pressure sensitive adhesives. Cardanol is an aromatic lipid that is derived from cashew nutshell liquid. Cardanol contains a phenolic group that was reacted with acryloyl chloride at 40 °C for 8 h, to form cardanyl acrylates with differing R groups (Figure 38).^529^ However, no yields were reported. Ladmiral and co-workers took a different approach to the synthesis of cardanol methacrylate, where the first step was the epoxidation of cardanol with epichlorohydrin and tetrabutylammonium chloride.^530^ The epoxy product was obtained with a yield of 92% and was then subsequently reacted with methacrylic acid andtriphenylphosphine at 80 °C for 20 h. The product was formed with an 87% yield.

The synthesis of acrylates and methacrylates has been vastly explored and a wide range of biomass has been used. Many of these routes offer large potential as alternative routes to synthesize important monomers without the use of crude oil. For commodity polymers, such as acrylic acid and methacrylic acid, there are several different feedstocks that can be used and all offer advantages and disadvantages, for example, glycerol is a by-product from biodiesel production and is, therefore, readily available. However, when crude glycerol was used lower yields were obtained and thus, expensive purification methods were required prior to the monomer synthesis being carried out.^173,259^ For many of the processes that have been developed to synthesize acrylic acid and methacrylic acid from biomass, complex catalysts and high operating temperatures are required and this significantly limits the sustainability of these processes. Furthermore, many of these processes are plagued by low yields, with the most significant example being the synthesis of methacrylic acid from isobutane, when low conversions resulted in yields <10%.

Further to the synthesis of commonly used acrylic monomers, more complex monomer structures have been synthesized by using the unique structures of natural compounds. These include monomers synthesized from vegetable oils, terpenes, isosorbide and lignin. In contrast to the procedures for acrylic acid and methacrylic acid, low temperatures are used in these procedures. However, these methods consist of long multi-step organic syntheses, thus hindering yields, and often involves the use of toxic reagents. Due to the feedstock used, the structures of these monomers differ significantly from commonly used conventional monomers and, therefore, they do not act as direct replacements. However, the complex structures of these monomers offer unique properties, for example, the terpene and isosorbide derived monomers offer improved rigidity, due to their cyclic structures. While these developments in the synthesis of these monomers from biomass offers a great deal of potential, problems with high temperatures, the use of complex catalysts and poor yields and selectivity must be overcome for these methods to become viable to replace the petrochemical monomer synthesis.

### 3.2 (Meth)acrylamides

**Figure 39:**
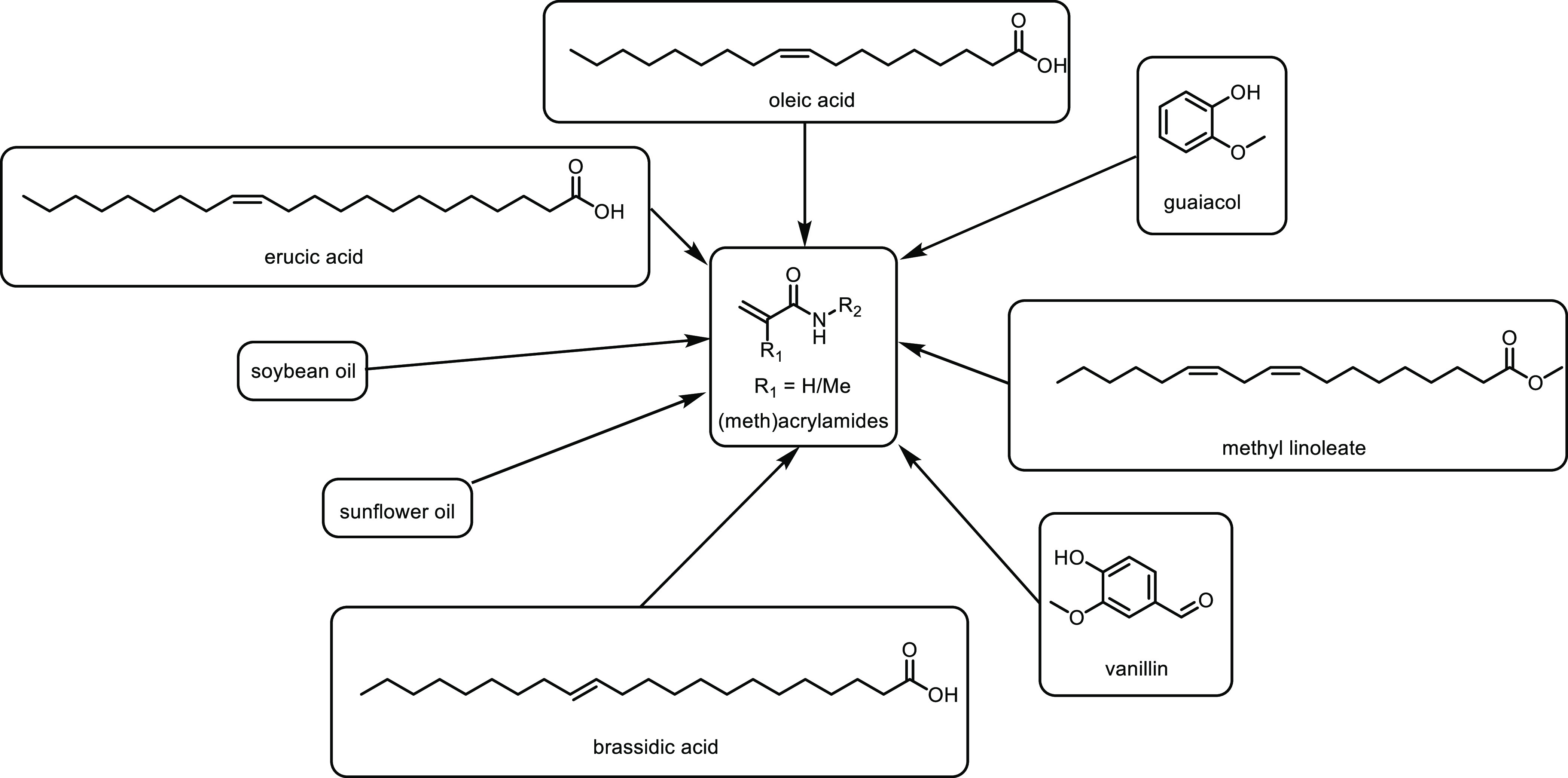
Structures of the different bio-based feedstock for the production of (meth)acrylamides.

Acrylamide is a toxic water-soluble monomer that is derived from the hydration of acrylonitrile. While poly(acrylamide)s have many applications, including as flocculants, poly(*N*-isopropylacrylamide) (PNIPAAm) is one of the most widely studied and is well known for its thermoresponsive behavior. Indeed, hydrogels based on PNIPAAm are used in many fields including astronomy, energy and in the biomedical field, especially in drug delivery and tissue engineering. Like for acrylic monomers, acrylamide monomers can be produced from biomass. However, examples of bio-based acrylamides have been much less reported and only a handful of synthetic procedures have been highlighted. Whereas when considering (meth)acrylates, the most used monomers (acrylic acid, methacrylic acid, methyl acrylate and methyl methacrylate) have had their potential synthetic routes from biomass investigated, there has been very little research into the synthesis of acrylamide or NIPAAm from biomass. Thus, in this section, the focus is on those examples of forming bio-based acrylamides from vegetable oils, either through the functionalization of carbon–carbon double bonds or the acid functionality, and the lignin derived guaiacol and vanillin. The structures of the resulting monomers are very different from the most commonly used acrylamides and so do not act as a direct replacement for conventionally derived acrylamides. The structures of some of the bio-based feedstock to produce (meth)acrylamides are displayed in Figure 39. Similar to their acrylate counterparts, (meth)acrylamide processes are not entirely bio-based as coupling agents such as acryloyl chloride are used.

**Figure 40:**
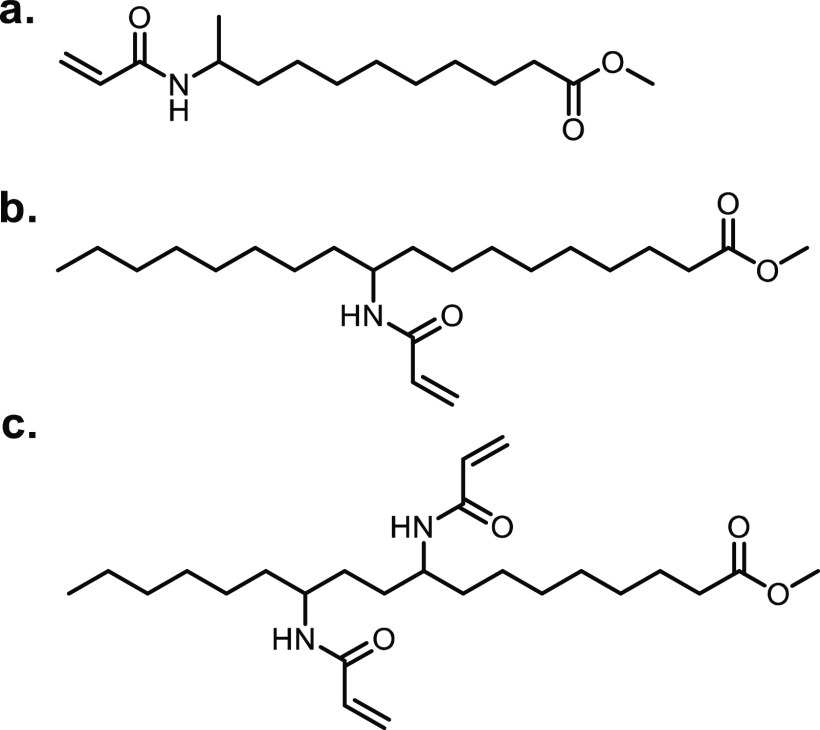
Examples of acrylamides derived from methyl 10-undecanoate (a), methyl oleate (b) and methyl linoleate (c).

Acrylamidation of fatty acids and fatty esters have been reported as one synthetic method to form bio-based acrylamides.^388^ As with the production of acrylates from vegetable oils, either the carbon–carbon double bonds or the carboxylic group can be functionalized.^388^ When considering reacting the alkene bond, the double bond was protonated with concentrated sulfuric acid, forming a carbocation.^388,531^ The carbocation can then react with a nucleophilic nitrile, which forms an amine upon hydrolysis. Some examples of acrylamides derived *via* this method are displayed in Figure 40. The first example of this applied to fatty acids was reported by Roe and Swern in 1953 where the addition of different nitriles to oleic acid was investigated.^532^ When acrylonitrile was used, an acrylamide was formed with 89% yield. Sinnreich *et al*. investigated the same reaction for a wider range of fatty acids including erucic acid and brassidinic acid.^533^ Eren and co-workers synthesized several acrylamides including methyl oleate acrylamide, methyl linoleate acrylamide, sunflower oil acrylamide and soybean oil acrylamide.^534^ The yields reported for the linoleate acrylamide, sunflower oil acrylamide and soybean oil acrylamide were 60%, 50% and 45%, respectively.

**Figure 41:**
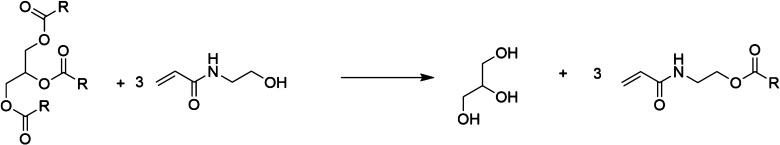
Transesterification of fatty esters to form acrylamide monomers.

Acrylamides can also be formed through acrylamidation of the carboxylic functionality of fatty acids, with an example illustrated in Figure 41. Tarnavchyk *et al*. reported a transesterification of triglycerides from soybean oil with *N*-(hydroxyethyl)acrylamide in the presence of sodium hydroxide at room temperature.^535^ The resulting acrylamide monomer was formed in a 90% yield, and was subjected to free radical polymerization. Given the success in the monomer synthesis, it was expanded to include other oils, including sunflower oil, linseed oil and olive oil. Using THF as the solvent and sodium hydroxide as the catalyst allowed for yields of between 93% and 96% to be achieved.^536,537^

**Figure 42:**
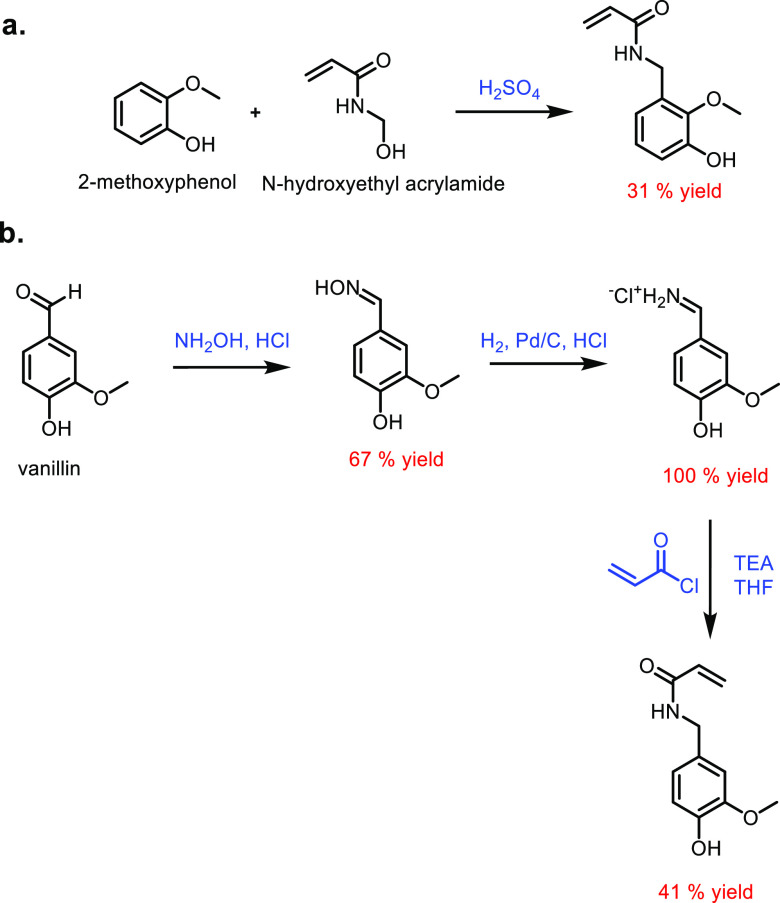
Two different routes to form aromatic acrylamide monomers *via* a single-step Friedel–Crafts reaction (a) or a multi-step reaction (b).

A final example of producing a bio-based acrylamide is a method using the lignin-derived chemicals guaiacol and vanillin, for which two approaches were devised.^22,538^ The first approach was a single-step synthesis using guaiacol as the starting material (Figure 42a), and proceeded *via* a Friedel–Crafts alkylation with *N*-hydroxymethylacrylamide. However, low yields were reported for this synthetic route. When a strongly acidic cationic exchange resin was used as the catalyst, a yield of 25% was obtained after 4 days. When the catalyst was changed to sulfuric acid, the yield increased slightly to 31% with the same reaction time. It was shown that for both catalysts further increasing the reaction time did not lead to any improvement in the yield. The second approach was a three-step synthetic route starting with vanillin (Figure 42b). The first step was the formation of an oxime from vanillin through a reaction with hydroxylamine in the presence of hydrochloric acid, and a yield of 67% was reported. This was followed by a reduction with a Pd/C catalyst that proceeded to completion. The final step to form the acrylamide was a reaction with acryloyl chloride and a yield of 41% was obtained for the final step. The overall yield of the three-step synthesis was recorded as 27%.

While the methods highlighted above show the potential to produce acrylamide monomers from biomass, the scope has thus far been very limited. The sources of biomass used in these methods results in monomers with structures that significantly differ from conventional used acrylamides, such as NIPAAm, and this hinders their potential applications on an industrial scale. Furthermore, several of the limited methods that have been investigated are plagued by low yields, require multiple steps or use hazardous reagents and catalysts. Therefore, improvements into these methods have to be explored, as well as the potential to increase the scope of biomass that can be used for the production of acrylamide monomers.

## 4 Cyclic Monomers

### 4.1 Lactones

**Figure 43:**
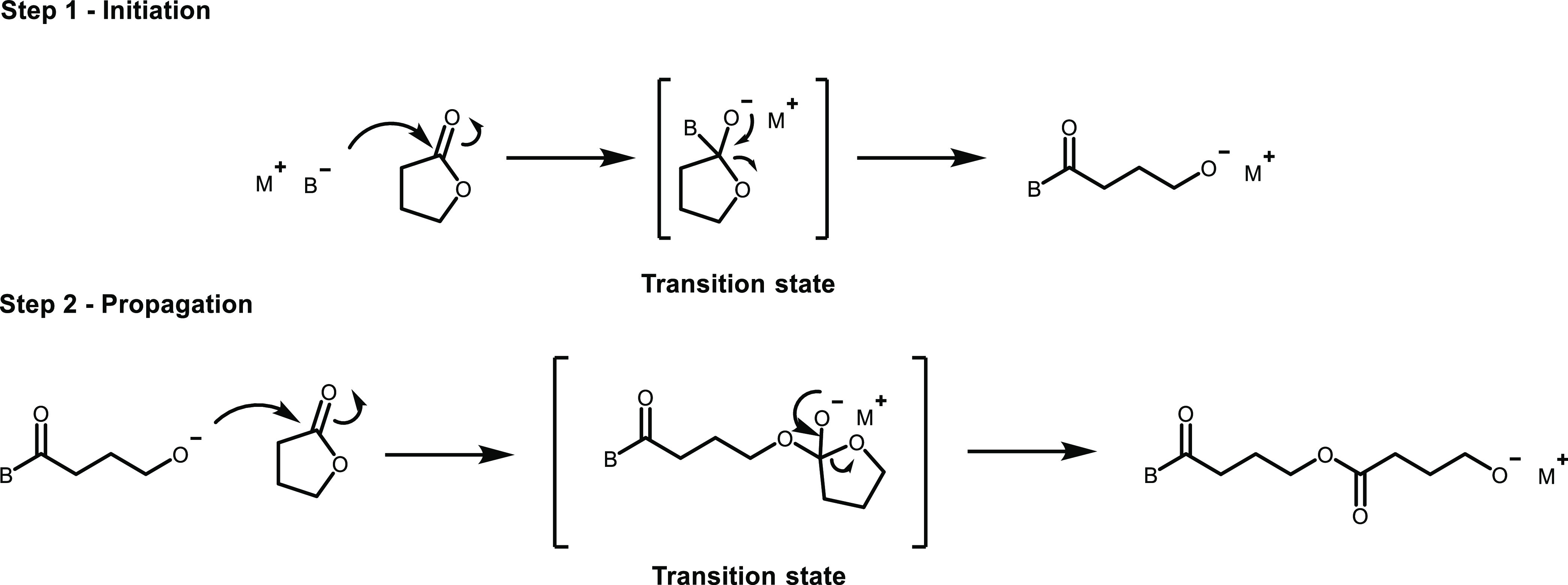
Mechanism for the ring-opening polymerization of lactones.

Lactones are cyclic esters that can be utilized to synthesize polyesters *via* ring-opening polymerization (ROP), of which the mechanism can be seen in Figure 43. The French chemist Théophile-Jules Pelouze first coined the term “lactone” when he derived the cyclic structure from lactic acid in 1844. Poly(lactone)s have the added benefit of possessing a regular repeating ester linkage along their backbone causing them to be biodegradable under mild conditions.

**Figure 44:**
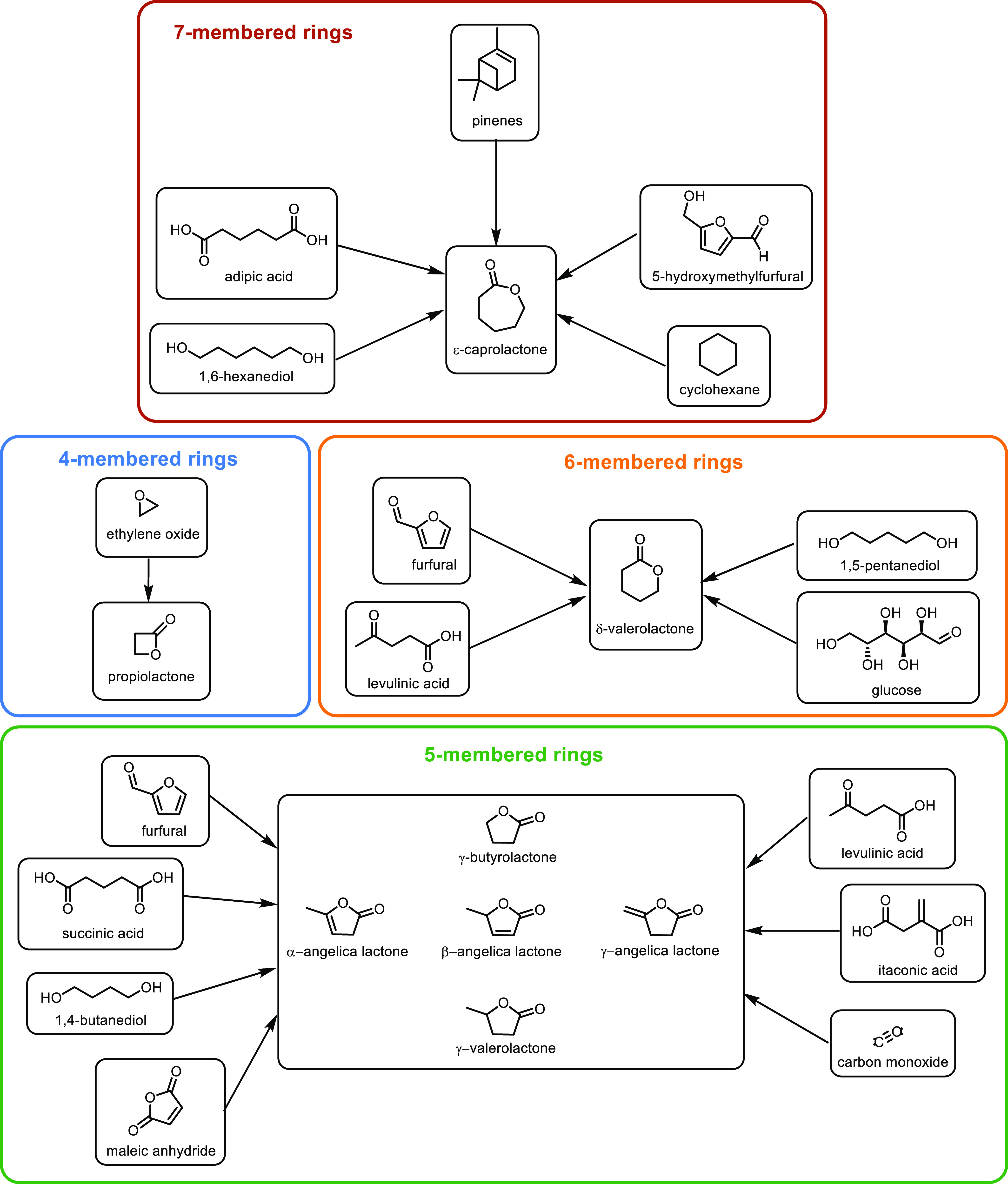
Sources of bio-based, sustainable lactone monomers.

This review is designed to discuss monomers that are derivable from sustainable sources, and hence is not intended to offer a comprehensive review of all aspects of lactone polymer chemistry. Carlotti and co-workers provided an excellent review in 2020 highlighting the wide variety of lactones available and the range of potential initiators that can be employed.^539^Figure 44 shows the general lactone structures that are covered here and their sustainably sourced starting materials. First, ε-caprolactone is discussed, followed by δ-valerolactone, 5-membered rings, then propiolactone, and finally some other lactone structures are described.

#### 4.1.1 7-Membered Lactones

The emergence in recent years of tissue engineering within the field of biomaterials has sparked something of a renaissance in interest in poly(caprolactone)s. This is because poly(caprolactone)s exhibit superior viscoelastic and rheological properties compared to their counterparts, and possess the status of an FDA approved chemical.^540^ Generally, the synthesis of ε-caprolactone is carried out by the Baeyer–Villiger oxidation of cyclohexanone; however, there are still opportunities to improve this well-developed synthetic route. For example, the oxidation step can be catalyzed by SiO_2_ nanorods, which are reusable, easily separable and metal-free.^541^ Furthermore, the oxidation can be carried out in ethyl acetate, which can be considered to be an environmentally friendly solvent. As well as chemical catalysis, biological-derived enzymes such as lipases have been shown to be extremely promising biocatalysts for the oxidation step, which remove the need for complex catalysts entirely.^542,543^

Increasingly, bioengineering has become a more important method for monomer synthesis in general, and the production of ε-caprolactone is no different. Bioengineering removes the need for metal catalysis and harmful solvents while relying on the enzymatic power of bacteria. One route to ε-caprolactone is *via* oxidation of cyclohexane by bacteria. Although cyclohexane is typically regarded as a petrochemical, it can be derived from sustainably sourced anisole with excellent selectivity.^544^ Nonetheless, recent examples of the bioconversion of cyclohexane into ε-caprolactone tend to suffer from low conversion.^545^ However, a recently bioengineered version of *Pseudomonas taiwanensis* was able to fully convert 5 mM cyclohexane into ε-caprolactone and 6-hydroxyhexanoic acid.^546^

Next to cyclohexane, 1,6-hexanediol can be considered as another sustainably sourced precursor for ε-caprolactone, as it can be derived from adipic acid and 5-hydroxymethylfurfural. 5-Hydroxymethylfurfural is a well-known renewable platform chemical, and adipic acid can be formed from the oxidation of fatty acids.^547^*Gluconobacter oxydans* has been shown to be able to oxidize 1,6-hexanediol into 6-hexanoic acid, which can then be chemically ring closed with a yield of around 73%.^548^ Chemical catalysis can also be used to convert 1,6-hexanediol into ε-caprolactone. For example, Heeres et al. carefully optimized a four-step route to ε-caprolactone with an overall selectivity of 95%.^549^ Silica supported copper is able to generate ε-caprolactone alongside 1-hexanol and 1,6-hexanediol,^550^ and Pt-loaded SnO_2_ can be used to lactonize 1,6-hexanediol with an 86% yield.^551^

**Figure 45:**
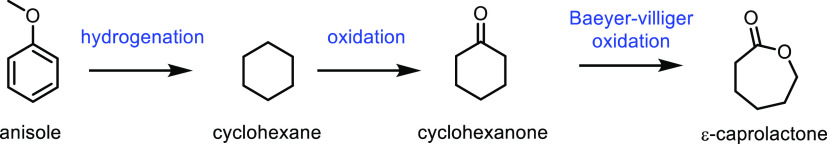
General scheme for the transformation of anisole to caprolactone.

Generally, the synthesis of ε-caprolactone is carried out by the Baeyer–Villiger oxidation of cyclohexanone, which in turn can be derived from bio-derived anisole *via* a hydrogenation and oxidation process (Figure 45).^552,553^ However, there are still opportunities to improve this well-developed synthetic route.

Lastly, ε-caprolactone can even be derived from pinenes; however, the only example of this synthetic route requires four steps, making use of ozone, AlCl_3_, a Ru catalyst, and mCPBA to give an overall moderate yield of 64%.^554^

#### 4.1.2 6-Membered Lactones

δ-Valerolactone is somewhat less recognized than ε-caprolactone; however, polymers synthesized from δ-valerolactone are versatile with good mechanical properties and degradability.^555^ As was seen with ε-caprolactone, synthesis routes to δ-valerolactone from platform chemicals tend to require multiple steps and this is currently a disadvantage for the industrial viability of biomass-derived lactone monomers.

δ-Valerolactone can be made from a variety of platform chemicals and sustainably derived chemicals, including furfural, levulinic acid and 1,5-pentanediol. The routes discussed have positive and negative aspects, but they are all interesting and worth considering. Furfural is a well-known platform chemical,^13^ and has been previously converted to δ-valerolactone by Tomishige and co-workers. They initially oxidized furfural to 2-furancarboxylic acid, which was then hydrogenated to form 5-hydroxyvaleric acid, followed by ring closure to δ-valerolactone. Unfortunately, the highest δ-valerolactone yield was only 7%; however, methyl 5-hydroxyvalerate was also produced with a 55% yield, which could subsequently ring closed to form δ-valerolactone. Furthermore, a platinum catalyst was required, diminishing the sustainability of the project. However, the catalyst could be regenerated by calcination.^556^

**Figure 46:**
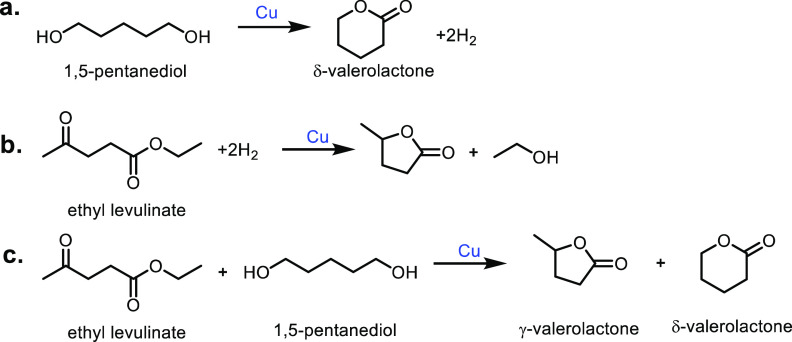
Synthesis protocols for valerolactones.

As an alternative to furfural, 1,5-pentanediol can also be used as a different sustainably sourced precursor for δ-valerolactone.^557^ A route with mild conditions and high yields (> 98%) was achieved by coupling the dehydrogenation reaction of 1,5-pentanediol with the hydrogenation reaction of ethyl levulinate (Figure 46B).^558^ This route also produces γ-valerolactone in high yield, but it is nonetheless an important route to sustainable 6-membered lactones. It should be noted that 1,5-pentanediol itself is not a platform chemical but can be derived from glutamic acid, which is an industrially scaled amino acid.

As well as furfural and 1,5-pentanediol, 6-membered lactones can be derived from levulinic acid. Levulinic acid is a promising platform chemical that can be synthesized on an industrial scale.^559^ The procedure to make the lactone in question, 4-ketovalerolactone, required a bromination step in chloroform, followed by ring closure with triethylamine. Although this route is relatively straightforward requiring just two steps to the lactone, the use of halogenated solvents and bromine detract from the sustainable aspect of the work and benefit from improvements.^560^

An enzymatic approach can also be taken to produce δ-valerolactone, as opposed to a purely chemical synthesis. For example, isotactic, biodegradable poly(lactone)s have been made from β-methyl-δ-valerolactone, which in turn were prepared from glucose using enoate reductase enzymes.^561^ Interestingly the isotactic polymers were amorphous like their atactic counterpart, and also had a similar softening temperature (221 K). The main benefit of taking an enzymatic approach is that they are generally much more specific than a chemical catalyst.

Although many of the routes here use well-known platform chemicals, they require several steps and often chemicals that are of environmental concerns.

#### 4.1.3 5-Membered Lactones

There are several different bio-based 5-membered lactones including γ-butyrolactone, isomers of angelica lactone and γ-valerolactone. There is a plethora of sustainable routes to 5-membered lactones, which is due to the large number of potential precursors including succinic acid, fumaric acid, and maleic anhydride. In this section, first γ-butyrolactone is discussed as it is the most simple 5-membered lactone, followed by γ-valerolactone and then the angelica lactones, before any anomalous lactones are discussed.

##### 4.1.3.1 γ-Butyrolactone

γ-Butyrolactone is of interest to the sustainable chemist because of the diverse range of bio-based feedstocks that it can be derived from. In fact, of all the lactone and lactam rings explored in this review, γ-butyrolactone has the widest range of potential bio-based starting materials. Nonetheless, γ-butyrolactones do have one major issue, which is the difficulty in polymerizing them *via* ring-opening polymerization. This is because γ-butyrolactone is a 5-membered ring and possesses relatively low ring strain, which does not bring about a sufficient change in enthalpy upon its ring-opening to offset the entropic cost of polymer formation. Moreover, extreme conditions such as 20 000 atm and 160 °C were shown to only be sufficient to produce oligomers.^28^ More recently, new techniques such as copolymerization with other lactones, and the use of powerful initiators (e.g. BuLi) has allowed the polymerization of γ-butyrolactone to yield polymers with molecular weights of over 10 kDa that are degradable.^562,563^

One of the more common feedstocks for γ-butyrolactone is succinic acid, which is another well-known platform chemical.^564^ The conversion of succinic acid to γ-butyrolactone requires a catalyst, which has been the focus of numerous investigations. Here, some of the more recently developed catalytic systems (post-2010) to obtain γ-butyrolactone from succinic acid are highlighted. An overview of these catalysts, conditions, selectivity, and γ-butyrolactone yield is presented in Table 12. The generally taken approach for the production of γ-butyrolactone from succinic acid is liquid phase hydrogenation. The best catalysts typically use Pd/Pt/Ru metals as they are the most efficient. Moreover, the catalytic metal is generally supported on a framework and significant efforts have been undertaken to improve the catalytic performance of these supported metals.

**Table 12:** Different Catalytic Systems for the Formation of γ-Butyrolactone from Succinic Acid

Catalyst	Solvent	Temperature (°C)	Pressure (bar)	Conversion (%)	Selectivity (%)	Yield (%)	Ref.
Pd/Al_2_O_3_	1,4-Dioxane	260	60	64	70	45	(567)
Pd/Al_2_O_3_	1,4-Dioxane	240	60	81	69	56	(568)
Pd/Al_2_O_3_	1,4-Dioxane	240	60	79	65	51	(569)
Pd/Al_2_O_3_	1,4-Dioxane	240	60	97	94	91	(570)
Pd/SiO_2_	EtOH + water	250	100	65	39	25	(571)
Pd/TiO_2_	Water	160	150	75	95	71	(572)
Pd-Re/TiO_2_	Water	160	150	> 50	95	48	(573)
Pd-Re/TiO_2_	1,4-Dioxane	140	80	> 99	56	56	(574)
Pd-Re/C	Water	240	80	92	nr	67	(575)
Pd-Cu/Al_2_O_3_	2-Isopropanol	190	70	72	94	68	(576)
Ru(acac)_3_-triphos^a^	Methanol	120	80	96	nr	76	(577)
Ru/SiO_2_	1,4-Dioxane	200	50	nr	nr	90	(578)
Ru/C	1,4-Dioxane	240	60	90	74	67	(579)
Ru/C	1,4-Dioxane	240	80	45	98	44	(580)
Ru/C	1,4-Dioxane	240	80	95	nr	20	(581)
Re-Cu/C	1,4-Dioxane	240	80	100	23	23	(582)
Re-Ru/C	1,4-Dioxane	140	80	64	75	48	(583)
Re/C	Water	240	80	34	93	32	(584)
Re/C	1,4-Dioxane	240	80	80	77	62	(585)
Mo/TiO_2_	Water	240	150	81	68	55	(586)
Ir/C	Water	240	150	72	88	63	(587)

a Triphos: 1,1,1-tris(diphenylphosphinomethyl)ethane.

The main problem with using succinic acid as a feedstock for γ-butyrolactone production is that of catalyst selectivity. Succinic acid can be transformed into other chemicals such as 1,4-butanediol, maleic anhydride, and succinic anhydride amongst others, and therefore reaction optimization is important.^565^ Hence, many of the reactions use high temperatures and high pressures, as well as less environmentally preferred solvents, all of which are disadvantageous in terms of the overall sustainability of γ-butyrolactone production.

Another well-known bio-based acid is fumaric acid, which can be hydrogenated to succinic acid and then subsequently converted into γ-butyrolactone; however, this is much less reported than the direct conversion of succinic acid. This has been performed recently with the use of a silica supported Pd–Re catalyst with a selectivity of 91% toward γ-butyrolactone, supported by the use of computer modelling.^566^

Furfural is a platform chemical that is derived from the hydrolysis and subsequent dehydration of xylan, which is a polysaccharide found in lignocellulosic biomass.^588^ In more recent times, the conversion of furfural into other useful chemicals, including γ-butyrolactone has begun to be explored.^589,590^ Generally, the first step to γ-butyrolactone from furfural is the conversion of furfural into furanone; however, other furfural derivatives can be used to make γ-butyrolactone. For example, a metal-free route to γ-butyrolactone was presented very recently, whereby derivatives of furfural such as tetrahydrofurfuryl alcohol were aerobically oxidized with mesoporous carbon nitride as a catalyst to yield γ-butyrolactone.^591^ Using this method, tetrahydrofurfuric acid was able to be converted into γ-butyrolactone with a yield of around 85%, under very mild conditions. The conversion of furanone to γ-butyrolactone requires catalysis, and several examples have been reported in the literature (see Table 13). As with succinic acid, the catalysts are required for hydrogenation, and Pt and Pd are typically the best metals for this. The examples below typically focus on mixing the metal catalyst with dopants, and how they are fixed to the solid support.

**Table 13:** Catalysts, Conditions and Yields for γ-Butyrolactone from Furfural/Furanone

Catalyst	Solvent	Temperature (°C)	Pressure (bar)	Conversion (%)	Selectivity (%)	Yield (%)	Ref.
Pd/SiO_2_	Methanol	80	35	100	77	77	(592)
Ni-Fe/SiO_2_	Methanol	80	35	100	90	90	(593)
Pt-Ni/SiO_2_	Methanol	80	3.5	100	78	78	(594)
Pt-Ni/SiO_2_	Methanol	80	35	100	76	76	(595)
Pt/Al_2_O_3_	Methanol	80	35	100	61	61	(595)
Pt-Ni/ZrO_2_	Methanol	80	35	100	51	51	(595)
Pt-Ni/TiO_2_	Methanol	80	35	100	59	59	(595)
Pt-Ni/TiO_2_-ZrO_2_	Methanol	80	35	100	55	55	(595)
Pt-Nb_5_Zr_5_/solid acid	1,4-Dioxane	140	50	100	97	97	(596)

1,4-butanediol can be derived from natural sugars such as dextrose, making this an important chemical for sustainably derived chemicals.^597^ Furthermore, the transformation of 1,4 butanediol into γ-butyrolactone produces minimal waste with the only side product being 2 equiv of hydrogen. In fact, the dehydrogenation of 1,4-butanediol can be used *in situ* to act as a hydrogen source for hydrogenation reactions.^598,599^ Interestingly, the reversible nature of the hydrogenation/dehydrogenation reactions between 1,4-butanediol and γ-butyrolactone result in a potential hydrogen storage system.^600^

Table 14 shows some of the more recent examples of the transformation of 1,4-butanediol into γ-butyrolactone. All examples use metal catalysis, which is not desirable; however, the conversion of 1,4-butanediol is generally very high, as well as the selectivity toward γ-butyrolactone. In general, the transformations are carried out in the vapor phase at elevated temperature, which result in an energy intensive process. However, readily available and safe metals such as copper can be used for catalysis. Nonetheless, one of the main disadvantages to these catalysts is their reduction in activity over time. Dopants such as La_2_O_3_ and Cr_2_O_3_ can be used to reduce catalyst poisoning further increasing the lifespan of the metals.

**Table 14:** Catalysts, Conditions and Yields for γ-Butyrolactone from 1,4-Butanediol

Catalyst	Solvent	Temperature (°C)	Pressure (bar)	Conversion (%)	Selectivity (%)	Yield (%)	Ref.
Cu/SiO_2_	No solvent	250	1	>99	>99	>99	(601)
Cu/SiO_2_	No solvent	250	1	nr	nr	97	(602)
Cu/Al_2_O_3_	1,4-Dioxane	250	16	100	98	98	(603)
Cu(Co,Cr,Zn)/MgO	No solvent	250	1	95	96	91	(598)
Cu/La_2_O_3_-ZrO_2_	No solvent	250	1	97	96	93	(604)
Cu/CeO_2_	No solvent	250	1	93	98	91	(605)
Cu/SiO_2_	No solvent	250	1	100	98	98	(606)
Cu/CeO_2_-Al_2_O_3_	No solvent	240	1	100	99	99	(607)
Cu/MgO	No solvent	250	1	70	99	69	(599)
Cu_2_/Zn_2_Mg_2_Al_2_O_7_	No solvent	240	1	71	>99	70	(608)
Au/FeO_*x*_	TBP	140	12.5	100	86	86	(609)
Au-Fe/Al_2_O_3_	TBP	140	12.5	100	88	88	(610)
Au/Mn_2_O_3_	TBP	120	12.5	98	98	96	(611)
Co-Cu/MgO	No solvent	250	1	95	98	96	(612)
Ir/bipy	1,2-DME	84	1	nr	nr	100	(600)

The hydrogenation of maleic anhydride is an important process for the production of several chemicals including γ-butyrolactone, tetrahydrofuran and succinic anhydride. In Table 15, several catalysts can be seen with their associated conditions. Many of these catalysts lose potency after several hours of reaction, so the yield of butyrolactone mentioned is the maximum yield at any point of reaction. There are two main methods for the production of γ-butyrolactone derived from maleic anhydride. First, the use of supercritical CO_2_ (sCO_2_) as a solvent, which is environmentally friendly and is excellent at dissolving reactive gases such as hydrogen and oxygen; however, they require expensive palladium-based catalysis. The alternative method uses gas phase hydrogenation of maleic anhydride, which requires lower pressures and employs the use of cheaper metal catalysts such as copper. The Cu-CeO_2_ catalysts employed are very promising showing 100% conversion of maleic anhydride with complete selectivity toward γ-butyrolactone.

**Table 15:** Catalysts, Conditions and Yields for Γ-Butyrolactone from Maleic Anhydride; sCO_2_, Supercritical Carbon Dioxide

Catalyst	Solvent	Temperature (°C)	Pressure (bar)	Conversion (%)	Selectivity (%)	Yield (%)	Ref.
Pd/Al_2_O_3_	scCO_2_	200	120	100	80	80	(613)
Pd/Al_2_O_3_	scCO_2_	200	120 CO_2_, 21 H_2_	100	80	80	(614)
Pd/C	scCO_2_	200	140	100	93	93	(615)
Cu-CeO_2_/Al_2_O_3_	No solvent	220	1	100	100	100	(616)
Cu-CeO_2_/Al_2_O_3_	No solvent	240	1	100	100	100	(617)
Cu-ZnO/SiO_2_	No solvent	220	1	∼96	∼96	∼92	(618)
Cu-Ni/Al_2_O_3_-SiO_2_	No solvent	220	1	nr	90	85	(619)
Ni/Al_2_O_3_-SiO_2_	No solvent	220	1	nr	89	83	(620)

Finally, carbon monoxide can be used to make γ-butyrolactone *via* cyclotetramerization with a trinuclear titanium polyhydride complex.^621^ This method was able to yield γ-butyrolactone in 55% yield, with the by-product being methanol, thus showing a promising technology that can convert carbon monoxide into compounds useful for polymerization.

##### 4.1.3.2 γ-Valerolactone

As mentioned earlier, levulinic acid is a promising platform chemical that can be transformed into various other fine chemicals. In fact, levulinic acid is the most common precursor for γ-valerolactone production. This is because the transformation of levulinic acid into γ-valerolactone follows a simple cascade type reaction where it is initially hydrogenated and then subsequently cyclized to form γ-valerolactone. Generally, catalysts are heterogenous with molecular hydrogen used as a hydrogen source as this is generally the most efficient route that is suitable for industry. For a sustainable process, water is a good solvent from a sustainablity point of view; however, once levulinic acid is dissolved, it becomes acidic and catalyst leaching can occur, thus other solvents are frequently used. In Table 16, various examples of different catalysts and conditions can be seen, with their associated yield of γ-valerolactone. Given the numerous examples of different catalysts that have been reported, only literature examples post-2018 have been selected.

**Table 16:** Catalysts, Conditions and Yields for γ-Valerolactone from Levulinic Acid; CNT, Carbon Nanotube

Catalyst	Solvent	Temperature (°C)	Pressure (bar)	Conversion (%)	Selectivity (%)	Yield (%)	Ref.
Co-Re/TiO_2_	1,4-Dioxane	220	nr	>99	>99	>99	(622)
Co/C	Water	220	20	100	100	100	(623)
Co^a^	Water	150	30	99	92	91	(624)
Co/SiO_2_	No solvent	200	30	100	98	98	(625)
Ni/C	2-Isopropanol	200	n/a	>99	>99	>99	(626)
Ni(OAc)_2_	No solvent	200	n/a	n/a	n/a	98	(627)
Ni-Pd/Al_2_O_3_	Water	190	n/a	83	79	66	(628)
Zr-Al/β-zeolite	2-Isopropanol	170	n/a	95	n/a	90	(629)
ZrO_2_^b^	Ethanol	250	n/a	100	∼70	∼70	(630)
Ru/CNT	Water	120	30 (H_2_)	86	> 99	85	(631)
Ru/RuO_2_·*x*H_2_O	Water	100	10 (H_2_)	100	100	100	(632)
Ru/MIL-101-S_100_	Water	80	5	99	87	86	(633)
CePO_4_	2-Isopropanol	140	n/a	97	78	76	(634)
CePO_4_/Co_2_P	Water	90	40	98	97	95	(635)
Pt/ZrO_2_	Water	240	n/a	97	n/a	90	(636)
Hf/zeolite	2-Isopropanol	120	10	95	93	88	(637)
Cu-Ni/SiO_2_^c^	2-Isopropanol	120	40	99	97	96	(638)

a Prepared in ethanol.

b High surface area.

c Nanosphere. MIL-101-S_100_ is a metal organic framework.

**Figure 47:**
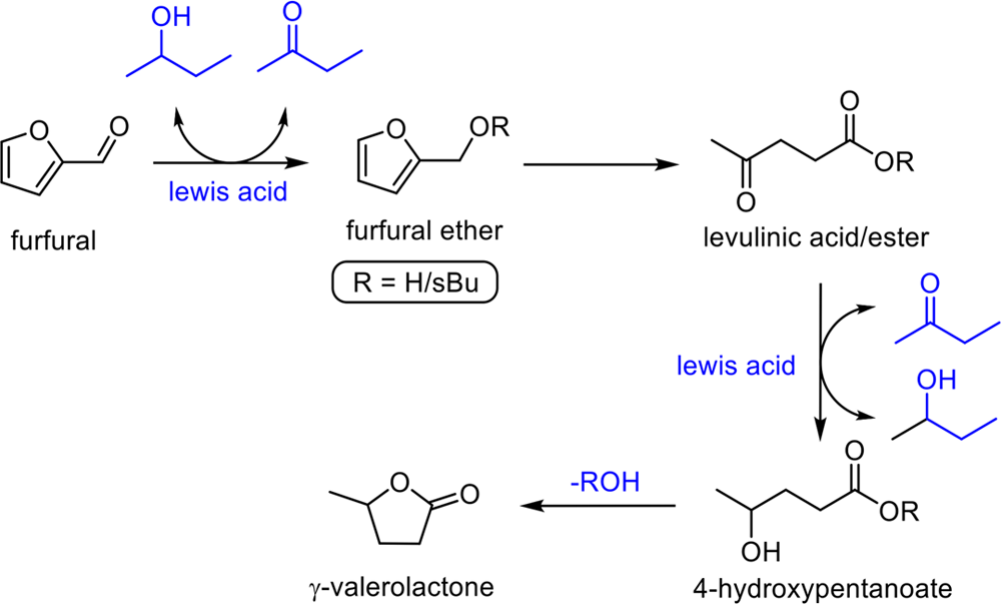
Preparation of γ-valerolactone starting from furfural.

Furfural can be converted into γ-valerolactone using a variety of different methods. Although not as well developed as the transformation of levulinic acid into γ-valerolactone, moderate yields (i.e., 65–86%) of γ-valerolactone from furfural are obtainable. The production of γ-valerolactone from furfural requires several steps.^639^ First, a transfer hydrogenation step is carried out with the use of a Lewis acid to convert the furfural into furfural alcohol/ether. Next, a Lewis acid is used to ring open the furfural alcohol/ether to a levulinate ester. The levulinate ester then undergoes a subsequent transfer hydrogenation to form 4-hydroxypentanoate, which can then be lactonized (Figure 47).

The transfer hydrogenation step is also known as a Meerwein–Ponndorf–Verley (MPV) reaction and has the benefit of avoiding the use of gaseous hydrogen. Solvents such as 2-isopropanol are typically used as they act as a proton source for hydrogenation. Popular catalysts tend to center around the use of a combination of Lewis acid metal sites embedded in alumina/silica zeolites as they are highly selective.

Table 17 highlights the most recent catalytic systems for the transformation of furfural into γ-valerolactone. Generally, Lewis acid sites in zeolites in combination with alcohols are used as a hydrogen source. One notable example is the use of organophosphate polymers prepared from vinylphosphonic acid, *p*-toluenesulfonic acid and HfCl_4_. These polymers were very effective catalysts that showed excellent recyclability, demonstrating the power of polymers as a framework for catalysts.

**Table 17:** Catalysts, Conditions and Yields for γ-Valerolactone from Furfural

Catalyst	Solvent	Temperature (C)	Pressure (bar)	Conversion (%)	Selectivity (%)	Yield (%)	Ref.
Organophosphate-Hf/polymers	2-Isopropanol	180	n/a	100	81	81	(640)
Hf/USY zeolite	2-Isopropanol	140	n/a	100	65	65	(641)
Mesoporous HSO_3_/Al_2_O_3_	2-Butanol	120	n/a	100	86	86	(642)
Zr-TPA/SiO_2_^a^	2-Isopropanol	170	n/a	100	81	81	(643)
ZrPO_4_/SAPO-34^b^	2-Isopropanol	150	n/a	100	80	80	(644)
AuRu/ZrO_2_	2-Isopropanol	150	5 (N_2_)	∼85	80	68	(645)

a TPA: terephthalic acid.

b SAPO-34: silicoaluminophosphate zeolite.

##### 4.1.3.3 Angelica Lactones

The majority of angelica lactone is produced as an intermediate or a by-product during the synthesis of γ-valerolactone, and thus the literature on angelica lactones is rather underdeveloped. However, there are examples of its use in polymer chemistry.^646,647^ Furthermore, angelica lactone contains a double bond, which could enable its radical polymerization and thereby broaden the scope of this lactone in the field of polymer chemistry.

Catalysts in the literature generally upscale platform chemicals such as levulinic acid into a range of fine chemicals including angelica lactones. However, there are examples where the synthesis of angelica lactone is targeted and thus are especially high yielding. For instance, SiO_2_ is reported to be the most efficient catalysts for the formation of angelica from levulinic acid, and this was demonstrated by Sato and co-workers when they managed an angelica lactone yield of 86% with a levulinic acid conversion of 95%.^648^ Generally, the selectivity toward α- and β-angelica lactones was around 30–40%, while γ-angelica lactone was obtained in 5% conversion. It must be noted that the reaction required decreased pressure and a temperature of around 550 K to generate this yield, highlighting how energy intensive the synthesis process is. Another example used a HZSM5/SiO_2_ catalyst to generate an angelica lactone yield of 97% consisting of 64% α-angelica lactone and 33% of the β-angelica derivative.^649^ This route was less energy intensive as it required a lower temperature of 403 K and a pressure of 0.1 bar. Montmorillonite K10 clay has been shown to be able to convert levulinic acid to angelica lactone with a yield of 92% at a temperature 438 K and pressure of 0.067 bar.^650^ Finally, WO_3_/Al-HZSM-5 catalysts can convert levulinic acid into angelica lactone and ethyl levulinate. The catalyst managed to obtain levulinic acid conversions of up to 98% with a selectivity toward angelica lactone of 75%.^651^ Many other catalysts that have been screened do not target angelica lactone in specific, and thus the corresponding yields of angelica lactone are lower, for example, the use of a Sn/Al zeolite beta catalyst to upscale furfural. The best performing catalyst system managed to convert 95% of the inputted furfural after 24 h, with the main products being furfuryl 2-butyl ether, levulinic acid, and angelica lactones with a selectivity of 29%, 14% and 23%, respectively.^652^

**Figure 48:**
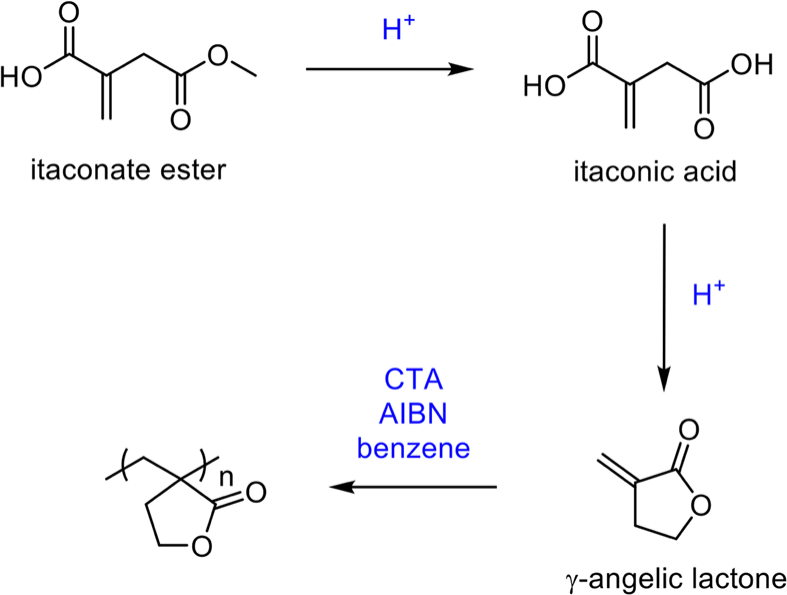
Synthesis of γ-angelica lactone from itaconic acid monomethyl ester, and its subsequent radical polymerization.

Itaconic acid can be used as a bio-based starting material for γ-angelica lactone. Itaconic acid is one of the top valued chemicals from biomass as described by the US Department of Energy, and can be used to form γ-angelica lactone (Figure 48).^485,653^ First, itaconic acid monomethyl ester need to be partially reduced before it undergoes intramolecular cyclization to form γ-angelica lactone. This has been previously demonstrated to give a yield of 40–45%. The thus synthesized γ-angelica lactone was subsequently polymerized through the double bond to yield a polymer with pendant lactone groups, which could potentially then undergo ring-opening post-polymerization modification.

##### 4.1.3.4 Other 5-Membered Lactones

1,3-Dihydroxyacetone is a triose sugar that can be derived from biomass, which when combined with formic acid can be converted into α-hydroxy-γ-butyrolactone with the use of a Lewis acid catalyst, typically using tin as the metal center. Furthermore, these catalysts can also be used to convert other chemicals such as ethylene glycol and glycerol into lactones.^654^

#### 4.1.4 4-Membered Lactones

Much of the research into the synthesis of propiolactone is antiquated, of which many routes cannot be considered to be sustainable, with many procedures using multiple steps and harsh chemicals.^655,656^ Of the very few methods to synthesis propiolactones, however, one route using ethylene oxide as a precursor material for the synthesis of β-propiolactone is a notable exception.^657^ The latter relies on the carbonylation of ethylene oxide with carbon monoxide in the presence of a bimetallic aluminum/cobalt catalyst, resulting in >99% conversion of the ethylene oxide feed with a selectivity of over 96% toward β-propiolactone. Importantly, ethylene oxide can be derived from sustainable sources and thus this route is important for sustainable propiolactones (PLs).^658^

#### 4.1.5 Other Lactones

Fatty acids can be used to make macrolactones, which can also be contracted to make 5- and 6-membered lactones with the use of a W(OTf)_6_ catalyst.^659^ Indeed, this method was able to produce γ-caprolactone from ε-caprolactone in 94% yield. Furthermore, the natural abundance of fatty acids makes this method an exciting prospect for further research.

**Figure 49:**
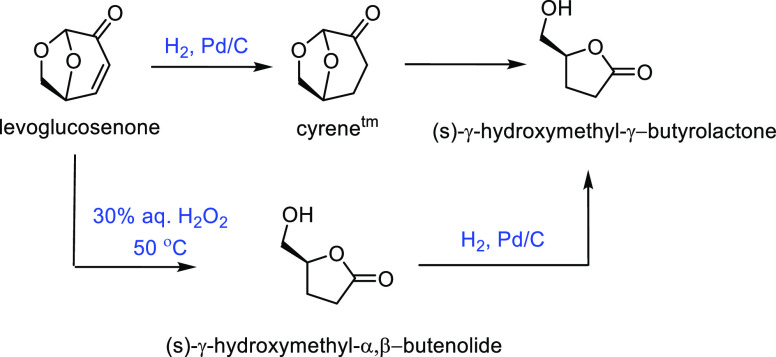
Formation of (*S*)-γ-hydroxymethyl-α,β-butenolide and (*S*)-γ-hydroxymethyl-γ-butyrolactone derived from levoglucosenone.

Levoglucosenone is a 7-membered ring type structure that can be hydrogenated to form Cyrene, which is an emerging green, dipolar aprotic solvent that can replace less sustainable solvents such as DMF.^504^ Furthermore, levoglucosenone and Cyrene can be oxidized with the use of aqueous hydrogen peroxide to form (*S*)-γ-hydroxymethyl-α,β-butenolide and (*S*)-γ-hydroxymethyl-γ-butyrolactone, respectively (Figure 49). The conditions used were reasonably mild and the yields were generally above 80%, highlighting a facile route to sustainable lactones.^660^ Furthermore, the (*S*)-γ-hydroxymethyl-α,β-butenolide could be converted into the lactone *via* hydrogenation.

As highlighted, multiple sustainable sources have been explored to produce various lactones that can serve as feedstock for ring-opened polymers. Some of the precursors used are more sustainable than others, and there is variety in the “greenness” of the transformations to lactones. A wide range of catalysts, solvents, temperatures, and pressures can be used with varying degrees of success. Furthermore, any polyester synthesized from the ring-opening polymerization of a lactone should theoretically be biodegradable due to the regular ester linkages that can be severed under acidic or basic conditions.

### 4.2 Lactams

**Figure 50:**
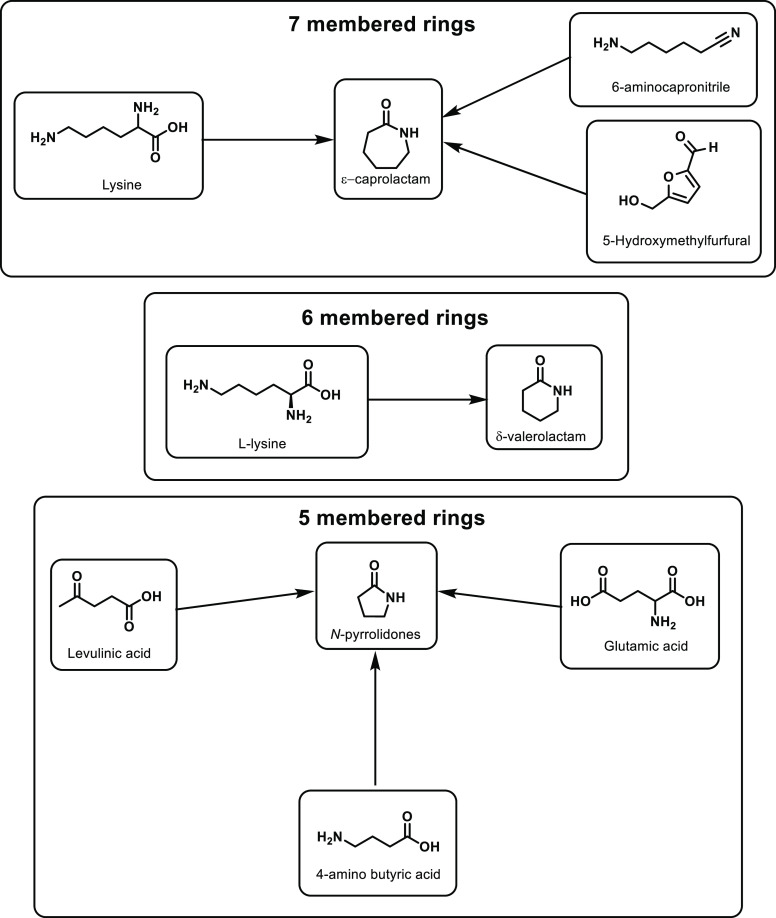
Sources of bio-based, sustainable lactams.

Lactams are cyclic amides with an analogous structure to lactones, hence providing some overlapping opportunities as a chemical feedstock. For instance, the mechanism for the ring-opening polymerization of lactams is essentially the same as that of lactones, except that a polyamide is formed instead of a polyester. Amide bonds are more difficult to degrade in comparison to esters and this results in more durable and resistant polymers. In this section, the synthesis of ε-caprolactam from sustainable feedstocks is discussed, followed by δ-valerolactam, then *N*-pyrrolidones (Figure 50).

#### 4.2.1 7-Membered Lactams

ε-Caprolactam is one of the most important plastic commodity chemicals in the world as the ring-opening polymerization of ε-caprolactam produces Nylon-6. Nylon-6 is very tough and extremely durable, thus difficult to degrade, and so finds applications as weather-resistant fibers and materials.^661^ ε-Caprolactam is generally made on an industrial scale *via* the Beckmann rearrangement of cyclohexanone; however, there are several major environmental disadvantages to this route. First, cyclohexanone is produced from the aerobic oxidation of cyclohexane, but the conversion must be kept extremely low (i.e., 3–6%) to maintain high selectivity. Second, the major by-product of the conversion of cyclohexanone to ε-caprolactam is ammonium sulfate. Although the ammonium sulfate by-product is not wasted as it finds use as a fertilizer, it does decompose to sulfuric acid and subsequently acidifies soil and water sources.^662^ While many research efforts have been devoted to reduce the formation of ammonium salt by-products, replacing the cyclohexanone feed by alternative sources could present more sustainable production opportunities.

**Figure 51:**
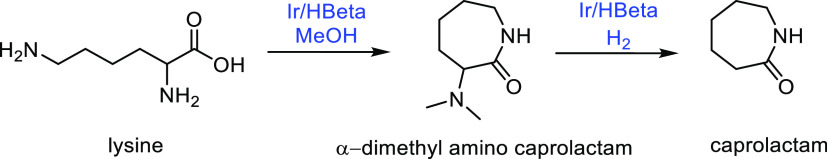
Ring closure of lysine to α-dimethyl amino caprolactam followed by amine removal with Ir catalyst.

An extremely promising sustainable feedstock due to its abundance is lysine. It is produced industrially as a side product of the fermentation of glucose, and 2.5 million metric tons were produced in 2016.^663^ The ring closure step of lysine to α-dimethyl amino caprolactam is fairly straightforward, but the subsequent removal of the amine in a simple, environmentally benign manner is more complex (Figure 51). However, Zhang and co-workers achieved both steps *via* a one-pot catalytic conversion using an Ir/HB-124 catalyst.^664^ Nonetheless, the best achieved ε-caprolactam yield was 30%, which is far from ideal in the context of industrial manufacturing and shows that further improvement is needed.

ε-Caprolactam can also be synthesized from 6-aminocapronitrile, which in turn can be produced from butadiene, which can be derived from biomass.^665,666^ Coote and co-workers used high-temperature water to hydrolyze the terminal nitrile on 6-aminocapronitrile to form 6-aminocaproic acid, which could then be ring closed to form ε-caprolactam. This method used water as a benign solvent and used a flow reactor method to generate a 90% ε-caprolactam yield with a calculated residence time of 96 seconds at 673 K and 400 bar. This route demonstrates a promising alternative to the traditional Beckmann rearrangement of cyclohexanone, but the sustainability of the conversion of butadiene into 6-aminocapronitrile is debatable, as a Michael reaction between 1,3 butadiene and hydrogen cyanide is required, followed by partial hydrogenation.^662^

**Figure 52:**
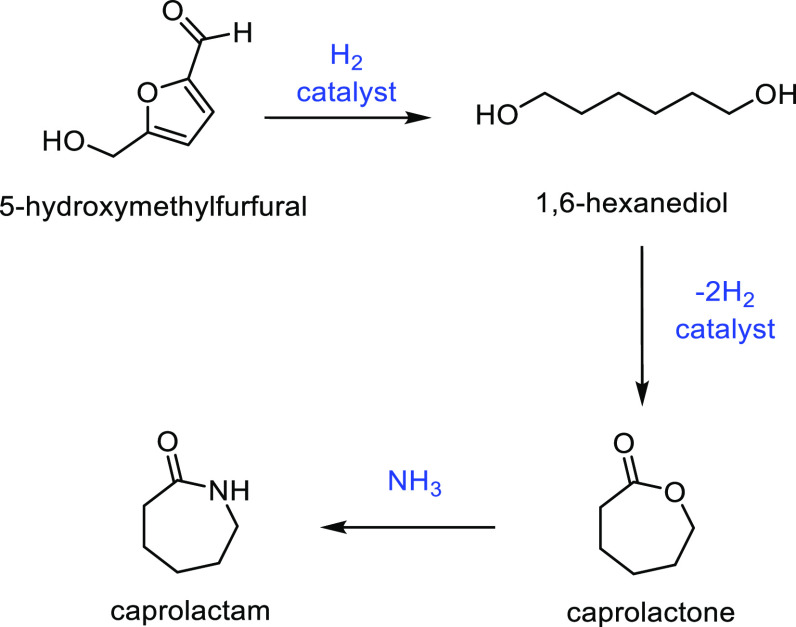
Synthesis of caprolactam from 5-hydroxymethylfurfural.

5-Hydroxymethylfurfural has also been shown to be a feasible precursor for ε-caprolactam. Deuss and co-workers demonstrated the conversion of 5-hydroxymethylfurfural into 1,2,4-benzenetriol *via* a Lewis acid catalyzed rearrangement, which could then be deoxygenated to form cyclohexanone.^667^ Nonetheless, the maximum yield of 1,2,4-benzenetriol was 55 mol % and metal catalysts, high temperatures, and high pressures were required to generate cyclohexanone, which then requires further transformation into ε-caprolactam. As previously mentioned in the section on caprolactone, Heeres and co-workers derived a method to also generate also caprolactam from 5-hydroxymethylfurfural. The latter being converted into ε-caprolactam in a 4-step-one-pot reaction (Figure 52).^549^

There are several promising routes toward the sustainable production of ε-caprolactam; however, currently they are somewhat hampered by less than desirable yields, high pressures and temperatures and rare earth catalysts. Perhaps the best course of action currently is to improve the current process of the Beckmann rearrangement of cyclohexanone and devise methods to generate cyclohexanone more sustainably.

#### 4.2.2 6-Membered Lactams

**Figure 53:**
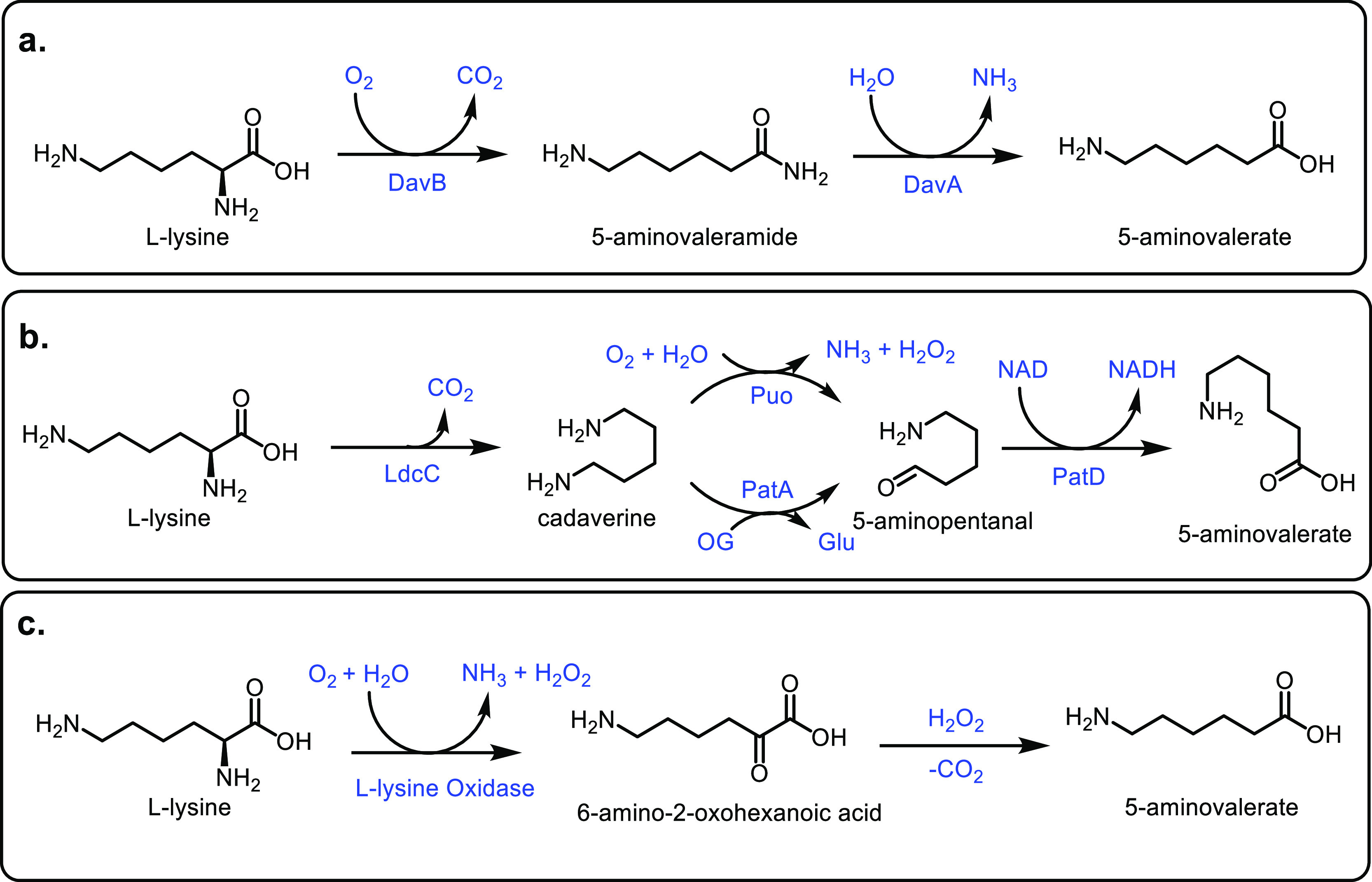
Three biological pathways to 5-aminovaleric acid.

The most well-known 6-membered lactam is δ-valerolactam, which can be used to make nylons. The majority of synthetic routes to δ-valerolactam *via* a bio-based sourced chemical are through the conversion of l-lysine into 5-aminovalerate, which can subsequently be ring-closed into the lactam. Generally this conversion is carried out metabolically by genetically engineered *E. coli*, of which there are three main methods (Figure 53).

The first method (Figure 53a) involves *E. coli* engineered to express DavA (δ-aminovaleramidase) and (DavB l-lysine 2-monooxygenase) genes. DavB initially strips l-lysine of the amine in the 2-position and converts the carboxylic acid into an amide, which DavA then converts back into a carboxylic acid to form 5-aminovaleric acid. Examples in the literature have managed to generate yields of 20.8 g/L 5-aminovalerate from 30 g/L l-lysine in 12 h,^668^ and 36.5 g/L from 60 g/L l-lysine in 24 h.^669^ Another method incorporated an acyl-CoA ligase known as ORF-26 to ring close the 5-aminovalerate to generate 705 mg/L δ-valerolactam from 10 g/L l-lysine.^670^

A second method (Figure 53b), which has been developed by Wendisch and co-workers, involves a three-step metabolic pathway.^671^ First, l-lysine is decarboxylated with l-lysine decarboxylase to yield cadaverine, which is subsequently converted into 5-aminopentanal with the use of putrescine transaminase, followed by oxidation to the carboxylic acid with γ-aminobutyraldehyde. This method typically yields around 5 g/L of 5-aminovalerate when using shake flasks.^672^

Thirdly, l-lysine α-oxidase can be used to oxidize the 2-position of l-lysine to form 6-amino-2-ketocaproic acid, which also generates hydrogen peroxide (Figure 53c). The H_2_O_2_ can then react with the 6-amino-2-ketocaproic acid to form 5-aminovaleric acid *via* an oxidative decarboxylation. In the method developed by Franssen and co-workers, the l-lysine α-oxidase was immobilized on an epoxy activated solid support to give a synthesis method with 5-aminovaleric acid yields of up to 95%.^673^ During the process, there is an equilibrium mixture, which could be influenced by pH to favor δ-valerolactam at higher pH.^674^ Furthermore, they managed to generate yields of 6.88 g/L of δ-valerolactam from 40 g/L l-lysine.

Other bio-based chemicals other than lysine can be used for the production of δ-valerolactam. For example, triacetic acid lactones can be biosynthesized from glucose.^675^ These lactones can then be converted into lactams as demonstrated by Han and co-workers in 2021.^676^ They initially reacted the lactone with ammonia to form 4-hydroxy-6-methylpyridin-2(1*H*)-one, which was then selectively transformed into 6-methylpiperidin-2-one with a Ru catalyst to give yields of up to 77%.

The production of sustainably sourced δ-valerolactam is quite well developed, with l-lysine being an abundant natural feedstock. Moreover, the use of biochemistry means metal catalysts are not generally required, and harmful by-products are limited.

#### 4.2.3 5-Membered Lactams

**Figure 54:**
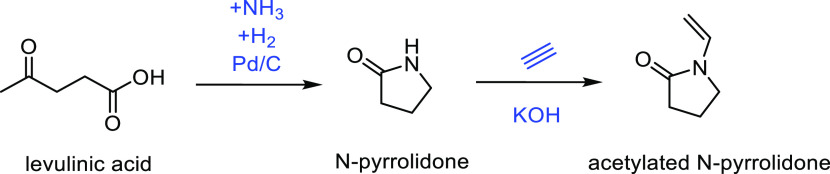
Production of acetylated *N*-pyrrolidones from levulinic acid.

Butyrolactam, or 2-pyrrolidone, is the most basic 5-membered lactam, and can be functionalized *via* the R group on the nitrogen to yield materials with additional functionality.^677^ The most common ways of generating butyrolactam sustainably tend to be from the platform chemical levulinic acid. The reductive amination of levulinic acid has been researched quite thoroughly, with both heterogenous and homogenous catalyst being investigated. An excellent heterogenous catalytic system was developed by Shimizu and co-workers, where they demonstrated the amination with a wide range of amines with generally high yields and no solvent.^678^ Another example used Zr-Co catalysts prepared using chitosan as a carbon and nitrogen source to generate 5-methyl-2-pyrrolidone with a 93% yield.^679^ Homogenous catalysts were first reported in 2011 with the use of a ruthenium catalyst and formic acid.^680^ This was an effective method; however, it had the disadvantages of requiring high catalytic loading while displaying poor reactivity with bulky amines. In contrast, Fischmeister and co-workers developed a homogenous Iridium catalyst system using hydrogen as a proton source, which could tolerate bulky amines and required low catalyst loadings.^681^ A catalyst-free system was developed in 2015 that could generate *N*-pyrrolidones with yields between 80% and 90%; however, temperatures up to 473 K were required.^682^ An electrocatalytic approach was taken by Palkovits and co-workers, which has the advantage of being metal-free, and generates very good yields at temperatures between 80 and 150 °C, H_2_ pressures of up to 5 MPa and within a 1–24 h reaction time.^683^ Finally, 2-pyrrolidone synthesized from levulinic acid was functionalized by reaction with acetylene at 150 °C and 15 bar to generate vinyl monomers with yields of up to 82% (Figure 54).^684^ These could then be polymerized through the vinylic groups and then subsequently ring-opened to form complex polymeric structures; however, one of the issues with the method is that the catalyst was not recoverable as the carbon support broke down.

As well as levulinic acid, other bio-based acids such as glutamic acid can be converted into butyrolactam. γ-Aminobutyric acid can easily be synthesized from glutamic acid, which can then be converted into *N*-methyl-2-pyrrolidone.^685^ This can be done in a simple one-pot reaction with the use of methanol as a methylating agent in the presence of a halogen salt such as ammonium bromide. This particular method shows both high selectivity up to 90% and high conversions.^686^ This method was also attempted with dimethyl carbonate as the methylating agent in the presence of a NaY zeolite; however, the selectivity was much lower at around 67%. To avoid the conversion step of glutamic acid into γ-aminobutyric acid, glutamic acid can be directly converted into various *N*-alkyl-2-pyrrolidones in a one-pot reaction.^687^ First, the amine is reacted with a ketone and subsequently reduced under mild conditions with a palladium catalyst, and the subsequent product is ring closed followed by decarboxylation at 250 °C with a Pd/Al_2_O_3_ catalyst. This route does provide reasonable yields but also requires metal catalysis and high temperatures, which detract from the sustainable aspect of the route.

Finally, butyrolactam has been synthesized from 4-aminobutyric acid *via* a metabolic pathway using engineered *E. coli*. Titers could be produced of up to 54 g/L and other lactams such as caprolactam and valerolactam were also possible but at not as high a titer.^688^

The variety of sustainable precursors for lactam synthesis is not as well developed as for lactones. However, l-lysine stands out as being a very promising feedstock as it is abundant in nature and can be transformed into ε-caprolactam and δ-valerolactam. Although polyamides are very robust and durable polymers, they are also much more difficult to degrade than polyesters and so reside in the environment for extended periods.

### 4.3 Lactides

**Figure 55:**
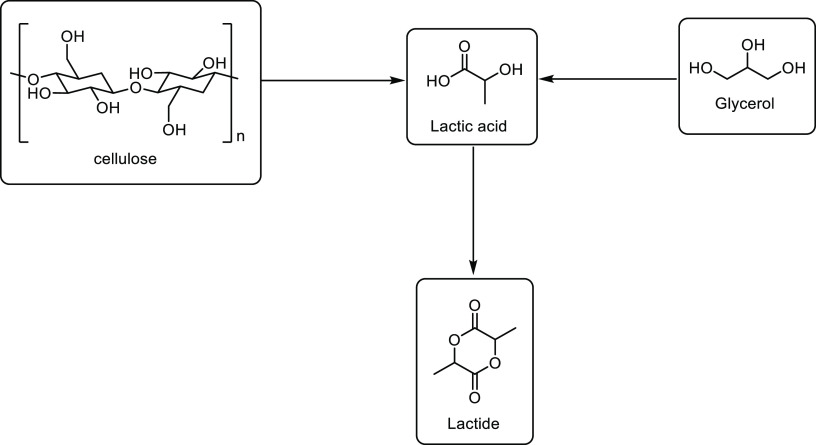
Sources of bio-based and sustainable lactides.

Polylactic acid (PLA) is perhaps one of the most promising biodegradable, biocompatible and renewable polymeric materials in today’s polymer landscape. These attributes of PLA make it suitable for a range of applications such as inkjet printing,^689^ tissue engineering^690^ and bone fixation.^691^ Although PLA can be produced from lactic acid, several disadvantages are associated with this direct polymerization, including difficulty reaching high monomer conversion, side reactions such as intermolecular transesterifications, and racemization between l-lactic acid and d-lactic acid.^692^ Alternatively, lactic acid can be converted into the cyclic lactide intermediate, which can be subjected to ring-opening polymerization, thereby allowing for the formation of PLA is a very controlled manner with high molecular weights and low dispersities.^693^ In the following, we will focus the discussion on the synthesis of lactic acid from renewable sources, as virtually all lactide is synthesized from lactic acid (Figure 55).

As of 2018, the global lactic acid market was valued at $2.9 billion, and is expected to grow to between $8 and $10 billion by 2025.^694^ Currently, the majority of lactic acid is produced *via* the fermentation of carbohydrates; however, this route has several disadvantages including long fermentation times, low substrate concentration and complicated purifications.^695^ There are three main chemical synthesis routes to convert glycerol into lactic acid, i.e., dehydrogenation, selective oxidation and hydrothermal conversion.

**Figure 56:**
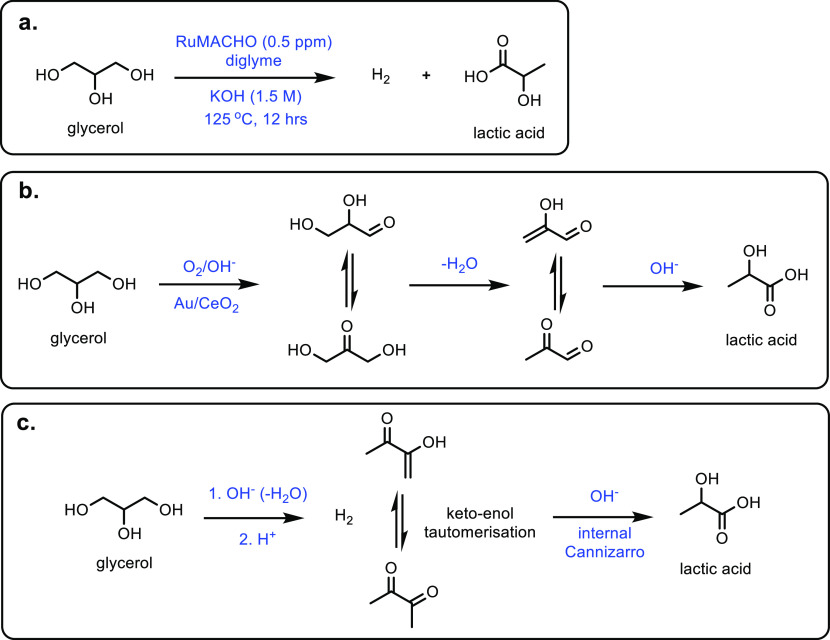
Use of RuMACHO for dehydrogenation of glycerol (a). Au nanoparticles on CeO_2_ for lactic acid production (b). Hydrothermal conversion of glycerol into lactic acid (c).

Glycerol can be dehydrogenated to generate lactic acid and hydrogen gas by means of a technique developed by Cole-Hamilton and co-workers in 1988 using a RuH_2_N_2_(Ph_3_P)_3_ catalyst.^696^ This method was primarily developed for hydrogen generation, but has been further developed by making use of a RuMACHO (RuHCl(PNPPh)CO) catalyst (Figure 56a). Using this catalyst, complete conversion of glycerol with 67% lactic acid yield was realized.^697^

Selective oxidation can be used to generate a variety of oxygenated products from glycerol including lactic acid (Figure 56b). Several catalysts such as CeO_2_ supported gold nanoparticles and Pt/activated carbon^698,699^ generate high yields of lactic acid, but require basic catalysts that result in further purification steps to obtain lactic acid. Fan and co-workers developed a Pt/Sn-MFI catalyst that could generate lactic acid from glycerol with a selectivity of 81% and a conversion of 90%. Furthermore, the route does not require a base, implying a greener synthesis route than traditional oxidative routes of lactic acid.^700^

Hydrothermal conversion is a good technique for obtaining low molecular weight organic acids, and the generation of lactic acid from glycerol is a good example of this (Figure 56c). Yields of up to 90% have been reported previously using a basic solution of 0.33 M glycerol. However, such a low substrate concentration is a major disadvantage for an industrial viable process.^701^ Since then, the process has been improved to give a lactic acid yield of ∼80% when using an initial glycerol concentration of 2.5 M.^702^

Cellulose is another common precursor for lactic acid, and the transformation from cellulose to lactic acid involves three distinct steps. First, cellulose is hydrolyzed into glucose using a Brønsted acid. The glucose is then isomerized into fructose, which is then converted into lactic acid *via* a retro-aldol reaction. The conversion of glucose into lactic acid requires a catalyst with Lewis acidity, of which Ba(OH)_2_ has been shown to be a promising example, showing a lactic acid yield of 95% after 48 h from treatment of glucose under anaerobic conditions. Nevertheless, this method requires low substrate concentrations to achieve high conversions resulting in an issue for any potential industrial interest.^703^ Various other soluble Lewis acid salts have been used, including Pb^2+^, VO^2+^, Er^3+^ and Al^3+^/Sn^2+^.^704−707^ Pb^2+^ has been shown to be able to convert ball-milled cellulose into lactic acid with a 68% yield; however, the toxicity of the salt can be problematic.^704^ ErCl_3_ is an extremely promising catalyst, showing a quantitative conversion of cellulose with 91% yield of lactic acid; however, this method is hampered by low substrate concentrations.^706^

One of the main issues with using homogenous Lewis acid salts is the difficulty in purifying them, which gives heterogenous catalysts an advantage industrially. An Erbium exchanged montmorillonite K10 was developed as a heterogenous catalyst by Dong and co-workers that could transform cellulose with 100% conversion, with a lactic acid yield of up to 68%. However, the catalyst was shown to undergo structural changes upon recycling resulting in decreased catalytic performance.^708^ To build on this, Lappas and co-workers developed a *p*-toluenesulfonic acid (TSA)/SiO_2_-Al_2_O_3_ catalyst that demonstrated a selectivity of 38% toward lactic acid, with a yield of 24%. Importantly, the catalyst was shown to be reusable for at least 3 reaction cycles.^694^

### 4.4 Cyclic Olefins for Ring-Opening Metathesis Polymerization

**Figure 57:**
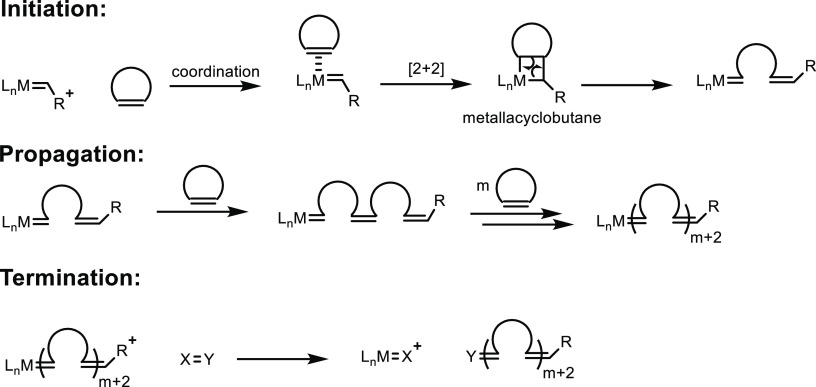
General mechanism for ring-opening metathesis polymerization (ROMP).

Ring-opening metathesis polymerization (ROMP) is a type of olefin metathesis reaction that uses relief of ring strain as its driving force. ROMP has increased in popularity since the 1950s because of the development in its mechanistic understanding, leading to well-defined catalysts and consequently polymers with more complex architectures.^709^ One of the most important features of olefin metathesis reactions is that any unsaturation present in the monomer is retained in the polymer. Common catalysts for ROMP are transition metal alkylidene complexes, which undergo a [2+2] cycloaddition with the cyclic olefin to form a metallacyclobutane, followed by a cycloreversion to produce a new metal alkylidene (see Figure 57 for general ROMP mechanism). This process repeats until all the monomer is consumed or a termination event takes place. In this section, cyclic olefin monomers that are susceptible to ROMP and are derived from sustainable sources will be discussed.

**Figure 58:**
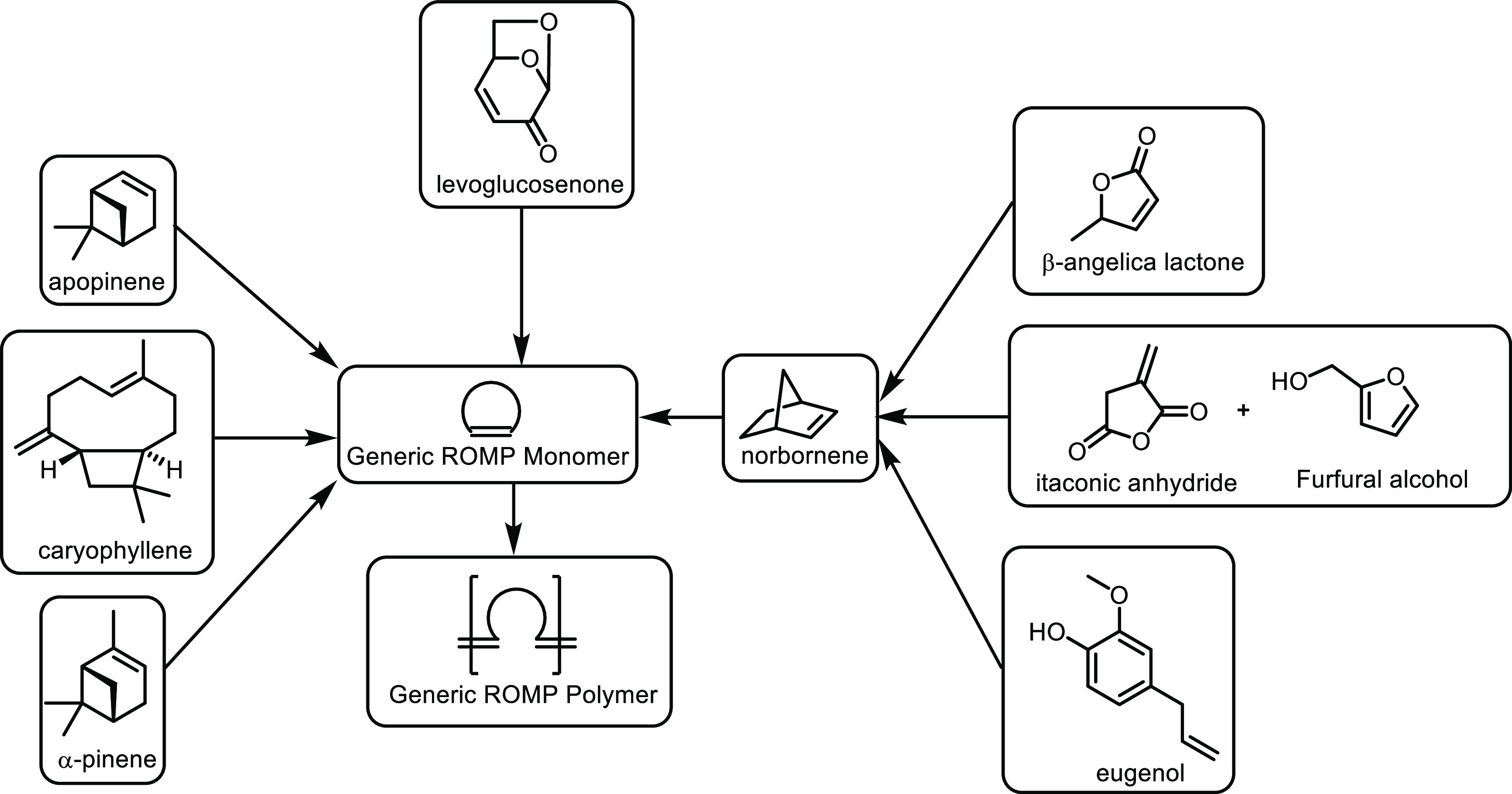
Sources of bio-based, sustainable monomers for ROMP.

Suitable monomers for ROMP require cyclic structures with an internal double bond, and these types of structures can be found naturally as terpenes. Indeed, research into terpenes such as α- and δ-pinene for ROMP is already prevalent and we will first begin by discussing monomers derived from them (Figure 58).

**Figure 59:**
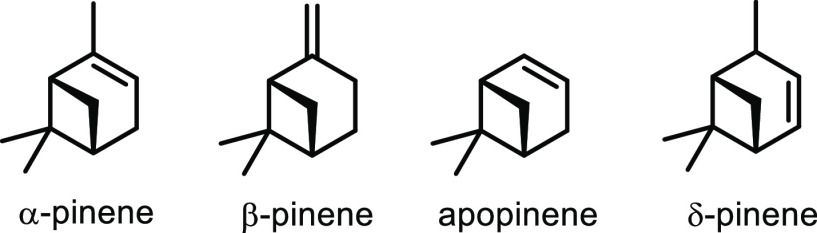
Chemical structures of pinene derivatives.

Terpenes make up a large market, with around 350 kilotons being harvested per annum. Generally, α-pinene is the major volatile component making up between 45-97% of pine sap. α-Pinene contains an internal double bond that is rather sterically hindered, making its polymerization difficult. For this reason, α-pinene is normally transformed into better polymerizable monomers such as δ-pinene or apopinene (Figure 59).

Apopinene can be synthesized by allylic oxidation of α-pinene with SeO_2_ followed by decarbonylation with Pd/BaSO_4_. Using a 3^rd^ generation Grubbs catalyst, apopinene can be polymerized to yield polymers of up to 16 kDa and dispersity values as low as 1.6.^710^ An alternative route to make use of α-pinene is by converting it into δ-pinene, which has previously been realized in a 3-step process followed by a distillation step.^711^ The resulting polymers in this case had *M*_n_ values of 70 kg/mol and low dispersity values of around 1.2. Both of these methods highlight the potential for pinenes to act as bio-based precursors for monomer synthesis; however, the synthetic routes are far from sustainable. Moreover, the distillation step in the δ-pinene requires reaction of α-pinene with chlorosulfonyl isocyanate to create a boiling point disparity between the pinenes to allow for distillation. To remove this difficulty, Kennemur and co-workers investigated the effect of α-pinene acting as a co-solvent during the polymerization of δ-pinene.^712^ They discovered that the polymerization of δ-pinene was still effective as long as the molar ratio of α-pinene to the 3^rd^ generation Grubbs catalyst did not exceed 40:1.

Caryophyllene is a sesquiterpene that makes up about 10% of clove oil and is generally burned as a waste product. However, caryophyllene contains a cyclobutene moiety and an exocyclic double bond, resulting in a structure with excellent potential for further transformation. Poly(caryophyollene) can be made with molecular weights of up to 20 000 g/mol with dispersity values of 1.8–1.9 with a generation III Grubbs catalyst.^713^ Furthermore, the exocyclic bonds present in poly(caryophyllene) can be cross-linked by various chemical strategies (e.g., thiol–ene reaction) to generate polymers with storage moduli between 1 and 100 MPa and an elongation until break of up to 360%.^714^

**Figure 60:**
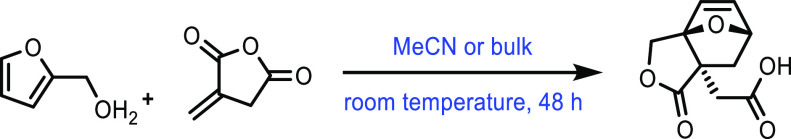
Oxanorbornene-lactone ring formation from itaconic anhydride and furfuryl alcohol.

Norbornenes are the monomer of choice for ROMP due to their commercial availability, ease of synthesis and polymerisability.^715^ Although norbornene is generally produced by the Diels–Alder reaction between ethylene and cyclopentadiene, other bio-based structures can be used instead. For example, a very interesting route was developed by North *et al.* where they attempted to form an ester between itaconic anhydride and furfuryl alcohol.^716^ However, the reaction gave an oxanorbornene-lactone ring as a single product instead (Figure 60). The reaction proceeded without the need for solvents or catalysts and was 100% atom economical, highlighting a potential monomer with definite green credentials. The subsequent polymers were then used to prepare a range of different esters and tertiary amides. After reaction optimization, polymers were made with monomer conversions of up to 93% and *M*_n_ values of 23.5 kDa. Polymers synthesized had glass transition (*T*_g_) values in the range of 115–203 °C and thermal decomposition temperatures above 300 °C.^717^

Norbornenes can also be synthesized by the Diels–Alder reaction between cyclopentadiene and other double bond containing compounds, thereby broadening the monomer substrate scope. For example, Fang and co-workers performed a Diels–Alder reaction between cyclopentadiene and a fluorinated eugenol derivative to generate high molecular weight, well controlled polymers with potential application in the microelectronic industry.^718^ As well as eugenol, angelica lactones can be combined with cyclopentadiene to generate norbornene derivatives that could then be polymerized to form polymers with *M*_n_ values of up to 122 kDa. However, obtaining pure β-angelica lactone can be challenging.^719^

Norbornene can also be functionalized and subsequently polymerized to generate useful materials. Notable examples include the esterification of 5-norbornene-2-methanol with various fatty acids, followed by polymerization to generate polymers with glass transition temperatures that span a large temperature range, i.e., from −32 to +102 °C.^715^

Next to terpenes and norbornenes, levoglucosenol can also be used as a bio-based monomer source for ROMP. Levoglucosenol can be derived from the reduction of levoglucosenone, which in turn is generated from the pyrolysis of cellulose. The reduction step can be carried out simply with sodium borohydride, and the subsequent polymerization yielded polymers with high molecular masses that were shown to be degradable, highlighting a promising polymeric system that is bio-based and degradable.^720^

### 4.5 2-Oxazolines

**Figure 61:**
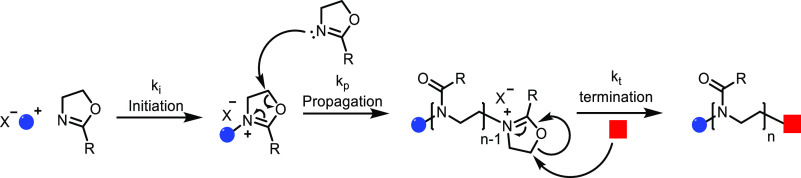
General mechanism of the cationic ring-opening polymerization of 2-oxazolines.

Poly(2-oxazoline)s are a versatile class of polyamide that are structurally analogous to polypeptides and thus can be regarded as pseudo-peptides. Poly(2-oxazoline)s are synthesized *via* the cationic ring-opening polymerization of 2-oxazoline ring structures (Figure 61), which was first carried out in the 1960s.^721^ One of the main benefits of the cationic ring-opening polymerization of 2-oxazolines is the degree of flexibility associated with this chemistry, which can be used to make complex polymeric architectures.

2-Oxazolines can easily be synthesized from carboxylic acids *via* one of three main routes.^722^ First, nonactivated carboxylic acids are reacted with ethanolamine to form the associated amide, followed by the subsequent catalyzed ring closure. The second route is known as the Witte-Seeliger method and involves conversion of a carboxylic acid to a nitrile followed by transformation into a 2-oxazoline. The third method is known as the Wenker method, where a carboxylic acid is coupled with chloroethylamine, which then undergoes ring closure with a solution of potassium hydroxide.

**Figure 62:**
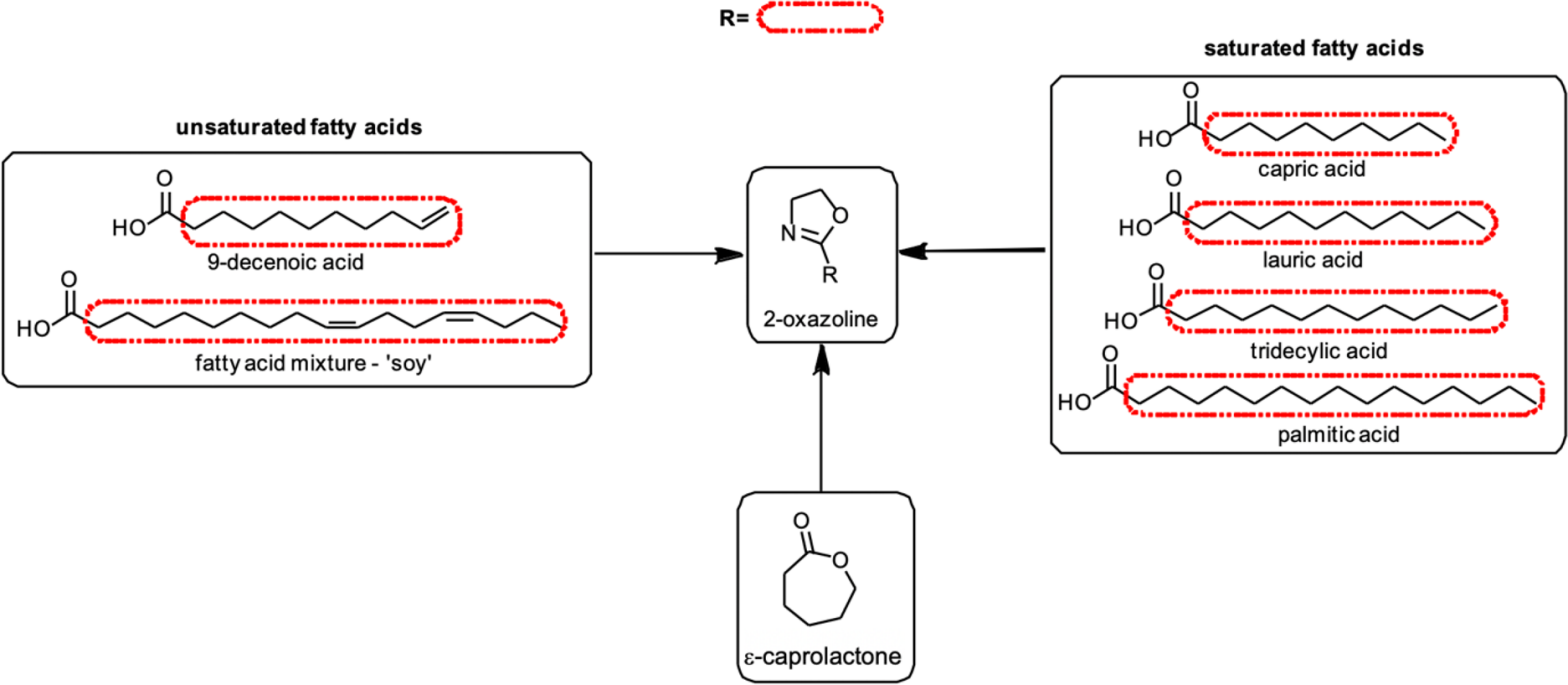
Sources of bio-based, sustainable 2-oxazoline monomers.

The majority of literature procedures on the sustainable synthesis of poly(2-oxazolines) is based on using fatty acids as a renewable feedstock (Figure 62). The resultant monomers therefore contain saturated and unsaturated hydrocarbon groups and thus are not particularly structurally diverse. However, the polymeric structures formed with 2-oxazolines derived from fatty acids have interesting physical properties such as low surface energies and high crystallinity.^723^ Nonetheless, unsaturated fatty acids can provide a route to further functionalization, as was demonstrated by Schubert and co-workers when they synthesized a 2-oxazoline from the sustainably derived fatty acid decenoic acid.^724^ The pendant double bond allowed for further modification that was realized by the thiol–ene addition reaction between the synthesized polymer and a thiolated glucose moiety. As well as glycosylation, other previous examples of functionalization of these double bonds include epoxidation, hydroboration, and hydrosilylation.^725,726^

**Figure 63:**
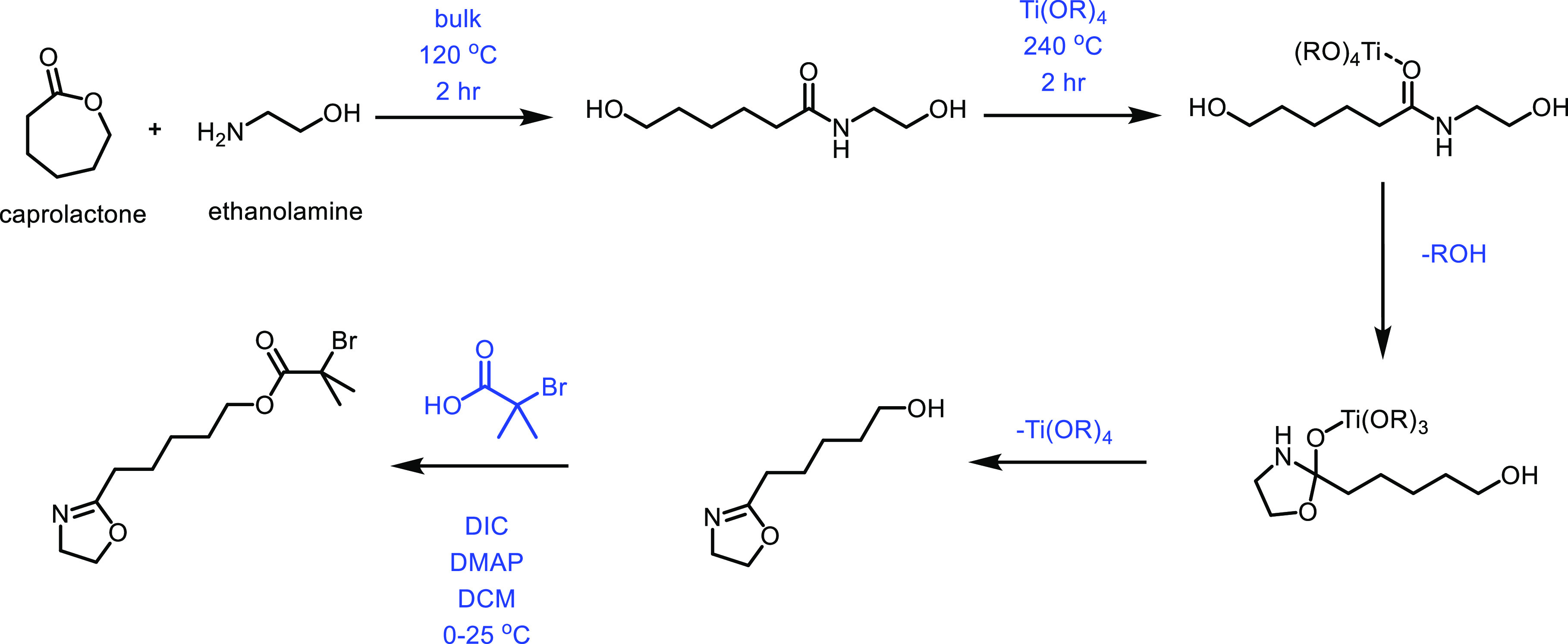
Formation of 2-oxazoline “inimer” and subsequent polymerization.

Differing from the majority of sustainable 2-oxazoline research, Becer and co-workers derived a functional monomer from caprolactone by using ethanolamine and a Lewis acid, notably the same chemicals that can be used to create 2-oxazolines from fatty acids (Figure 63). Their approach led to a monomer with pendant hydroxyl groups that could then undergo subsequent transformation. This was illustrated by grafting on an initiator for Cu(0)-mediated reversible-deactivation radical polymerization (RDRP) to form an “inimer”, and then polymerizing said inimer to form a poly(2-oxazoline) backbone with acrylate brushes.^727^

## 5 Bisfunctional Monomers Used in the Synthesis of Commodity Thermoplastics

**Figure 64:**
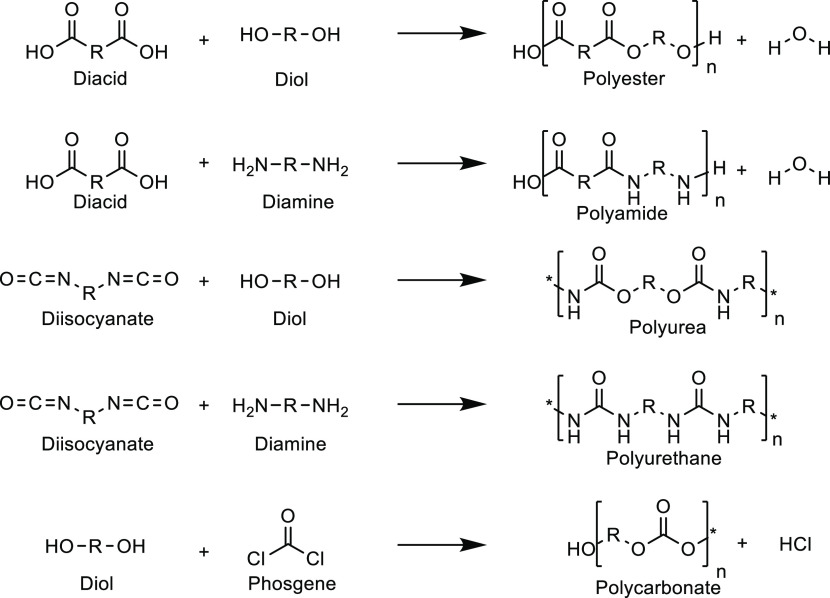
General reaction schemes for the conventional synthesis routes of common step-growth polymer classes, derived from difunctional monomers.

Step-growth polymerization is a class of polymerization wherein monomers bearing functionalities greater than 1 polymerize in a sequential manner, first forming di-, tri- and oligomers, and eventually long polymer chains. The polymerizations can either occur between two monomers bearing multiple of the same functional group (AA + BB type), or from a single monomer bearing two different functional groups (AB-type). Homo-difunctional compounds are typically easier to access than hetero-difunctional compounds and hence AA + BB type step-growth polymerizations are more commonly utilized, with the conventional synthesis of 5 major AA+BB step-growth polymer classes (i.e., polyester, polyamide, polyurea, polyurethane and polycarbonate) shown in Figure 64.

The polymer class produced *via* step-growth polymerization is determined directly by the combination of their respective monomer classes, with diacids, diols, diamines, diisocyanates and phosgene commonly used. Unlike the previous sections, which largely pertain to homopolymers made from single monomers, this section focusing on AA+BB step-growth necessitates multiple monomers for each polymer. In exploring routes from biomass to these step-growth polymers, the focus must be on the production of these key monomer classes, such that existing polymerization techniques can be used to transform renewable step-growth building blocks into the desired conventional polymer products.

### 5.1 Bifunctional Monomers

#### 5.1.1 Diacids

**Figure 65:**
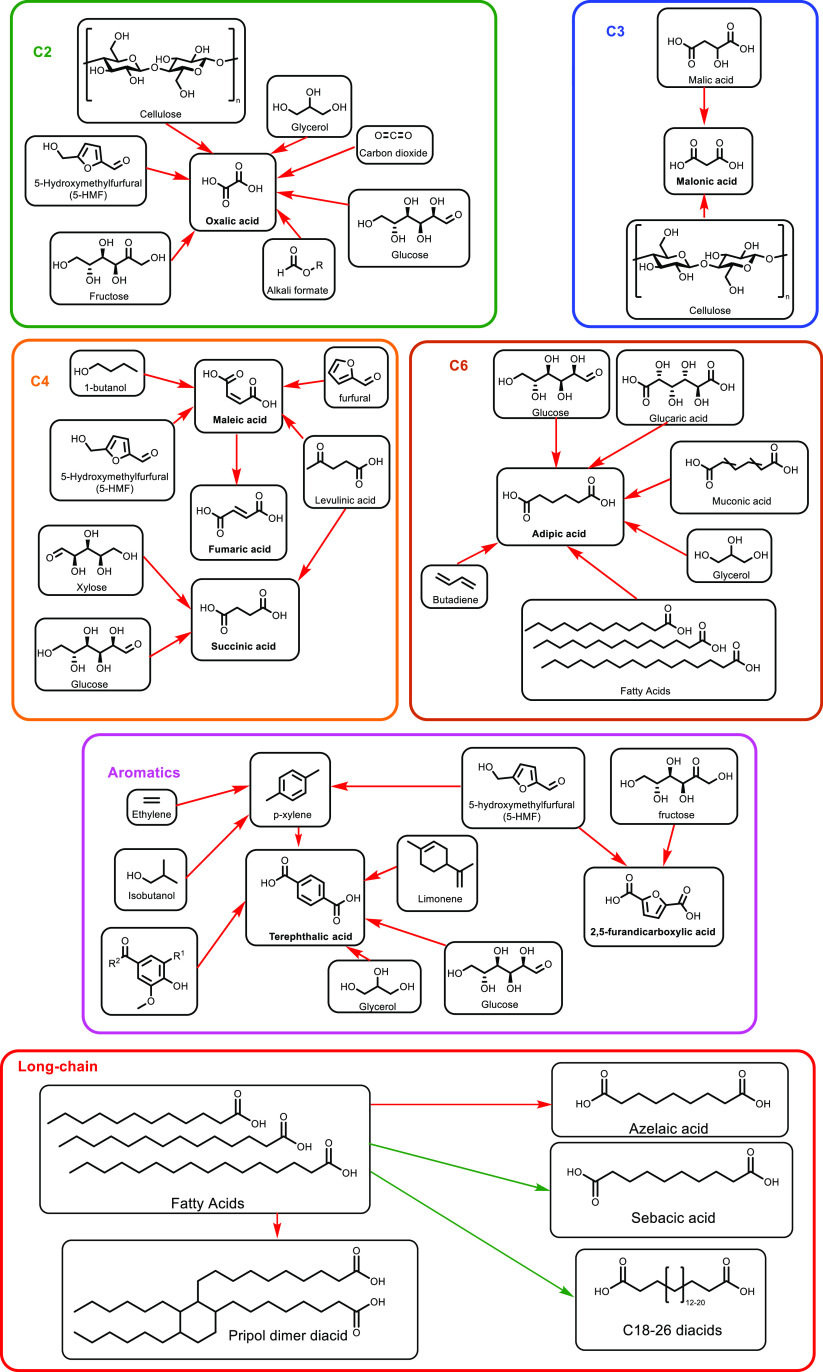
Summary of the routes to various dicarboxylic acids from biomass sources.

Diacids (dicarboxylic acids) are crucial for the synthesis of several key polymer types, including polyesters and polyamides. The wide availability, ease of synthesis and relative stability of acid moieties have enabled the preparation of a wide range of diacid compounds, although the scope of this review focuses on preparing α,ω-diacids, as these are the most industrially relevant diacid isomers (Figure 65).

##### 5.1.1.1 C2 Diacid

**Figure 66:**
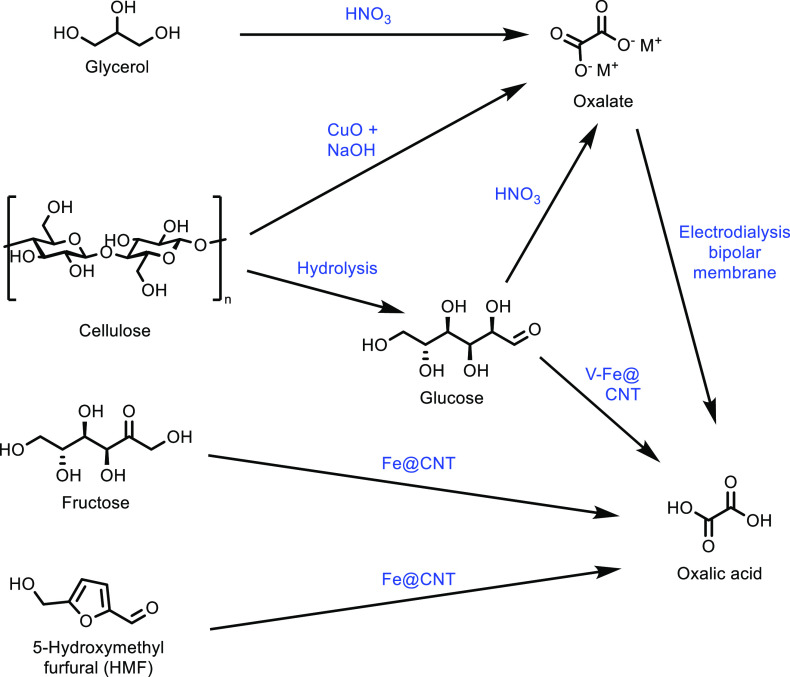
Routes to oxalic acid from sustainable biomass sources, excluding CO_2_ and CO.

Oxalic acid (OA, 1,2-ethanedioic acid) was discovered in 1734 and is currently used on a large scale in the pharmaceutical, agricultural, chemical and textile industries.^728^ Alongside its potential for use in step-growth polymerization, selective reduction of OA can yield several other commercially useful derivatives, including glyoxylic acid, glycolic acid, glyoxal, glycol aldehyde and ethylene glycol.^728^ OA is currently predominantly produced from coal-derived fossil naphtha, but routes to OA from several biomass feedstocks have been developed, including cellulose, ethanol, glycerol, glucose and other C_6_ substrates such as fructose and 5-hydroxymethlyfurfural (5-HMF), as well as from CO_2_ (Figure 66, Table 18).^729−731^

**Table 18:** Catalysts, Conditions and Yields for the Production of Selected Diacids and Important Intermediates from Biomass Sources

Entry	Target	Feedstock	Catalyst	Temperature (°C)	Pressure (bar)	Conversion (%)	Selectivity (%)	Yield (%)	Ref.
1	OA	Cellulose	CuO (+base)	200	3 (O_2_)	100	42	42	(735)
2	OA	Glycerol	Au/CeO_2_ (+base)	60	3 (O_2_)	98	56	55	(737)
3	OA	Fructose	Fe@CNT	140	10 (O_2_)	99	47	46	(738)
4	OA	Glucose	V-Fe@CNT	150	20 (O_2_)	97	48	46	(738)
5	OA	5-HMF	Fe@CNT	140	10 (O_2_)	99	49	48	(738)
6	OA	CO_2_	Cr_2_O_3_/Ga_2_O_3_@C	25	n/a	n/a	n/a	59	(743)
7	OA	K-formate	KH	190	1 (Ar)	n/a	n/a	97	(744)
8	MalonA	Cellulose	NaOH	300	n/a	n/a	n/a	40	(769)
9	MalonA	Malic acid	H_5_[PMo_10_V_2_O_40_]	100	10 (O_2_)	99	69	68	(770)
10	MalA	Furfural	V-oxides	320	0.05 (O_2_)	100	70	70	(771)
11	MalA	Furfural	H_3_PMo_12_O_40_	110	20 (O_2_)	50	69	35	(772)
12	MalA	Furfural	Ti-SiO_2_ @ Zeolite	120	n/a	99	72	71	(773)
13	MalA	Furfural	BHC	100	n/a	100	61	61	(774)
14	MalA	Furfural	Formic acid	60	n/a	n/a	n/a	95	(775)
15	MalA	5-HMF	Fe_3_O_4_@SiO_2_	110	10 (O_2_)	36	85	31	(776)
16	MalA	5-HMF	Formic acid	100	n/a	n/a	n/a	89	(775)
17	MalA	1-Butanol	Vanadyl pyrophosphate (VO)_2_P_2_O_7_	340	1 (Air)	n/a	39	n/a	(777)
18	MalA	LevA	VO_*x*_SiO_2_	300	n/a (O_2_)	100	71	71	(778)
19	FA	MA	P(4-vinylpyridine)	200	n/a	n/a	n/a	78	(779)
20	FA	MA	No catalyst	200	n/a	n/a	n/a	72	(779)
21	SA	Xylose	SO_3_H-CD	60	n/a	100	81	81	(780)
22	SA	LevA	Ru(III)@MNP, H_2_O_2_	150	14 (O_2_)	79	99	78	(781)
23	SA	LevA	Trifluoroacetic acid	90	n/a	100	62	62	(782)
24	SA	Glucose	Ru/NH_2_-rGO	161	18 (O_2_)	100	87	87	(783)
25	SA	HMF	Ru-FeO_3_@SiO_2_=CoOx	110	10 (O_2_)	90	81	73	(776)
26	SA	Fructose	Fe@CNT	140	10 (O_2_)	99	21	21	(738)
27	AA	Butadiene	Pd(II)/Rh(III)	120	50 (CO)	n/a	n/a	96	(784)
28	AA	SA	1) Pd-Re/TiO_2_, 2)Rh(PPh)_3_COCl	1) 200	1) 69 (H_2_)	1) 99	1) 86	1) 85	(785)
				2) 175	2) 48 (CO)	2) 100	2) 74	2) 74	
29	AA	GA	Pd-Rh/Davisil 635	140	49 (H_2_)	n/a	n/a	89	(786)
30	AA	MucA	Pt/C	24	24 (H_2_)	n/a	n/a	97	(787)
31	*p*-Xylene	Ethylene	1) Ir-based	1) 190	1) n/a	n/a	n/a	89	(788)
			2) no catalyst	2) 250	2) 41 (C_2_H_4_)				
			3) 400	3) Pt/Al_2_O_3_	3) n/a				
32	*p*-Xylene	Isobutanol	1) Al_2_SiO_3_	1) 327	n/a	n/a	n/a	63	(789)
			2) Al_2_SiO_3_	2) 327					
			3) CrOx/Al_2_O_3_	3) 527					
33	*p*-Xylene	Dimethyl furan	ZrO_2_	275	40 (C_2_H_4_)	99	90	89	(790)
34	TPA	Lignin oil	1) MoO_*x*_/AC	1) 320	1) 30 (H_2_)	n/a	n/a	15	(791)
			2) Pd(OAc)_2_	2) 25	2) n/a (CO)				
			3) Cu/Mn(OAc)_2_ + KBr	3) 120	3) 10 (O_2_)				
35	TPA	Glucose + glycerol	1)TiCl_4_	1) 25	1) n/a	n/a	n/a	68	(792)
			2)Pd/C	2) 100	2) n/a				
			3) Co-Mn(OAc)_2_	3) 100	3) 1(O_2_)				
36	TPA	Limonene	1) FeCl_3_	1) 50	1) n/a (N_2_)	n/a	n/a	85	(793)
			2) MnO_*x*_-FeO_*x*_	2) nr	2) n/a				
37	FDCA	HMF	Pt/N-doped-C	110	10 (O_2_)	100	99	99	(794)
38	FDCA	HMF	Pt_5_Bi_1_/C	100	40 (air)	100	>99	>99	(795)
39	FDCA	HMF	Pd/HT	100	n/a (O_2_)	100	92	92	(796)
40	FDCA	HMF	Fe_2_O_3_@HAP-Pd(0)	100	n/a (O_2_)	97	96	93	(797)
41	FDCA	HMF	Au/HY	60	3 (O_2_)	100	>99	>99	(798)
42	FDCA	HMF	Au/Ce_0.9_Bi_0.1_O_2_	65	10 (O_2_)	100	>99	>99	(799)
43	FDCA	HMF	Au_3_Pd_2_/La-CaMgAl-LDH	120	5 (O_2_)	100	100	100	(800)
44	FDMC	HMF	Au/CeO_2_	130	10 (O_2_)	100	>99	>99	(801)
45	FDCA	HMF	Ru/C	110	10 (O_2_)	100	97	97	(802)
46	FDCA	HMF	Ru/MnCo_2_O_4_	120	24 (O_2_)	100	99	99	(803)
47	FDCA	HMF	CuO-MnO_2_-CeO_2_	130	20 (O_2_)	99	99	98	(804)
48	FDCA	HMF	Co_3_O_4_-Mn_0.2_CoO	140	1 (O_2_)	100	>99	>99	(805)
49	FDCA	HMF	CoO_*x*_-mesoporous C	80	5 (O_2_)	98	97	95	(806)
50	FDMC	HMF	Co@CN	80	1 (O_2_)	100	95	95	(807)
51	FDCA	HMF	N-doped nanoporous C	80	n/a (O_2_)	100	80	80	(808)
52	FDCA	Fructose	Pd/CC	100	n/a (O_2_)	91	70	64	(809)
53	FDCA	Fructose	Amberlyst-15, Ru/C	110	40 (O_2_)	n/a	n/a	65	(810)
54	FDCA	Fructose	FDCA, Pt/C	110	40 (O_2_)	n/a	n/a	65	(811)
55	FDCA	Fructose	Amberlyst-15, AuPd/HT	95	n/a (O_2_)	n/a	n/a	78	(812)
56	FDCA	Glucose	CrCl_3_/Amberlyst-15, AuPd/HT	95	n/a (O_2_)	n/a	n/a	50	(812)

Cellulose can be converted to OA in two ways, *via* hydrolysis to glucose followed by nitric acid oxidation, or *via* direct immersion of cellulose in an alkaline solution (Figure 66).^732−734^ A series of catalysts have been used to optimize the second pathway, with a CuO catalyst in aqueous NaOH at 200 °C giving OA with a yield of 41.5%, through several organic intermediates (Table 18, entry 1).^735,736^ The conversion of glycerol to OA was investigated by Xu and co-workers with an Au/CeO_2_ catalyst in aqueous NaOH, giving 55% yield of OA at 98% glycerol conversion (Table 18, entry 2).^737^ Notably, in both the cellulose pathways and the glycerol pathway, the alkali conditions produce oxalate, which must then be acidified to OA, generating large amounts of waste.^731^ A base-free conversion of fructose, glucose, and 5-HMF directly to OA has been achieved by using Fe@CNT and V-Fe@CNT catalysts (Table 18, entries 3–5).^738^ Moderate OA yields (46–49%) were obtained with the only major side products being succinic and formic acid, two other valuable organic acids. The lack of selective and sustainable processes for the hydrolysis of cellulose must be overcome to continue developing carbohydrate-derived OA.^731^

**Figure 67:**
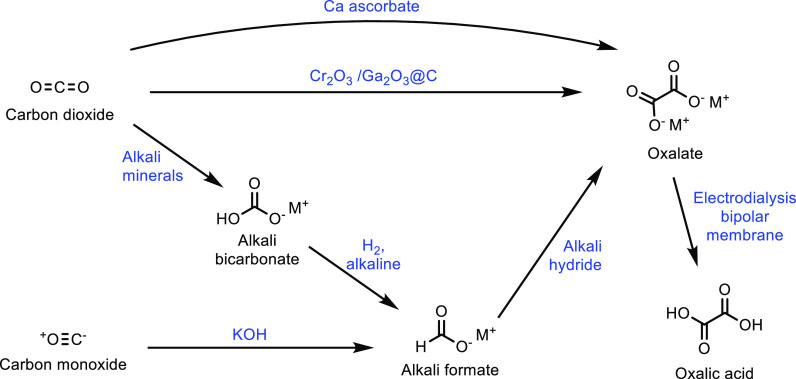
Selection of routes to OA from CO_2_ and CO feedstocks, *via* electrochemical and chemical processes.

CO_2_ and CO are frequently overlooked feedstocks for industrial synthesis and can be converted to OA in multiple ways, as was recently discussed in depth by Schuler *et al*.^728^ Therein, the most sustainable and practical routes identified for near-future commerciality are described briefly (Figure 67). For example, the direct conversion of CO_2_ to OA is possible *via* electrochemical reduction with metals, electrochemical reduction with metal complexes, and reduction with sacrificial calcium ascorbate. In the electrochemical reduction with metals, the absence of water is critical to prevent competing side-reactions and give high OA production.^739−741^ The zinc oxalate process, which utilized a sacrificial zinc anode, benefitted from the absence of side products and precious metal requirements.^742^ Although calculated to be price competitive on a 2 Mt scale, the process is limited by the requirement for dry solvents and has never been tested on a continuous closed-loop pilot scale. Recently, thin films of glassy carbon coated with Cr_2_O_3_ /Ga_2_O_3_ in aqueous conditions gave a 59% yield of oxalate (Figure 67, Table 18, entry 6).^743^

Electrochemical reduction with metal complexes suffers from complex systems, long reaction times, and toxic solvents, but benefits from high selectivity and the possible use of non-precious metals.^744^ A wide range of metal complexes have been studied for OA production from CO_2_, including Ag and Pd porphyrins, Rh-S clusters, and Ir, Co, Sa and Cu complexes.^745−749^ In 2012, Kumar *et al*. utilized a Cu-based MOF and tetrabutylammonium tetrafluoroborate in DMF to give OA with 90%.^750^ Sacrificial calcium ascorbate has been used as a non-toxic stoichiometric reducing agent for the conversion of CO_2_ to OA, but the ascorbic acid precursor is more expensive than the OA product (Figure 67).^553^

Formate-to-oxalate coupling is one of the oldest routes to OA, and the development of formate production from CO_2_ or CO places it as a pragmatic process for OA production.^728,751,752^ Routes to formates include the hydrogenation of carbonates, photochemical reduction and caustic CO reduction.^728^ Currently, alkali formates are commercially produced from CO and caustic alkalis, such as KOH (Figure 67).^753,754^ Alternatively, CO_2_ can be converted to bicarbonate *via* reaction with alkaline metals, and then hydrogenated to formate in an alkaline environment, with Ni-based catalysts found to be the most effective with 77% formate yield.^755^ To avoid the need for external H_2_ supply, processes utilizing reducing agent as hydride donors have been investigated.^756,757^ In a 2016 patent, carbonate salts were converted to formate with yields as high as 99% at 90 °C, using sodium nitrate and Aeroxide P90 TiO_2_ catalyst.^757^ Photocatalytic reduction of CO_2_ to formate has been developed, and presents an attractive route to harnessing solar radiation for the production of valuable chemicals.^728,758,759^ However, current systems are not commercially competitive due to low catalyst stabilities and efficiencies, with a maximum achieved formate yield of 65% when derived from CO_2_, expensive equipment and slow reactions.^759−762^

Formate coupling was found to occur when alkali metal formates are heated to 400 °C in the absence of air or oxygen, with oxalate obtained with a selectivity greater than 70%.^763^ Sodium and potassium formate give high oxalate yields of 91 and 82%, respectively, *via* heating at 390 and 455 °C, respectively.^744,764^ The oxalate yields from sodium and potassium formate were increased to 94 and 99%, respectively, using potassium hydroxide and alkali hydride as catalyst, respectively (Figure 67, Table 18, entry 7).^744^ Potassium formate gave the highest oxalate yield at a much-reduced temperature of 200 °C, and the large catalytic effect highlights the possibility of further reductions in reaction temperature and time *via* the development of new catalyst types.^744^ The conversion of the generated oxalate to OA can be accomplished simple with organic or inorganic acids, or *via* electrodialysis (Figure 67).^765−768^ Electrodialysis bipolar membranes (EDBM), in particular, are effective for producing organic acids *via* water splitting in bipolar membranes. EDBMs are used in multiple industrial processes, including wastewater desalination and lithium battery recycling.^728^ Processes that couple the electrodialysis process with other electrochemical processes are in development and could lead to additional production of high-value chemicals parallel to the salt splitting required to form OA.

##### 5.1.1.2 C3 Diacid

**Figure 68:**
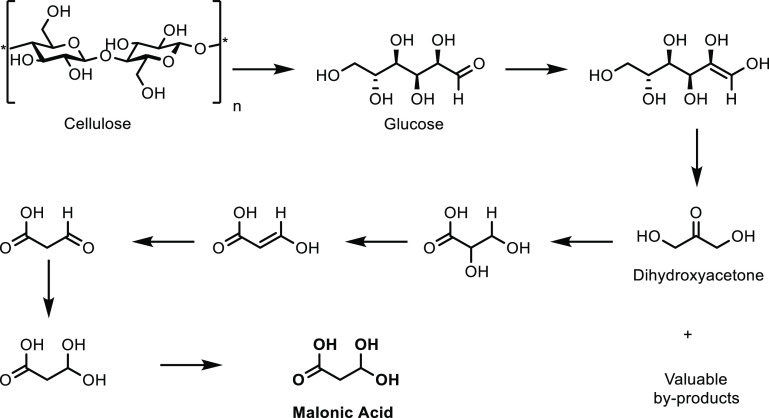
Pathway to malonic acid from cellulose *via* hydrothermal degradation.

Malonic acid (MalonA, 1,3-propanedioic acid) currently has little usage in polymer synthesis, but it could act as a feedstock to 1,3-propanediol synthesis, which in turn is a precursor to polytrimethylene terephthalate.^813^ Very few studies have investigated the synthesis of MalonA from biomass sources, but an article by Yan *et al*. found that hydrothermal degradation of cellulose above 160 °C gave a range of valuable products, one of which being MalonA (Figure 68).^769^ Degradation of a homogenous cellulose solution yielded lactic, acetic and formic acid, and up to 40% MalonA (Table 18, entry 8). An alternative route from malic acid to the more-stable dimethyl malonate (67%) proceeds *via* esterification and oxidative decarboxylation (Table 18, entry 9).^770^ Malic acid is considered a promising sugar-derived building block, although it is currently industrially produced *via* hydration of maleic anhydride (MalAn), which currently is limited in its synthesis from renewable feedstocks.^731^

##### 5.1.1.3 C4 Diacids

Maleic acid (MalA, *cis*-butenedioic acid) is a chemical used in most fields of the chemical industry, in particular within unsaturated polyester resins.^772^ Maleic anhydride (MalAn) is also widely used and the two chemicals can be readily interconverted by hydration or dehydration steps, such that the production of either chemical implies the availability of the other.^731^ The most studied biomass feedstocks for conversion into MalA are furfural and 5-HMF, although 1-butanol and levulinic acid have also been investigated (Figure 65).^777,778,805,814,815^

**Figure 69:**
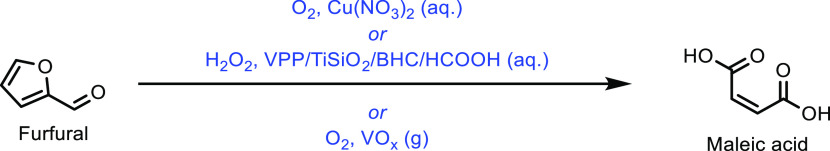
Summary of several reaction conditions for the oxidation of furfural to maleic acid. VPP = Vanadyl pyrophosphate, and BHC = betaine hydrochloride.

Furfural is produced from waste agricultural carbohydrate feedstock on a scale of 250–400 kT annum^–1^ and can be oxidized into MalA in either the liquid or the gas phase (Figure 69).^814,816^ The gas phase oxidation of furfural to MalAn is currently performed with vanadium-oxide based catalysts at temperatures between 200 and 360 °C (Table 18, entry 10).^771^ While the maximum yields reach 90%, the specific structures of the vanadium supports are critical to their catalytic activity. Additionally, furfural is prone to polymerization in O_2_, hence deactivating the catalyst, although this can be minimized by using high temperatures and high O_2_/furfural ratios.^817,818^

With O_2_ as oxidant in the aqueous phase with Cu(NO_3_)_2_, Yin *et al*. reached 86% furfural conversion yet low MalA yield (i.e., 24%).^819^ MalA yield was increased to 34.5% using a biphasic system, with a phosphomolybdic acid catalyst to suppress the furfural polymerization reaction, but tedious separation steps, made the system uncompetitive (Table 18, entry 11).^772^ Ca-doped copper phosphate gave an increased productivity but a low MalA yield of 37% and suffered from significant catalyst deactivation.^814^ Replacing O_2_ with H_2_O_2_ is somewhat less desirable from a sustainability perspective, but enables the oxidation of furfural at milder conditions, although a high ratio of H_2_O_2_/furfural is required to ensure selective formation of MalA.^590,820^ Metal-based and Brønsted-acid catalysts are used for furfural oxidation with H_2_O_2_, and the highest MalA productivity was observed with a heterogenous vanadyl pyrophosphate based catalyst and a low H_2_O_2_/ furfural ratio of 3, albeit with a low MalA yield of 36%.^821^ Titanium silicalite zeolite catalysts produce MalA with a yield of 78% with a H_2_O_2_/furfural ratio of 7.5, but exhibit deactivation due to impurities in the corncob-derived furfural.^822−824^ The deactivation can be alleviated with a γ-valerolactone (GVL)/H_2_O biphasic system, in which the MalA yield starts above 70% and levels off at 45%, even after 21 cycles (Table 18, entry 12).^773^

The deactivation of metal catalysts can be completely avoided using an organic acid, such as the sugar-beet-derived strong acid betaine hydrochloride (BHC).^731^ In aqueous solution, with a H_2_O_2_/furfural ratio of 10, BHC afforded a 61% yield of MalA, as well as a 31% yield of fumaric acid (Table 18, entry 13).^774^ Unlike other strong acids such as HCl, BHC can be recovered and reused without performance degradation. Formic acid can be used as both the solvent and catalyst, yielding 91% MalA with a H_2_O_2_/furfural ratio of 3, although the productivity of the system was low due to peracid formation reducing the solution acidity (Table 18, entry 14).^775,825^ The MalA yield was found to decrease with increasing number of carbons of organic acid used, with butyric acid only yielding 37% MalA.^775^

5-HMF is also reported as a starting material for the liquid-phase conversion to MalA, making use of a heterogeneous vanadium-based catalyst in GVL, due to detrimental effects seen with protic solvents such as water.^815^ The highest MalA selectivity reported (85% at 36% 5-HMF conversion) utilized Fe_3_O_4_@SiO_2_ as catalyst (Table 18, entry 15).^776^ The aforementioned formic acid procedure, repeated with 5-HMF, gave the best overall results, with 89% yield of MalA and a productivity more than twice that of the previous method (Table 18, entry 16).^775^ The gas-phase conversions of 1-butanol and levulinic acid (LevA) to MalAn have also been reported.^777,778^ Cavani *et al*. used vanadyl pyrophosphate to produce MalAn with a 39% yield from 1-butanol in air, but the system productivity was low and high purity 1-butanol was required (Table 18, entry 17).^777^ Improvements in feedstock purity and robust catalysts are desirable to expand the potential of the oxidation reaction. A VO_*x*_/SiO_2_ catalyst yielded MalAn with a yield of 71% from LevA, although the low melting point, high viscosity and low thermal stability of LevA are potential barriers to continuous industrial production (Table 18, entry 18).^778^

**Figure 70:**
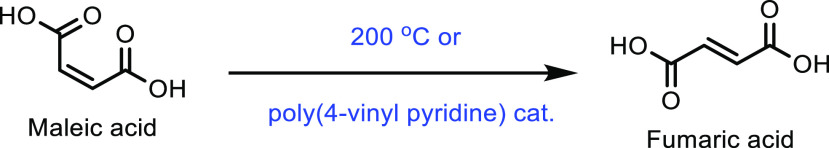
Isomerization of maleic acid to fumaric acid, at elevated temperatures, with a thiourea catalyst or with a poly(4-vinylpyridine) catalyst.

Fumaric acid (FA, *trans*-butanedioic acid) is used in the food industry, for feed processing and in the synthesis of unsaturated polyesters.^826^ The current methods for FA synthesis are primarily isomerization from MA and fermentation, although there are few relevant studies.^774,827^ During the conversion of furfural to MA, FA is produced as a side-product resulting from the MA double bond *cis*-*trans* isomerization (Figure 70).^774^ Current industrial production uses a homogeneous thiourea catalyst, but a poly(4-vinylpyridine) resin has been presented as a heterologous alternative (Table 18, entry 20).^827^ FA yields of 78% from MA have been obtained, but decreased significantly when initial concentrations of MA above 0.02 wt % were used, and catalyst deactivation was quickly observed. Without catalyst, the reaction proceeds readily at 200 °C, but this causes a color change, which can be considered disadvantageous for some applications (Table 18, entry 19).^779^

Succinic acid (SA, 1,4-butanedioic acid) is widely used in the food, chemical and pharmaceutical industries. Additionally, it is an important monomer for the preparation of biodegradable polymers, such as polybutylene succinate.^828−830^ SA is produced predominantly *via* the hydrogenation of petrochemically derived maleic anhydride, but is also commercially produced from biomass *via* a fermentative process, albeit generating significant Ca_2_SO_4_ waste during purification and thus decreasing SA productivity.^831,832^ Hence, it is worthwhile exploring recent developments in the synthesis of SA from biomass, *via* both biotechnological and catalytic routes.

**Figure 71:**
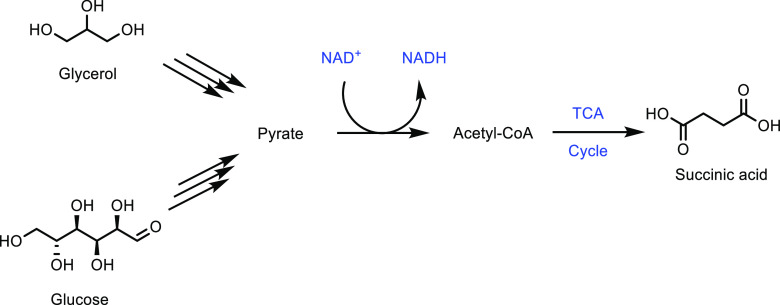
Biotechnological production of succinic acid from feedstocks including glucose and glycerol, *via* deletion of a succinate-metabolizing gene in the tricarboxylic acid (TCA) cycle.

An excellent review by Li *et al*. has recently discussed advances in biotechnological routes to prepare SA from renewable feedstocks.^833^ Typically, yeasts are more well-suited to fermentative SA production due to their abilities to use alternative feedstocks in a variety of adverse conditions. Current commercial routes typically use *E. coli* and glucose feedstocks. With an engineered *Y. lipolytica* strain, the highest-obtained SA titer, i.e., 209.7 g L^–1^, was achieved from a crude glycerol feedstock, with a yield of 0.45 g g^–1^ (Table 19, entry 1).^834−836^*Y. lipolytica* has been used to obtain a lower but nevertheless significant titer of 33.2 g L^–1^ SA from glucose, and can also utilize a range of additional feedstocks for SA production, including ethanol, rapeseed oil and even *n*-alkanes (Table 20, entry 2).^837−840^ The metabolic engineering employs strategies such as the blocking of a step within the TCA cycle, preventing the desired product from being further converted in the metabolic cycle (Figure 71).

**Table 19:** Microorganisms, Conditions and Yields for the Chemical Production of Selected Products from Biomass Sources *via* Biotechnological Conversion

Entry	Class	Product	Feedstock	Microorganism	Titer (g L^–1^)	Yield (mol %)	Mass yield (g g^–1^_feedstock_)	Ref.
1	Diacid	Succinic acid	Glycerol	*Y. lipolytica*	210	71	0.45	(834)
2	Diacid	Succinic acid	Glucose/Xylose	*Y. lipolytica*	33	n/a	0.58	(837)
3	Diacid	Adipic acid	Glucose	*T. fusca*	2.2	n/a	0.045	(850)
4	Diacid	Adipic acid	Glycerol	*E. coli*	68	73	0.38	(851)
5	Diacid	Adipic acid	Fatty acids	*C. tropicalis*	50	n/a	n/a	(547)
6	Diol	Ethylene glycol	Xylose	*E. coli*	108	87	0.36	(852)
7	Diol	Ethylene glycol	Xylose	*E. coli*	40	85	0.35	(853)
8	Diol	1,3-PDO	Glycerol	*K. oxytoca*	76	n/a	n/a	(854)
9	Diol	1,3-PDO	Glucose	*E. coli*	135	n/a	n/a	(855)
10	Diol	1,4-BDO	Glucose	*E. coli*	120	80	0.40	(597)
11	Diol	1,4-BDO	Xylose	*E. coli*	12	43	0.37	(856)
12	Diol	2,3-BDO	Glucose	*K. pneumoniae*	150	n/a	n/a	(857)
13	Diol	1,3-BDO	Glucose	*E. coli*	13	0.57	0.29	(858)
14	Diamine	1,3-PDA	l-Aspartate	*E. coli*	13	n/a	0.1	(859)
15	Diamine	1,4-BDA	Glucose	*E. coli*	42	n/a	0.26	(860)
16	Diamine	1,5-PeDA	l-Lysine	*E. coli*	221	92	47	(861)
17	Diamine	1,5-PeDA	Glucose	*C. glutamicum*	104	54	30	(862)
18	Diamine	1,5-PeDA	Xylose	*C. glutamicum*	103	32	22	(863)

**Table 20:** Catalysts, Conditions and Yields for the Production of Selected Diols from Biomass Sources *via* Chemical Conversion

Entry	Diol	Feedstock	Catalyst	Temperature (°C)	Pressure (bar)	Conversion (%)	Selectivity (%)	Yield^a^ (%)	Ref.
1	EG	Cellulose	Al-WO_3_-Ni-TUD-1	230	40 (H_2_)	100	76	76	(1044)
2	EG	Cellulose	Ni-W/SBA-15	245	60 (H_2_)	100	76	76	(1045)
3	EG	Cellulose	Ru/WO_3_	240	40 (H_2_)	100	76	76	(1046)
4	EG	Pre-treated lignocell.	Ni-W/MCM-41	250	60 (H_2_)	n/a	n/a	64	(1018)
5	EG	Pre-treated lignocell.	Ru/C + m-WO_3_	245	60 (H_2_)	100	63	63	(1019)
6	EG	Pre-treated lignocell.	RANEY Ni + SPT	245	60 (H_2_)	n/a	n/a	35	(1020)
7	EG	Glucose	Ni-W_2_C/AC	245	60 (H_2_)	n/a	n/a	35	(1023)
8	EG	Glucose	Ni-W_2_C/AC	245	60 (H_2_)	100	47	47	(1023)
9	EG	Glucose	Ru/AC + AMT	240	50 (H_2_)	n/a	n/a	60	(1024)
10	EG	Lignocell. hydrolysate	RANEY Ni + NaOH	230	110 (H_2_)	n/a	n/a	19	(1025)
11	EG	Glycerol	Cu/ZnO/MO_*x*_	300	1 (H_2_)	100	37	37	(1028)
12	EG	Glucose	1) n/a	1) 527	1) n/a	n/a	n/a	98	(1036)
			2) n/a	2) 230	2) Cu/C				
			3) Ru/C	3) 80	3) 90 (H_2_)				
13	1,2-PDO	Glycerol	Ru-Cu/modified bentonite	230	80 (H_2_)	100	85	85	(1029)
14	1,2-PDO	Glucose	1) Ca(OH)_2_ + CuCr	1) 140	1) 60 (H_2_)	100	53	53	(1047)
			2) Ca(OH)_2_ + CuCr	2) 220	2) 60 (H_2_)				
15	1,2-PDO	Cellulose	Ca(OH)_2_ + CuCr	245	60 (H_2_)	100	43	43	(1048)
16	1,2-PDO	Lignocellulose	Ni-W_2_C/AC	245	60 (H_2_)	n/a	n/a	39	(1049)
17	1,2-PDO	Lignocell. Hydrolysates	RANEY Ni + NaOH	230	110 (H_2_)	n/a	n/a	41^b^	(1025)
18	1,3-PDO	Glycerol	Pt/WO_*x*_/AlOOH	180	50 (H_2_)	100	66	66	(1050)
19	1,3-PDO	Glycerol	Pt-Cu/Zeolite	210	1 (H_2_)	90	59	53	(1051)
20	1,4-BDO	Dimethyl maleate	Cu/SBA-H	220	50 (H_2_)	100	66	66	(1052)
21	1,4-BDO	SA	Re-Pd/C	160	150 (H_2_)	100	66	66	(1053)
22	1,4-BDO	Furfural	Pt/TiO_2_-ZrO_2_	120	35 (H_2_)	1) 96	1) 94	85	(1054)
						2) 97	2) 98		
23	1,5-PeDO	Furfural	1) Ni	1) n/a	1) n/a	n/a	n/a	80	(1055)
			2) γ-Al_2_O_3_	2) 375	2)) n/a				
			3) nr	3) 130	3) 17 (He)				
			4) 45 (H_2_)	4) Ru-TiO_2_	4) 120				
24	1,6-HDO	HMF	Pd/SiO_2_ + Ir-ReO_2_/SiO_2_	100	70 (H_2_)	100	58	58	(1056)
25	1,6-HDO	THFDM	PtWO_*x*_/ TiO_2_	160	55 (H_2_)	n/a	n/a	83	(1057)
26	1,6-HDO	Cellulose	1) H_2_SO_4_	1) 210	1) 69 (He)	n/a	n/a	27	(1055)
			2) Ni/SiO_2_	2) 150	2) 34.5 (H_2_)				
			3) Pd/SiO_2_-Al_2_O_3_	3) 160	3) 55 (H_2_)				

a Molar yield of target diol.

b Mass yield of target diol.

**Figure 72:**
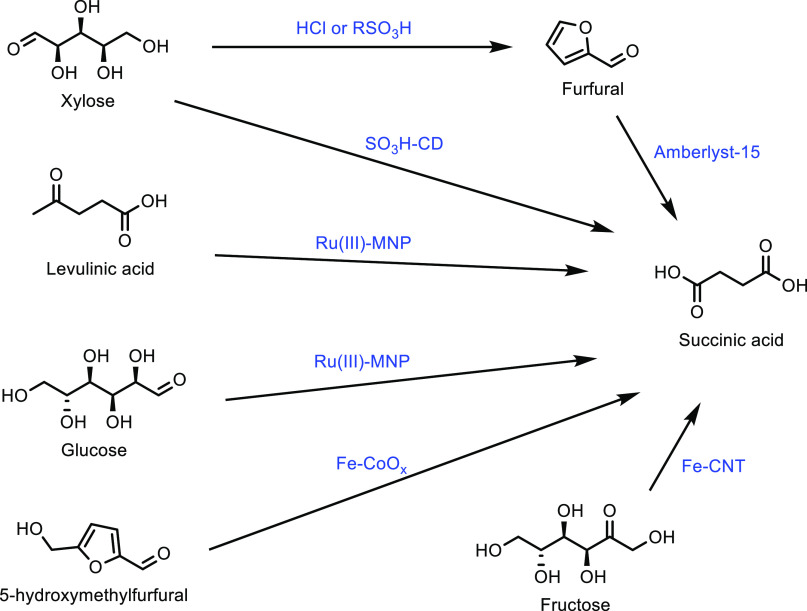
Chemical routes to succinic acid from a range of biomass sources.

Chemically, xylose can be converted into SA using Brønsted acid catalysts directly or indirectly *via* furfural (Figure 72).^590,820,841,842^ Indirectly, xylose can be converted to furfural using Brønsted acids, either supported on metal–organic frameworks (MOFs) or complimented by Lewis acids such as CrCl_3_, with SA yields exceeding 70%.^842,843^ Conversion of furfural to SA with the Brønsted acid Amberylst-15 resin in aqueous H_2_O_2_ gave a 74% SA yield at full conversion, attributed to the interactions of the Amberlyst-15 aromatic ring with the furfural furan ring.^590,820,844^ Direct conversion of xylose to SA resulted in numerous side-reactions, including furfural polymerization, unless a toluene/H_2_O biphasic system was used, in which case a 52% SA yield.^841^ A SO_3_H-CD-carbon catalyst with ultrasound irradiation increased the SA yield to 81% due to the increased interfacial area and increased mass and heat transfer (Table 18, entry 21).^780^ SA yields of up to 93% were obtained using a N-doped SO_3_H-carbocatalyst, although this catalyst suffered serious deactivation due to acid site leaching.^845^

The conventional process to SA from LevA utilizes hazardous nitric acid or mercury.^846,847^ A yield of 78% SA was obtained from LevA using 4 wt % ruthenium on silica magnetic nanoparticles (Ru^III^-MNP) in the presence of H_2_O_2_, which were easily separated using magnetism and reused with negligible Ru leaching, and further investigation with concentrated LevA and lower catalyst loading would be useful to evaluate the industrial potential of this route (Figure 72, Table 18, entry 22).^781^ To avoid metal-leaching, trifluoroacetic acid was used to convert LevA into SA in the presence of H_2_O_2_, yielding 60% SA after separation (Table 18, entry 23).^782^ The employed acid could potentially be recycled *via* dehydrating the azeotrope.^848,849^

Glucose was converted to SA with the aforementioned Ru^III^-MNP catalyst, achieving SA yields of 62% at full conversion (Figure 72).^864^ A SA selectivity of 88% could be maintained after multiple runs *via* the continuous addition of *n*-butylamine. Ru-oxyhydride nanoparticles on N-doped graphene under similar conditions gave 87% SA from glucose, but with a low productivity (Table 18, entry 24).^783^ A high SA selectivity of 81% was achieved from 5-HMF at 90% conversion in the presence of NaOH using a silica-immobilized Fe/Co-oxide based catalyst (Figure 72, Table 18, entry 25).^776^ Changing the base to *n*-butylamine increased the conversion but decreased the productivity, which was proportional to the basicity of the reaction medium. Dibendetto and co-workers used Fe-CNT catalysts for the production of OA from fructose, with SA produced as a side-product (Figure 72, Table 18, entry 26).^738^ Although initially high, the SA selectivity dropped to 21% by the time 99% fructose conversion was reached. Of the available feedstocks, monosaccharides such as glucose and fructose are among the most cost effective, but improvements to the reported catalysts are required before commercial-scale production could become viable.^731^

##### 5.1.1.4 C6 Diacid

**Figure 73:**
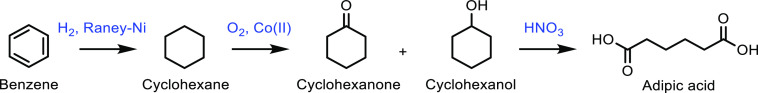
Dominant petrochemical route to adipic acid *via* the nitric acid oxidation process.

Adipic acid (AA, 1,6-hexanedioic acid) is produced on a scale of >3 MT annum^–1^, primarily for use in nylon 66, the most commonly used polyamide.^865^ Currently, adipic acid is mainly made *via* the nitric acid oxidation process.^865,866^ In this process, petrochemical benzene is reduced to cyclohexane, converted over a Co(II) catalyst to a mixture of cyclohexanone and cyclohexanol, called KA oil, and eventually oxidized to adipic acid with nitric acid (Figure 73).^866^ During the conversion, 90% of the unreacted cyclohexane must be recycled to prevent over-oxidation. A similar route utilizing cyclohexene is also used, although nitric acid is still required, generating potent NO_*x*_ greenhouse gases.^867,868^ Currently, NO_*x*_ released from AA production constitutes 20% of the CO_2_-equivalent emissions of the entire petrochemical production industry, as the warming potential of NO_*x*_ is ∼300 times greater than CO_2_.^869−871^ Despite technologies to reduce NO_*x*_ emissions, such as flue gas recirculation, many plants operate without recycling policies in place.^872−875^ Despite being extremely inefficient, the NO process will remain the major route until more-sustainable routes can be scaled at a similar cost.^865,872,876^

One route to NO_*x*_ free AA production is using alternative oxidants, such as H_2_O_2_ and O_2_.^872,876−878^ Several studies show high yields of AA obtained from cyclohexene with H_2_O_2_, on a large scale,^872,877,879−883^ as well as from cyclohexanone in water with ferrous catalyst and H_2_O_2_.^884^ However, life cycle analysis of H_2_O_2_ raises even greater concerns than NO, due to the rather polluting “anthraquinone process” used for its production.^865,880,885^ Air and O_2_ are also used widely as alternative oxidants.^865^ A commercialized route utilizes air for the conversion of cyclohexane to cyclohexanol/cyclohexanone, which is then combined with acetic acid to give AA with a yield of 50–70%, albeit with significant side products.^872,886−888^ However, the high required AA purity means the acetic acid must be completely removed.^787,872^ Currently, air/O_2_ oxidation routes are uncompetitive due to a lack of adequate catalysts.^865^

**Figure 74:**
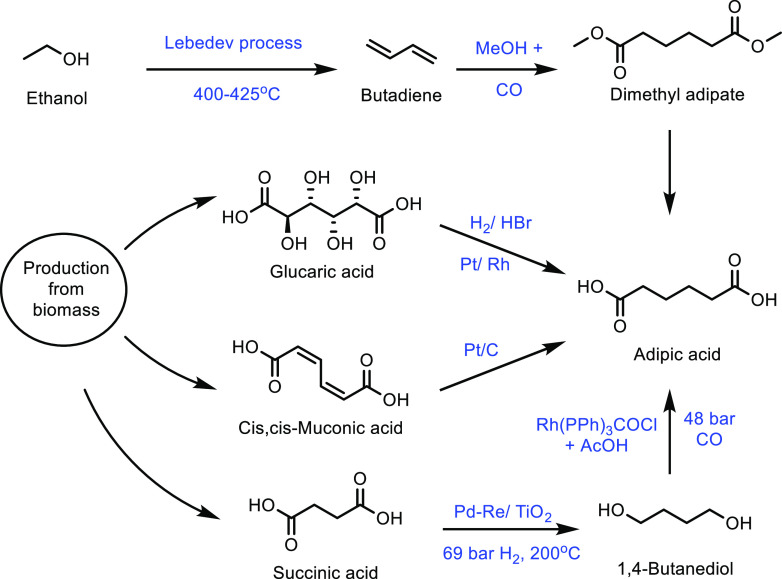
Methods for bio-based adipic acid production from ethanol, glucaric acid, *cis*,*cis*-muconic acid and succinic acid *via* chemical conversions.

An alternative route to NO_*x*_ free AA is to change the feedstock. Several substrates have been explored, including phenol, butadiene, adiponitrile and cyclohexene.^872,876,886^ However, these are still derived from benzene, and the phenol-based processes still generates NO_*x*_.^872,889^ Butadiene is the most promising alternative, which can be converted with MeOH and CO (syngas) to dimethyl adipate with yields as high as 96%, and subsequently converted to adipic acid without producing NO_*x*_ (Figure 74, Table 18, entry 27).^784,890,891^ Assuming some optimizations, this process could potentially compete with the NO process, but an unstable butadiene price has prevented adoption of this technology.^865^ Butadiene can be produced from sustainable biomass *via* several routes, the most prominent of which is the direct conversion from bio-sourced ethanol.^665,892,893^ Bio-ethanol derived butadiene would be 13–26% more expensive than conventional butadiene, but this difference would likely decrease as the catalysts are improved and the petrochemical feedstocks become less abundant. While the bioethanol route to butadiene avoids petrochemical consumption and results in significant reductions in CO_2_ emissions, the energy demand and water consumption of the route are substantially higher, increased by factors of ∼100 and ∼2, respectively.^892^

**Figure 75:**
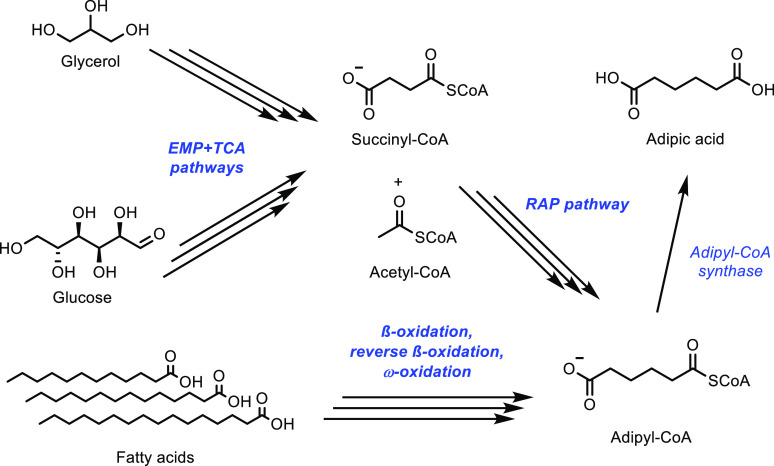
Direct biotechnological routes to adipic acid from glycerol, glucose and fatty acids, with the multiple steps of the tricarboxylic acid cycle (TCA), the glycolysis pathway (EMP), the reverse adipate pathway (RAP), the β-oxidation cycle, the reverse β-oxidation cycle and the ω-oxidation cycles simplified into single steps.

Enzymatic routes and combinations of enzymatic and conventional chemical routes provide additional pathways to bio-based AA. AA only appears as a metabolic intermediate at trace levels, so other routes have been explored.^850,894−898^ The reverse adipate pathways could theoretically produce AA from glucose with molar yields as high as 92%, although only a titer of 2.2 g L^–1^ has been obtained from glucose with an engineered *Thermobifida fusca* strain (Figure 75, Table 19, entry 3).^850,889,899,900^ Significantly higher yields have been achieved using glycerol, with an engineered strain of *E. coli* giving a titer of 68 g L^–1^ AA with a yield of 0.34 g g^–1^ (Figure 75, Table 19, entry 4).^900^

Oxidative pathways containing a mixture of β-oxidation, reverse β-oxidation and ω-oxidation processes enable the production of AA from a range of renewable substrates, including glycerol, glucose and fatty acids (Figure 75).^865,889,894,901,902^ While *E. coli*. has only yielded AA titers below 1 g L^–1^ from glycerol, significantly higher titers have been obtained from engineered yeasts.^865,901^ By disrupting the enzymes responsible for degrading C6 acyl chains, fatty acids are degraded to adipic acid but no further. Using this method, Picataggio and coworkers modified *C. tropicalis* to yield ∼50 g L^–1^ AA from coconut oil. This has been scaled to 300 L, although this commercial effort was halted in 2018 (Table 19, entry 5).^547,903−907^ Neither glycerol nor vegetable oil sources are economically competitive with NO, as waste glycerol is unsuitable for the process due to impurities and insufficient supply.^865^ Additional pathways such as the 2-oxopimelic acid pathway, the polyketide synthase pathway, and the lysine conversion pathways have been proposed, with only the first two demonstrated, both of which gave titers of <0.5 g L^–1^.^889,898,908^

Several indirect routes combining the production of an intermediate *via* chemical or fermentative processes with the biotechnological conversion to AA have been developed. The most-studied intermediates are succinic acid (SA), glucaric acid (GA) and *cis,cis-*muconic acid (MucA), with their conversions to AA shown in Figure 74.^865,889,894,896,902,909^ All three intermediates are naturally occurring and have additional potential as platform chemicals outside of AA synthesis.^895,910−914^ The first important intermediate, SA, can be converted to AA *via* hydrogenation to 1,4-butanediol followed by catalytic carboxylation, with the second step yielding 74% AA using a Rh(PPh)_3_COCl catalyst (Table 18, entry 28).^785,915^ Although scaled up to a pilot-plant, the process has not yet been commercialized due to the energy intensive conversion.^865^ Other biotechnological routes directly to 1,4-butanediol from sugars have been developed, which could be combined with the aforementioned routes to produce AA.^916^

**Figure 76:**
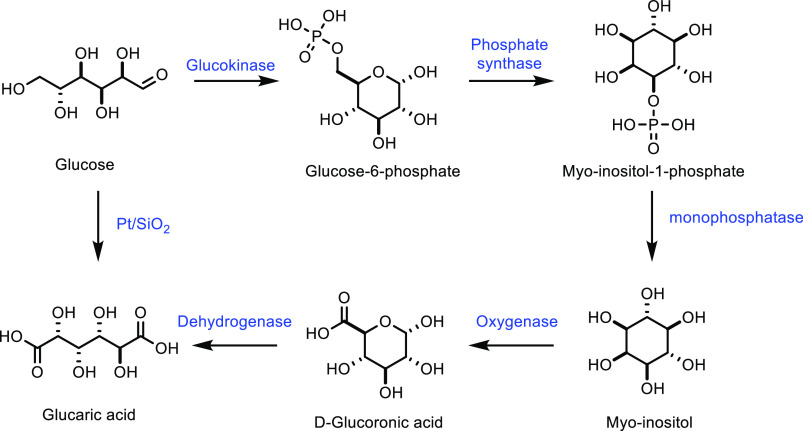
Chemical and biotechnological routes from glucose to glucaric acid.

The second important intermediate, GA, is a C6 dicarboxylic acid with a hydroxy group on each of its 4 internal carbons and has uses within the biomedical industry and within polymer chemistry.^910,914,917^ A 3-step pathway from glucose to GA has been developed, and *Saccharomyces cerevisiae* was used to give a titer of 6 g L^–1^ GA after 200 h (Figure 76).^910,918−921^ A slight improvement to 6.6 g L^–1^ GA after 80 h was obtained through co-feeding an engineered *Pichia pastoris* strain glucose and myo-inositol.^922^ A 2-step chemo-catalytic route from glucose to AA, *via* GA, has also been developed (Figure 76), with the second step applicable to GA obtained from enzymatic pathways.^896^ The oxidation and subsequent hydrodeoxygenation process has been piloted with AA yields of up to 89%, although significant amounts of base are required for the glucose oxidation, and improved catalyst longevity is desirable (Table 18, entry 29).^260,786,865,896,923−925^

**Figure 77:**
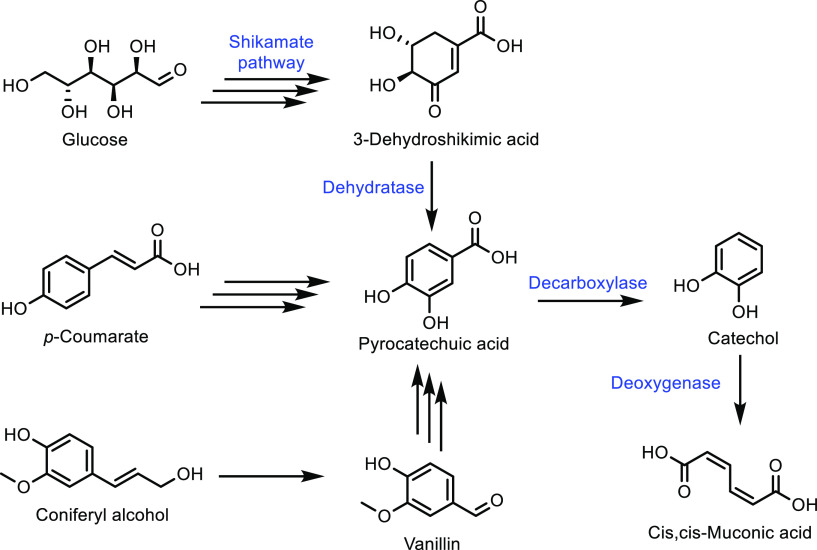
Biotechnological routes to *cis*,*cis*-muconic acid from glucose and lignin-derived aromatic compounds such as *p*-coumarate, coniferyl alcohol and vanillin.

The third important intermediate (MucA) is a natural intermediate produced in several microbes, and can be converted to AA *via* hydrogenation using various catalysts such as Pt/C, with yields up to 97% (Figure 77, Table 18, entry 30).^787,889,926−928^ MucA can also be isomerized to give *cis*,*trans*- and *trans*,*trans*-muconic acid, which have applications as a precursor for the synthesis of terephthalic acid and ε-caprolactam.^787,889,894,896,913^ The shikimate pathway has been engineered and optimized for MucA production from glucose, with the highest production of 59.2 g L^–1^ MucA observed by Frost *et al*. in an engineered strain of *E. coli*, with a molar yield of 30%.^889,909,913,926,929−932^ Lignin-derived aromatic compounds such as *p*-coumarate, ferulate and benzoate have been utilized for biotechnological MucA production with glucose as a co-substrate in *P. putida*, with MucA titers reaching 50 g L^–1^, beyond which the MucA toxicity becomes lethal.^911,933^ To overcome the issue of toxicity limiting product titers, expression of these pathways in a more tolerant organism, i.e., *S. cerevisiae*, has been engineered, albeit only with low titers (140 mg L^–1^).^931^ An Iron(III) catalyst was used for the chemical conversion of lignin-derived catechol into MucA using benign oxidants, but the reaction rate was low.^934^ Alternatively, an organic salt catalyst with H_2_O_2_ as an oxidant was used to give yields of up to 84%.^935^ Recently, also the enzymatic reduction of MucA to AA has been described, and combining both the synthesis of MucA and subsequent conversion to AA would enable the direct conversion of renewable feedstocks to AA *via* MucA in a single microorganism.^936,937^

The purity demands for AA are high, and currently no method can competently remove acidic and other organic impurities to below the 20 ppm threshold required for use within nylon synthesis.^865^ Attempted methods include utilizing the solubility of MucA in ethanol, reactive extraction and the utilization of deep eutectic solvents for extraction, but the purest MucA still contains 90 ppm of an elemental nitrogen impurity.^787,938−941^ Purification is a massive hurdle for widespread adoption and more advanced routes from diverse bio-feedstocks will face even greater impurities that require removal.^865^ This issue is not unique to bio-based feedstocks, as alternative petrochemical routes to AA have also been hindered by the purity requirement.^942^ The three most promising routes to bio-based AA are the chemical conversion of butadiene, the chemical oxidation of glucose *via* GA, and the biotechnological reverse adipate pathway.^889^ All three routes have potential for commercialization in the near future. Additionally, economic analysis of the biotechnological pathway *via* MucA suggested a roughly 2-fold cost reduction relative to the NO process, if lignin is used as a feedstock.^889^ This is due to an extremely cheap estimation of lignin, as low as $0.04 kg^–1^, which would require significant additional progress in the area of lignin purification and valorisation.^943,944^ As such, this route is still in its infancy, but arguably presents the best-case-scenario in the long term for AA synthesis.

##### 5.1.1.5 Long-Chain Diacids

Several long-chain diacids have been produced both chemically and biotechnologically, although these are typically produced and utilized on scales much smaller than shorter chain diacids. In step-growth polymerization, utilizing longer monomers can increase chemical resistance due to a subsequent decrease in the concentration of vulnerable bonds such as esters and amides. Additionally, these monomers enable greater variation in the thermal and mechanical properties of polymers produced, although these also greatly depend on the nature of the polymer chains.

Pimelic acid (PimA, 1,7-heptanedioic acid) is commercially produced *via* the oxidation of cycloheptanone with dinitrogen tetroxide and a number of patents exist relating to the its production *via* various routes.^945−947^ PimA was first identified as a metabolic intermediate in bacterial and yeast growth in 1964, and in 2011 Zhang *et al*. achieved a pimelic acid titer of 2.36 mg L^–1^ using an engineered bacterial strain.^948^ This exceptionally low titer highlights the lack of development in this area.

Suberic acid (SubA, 1,8-octanedioic acid) production from bio-based sources has not been the subject of many studies, although recently SubA has been produced with a yield of 13% from hydroxy cyclooctane *via* an enzymatic process using *E. coli*.^949^ A 2014 patent for the production of SubA from γ-valerolactone and an alcohol follows a 4-step procedure.^950^ Due to low monomer availability, SubA has not seen many applications within polymer chemistry, but the development of an efficient route to SubA from biomass sources could enable a range of novel polymers with interesting properties.

Azelaic acid (AzA, 1,9-nonanedioic acid) is produced from oleic acid, an unsaturated 18C fatty acid, *via* oxidative cleavage of the double bond. Oxidative cleavage is predominantly conducted *via* ozonolysis, which presents concerns regarding flammability and toxicity.^951^ Hydrogen peroxide present a valid alternative, due to high atom economy and reduced safety concerns compared to gaseous reactants.^952−956^ The process can either proceed in two steps or, more recently, in a single step, although the latter can require high catalyst loadings.^954−957^ Benessere *et al*. reported a method for both a two-step and a single-step process.^957^ In the two-step process, tungstic acid and H_2_O_2_ at 343 K convert oleic acid to a 9,10-dihydroxystearic acid intermediate, which was then fully converted to AzA by NaOCl at 25 °C with a maximum overall yield of 54%. In the one-step process, tungstic acid and H_2_O_2_ alone gave a high AzA yield of 91%, albeit with an increased ratio of H_2_O_2_ to oleic acid, increased reaction temperature (i.e., 373 K) and extended reaction time (i.e., 8 h).

Sebacic acid (SebA, 1,10-decanedioic acid) is mainly produced industrially *via* alkali saponification or catalytic hydrolysis of castor oil to ricinoleic acid, and subsequent alkali cracking, acidification, and purification.^958^ Biotechnological routes to SebA have been investigated for production from bio-based sources with reasonably productive results, as is discussed in a recent review by Li *et al*.^959^ An engineered strain of *E. coli* has been used to convert natural fats to SebA with yields reaching 50–60%.^960^ Suigharto *et al*. utilized the biotechnological transformation of methyl decanoate to produce SebA with a yield of 27 g mol^–1^ and a productivity of 0.5 g L^–1^ h^–1^.^961^ From glycerol, a readily available sustainable feedstock, a 0.06 g L^–1^ titer of SebA has been achieved, *via* β- and ω-oxidation.^901,962^ An engineered *C. tropicalis* was used to convert decane into SebA with a titer of 0.94 g L^–1^ h^–1^, which has potential for future sustainability due to the possibility of alkane production by enzymatic processes.^963,964^

**Figure 78:**
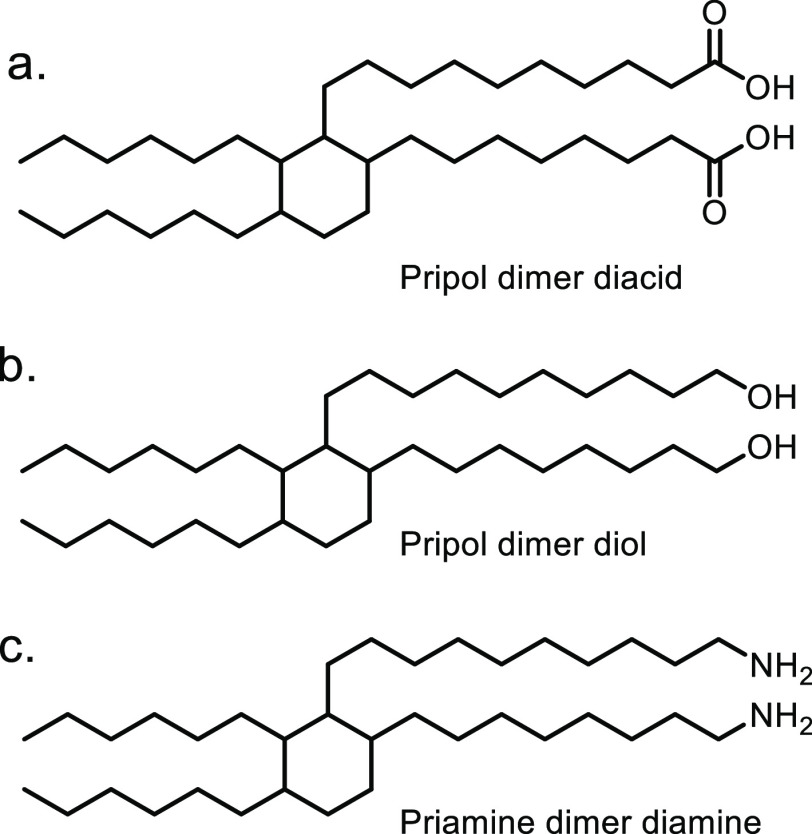
Long-chain C36 diacid, diol and diamines commercialized by Croda (approximate structures with omitted double bonds along the chains).

Longer chain α,ω-diacids can be produced catalytically *via* the metathesis of unsaturated fatty acids or biotechnologically *via* conversion of various renewable feedstocks.^965,966^ The self-catalysis of a number of unsaturated fatty acid and their ester derivatives with Ru-based Grubbs catalysts yielded a series of straight chain C18–C26 α,ω-dicarboxylic acids and esters, with yields ranging from 39% to 82%.^966^ However, these reactions suffer from harsh conditions, multi-step processes, and costly separations. Lee *et al*. reviewed the current state of long-chain diacid production from vegetable-oil sources *via* enzymatic processes, which avoids several of the aforementioned metathesis issues.^965^ Alkanes are currently the most effective substrate for conversion to long-chain α,ω-diacids *via* α,ω-oxidation, although their current petrochemical source limits their potential as a sustainable feedstock.^963,965^ Fatty acids can be converted into α,ω-diacids *via* three metabolic processes, where the ω-carbon is sequentially oxidized to form a ω-hydroxy fatty acid, a ω-oxo-fatty acid and finally an α,ω-diacid.^965^ The suppression of β-oxidation pathways and the overexpression of ω-oxidation pathways are identified as strategies for increased α,ω-diacid productions.^965^ Croda has patented the production of a C36 diacid obtained from the dimerization of unsaturated oleic and/or linoleic acids *via* Diels–Alder coupling, or a similar process (Figure 78).^967^ The non-terminal nature of the unsaturation in these fatty acids gives two alkyl branches. Whereas straight-chain long-chain aliphatic diacids are typically solids that increase the melting and glass transition points of polymers they are incorporated in, the C36 diacid is a liquid at room temperature, and reduces the glass transition and melting points of polymers, due to the long alkyl branches interrupting the polymer chain packing.

##### 5.1.1.6 Aromatic Diacids

**Figure 79:**
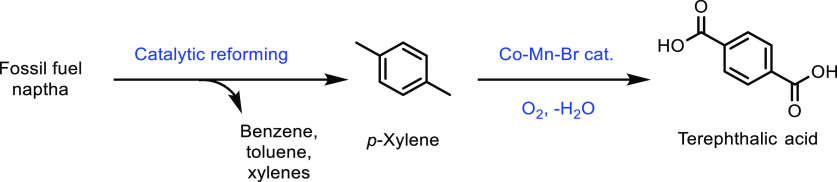
Conventional production of terephthalic acid from fossil fuel naphtha, *viap*-xylene.

Terephthalic acid (TPA) is produced on a massive scale exceeding 80 MT annually, primarily for the production of polyethylene terephthalate (PET) and some other polyesters.^968^ Currently, the vast majority of TPA is produced *via* the oxidation of *p-*xylene, which is obtained *via* catalytic reforming of petrochemical naphtha (Figure 79). The reforming process gives a mixture of aromatic compounds called BTX, largely consisting of benzene, toluene and xylene isomers.^731^ Producing TPA from bio-based sources either involves the production of bio-based *p*-xylene for conventional conversion to TPA, or direct production of TPA from bio-based sources.

**Figure 80:**
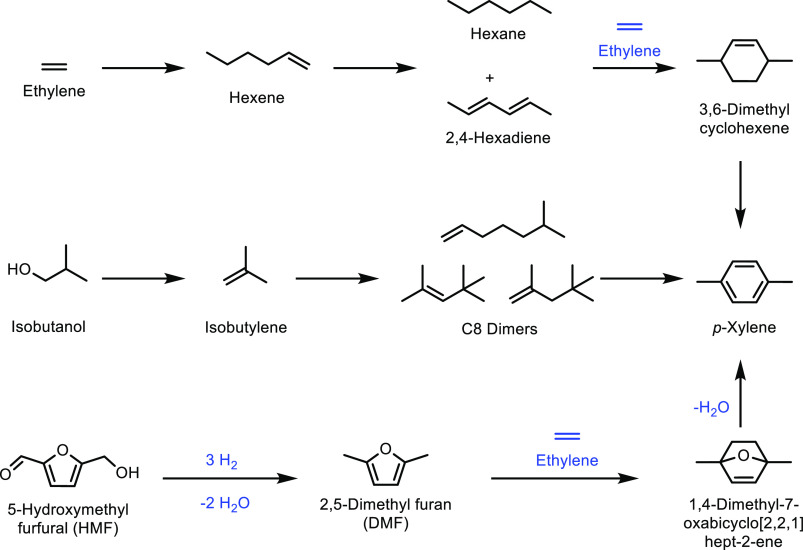
Established routes to bio-based *p*-xylene from ethylene, *iso*-butanol and 5-hydroxymethyl furfural (HMF).

Several possible bio-based routes to *p*-xylene have emerged from feedstocks including furan, 2-methylfuran, ethylene, *iso*-butanol, 2,5-dimethanylfuran and HMF, as shown in Figure 80.^731^ Catalytic fast pyrolysis (CFP) is a process that produces BTX from solid raw biomass with an inexpensive zeolite catalyst *via* Diels–Alder reactions with alkenes.^969−971^ Following the fine-tuning of the pore structure of a coked ZSM-catalyst, the total *p*-xylene selectivity of the CFP of furan and 2MF with propylene reached 1.8 and 15%, respectively.^971−973^

Ethylene, which is commercially derived from sugarcane and corn (section 2.1), can be converted to *p*-xylene *via* a four-step process (Figure 80).^114^ The ethylene undergoes trimerization to hexene, followed by disproportionation to hexane and 2,4-hexadiene, and a Diels–Alder reaction of 2,4-hexadiene with ethylene to give 3,6-dimethylcyclohexene. The latter is dehydrated to *p*-xylene. The disproportionation shows poor 2,4-hexadiene selectivity, but an iridium-based catalyst at 180 °C achieved a TON of 777 over 3.5 h (Table 18, entry 31).^788^ A one-pot diene formation and Diels–Alder cyclization achieved 66% 3,6-dimethylcyclohexene selectivity, and dehydrogenation with Pt/Al_2_O_3_ yielded 93% *p*-xylene, giving an overall yield of 89%. While bio-ethylene is expected to become cheaper, significant research is required to increase the reaction efficiency and use heterogeneous catalysts.

Isobutanol, which can be produced *via* starch fermentation, has been studied as a feedstock for *p*-xylene production and commercialized by Gevo Inc.^789,974^ Isobutanol is dehydrated to isobutylene with a BASF-AL3996 catalyst at 350 °C, oligomerized to di-isobutylene with a ZSM-5 catalyst, and converted to *p*-xylene with a CrO_*x*_-doped-Al_2_O_3_ catalyst (Figure 80, Table 18, entry 32). The respective yields of the three steps are 95, 89 and 75%, giving an overall total yield of 63%. Starch is an expensive feedstock of isobutanol, constituting 46% of the operating cost, and the development of catalysts for the conversion of cheaper lignocellulosic biomass is required.^731,975^

2,5-Dimethylfuran (DMFu) can be produced from carbohydrates and can undergo Diels–Alder coupling with ethylene, followed by dehydration, to give *p*-xylene (Figure 80).^976^ A dealuminated commercial H-Beta zeolite catalyst gave 99% conversion and a *p*-xylene yield of 97%, attributed to a balanced Brønsted/Lewis acid ratio of 2.3.^977−980^ However, the microporous nature of the zeolite catalysts lead to pore blockage, coke formation and consequential catalyst deactivation.^981^ Doping with γ-Al_2_O_3_ maintained a 97% *p*-xylene yield and decreased the coke deposit on the H-Beta catalyst from 12 to 7 wt %, but additional research into catalyst deactivation is important.^982^ HMF can be converted into DMFu *via* hydrodeoxygenation and then converted to *p*-xylene *via* the aforementioned route.^983,984^ A Pd-Au/ZrO_2_ catalyst was reported to convert HMF to *p*-xylene *via* these steps, with a yield of 89% and significantly higher reusability than the aforementioned zeolite catalyst (Table 18, entry 33).^790^ In 2013, Virent patented a heterogenous catalysis process for the derivation of ≥C8 hydrocarbons, including *p*-xylene, from biomass-derived sources.^985^ Commercialized as BioFormPX, their bio-based *p*-xylene has been converted to PET *via* traditional methods and used in PET applications.^986,987^

**Figure 81:**
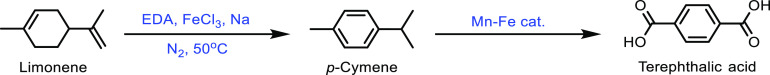
Conversion of bio-based limonene to terephthalic acid (TPA) *viap*-cymene.

Non-*p*-xylene routes to TPA are mostly from lignin or lignin-derived compounds. Lignin benefits from high aromatic content and extremely high renewable abundance, and many useful phenolics have been successfully obtained from lignin, including vanillin, vanillic acid, syringaldehyde and syringic acid.^988−990^ While initial work focused on lignin oxidation treatment, recent advancements in catalytic transformation reveal that reductive lignin depolymerization gives aromatic monomers with much higher yields.^988,991−993^ Corn-stover derived lignin oil was converted to phenolics *via* this route, and then transformed into TPA in 3 steps (Table 18, entry 34).^791^ Demethoxylation with MoO_*x*_/AC catalyst and subsequent carbonylation with a homogeneous Pd-based catalyst converted the phenolics into 4-alkylbenzoic acids, which were then oxidized to TPA with a [Co(OAc)_2_ + Mn(OAc)_2_ + KBr] catalyst. While the TPA yield from was only 15.5 wt %, this is promising work for the valorization of cheap lignin. Glucose and glycerol were converted to isoprene and acrylic acid, respectively, and underwent cycloaddition catalyzed by TiCl_4_ to give 4-methlycyclohex-3-enecarboxylic acid with a 90% yield (Table 18, entry 35).^792^ This was then converted to TPA *via* vapor-phase aromatization and oxidation. Treatment of limonene, readily derived from citrus peel, with FeCl_3_, ethylenediamine and sodium at 50 °C gave a 99% yield of *p*-cymene, which was then oxidized with a MnO_*x*_-FeO_*x*_ catalyst to give TPA with a yield of 51% (Figure 81, Table 18, entry 36).^793^

Current bio-based routes to TPA lack economic viability due to low yields, complicated procedures, feedstocks with limited supply and harsh reactions conditions.^731^ Significant research into additional feedstocks and process integration to reduce purification steps are required before significant amounts of TPA from bio-sources will be produced.

2,5-Furandicarboxylic acid (FDCA), one of the top value-added chemicals derived from biomass, is produced on a 25 MT annum^–1^ scale.^731,994^ FDCA is anticipated to find applications in polyesters such as polyethylene 2,5-furanoate (PEF) as a substitute for the petrochemically derived TPA in polyethylene terephthalate (PET).^995^ Consequently, FDCA synthesis from a range of feedstocks has been widely studied, with HMF emerging as currently the most suitable. Although reviewed in detail elsewhere,^731^ a consolidated summary of the optimal results within each catalyst class from each feedstock is provided herein. Commercially, FDCA and subsequent polyesters of FDCA are produced by Avantium with their YXY catalytic Technology, predominantly from fructose.^996^

**Figure 82:**
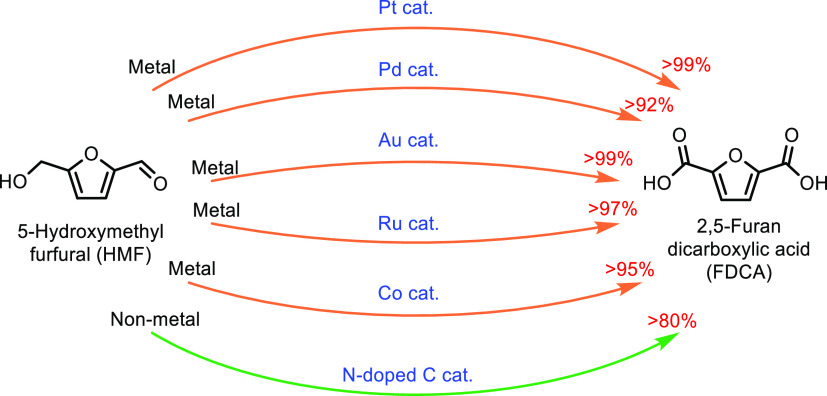
Most effective metal and non-metal catalyst types for the conversion of HMF to FDCA.

The majority of work in this area concerns the conversion of HMF to FDCA or its methyl diester, i.e., 2,5-furandimethyl carboxylate (FDMC), and several other feedstocks are converted *via* HMF.^809,997,998^ A range of noble-metal based Pt, Pd, Au and Ru catalytic systems have been investigated, alongside several non-noble-metal based catalytic systems, with the catalytic performance significantly affected by the nature of their supports (Figure 82, Table 18, entries 37–51).^731^ A Pt/N-doped-C catalyst without additive and a Pt_5_Bi_1_/C catalyst with Na_2_CO_3_ additive produced FDCA from HMF with yields of 99+%.^795,999^ Pd systems suffer lower selectivity than Pt, with FDCA yields of 92% using Pd/HT without additive and 93% using Fe_2_O_3_@HAP-Pd(0) with K_2_CO_3_.^731,796,797^ Au catalytic systems are highly active for HMF oxidation in basic medium with high, with yields of >99% obtained using both Au/HY zeolite and Au/Ce_0.9_Bi_0.1_O_2_ catalysts with NaOH as additive.^798,799^ A Pd-Au catalyst had a synergistic effect on the conversion, with 100 FDCA% selectivity and yield of FDCA.^800^ A Au/CeO_2_ catalyst achieved a 99% yield of FDMC, the methyl diester of FDCA, due to the enhanced solubility of the methyl ester.^801^ Ru offers an economical advantage over the other noble-metal systems, and affords moderate FDCA production both with and without base.^802,998,1000^ A simple Ru/C catalyst with a Mg(OH)_2_ additive and a Ru/MnCo_2_O_4_ catalyst without additives gave FDCA yields of 97 and 99%, respectively.^802,803^ With Ru-based systems, an increase in productivity is observed with microwave heating.^1001^ Several mixed metal oxides of non-noble metals including Cu, Ce, Mn and Co have also been proven effective at FDCA conversion from HMF, achieving yields 99+%.^804−806^ A carbon nanotube-supported Co catalyst (Co-CN) used for FDMC production from a concentrated HMF solution (24%) gave a 95% yield (only 89% was isolated) and avoided the polymerization of HMF. Interestingly, a non-metal N-doped nanoporous-C catalyst with K_2_CO_3_ as an additive has also been used for HMF oxidation, with FDCA yields of 80%, albeit it with a low productivity.^808^ Low productivity may be less of an issue for non-metal catalysts as lower prices enable higher catalytic amounts to remain cost-effective, especially if the non-metal catalysts can be produced sustainably.

**Figure 83:**
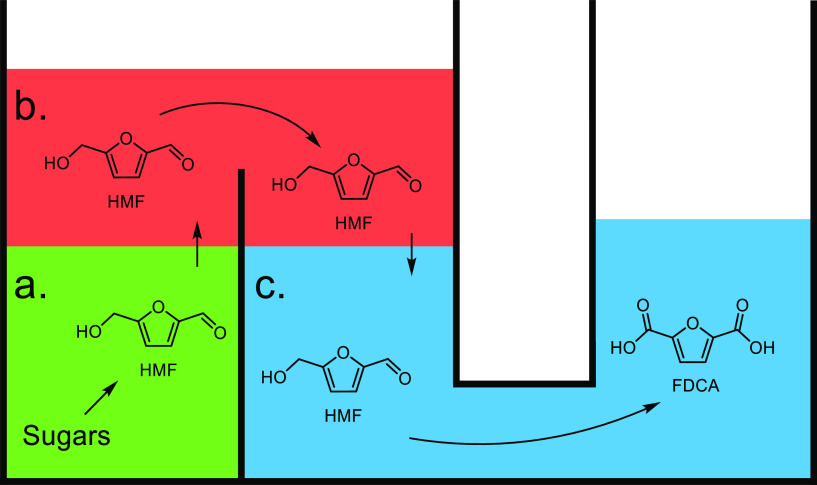
Conversion of glucose and fructose to FDCA *via* HMF in a triphasic system. (a) Phase 1: Tetraethylammonium bromide (TEAB), Amberlyst-15/CrCl_3_, H_2_O. (b) Phase 2: Methyl isobutyl ketone (MIBK). (c) Phase 3: Au_8_Pd_2_/HT, Na_2_CO_3_, H_2_O.

Glucose and fructose have also been investigated as feedstocks for FDCA production (Table 19, entries 52–56).^809−812,997,998,1002,1003^ Some studies simply convert the sugars to HMF, separate the HMF, and then convert it to FDCA, one-pot routes without the need to separate intermediates are advantageous with a view to industrial upscaling. A Pd/CC catalyst with acidic-SO_3_H sites was used to first dehydrate fructose to HMF under N_2_, and then to convert HMF to FDCA with K_2_CO_3_ under oxygen, giving an overall yield of 64%.^809^ Similar yields of 65% were reported both using Amberlyst-15 and Ru/C catalysts for the first and second steps, respectively, and using FDCA and Pt/C for the first and second steps, respectively.^810,811^ Notably, the latter study utilizes the FDCA product as the acid catalyst. The highest reported yield of FDCA from fructose was performed using Amberlyst-15 and AuPd/HT in the first and third phase of a triphasic system for the dehydration and oxidation steps, respectively, yielding 78% FDCA (Figure 83).^812^ Additionally, the inclusion of CrCl_3_ in the first phase enabled glucose to be used directly by first undergoing isomerization to fructose, achieving a 50% yield from glucose.

#### 5.1.2 Diols

**Figure 84:**
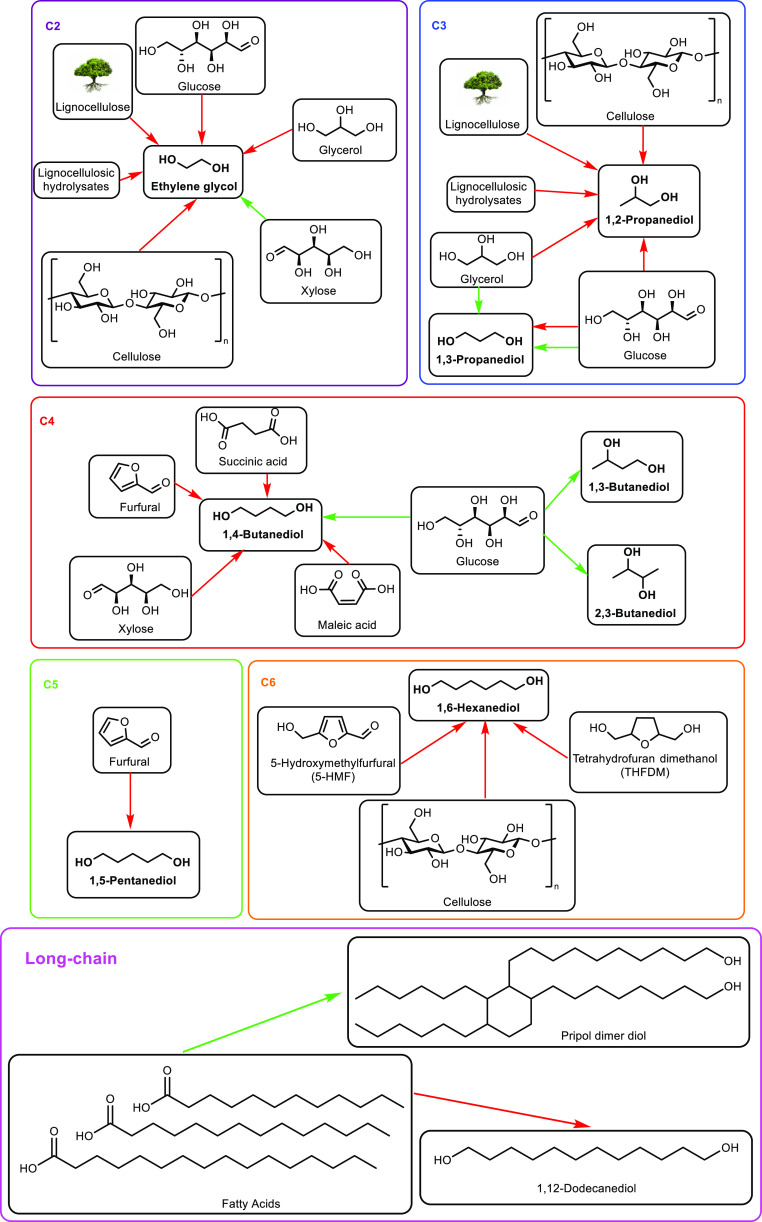
Summary of the chemical (red arrows) and biotechnological routes (green arrows) to short chain aliphatic organic diols from biomass sources.

Diols are crucially important difunctional compounds for polymer synthesis due to their use in polyureas, polycarbonates and, in particular, polyesters. The wide availability, ease of synthesis and relative stability of hydroxy-functionalized chemicals have enabled the preparation of a plethora of diol compounds, although for the scope of this review only aliphatic diols are reviewed in detail (Figure 84). Beyond 4-carbon diols, only the production of α,ω-diol isomers are reviewed in the aliphatic part of the report, as the number of structural isomers grows exponentially with the number of carbons.

##### 5.1.2.1 C2 Diol

Ethylene glycol (EG, 1,2-ethanediol), largely used for the production of anti-freeze liquids and polyethylene terephthalate (PET), is predominantly made from petrochemical ethylene.^1004,1005^ Ethylene is oxidized to ethylene oxide with a Ag catalyst, and subsequently hydrated to EG in the presence of a catalyst, such as a solid acid.^1006,1007^ In recent years, there has been significant progress on utilizing renewable feedstocks for ethylene glycol synthesis, both *via* chemical transformations and *via* biotechnological routes.

**Figure 85:**
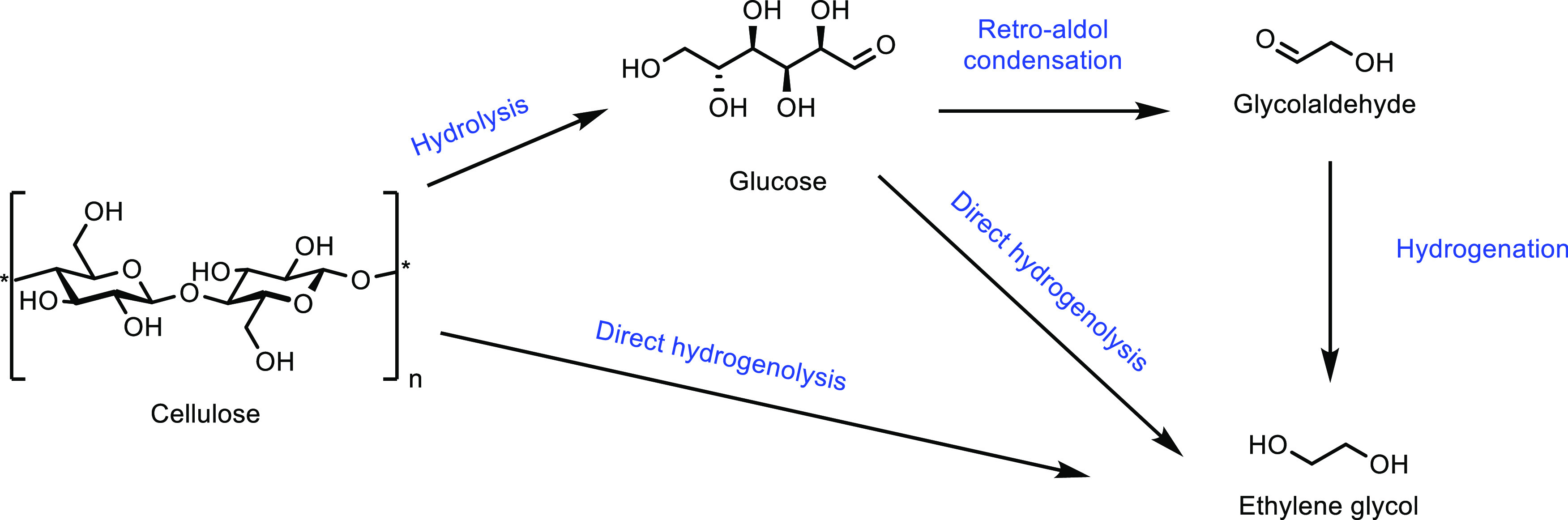
Conversion of cellulose and glucose to ethylene glycol (EG). Cellulose can be converted in a three-step procedure or *via* direct hydrogenolysis. Glucose is an intermediate of the direct hydrogenolysis pathway and can be used directly as a feedstock for direct hydrogenolysis to EG.

The dominant route to biomass-derived EG involves the drop-in of biomass-derived ethylene to the conventional route.^1008,1009^ Biomass-derived ethylene is predominantly produced *via* the fermentation of sugar to ethanol and the subsequent dehydration to ethylene (see section 2.1). A direct route *via* the hydrogenolysis of sugars suffers from poor EG selectivity, coyielding propylene glycol (PG) and glycerol as other valuable side products (Figure 85).^1010,1011^ The EG selectivity from sugar has been improved to 76% with a tungsten based catalyst.^1012,1013^ Cellulose can also be converted to EG *via* hydrolysis to glucose, retro-aldol condensation to glycolaldehyde, and finally hydrogenation to EG (Figure 85).^1014−1017^ An acidic aqueous solution at ∼250 °C and under high pressure, is used for the hydrolysis, with the 2^nd^ and 3^rd^ step using a tungsten-based and Raney-Ni catalyst, respectively. EG yields above 75% are reported for the hydrogenolysis of pure cellulose (Table 20, entries 1–3), while yields from pre-treated lignocellulosic biomass under similar conditions have 64% (Table 20, entries 4 and 5).^1018,1019^ Cellulose directly from pre-treated lignocellulosic biomass with 39% lignin content gave an EG yield of 35%, with the lignin found to not significantly retard the reaction, as was expected elsewhere (Table 20, entry 6).^1020−1022^

Direct cellulose conversion brings with it difficulties in handling solid cellulose and the need for catalysts capable of performing multiple reactions.^1011^ As such, the direct conversion of sugars to EG has been investigated under similar conditions, benefiting from higher feasible feedstock concentrations (up to 35 wt %) and enhanced catalyst lifetime in the absence of typical lignocellulosic impurities (Table 20, entries 7–9).^1023−1026^ However, EG yields from sugar hydrogenolysis exceeding 60% have yet to be reported, while reported yields from cellulose are higher.^1027^ The counterintuitive higher yield obtained from cellulose was attributed to the slow cellulose hydrolysis reaction preventing a build-up of sugars prior to the slow retro-aldol reaction, which otherwise would result in the production of sorbitol and other side-products. Ooms *et al*. achieved an EG productivity of 300g L^–1^ h^–1^ with a yield of only 36 wt %, suggesting that the ease of operation somehow compensate for the lower yields. While less studied, biomass hydrolysates are a more pragmatic feedstock than glucose, and corn stover hydrolysates were converted to EG with a cheap Raney-Ni/NaOH catalyst system with a mass yield of 0.19 g g^–1^ (Table 20, entry 10). Compared to the best-reported glucose conversion to EG (mass yield of 0.62 g g^–1^) using a Ni/W catalyst, this reasonably high 0.19 g g^–1^ yield is promising.^1024,1025^

EG is also produced as a side product during glycerol hydrogenolysis toward 1,2-propanediol, with increased reaction temperatures to 300 °C enabling EG yields up to 37 mol %, albeit with increased side product formation (Table 20, entry 11).^1028,1029^ However, the separation of EG is difficult and most studies optimize the process to reduce EG production, so glycerol hydrogenolysis is unlikely to develop as a route to sustainable EG.^1030,1031^

In 2019, Avantium completed the construction of a demonstration plant for the one-step conversion of glucose to EG using a Ru-H_2_WO_4_ catalyst system with a promotor metal.^1032,1033^ Interestingly, 1,3-butanediol is also produced as a side-product, which could be separated *via* azeotropic distillation. In 2021, a collaboration between Braskem and Haldor Topsoe also resulted in the completion of a demonstration plant for EG production from glucose using patented MOSAIK technology (Table 20, entry 12).^1034−1036^ The three-step process begins with the pyrolysis of a sugar solution to predominantly glycolaldehyde at >500 °C, and then hydrogenating those compounds with Cu/C at 230 °C followed by Ru/C at 80 °C, in the gas and liquid phase, respectively.^1035,1037^ The inventors report a combined yield of the hydrogenation steps of >98%, and expect a commercial plant production by 2023.^1028,1036^ The amount of current investments in producing EG from sugars suggests that this route has been deemed economically viable, but the field of EG production would benefit from continued research into improved catalysts, particularly for more realistic feedstocks such as lignocellulosic hydrolysates.

There are no natural biological pathways to synthesize EG directly from glucose, although engineering microbes enables some biotechnological routes to EG (bio-EG) from xylose.^1038^ Metabolic simulations supported the engineering of an engineered *E. coli* strain that achieved a titer of 108.2 g L^–1^ EG at a yield of 0.36 g g^–1^ xylose, the highest titer thus far reported from xylose (Table 19, entry 6).^852^ Two additional pathways have been proposed, both of which pass through glycolaldehyde and dihydroxyacetone phosphate, with the second pathway also including an initial epimerization of d-xylulose to d-ribulose.^853,1039^ Optimization of the two routes yielded titers of 20 and 40 g L^–1^ of EG, respectively, with yields of 0.38 and 0.35 g g^–1^ xylose, respectively (Table 19, entry 7). The carbon utilization of these pathways is limited to 40% as the 5-carbon pentose is converted into a 2-carbon glycolaldehyde and a 3-carbon intermediate, with only the former converted into EG.^1038^ The combination of these pathways with a serine synthesis pathway allows utilization of the 3-carbon intermediates to produce additional glycolaldehyde, doubling the maximum total conversion of pentose carbon to 80%, but this requires significant further work.^1040−1042^ A recent study produced bio-EG from glycerol *via* an enzymatic cascade through glycolaldehyde, and yielded 0.52 g EG/g glycerol, the highest reported yield from any route to date.^1043^ The current price of EG is low due to established large scale industrial production, and titers and yields of EG should exceed 100 g L^–1^ and 0.5 g g^–1^ feedstock before presenting bio-EG as a competitive alternative.

##### 5.1.2.2 C3 Diols

The two propanediol isomers, 1,2-propanediol (1,2-PDO; propylene glycol) and 1,3-propanediol (1,3-PDO; trimethylene glycol) are industrially important chemicals commonly used in polymer synthesis to make unsaturated polyester resins and polytrimethylene terephthalate, respectively. Many studies have investigated to both 1,2-PDO and 1,3-PDO from biomass feedstocks such as glycerol, glucose and cellulose, both *via* chemical and biotechnological routes.

**Figure 86:**
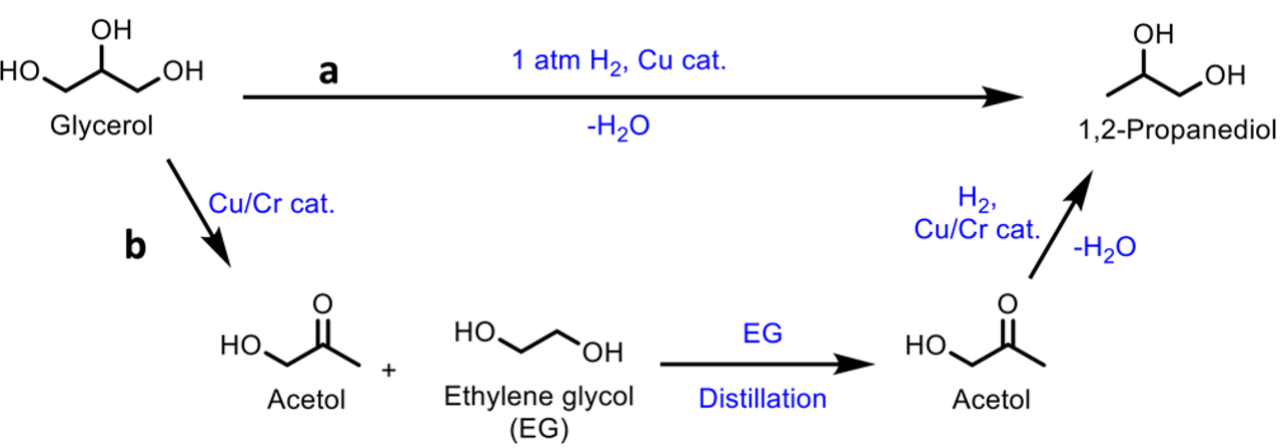
Hydrogenolysis of glycerol to 1,2-propanediol *via* (a) a single-step approach, or (b) a two-step approach utilizing the difference in boiling point to remove the EG by-product before conversion of acetol to 1,2-PDO.

1,2-PDO is readily produced via the selective catalytic hydrogenolysis of glycerol, a route that is well established as it can be integrated with waste glycerol produced during the transesterification of fatty acids for biodiesel production (Figure 86).^1058^ Glycerol hydrogenolysis produces a range of C2 and C3 alcohols and diols, including EG, and 1,3-PDO, but has the highest selectivity for 1,2-PDO. Hence, glycerol hydrogenolysis is typically studied to maximize 1,2-PDO selectivity and yield. A 1,2-PDO yield of 85% was obtained from glycerol *via* hydrogenolysis at 230 °C with a Ru/Cu-modified bentonite catalyst (Table 20, entry 13).^1029^ The Archer Daniels Midland Company (ADM) and Oleon/BASF have both commercialized 1,2-PDO production from bio-diesel-derived glycerol, with annual production capacities of 100 and 20 kt 1,2-PDO, respectively.^1059,1060^ ADM reported a 61% reduction in greenhouse gas emissions, compared to the petrochemical route.^1059^ Glycerol hydrogenolysis typically occurs either in a single, high temperature step that encourages glycerol conversion at the cost of reduced 1,2-PDO selectivity, or *via* a patented two-step approach (Figure 86a,b, respectively).^1061,1062^ In the latter route, glycerol is converted to acetol at high temperature and reduced pressure and then hydrogenated at a lower temperature and higher hydrogen pressure.^1062^ The EG side product is removed before the hydrogenation step due to the separation of EG from acetol being significantly easier than from 1,2-PDO, and the hydrogenation step requires H_2_/glycerol ratios lower than 15, hence reducing production costs.^1062^ A fixed-bed reactor with a 130–200 °C temperature gradient and Cu catalysts achieved a similar effect, albeit without the intermediate removal of EG, and obtained a 1,2-PDO yield of 0.79 g g^–1^.^1061^ However, the scalability of this system is questionable due to both the high H_2_/glycerol ratio used (i.e., 140:1) and the exothermicity of the overall reaction, so the two-step process currently appears to be the most pragmatic route.

Since H_2_ production often relies on either petrochemical sources or energy-intensive processes, some studies have investigated glycerol hydrogenolysis without external H_2_, but estimated 1,2-PDO costs were 2-3 times higher and the estimated environmental impact was even greater than the petrochemical route.^1011,1058,1063^ This area has benefitted from significant research in recent years, but further study to achieve lower H_2_ requirements, catalysts for utilizing raw glycerol and improved separation procedures are all required for increased commerciality.

1,2-PDO can also be produced from cellulose and glucose feedstocks, as long as an isomerization of glucose to fructose takes place prior to hydrogenolysis, typically with a basic catalyst.^1011^ Similar yields and productivities are reported using various different catalytic systems, including Cu-WO_*x*_/Al_2_O_3_, Pd-WO_*x*_/Al_2_O_3_ and CuCr in the presence of Ca(OH)_2_.^1047,1064,1065^ Xiao *et al*. reported the highest 1,2-PDO yield from both glucose and cellulose by hydrogenolysis (53 and 42%, respectively) *via* first converting the feedstock to sorbitol or mannitol at 140 °C and then converting to glycols under pressurized H_2_ and in the presence of Ca(OH)_2_ and CuCr (Table 20, entries 14 and 15).^1047,1048^ Directly using lignocellulosic sources presents a more realistic use-case, with a 1,2-PDO yield of 39% achieved with a Ni-W_2_C/AC catalyst from a Jerusalem artichoke feedstock, comparable to the results with purified cellulose (Table 20, entry 16).^1049^ Similarly, hydrogenolysis of lignocellulosic hydrolysates with Raney-Ni and NaOH produces 1,2-PDO as the major product with a yield of 41% (Table 20, entry 17).^1025^

Besides catalytic chemical routes, several microbes naturally produce 1,2-PDO (bio-1,2-PDO) from l-rhamnose or l-fucose, although these carbon sources are considered too expensive for commercial viability.^1066^ Alternatively, dihydroxyacetone phosphate can be converted to bio-1,2-PDO *via* methylglyoxal, and can be produced from a range of sustainable feedstocks including sugars, glycerol or CO_2_.^1067−1069^ The reductant coenzyme-1 (NADH) is consumed to produce 1,2-PDO from glucose or glycerol, so co-production of 1,2-PDO with NADH-generating acetate production was engineered and optimized to give a titer of 0.59 g L^–1^ and a 1,2-PDO yield of 0.48 g per gram glucose.^1067,1070,1071^ To reduce the effect of toxic intermediates, zinc finger enzyme scaffolds and microcompartments have been investigated, which increased 1,2-PDO yields by 350% and 250%, respectively, relative to the free-enzyme strain.^1072,1073^ CO_2_ has been also been converted into 1,2-PDO in *S. elongatus*, although titers are very low (i.e., <0.2 g L^–1^). Interestingly, engineered *E. coli* can produce enantiomerically pure (>98% ee) *R-* and *S-*1,2-PDO from d- and l-lactate, respectively, *via* a non-methylglyoxal-dependent pathway, with titers of 1.5–1.7 g L^–1^.^1074^ The titers and yields of bio-1,2-PDO are much lower than those of biotechnologically produced 1,3-PDO, while commercial 1,2-PDO is cheaper than 1,3-PDO. Significant further research is required for feasible commercialization of bio-1,2-PDO, but enantiomerically pure 1,2-PDO could provide interesting polymer possibilities, as tacticity influences physical polymer properties.

**Figure 87:**
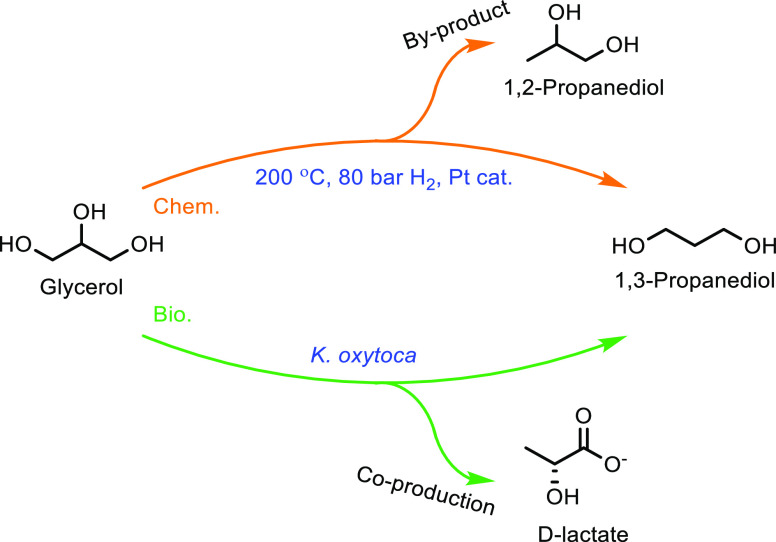
Chemical and biotechnological routes to 1,3-propanediol from glycerol. The chemical hydrogenolysis requires elevated temperatures and pressures to reduce the amount of the 1,2-Propanediol by-product product. The biotechnological route performs best with the coproduction of d-lactate, due to the balancing of oxidative and reductive pathways.

Research into bio-based 1,3-PDO production focuses on the conversion of glycerol (Figure 87). While catalytic studies focus on utilizing more concentrated, purer glycerol feedstocks to prevent catalyst deactivation, fermentative conversion has been more successful with raw glycerol streams due to high enzyme selectivity.^1075,1076^ The hydrogenolysis of glycerol to produce 1,3-PDO usually proceeds at 140–200 °C, with 40–80 bar H_2_, over a Pt catalyst, with 1,3-PDO yields in the range of 0.2–0.4 g g^–1^ and selectivity of 30–71%.^1011^ Most catalysts for glycerol hydrogenolysis produce 1,2-PDO with much higher selectivity than 1,3-PDO, unless the temperature, glycerol concentration, or H_2_ pressure are increased.^1011,1051,1077,1078^ The highest yield obtained in batch operation, 66% and 0.55 g g^–1^, utilized a dilute glycerol solution and a Pt/WO_*x*_/AlOOH catalyst, but required 50 bar H_2_ and energy intensive separation and/or purification steps (Table 20, entry 18).^1050^ Contrastingly, continuous operation at atmospheric pressure over a zeolite Pt-Cu catalyst has achieved yields of 53% and 0.45 g g^–1^ (Table 20, entry 19).^1051^ Despite the slightly lower yields, the milder conditions, more concentrated feed and continuous nature of the process presents the best current option for commercial 1,3-PDO production *via* catalytic glycerol hydrogenation. Although yields remain lower than for 1,2-PDO, the higher selling price of 1,3-PDO could result in commercial viability.^1011,1079^

Bio-1,3-PDO is naturally produced from glycerol by several microbes, *via* dehydration to 3-hydroxypropionaldehyde (3-HPA) and then reduction to 1,3-PDO.^1080−1083^ The reductant, NADH, is typically regenerated *via* the glycerol oxidation pathway, producing several natural by-products.^1084^ Most previous strategies sought to optimize 1,3-PDO synthesis from glycerol by blocking one or some of the by-product synthesis pathways, but this proved ineffective at increasing yields as one oxidative pathway is required to enable NADH regeneration.^853,1085−1089^ Instead, the coproduction of 1,3-PDO and another valuable NADH-regenerating chemical has been conducted and an engineered *Klebsiella oxytoca* has produced 76 g L^–1^ 1,3-PDO and 112 g L^–1^d-lactate at a 95% conversion yield from glycerol (Table 19, entry 8).^854,1090,1091^ From glycerol and glucose as cosubstrates, parallel pathways in an engineered *C. glutamicum* generated 1,3-PDO and glutamate, respectively, with NADH consumed in the former pathway and regenerated in the latter.^1091^ The 1,3-PDO yield was extremely high (∼1.0 mol/mol glycerol) and the glutamate yield was higher than in the industrial glutamate-producing strain. d-Lactate and glutamate can be easily separated from 1,3-PDO, evidence that coproduction processes are promising for the future of efficient, cost-effective biorefineries.^1038^ Further, an engineered *K. pneumoniae* produced 1,3-PDO and 3-HP at a yield of 82%, with an additional supply of B_12_.^1090^

While there are no natural pathways directly to 1,3-PDO from sugars, engineered microbes can combine the glucose-to-glycerol and glycerol-to-1,3-PDO pathways to produce 1,3-PDO from glucose.^1038^ Using engineered *E. coli*., DuPont have achieved a titer of 135 g L^–1^ of 1,3-PDO from glucose, although the pathway requires additional B_12_ (Table 19, entry 9).^855,1092^ A proposed non-natural route to circumvent the B_12_ requirement utilizes homoserine as a precursor and can use simple media.^1093^ Since serine synthesis pathways are established, the two routes could be effectively integrated to give up to 1.5 mol 1,3-PDO per mole glucose without the need for B_12_ supplementation.^1094−1096^ While Bio-1,3-PDO has been commercialized, wide adoption is limited by the relatively high price, partly due to the low price of crude glycerol.^1038^ Additionally, implementation of B_12_-avoiding pathways can significantly reduce the production cost of Bio-1,3-PDO.

##### 5.1.2.3 C4 Diols

**Figure 88:**
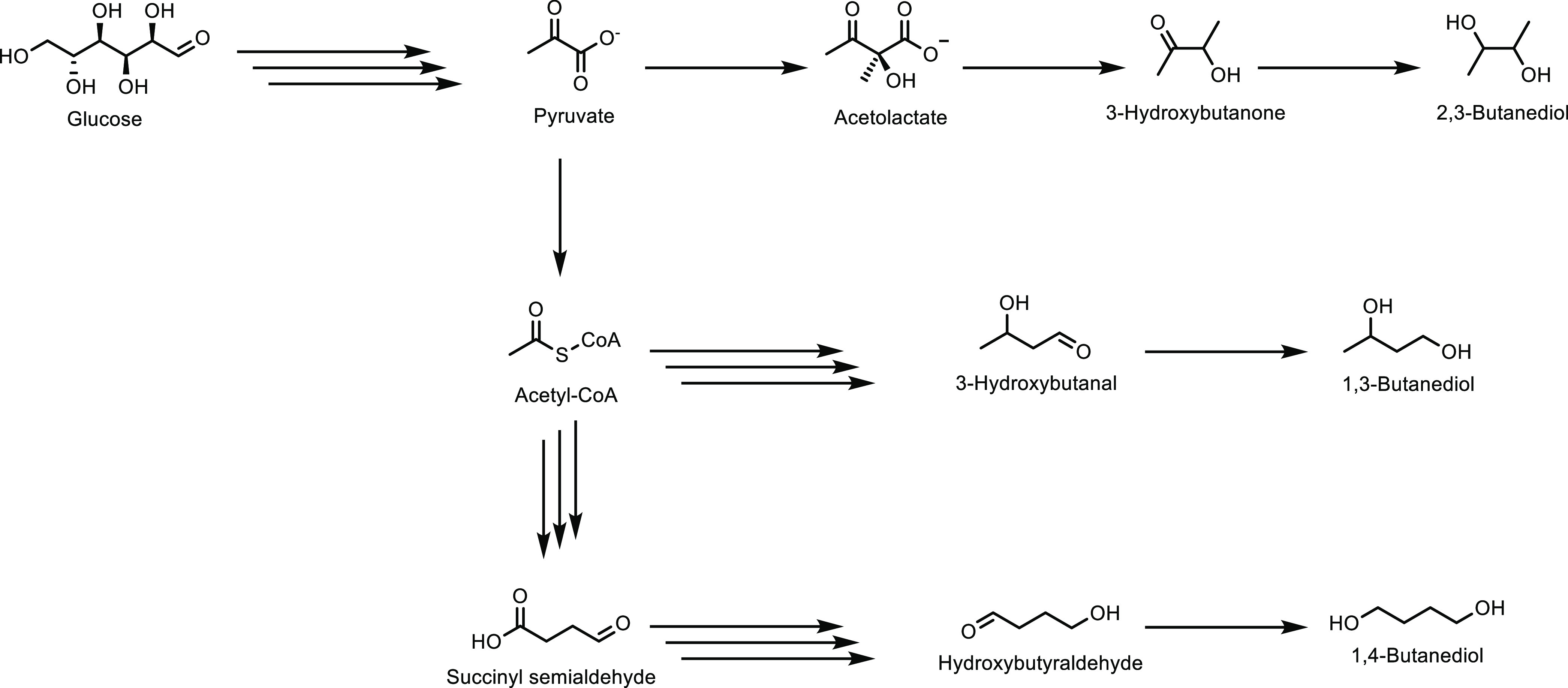
Synthesis pathways of butanediols starting from glucose.

There are four butanediol isomers, 1,2-BDO, 1,3-BDO, 2,3-BDO and 1,4-BDO, which all show industrial applications. 1,4-BDO is most important as a monomer and solvent, and an annual production of 2 million ton.^1097^ 2,3-BDO is considered as a potential platform chemical, 1,3-BDO is a widely used solvent and 1,2-BDO finds use in polyester resins.^1098−1101^ The majority of work on the production of butanediols study biotechnological routes. Bio-1,4-BDO production has been reviewed in depth elsewhere.^597,1038^ 1,4-BDO is predominantly produced *via* hydrogenation of butynediol, acetoxylation of butadiene and hydrogenation of maleic anhydride, with the last route attracting interest due to its operational safety advantages and possible integration with sustainable maleic anhydride production (Figure 88).^1102^ The hydrogenation of maleic anhydride or maleic anhydride-derived dialkyl esters *via* a γ-butyrolactone (GBL) intermediate has been conducted with noble-metal and Cu-based catalysts, with 1,4-BDO selectivity above 66% (Table 20, entry 20).^614,1052,1103^ 1,4-BDO has also been obtained from several renewable platform molecules, including succinic acid (SA) and furfural, although the 2 Mt annum^–1^ scale of 1,4-BDO production far exceeds that of both SA and furfural, at 60 and 604 kt annum^–1^, respectively (Table 20, entries 21 and 22).^580,583,1054,1104−1109^ If produced sustainably on a larger scale, these feedstocks could provide pragmatic routes to 1,4-PDO. A range of noble metal catalysts for the direct hydrogenation of SA have been investigated, with a Re/Pd catalyst giving the highest 1,4-BDO yield of 66%.^580,583,1053,1106,1108,1109^ The oxidation of furfural yields SA, maleic anhydride, and 2(5*H*)-furanone, which can all be converted to 1,4-BDO *via* GBL and a two-step oxidation of furfural over a Pt/TiO_2_-ZrO_2_ catalyst with 85% 1,4-BDO yield.^818,1054,1107,1110,1111^

A highly engineered *E. coli* strain produces a 1,4-BDO titer and yield of over 120 g L^–1^ and 80% from glucose (Table 19, entry 10).^597^ An alternative pathway from xylose yielded 12 g L^–1^ with glucose as a cosubstrate (Table 19, entry 11).^856^ While the yields from xylose are much lower than from glucose, using non-food sugars can help to avoid food supply conflicts, and similar pathways can be engineered from other non-food sugars.^856^ In 2020, a biorefinery modelled by De Bari *et al*. estimated the conversion of 60 kt annum^–1^ residual lignocellulosic biomass to 8 kt annum^–1^ of 1,4-BDO, with the energy generated from secondary streams capable of sustaining 78% of the total electricity demand.^1112^ 2,3-BDO is produced naturally by various microorganisms and exists as a mixture of three stereoisomers.^1113−1117^ No non-natural pathways have been proposed, but wild-type microorganisms such as *K. pneumoniae* can produce titers of 150 g L^–1^ 2,3-BDO from glucose, and strain engineering has increased the molar yield to 96% (Table 19, entry 12).^857,1038,1070,1118^ Enantiopure stereoisomers of 2,3-BDO have also been investigated, with *S. cerevisiae* yielding titers of over 100 g L^–1^ (2*R*,3*R*)-BDO from a mixture of glucose and galactose, two major components of red algae.^1119^ Pilot scale production of 2,3-BDO and coproduction of 2,3-BDO with 1,3-PDO have been demonstrated.^1120^ The remaining BDOs are not produced naturally, but pathways to 1,4-BDO and 1,3-BDO have been proposed in recent years and the biotechnological production of 1,4-BDO has been commercialized by Genomatica.^916,1097,1101,1121^ A 4-step pathway from glucose to 1,3-BDO in engineered *E. coli* and gave a titer and yield of 15.7 g L^–1^*R*-1,3-BDO (98% ee) and 37%.^1101,1122^ Optimizations including the conversion of a by-product into desired 1,3-BDO, increased the molar yield to 57% (Table 19, entry 13).^858^ To date, no metabolic pathways to synthesize 1,2-butanediol have been reported.

##### 5.1.2.4 C5 Diol

**Figure 89:**
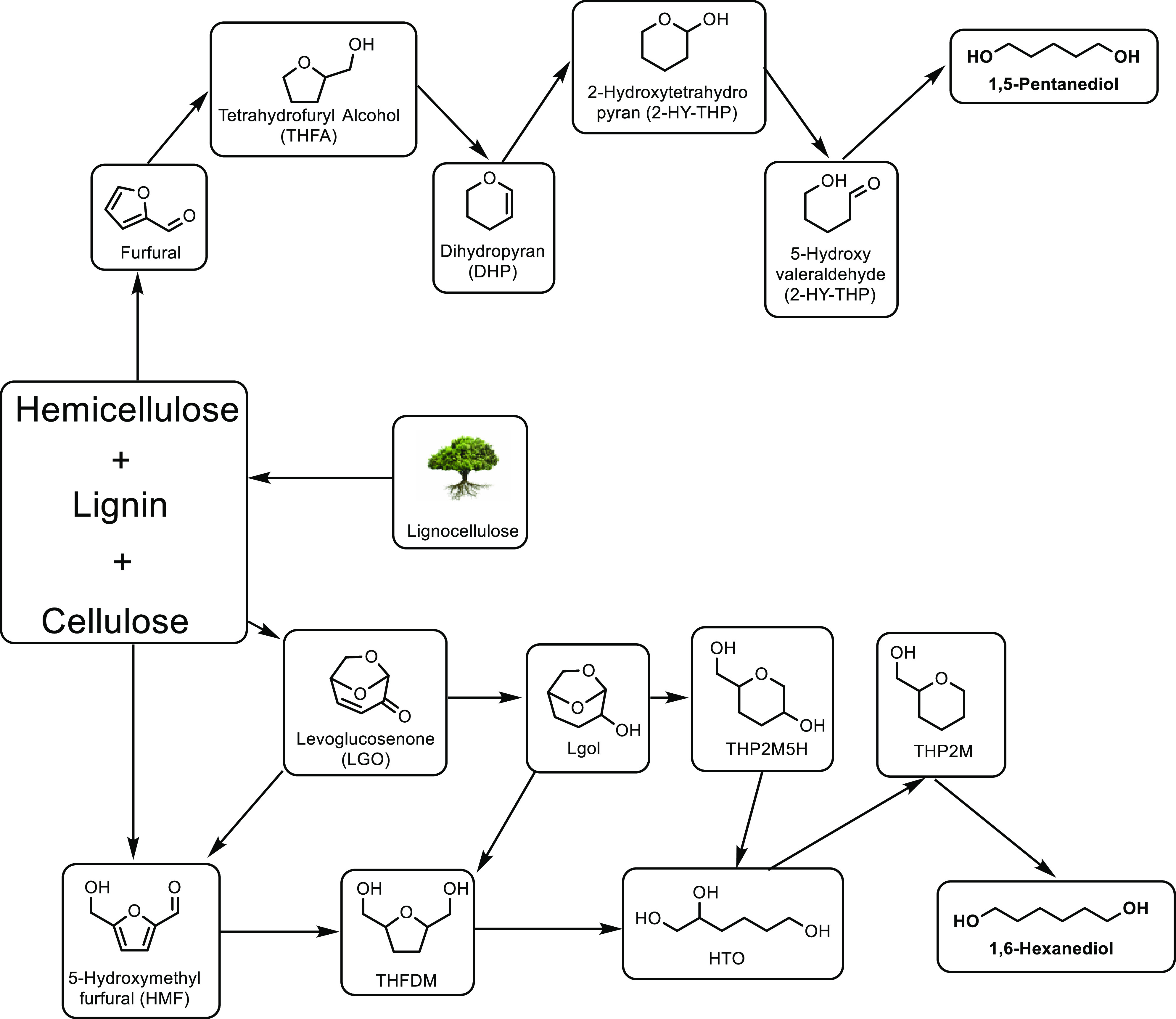
Simplified reaction scheme for the combined production of 1,5-pentanediol (1,5-PeDO) and 1,6-hexanediol (1,6-HDO) from lignocellulose, as present by He *et al*.

1,5-Pentanediol (1,5-PeDO) is produced on a 3 kt annum^–1^ scale, with a consequentially high $6000 ton^–1^ market price, three times higher than that of the shorter alkyl spaced 1,4-BDO, due to limited accessibility of C5 petrochemical feedstocks.^1123^ 1,5-PeDO is currently produced through the hydrogenation of glutaric acid, which is produced from acetylene and formaldehyde *via* the 1,4-butynediol intermediate.^1124,1125^ 1,4-butynediol undergoes hydrogenation to 1,4-butanediol, dehydration to GBL, ring opening with potassium cyanide, and finally hydrolysis to glutaric acid. This route suffers from multiple steps, toxic chemicals such as potassium cyanide, and relies on petrochemical feedstocks. Research into 1,5-PeDO production from biomass sources has focused on the selective hydrogenolysis of tetrahydrofurfuryl alcohol, which is produced *via* Ni- or Cu-catalyzed hydrogenation of the platform chemical furfural.^1126−1128^ The hydrogenolysis is conducted over bimetallic noble metal catalysts, but the catalysts are expensive and have low activities.^1055^ He *et al*. demonstrated a multi-step process to produce PeDO from furfural with a sequential dehydration-hydration-hydrogenation route *via* tetrahydrofurfuryl alcohol, dihydropyran (DHP), and 2-hydroxytetrahydropan (2-HY-THP) (Figure 89, Table 20, entry 23).^1055^ The overall process has a 1,5-PeDO yield of 80% from furfural, and a modelled pathway from white-birch lignocellulose estimated a 1,5-PeDO selling price of ∼$4000 ton^–1^, a reduction of ∼33% from current 1,5-PeDO prices.^1055^

##### 5.1.2.5 C6 Diol

1,6-Hexanediol (1,6-HDO) is currently produced on a scale of 130 kt annum^–1^, with a market value of ∼$4400 t^–1^, *via* the catalytic reduction of adipic acid (AA) or its esters over Cu-, Co- or Mn-based catalysts, at ∼200 °C and 150–300 bar H_2_.^1055,1123^ While AA is currently produced from petrochemical (section 5.1.1.4), the scale of AA production (3 Mt annum^–1^) dwarfs that of 1,6-HDO, and any transition to sustainable AA production would enable direct substitution into 1,6-HDO production. Furthermore, many of the mentioned routes to bio-based AA had impurities rendering them unsuitable for nylon production, but not necessarily unsuitable for use in applications such as 1,6-HDO production.

A three-step process for 1,6-HDO production from biomass *via* hydrolysis/dehydration to HMF with dilute acid, hydrogenation to tetrahydrofuran dimethanol (THFDM) over Raney-Ni, and hydrogenation to 1,6-HDO with Cu_2_Cr_2_O_5_ was patented in 1983.^1129^ In the years since, several studies have reported conversions from HMF or one of the other intermediates.^1056,1130,1131^ A 1,6-HDO yield of 58% directly from HMF was achieved with expensive Pd/SiO_2_ and Ir-ReO_*x*_/SiO_2_ catalysts (Table 20, entry 24).^1056^ A high yield of 83% 1,6-HDO from levoglucosenone (LGO)-derived THFDM was reported in a DuPont patent using PtWO_*x*_/TiO_2_ at 160 °C and 5.5 MPa H_2_ (Table 20, entry 25).^1057^ He *et al*. reported a multi-step catalytic process for the production of 1,6-HDO from cellulose (Figure 70, Table 20, entry 26).^1055^ In the process, LGO and HMF were obtained from cellulose, converted to THFDM *via* hydrogenation and hydrogenolysis with Ni- and Pd-based catalysts, and converted to 1,6-HDO by hydrogenolysis of THFDM with a Pt-based catalyst.^1132,1133^ A combined LGO and HMF yield of 48% was achieved from cellulose with dilute sulfuric acid in a 99:1 THF/H_2_O mixture. LGO and HMF were converted to LGOL and THFDM, respectively, at 100 °C using Ni/SiO_2_ with near-quantitative yields, and then further converted at 150 °C with Pd/Si-Al, where LGOL underwent hydrogenolysis to THFDM with a selectivity of 63%. Overall, the selectivity of the LGO+HMF conversion to THFDM was 83%, but the total selectivity toward 1,6-HDO was estimated to be 94%, due to the conversion of an additional intermediate, THP2M5H, to 1,6-HDO *via* ring opening followed by the same steps as with THFDM. The THFDM, and THP2M5H, then underwent hydrogenolysis to HTO and conversion to 1,6-HDO in a batch reactor at 160 °C and 5.5 MPa H_2_, with a Pt-based catalyst and an overall 1,6-HDO yield of >70% from HMF. A modelled pathway from white-birch lignocellulose estimated a 1,6-HDO selling price of ∼$4000 ton^–1^, a similar price to 1,6-HDO derived from petrochemical sources.^1055^ Recently, Kim *et al*. presented the modelling of a similar multi-step process, with the final 1,6-HDO selling price estimated at $4700 ton^–1^ if the overall product yield can be increased from 23% to 40%, which is an estimate in quite good agreement with the work of He and co-workers.^1055,1134^

##### 5.1.2.6 Long-Chain Diols

The biotechnological production of C8–C16 α,ω-diols (α,ω-DOs) from renewable fatty acids has been conducted with modified *E. coli*, *via* ω-oxidation of fatty acids to ω-hydroxy fatty acids and their subsequent reduction.^1135^ The obtained α,ω-DOs yield peaked at 5 g L^–1^ at 96% conversion for the production of 1,12-dodecanediol, a 66-fold increase since biotechnological 1,12-dodecane was first reported in 2006.^1135,1136^ Croda produce a diacid from dimerizing unsaturated fatty acids (cf. section 5.1.1.5), which has been converted to diol and commercialized under the brand name Pripol (Figure 78b).^967^ As well as simple aliphatic diols, low-molecular weight hydroxyl-terminated polymers are often used as diols in step-growth polymerizations, including for polyurethane and polyurea formation but can also be employed for the synthesis of other polymer classes, e.g., polyethers, polyesters and polycarbonates. Although conventionally petrochemically sourced, oligomeric/polymeric diols have been produced from biomass sources including lignin, terpenoids, corn and soybean oil, as has been recently reviewed in detail by Gupta *et al*. and Sardon *et al*.^1137,1138^

##### 5.1.2.7 Aromatic Diols

Aromatic diols, typically referred to as bisphenols, are aromatic structures with 2 hydroxy groups attached to aromatic structures. Bisphenol A (BPA) is a diphenylmethane derivative with two *para* hydroxy groups, and has been widely used in polymer synthesis, particularly in polycarbonates. BPA is produced *via* the solid-acid catalyzed hydroxyalkylation of acetone with excess phenol.^1139^ As it is possible to produce both phenol and acetone from biomass, bio-based BPA can be produced from biomass *via* the current hydroxyalkylation route.^253,1140^ However, BPA and residual phenol are very toxic and several substitutes to BPA have been proposed and studies. Some alternative petrochemical bisphenols such as bisphenol F (BPF) and bisphenol S (BPS) have been produced to avoid the growing body of legislation limiting BPA use, but these exhibit similar toxicity issues and may face similar legislative constraints in the near future.^1141−1144^

**Figure 90:**
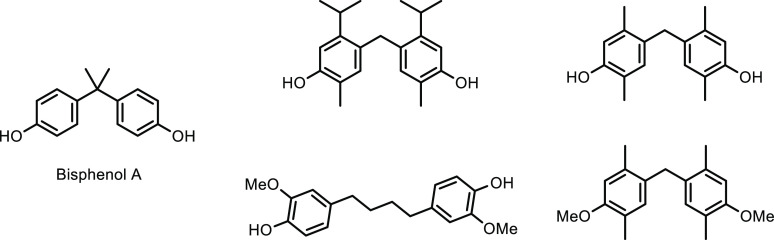
Bisphenol A (BPA) and a series of bio-based bisphenol alternatives with lower toxicity.

A lot of work has been completed to replace BPA with less-toxic bio-based monomers, as recently reviewed by Liguori *et al*.^1145^ A wide range of aromatic or rigid cyclic diols have been produced from sustainable feedstocks including cellulose-derived glucose and HMF, lignin-derived phenolics, terpenes and citric acid.^1145^ However, the lack of relevant safety data is not evidence of reduced toxicity, and these bisphenols are simply potential BPA substitutes until their toxicity can be studied. Garrison *et al*. produced a series of nine bis- and tris-phenols from a range of abundant aromatic biomass sources such as eugenol and creosol and found them to have lower toxicities than BPA (Figure 90).^1146^ This area of research would benefit from similar work across the whole range of sustainably produced aromatic and rigid cyclic diols, proving their reduced toxicity, as well as work investigating the implementation of these monomers in polymers to determine if they are functional BPA replacements.

#### 5.1.3 Diamines

**Figure 91:**
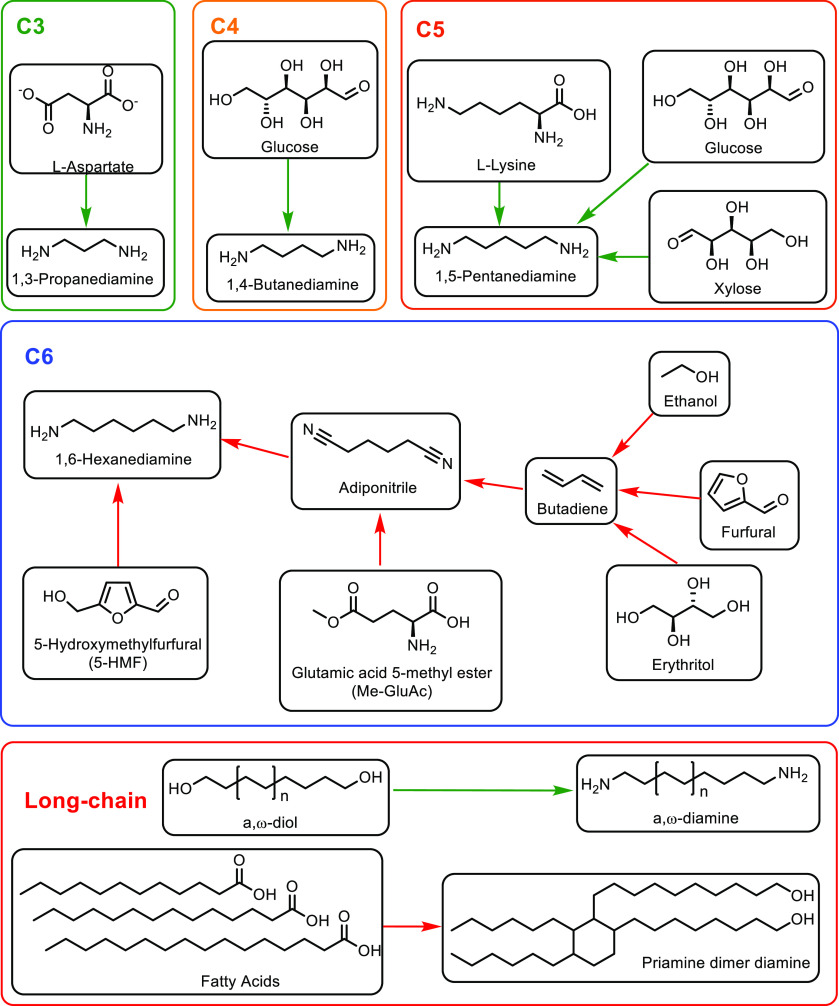
Summary of the routes to short chain aliphatic organic diamines from biomass sources, where chemical catalytic conversions are shown with red arrows and biotechnological conversion are shown with green lines.

Diamines are widely used in polymers classes such as polyamides and polyurethanes as monomers and chain extenders and have traditionally been produced largely from petrochemical sources.^1147^ Synthetic routes to biomass-derived diamines have been developed in several instances, and almost exclusively occur *via* biotechnological processes.^1147^ This is with the exception of 1,6-hexanediamine, for which extensive research has also occurred *via* chemical, catalytic routes, due to its commercial significance in the production of polyamides.^1148^ A recent review by Wang *et al*. discussed in detail the biotechnological pathways to a range of α,ω-aliphatic diamines, although importantly 1,2-ethanediamine was omitted.^1147^ While little work on the chemical production of renewable α,ω-diamines exists, the reductive amination of sustainable α,ω-diacids presents an opportunity for the sustainable preparation of a wide range of diamines. Coeck and De Vos recently published a sustainable one-pot reductive amination of carboxylic acids to primary amines using mild conditions, recyclable heterogeneous RuWO_*x*_ catalysts, and achieving yields of up to 96%.^1149^ While this has yet to be demonstrated on a significant scale with commercially relevant diamines, it has great potential to be explored as a chemical route to diamine step-growth monomers (Figure 91).

##### 5.1.3.1 C2 Diamine

**Figure 92:**
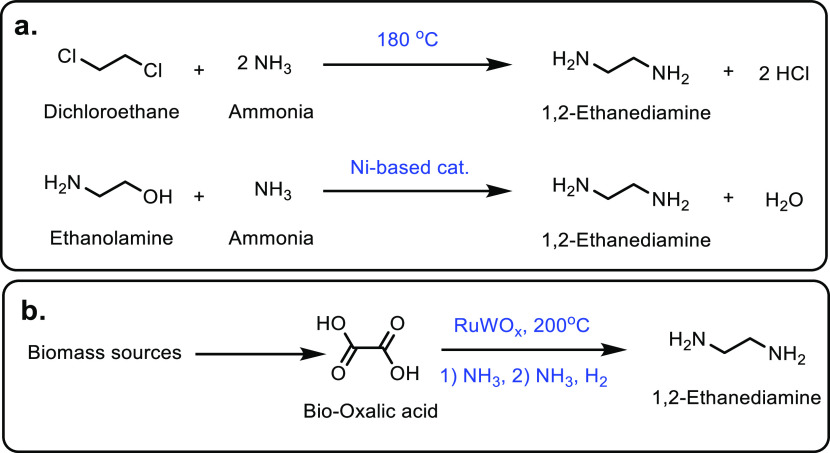
(a) Conventional methods for 1,2-ethanediamine (1,2-EDA) production from dichloroethane and ethanolamine, and (b) potential route to bio-based 1,2-EDA *via* the sustainable reductive amination of oxalic acid.

1,2-Ethanediamine (1,2-EDA, ethylene diamine) has a range of uses, including within polymer synthesis. Industrially, 1,2-EDA is made by the reaction of ammonia with either 1,2-dichloroethane or ethanolamine (Figure 92).^1150,1151^ However, in 2012 Metabolic Explorer filed for a patent on the fermentative conversion of l-serine to 1,2-EDA, suggesting commercial interest in a renewable synthesis route.^1152^ To the best of our knowledge, there are no published research articles exploring the direct synthesis of 1,2-EDA from sustainable sources, making 1,2-EDA a primary candidate for further research. Due to the numerous routes to bio-based oxalic acid (OA, section 5.1.1), the above-mentioned reductive amination of OA may present a good starting point to develop a sustainable production of 1,2-EDA (Figure).^1149^

##### 5.1.3.2 C3 Diamine

1,3-Propanediamine (1,3-PDA) has a variety of potential uses, including within step-growth polymers, as a cross-linker in epoxy resin systems, or as a pharmaceutical, agrochemical or chemical precursor. Two natural pathways to 1,3-PDA have been investigated, the C4 and C5 pathways in *Acinetobacter baumannii* and *P. aeruginosa*, respectively.^1153−1155^ Due to the cosubstrate required in the C5 pathway, the C4 pathway is considered more efficient.^859^ An engineered *E. coli* strain produced a titer of 13 g L^–1^ of 1,3-PDA from l-aspartate, highlighting the potential for efficient 1,3-PDA production *via* engineered microorganisms (Table 20, entry 14).^859,1156^ However, the l-aspartate substrate limits scalability and future routes from more readily available bioresources such as xylose are necessary.

##### 5.1.3.3 C4 Diamine

With potential uses within bio-plastics, surfactants and the agricultural and pharmaceutical industry, demand for 1,4-BDA could rise far past the current demand of approximately 10 kT annum^–1^.^1156,1157^ Two routes to 1,4-BDA are found in a wide range of organisms from l-arginine or l-ornithine.^1156^ A single-step reaction catalyzed by l-ornithine decarboxylase ODC converts l-ornithine to 1,4-BDA, while l-arginine decarboxylase ODC catalyzes the conversion of l-arginine to agmatine, which is then converted to 1,4-BDA by agmatinase.^1158^ Both *E. coli* and *C. glutamicum* have been engineered and optimized to produce 1,4-BDA, with highest reported titers of 42.3 and 19 g L^–1^ of 1,4-BDA from glucose, respectively (Table 20, entry 15).^1156,1158−1162^

##### 5.1.3.4 C5 Diamine

**Figure 93:**
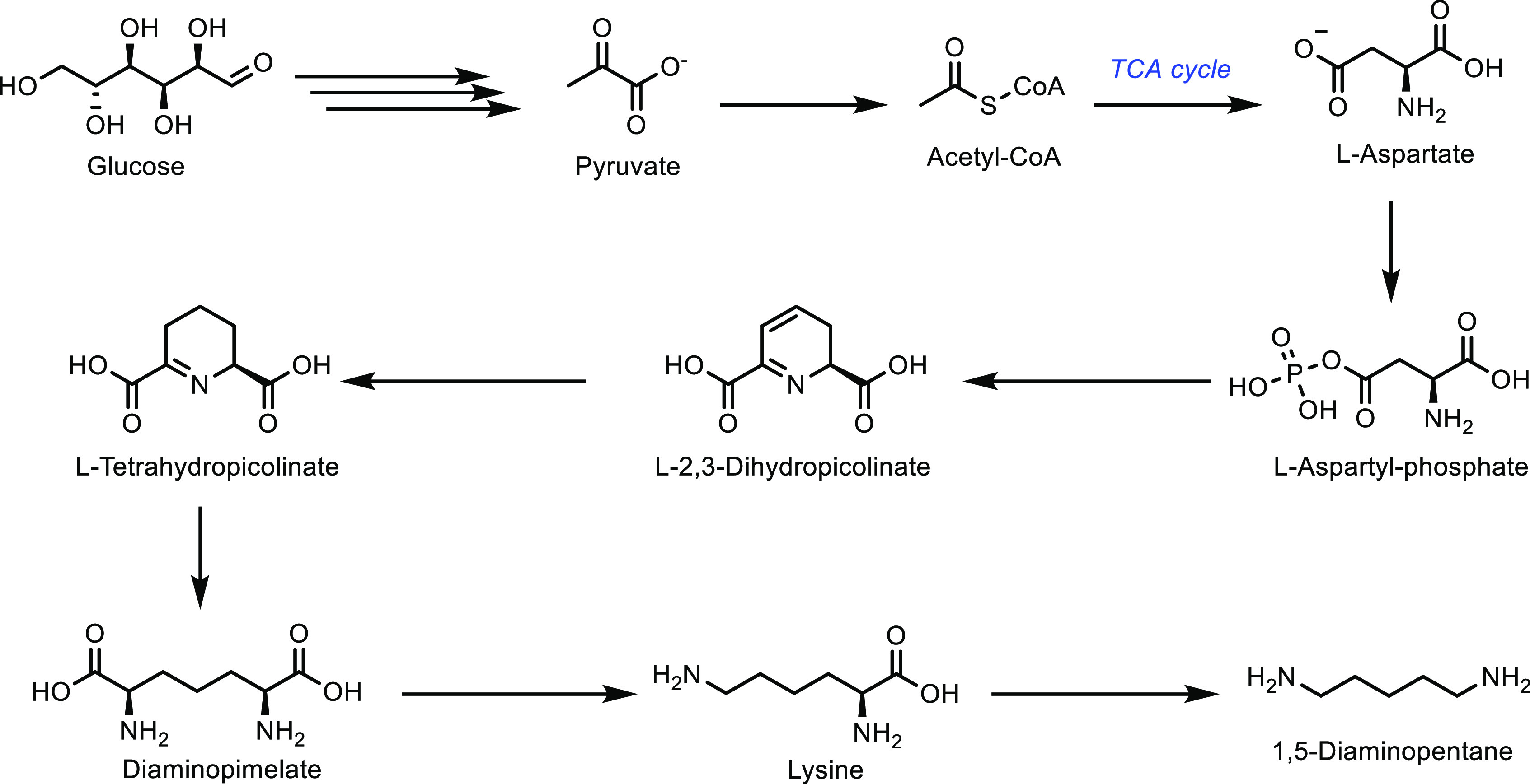
Biotechnological production of 1,5-pentanediamine from glucose, *via*l-lysine, where triple arrows indicate multiple steps.

1,5-PeDA, a C5 platform chemical, is currently biosynthesized *via* the decarboxylation of l-lysine, but has also been obtained *via* both whole-cell bio-conversion and direct fermentation processes (Figure 93).^1147^ Engineered *E. coli* with overexpressed lysine decarboxylases from various organisms have been developed for the whole cell bioconversion of l-lysine to 1,5-PeDA.^1163−1165^ The cell membranes of the impose a mass transfer bottleneck, limiting 1,5-PDA production in whole-cell bioconversion.^1147^ Overexpression of the genes responsible for l-lysine intake and 1,5-PeDA secretion resulted in the highest reported 1,5-PeDA titer and yield, 221 g L^–1^ and 92%, from l-lysine (Table 19, entry 16).^861,1166^ Whole-cell bioconversion to 1,5-PeDA has been commercialized on an industrial scale by two companies in China, Ningxia EPPEN Biotech (Ningxia) and Cathay Industrial Biotech (Shanghai).^1167^

However, the l-lysine feedstock increases the cost of whole-cell bioconversion, relative to the cost of cheaper, more abundant, sugar feedstocks. Direct fermentation of sugar feedstocks to 1,5-PeDA is highly desirable for the future of sustainable 1,5-PeDA production and has been investigated with *E. coli* and *C. glutamicum* as engineered host microbes. Despite not naturally producing detectable amounts of 1,5-PeDA, *E. coli* was engineered with suppressed 1,5-PeDA degradation and overexpressed 1,5-PeDA production.^1168,1169^ An additional iteration of relevant gene suppression gave a titer of 12.6 g L^–1^ of 1,5-PeDA from a glucose-containing medium.^1170^*C. glutamicum* has naturally high l-lysine production and has potential for the direct fermentation of glucose to 1,5-PeDA if l-lysine can be produced as an *in situ* feedstock for 1,5-PeDA production (Figure 93).^1147^ Engineered *C. glutamicum* strains have reached 1,5-PeDA titers of 103.8 g L^–1^ (Table 19, entry 17).^862,1171−1173^ To decrease production costs and avoid conflicting with food supplies, a different engineered *C. glutamicum* strain reached a 1,5-PeDA titer of 103 g L^–1^ from xylose, almost as high as the titer from glucose (Table 19, entry 18).^863,1174^ Methanol, produced on a 60 MT annum^–1^ scale and available from biomass, has been used as a cosubstrate for 1,5-PeDA by an engineered *C. glutamicum* strain, presenting an additional potential secondary feedstock for 1,5-PeDA production.^1175−1179^

##### 5.1.3.5 C6 Diamine

1,6-Hexanediamine (1,6-HDA), commonly known as hexamethylenediamine, is in huge demand as a monomer for the synthesis of polyamides 66 and 610, with the former responsible for ∼50% of all polyamides currently produced.^1148^ Almost all routes to 1,6-HDA are chemo-catalytic in nature, although a multi-step bioconversion of 2-amino-6-oxopimelate has been developed, and several non-natural metabolic pathways to 1,6-HDA have been proposed^1004,1147,1180^ 1,6-HDA is synthesized by hydrogenation of adiponitrile, *via* 6-aminocapronitrile, at high temperatures by a range of metal catalysts.^1181−1189^ 1,6-HDA is not yet produced from biomass on an industrial scale due to economic and technical limitations when compared to traditional petrochemical adiponitrile sources.^1148^ Due to the high purity requirements for use in nylon-66 synthesis and the difficulties removal of homogenous catalysts, particular focus has been given to the development of heterogeneous catalysts.^1148,1190,1191^

**Figure 94:**
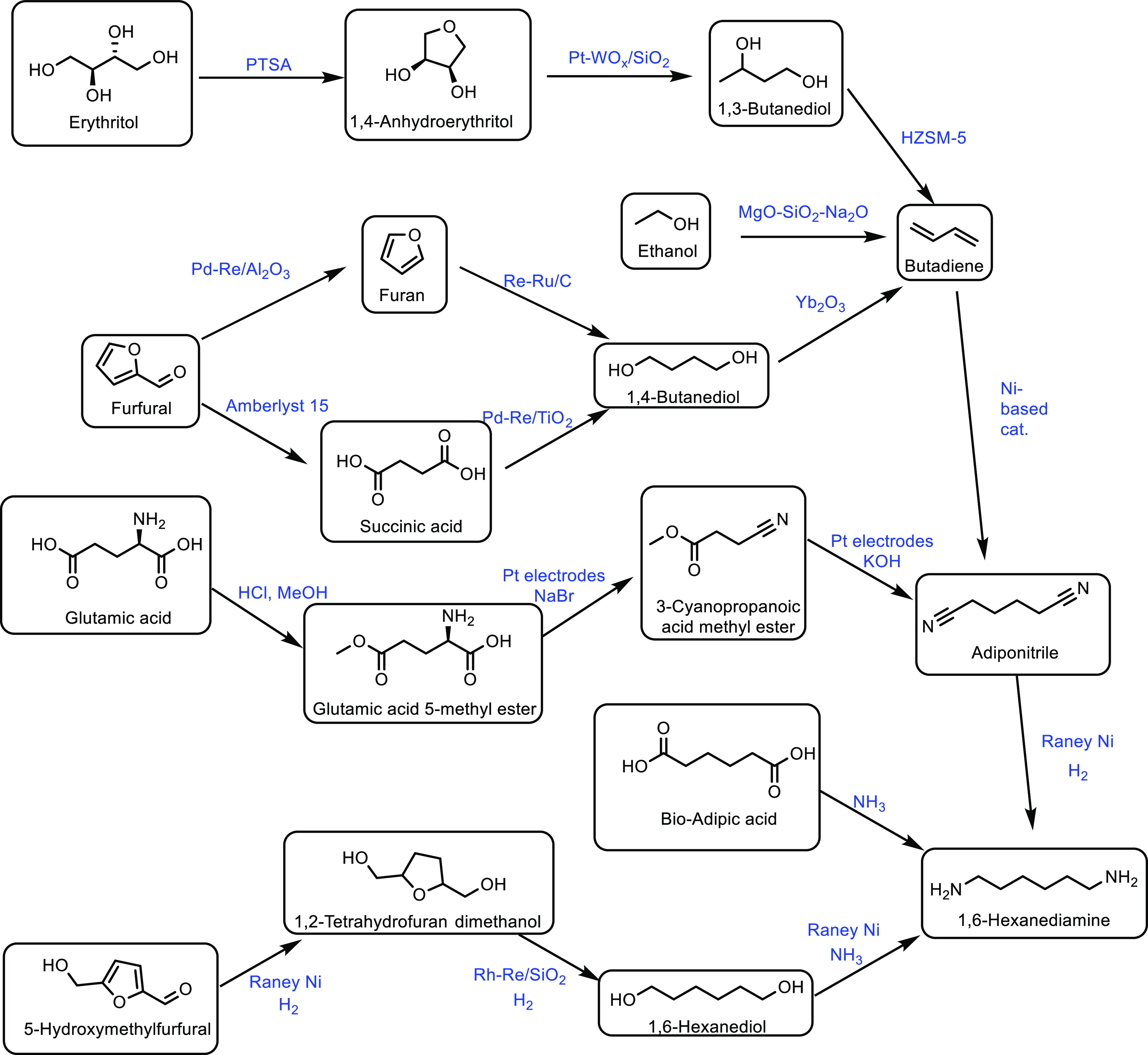
Range of routes to 1,6-hexanediamine (1,6-HDA) production from biomass sources.

The simplest route to bio-based 1,6-HDA is to produce renewable adiponitrile for use as a drop-in substitute in 1,6-HDA synthesis. Adiponitrile is most often made *via* the hydrocyanation of butadiene, which can be generated from a number of biomass derivatives such as ethanol, furfural, and 1,4-anhydroerythritol (Figure 94).^1148^ Butadiene hydrocyanation is conducted by Du Pont with a Ni-based catalyst and HCN in two steps, but a more sustainable system utilizing formamide to provide a cyano group without the need for HCN has been developed by Shu *et al.*, albeit with only 28% yield.^1192,1193^ Bio-ethanol can be converted to butadiene in a single step *via* the Lebedev process (Figure 94, Table 21, entry 1).^1194−1200^ Furfural, produced on a 0.5 MT scale yearly from hemicellulose, provides two distinct routes to 1,6-HDA *via* 1,4-butanediol (1,4-BDO) (Figure 94, Table 21, entries 2 and 3).^1201−1203^

**Table 21:** Catalysts, Conditions and Yields for the Chemical Production of 1,6-Hexanediamine and Its Intermediates from Biomass Sources *via* Chemical Conversion

Entry	Target	Feedstock	Catalyst	Temperature (°C)	Pressure (bar)	Conversion (%)	Selectivity (%)	Yield (%)	Ref.
1	Butadiene	Ethanol	MgO-SiO_2_-Na_2_O	350	1 (N_2_)	100	87	87	(1196)
2	Butadiene	Furfural	1) Pd-K/Al_2_O_3_	1) 260	1) nr	nr	nr	66	1) (1206)
			2) Re-Ru/C	2) 160	2) 30 (H_2_)				2) (1210)
			3) Yb_2_O_3_	3) 360	3) 1 (N_2_)				3) (1211)
3	Butadiene	Furfural	1) Amberlyst-15	1) 80	1) nr	nr	nr	59	1) (590)
			2) Pd-Re/TiO_2_	2) 160	2) 150 (H_2_)				2) (572)
			3) Yb_2_O_3_	3) 360	3) 1 (N_2_)				3) (1211)
4	Butadiene	Erythritol	1) Ion-exchange resin	1) nr	1) nr	nr	nr	29	1) (1212)
			2) Pt-WO_*x*_/SiO_2_	2) 140	2) 80 (H_2_)				2) (1214)
			3) SiO_2_/Al_2_O_3_	3) 300	3) 1 (nr)				3) (1215)
5	Adiponitrile	Butadiene	Ni(acac)_2_/Xantphos	150	1 (Ar)	nr	nr	28	(1193)
6	Adiponitrile	Me-GluAc	Pt electrodes	1) 0	nr	nr	nr	61	(1216)
				2) 60					
7	1,6-HDA	Adiponitrile	Raney Ni 2400	100	30 (H_2_)	100	100	100	(1187)
8	1,6-HDA	Adiponitrile	PdAg	50	nr	99	99	98	(1217)
9	1,6-HDA	5-HMF	1) Raney Ni	1) 100	1) 90 (H_2_)	nr	nr	34	1) (549)
			2) Rh-Re/SiO_2_ + Nafion SAC-13	2) 120	2) 10 (H_2_)				2) (549)
			3) Raney Ni	3) 200	3) 138 (nr)				3) (1218)

Furfural can be oxidized to SA and then hydrogenated to 1,4-BDO, or decarbonylated to furan and then converted to 1,4-BDO using a bimetallic catalyst.^571,573,590,820,1204−1210^ The best reported overall 1,4-BDO yields of these two routes are 61% and 68%, respectively, although both utilize catalysts containing palladium, an expensive rare earth metal.^573,843,1148,1206,1210^ 1,4-BDO is then dehydrated to butadiene, which has been reported with a yield of 97% using additional rare earth oxide catalysts.^1211^

Erythritol, produced from biomass on a 60 kT scale annually, can also be converted into butadiene (Figure 94, Table 21, entry 4). It is first dehydrated to 1,4-anhydroerythritol by simple Brønsted acid catalysts, with yields that can exceed 90% if reactive distillation systems are utilized.^1212,1213^ Then, hydrogenolysis of 1,4-anhydroerythritol with a Pt- and W-containing catalyst produces 1,3-BDO with a yield of 54%.^1214^ Finally, the 1,3-BDO is dehydrated to butadiene with a 60% yield using a zeolite catalyst, although catalyst deactivation occurs shortly after 100 h of use.^1215^ Both 1,3-BDO and 1,4-BDO can be produced *via* biotechnological routes (section 5.1.2), and these could be integrated with the mentioned butadiene syntheses. The butadiene obtained *via* all the routes can then be converted into adiponitrile though multi-stage hydrocyanation (Table 21, entry 5).

Adipic acid (AA) was formerly the dominant route to adiponitrile *via* high-temperature ammonization, although it is no longer used on a large scale due to economic reasons.^1219,1220^ If AA can be obtained from biomass (section 5.1.1.4), this would provide an additional route to bio-based 1,6-HDA. An electrochemical synthesis of adiponitrile from glutamic acid 5-methyl ester (Me-GluAc) *via* 3-cyanopropanoic acid methyl ester has been reported with Pt-containing electrodes.^1216^ Me-GluAc is easily accessible at high yields from glutamic acid (GluAc), and the combined steps from GluAc to adiponitrile have a yield of 58% (Table 21, entry 6).^1148,1221^ Electrochemical processes are considered more environmentally friendly than traditional chemical synthesis due to the potential use of renewable energy sources instead of harsh chemicals.^1222^ A series of hydrogenation steps converts adiponitrile to 1,6-HDA, although there are several possible side-products that can reduce yields and introduce impurities, and these must be minimized.^1223,1224^ Several techniques to impede side-product formation and increase 1,6-HDA yields have been reported, including the addition of ammonia, water, alkali metal hydroxides and ionic liquids, and the highest yields have been obtained with Raney catalysts.^1187,1225−1228^ For instance, Raney Ni has been used to achieve a quantitative yield of 1,6-HDA from adiponitrile with H_2_ at 100 °C (Table 21, entry 7).^1187^ However, using molecular H_2_ in the hydrogenation steps can be considered less sustainable due to its common production from petrochemicals, and the hazards associated with using H_2_ at high pressures (∼3 MPa).^1229^ Alternatively, formic acid, which can be sustainably produced, was used as a hydrogen donor for the hydrogenation of adiponitrile with Pd–Ag nanowires, with a 1,6-HDA yield of 98% achieved (Table 21, entry 8).^1217^

Bypassing adiponitrile, 1,6-HDA can also be made from 5-hydroxymethylfurfural (5-HMF), a platform chemical produced from glucose *via* isomerization to fructose and subsequent dehydration (Table 21, entry 9).^1230−1233^ 5-HMF is hydrogenated with Raney Ni to 2,5-tetrahydrofuran-dimethanol, hydrogenated again to 1,6-hexanediol (1,6-HDO), and then aminated to 1,6-HDA.^1129,1218,1234,1235^ The first two steps have a high combined yield of 85%, but the final amination step has a low yield of 40%.

Widespread adoption of non-petrochemical routes to 1,6-HDA is impeded by the prices of necessary intermediate chemicals, and the repurposing of waste materials from these routes could generate additional value, such as use as fuel for energy generation.^1236^ The 5-HMF route to bio-derived 1,6-HDA is considered the most competitive, with the prices of petrochemical and corn-syrup-derived 1,6-HDA estimated at $1.84 and $2 kg^–1^, respectively.^1237^ While the petroleum route narrowly remains the cheapest route, optimization of the final amination step, increasing butadiene prices, or decreasing corn syrup prices would shift the economic choice toward bio-based 1,6-HDA.^1237^ Life cycle assessment of the 5-HMF bio-based route to 1,6-HDA established that the process acts as a carbon sink, presenting an advantage when considering the climate, but can also have a higher impact on the environment, terrestrial and marine ecosystems and freshwater eutrophication.^1237^

##### 5.1.3.6 Long-Chain Diamines

Engineered *E. coli* have been designed to express an alanine dehydrogenase, which can produce non-natural diamines from their corresponding diols, l-alanine and ammonium chloride.^1238^ 1,8-Diaminoactane, 1,10-diaminodecane and 1,12-diaminododecane were produced with yields of 87%, 100% and 60%, respectively. A similar system with *E. coli* expressing an aldehyde reductase and a transaminase yielded the same three diamines with yield of 96%, 57% and 39%, respectively.^1239^ No direct metabolic pathway from biomass sources to long-chain diamines has been reported, but chemical conversion of biomass could be used to produce long-chain diamines, such as *via* the amination of castor oil-derived sebacic acid.^1240^ Croda produce a diacid from dimerizing unsaturated fatty acids, which has been converted to diamine and commercialized under the brand name Priamine (Figure 78c).^1241^ The nature of the dimers is the same as those previously mentioned (Section 5.1.1.5), only with the diacid functionality replaced with diamine functionality.

##### 5.1.3.7 Aromatic Diamines

**Figure 95:**
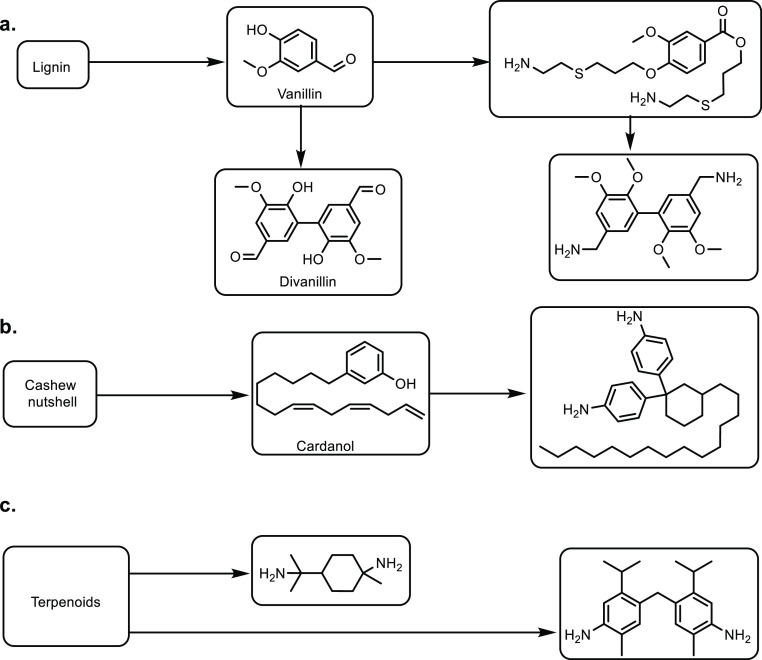
Examples of aromatic/cyclic aliphatic diamines produced from three key bio-based sources, i.e., (a) lignin, (b) cashew nutshell and (c) terpenoids.

Aromatic diamines are key building blocks in the chemical industry, and the development of aromatic diamines from renewable carbons opens the possibility for a range of novel bio-based polymers with useful properties such as high strength and high melting and glass transition temperatures.^1147^ In recent years, bio-based diamines have been derived from three key biomass feedstocks: lignin, cashew nutshell and terpenoids, with some examples shown in Figure 95.

Lignin, as the most abundant source of aromatics from biomass, is the ideal feedstock for aromatic bio-based diamines. Vanillin is produced commercially from lignin and can be converted to pure divanillin *via* enzymatic catalysis (Figure 95a).^1242−1244^ Three diamines have been derived directly from vanillin, while two more have been derived from divanillin, with the resultant diamines containing multiple pendant methoxy groups and one or two aromatic rings, respectively.^1244,1245^ The latter have been used to cross-link bio-based epoxy thermosets, exhibiting utility within thermoset materials.^1246^

Cashew nutshell liquid is an attractive biomass feedstock due to its low cost, high abundance, and easy separation into various valuable chemicals exhibiting unique structural features.^1247,1248^ An excellent review article on the synthesis of bio-based amines had been reported by Caillol et al.^1248^ One such chemical, isolated *via* distillation, is cardanol, which contains an unsaturated alkenyl side chain, an aromatic ring and a phenolic hydroxyl group, and is considered desirable for the synthesis of difunctional monomers (Figure 95b).^1247,1248^ Multiple diamines have been derived from cardanol, with diverse structural features such as long aliphatic spacers or the removal of the phenyl hydroxyl functionality.^1249−1251^

Terpenoids are abundant biomass hydrocarbon compounds have also been shown as a potential source of a range of diverse bio-based aromatic compounds. A primary non-aromatic aliphatic cyclic diamine, menthane diamine, was synthesized at low temperature in the presence of water, sulfuric acid and hydrogen cyanide, while *p*-cymene was converted to a diamine containing two aromatic rings (Figure 95c).^1239,1252^

#### 5.1.4 Diisocyanates

**Figure 96:**
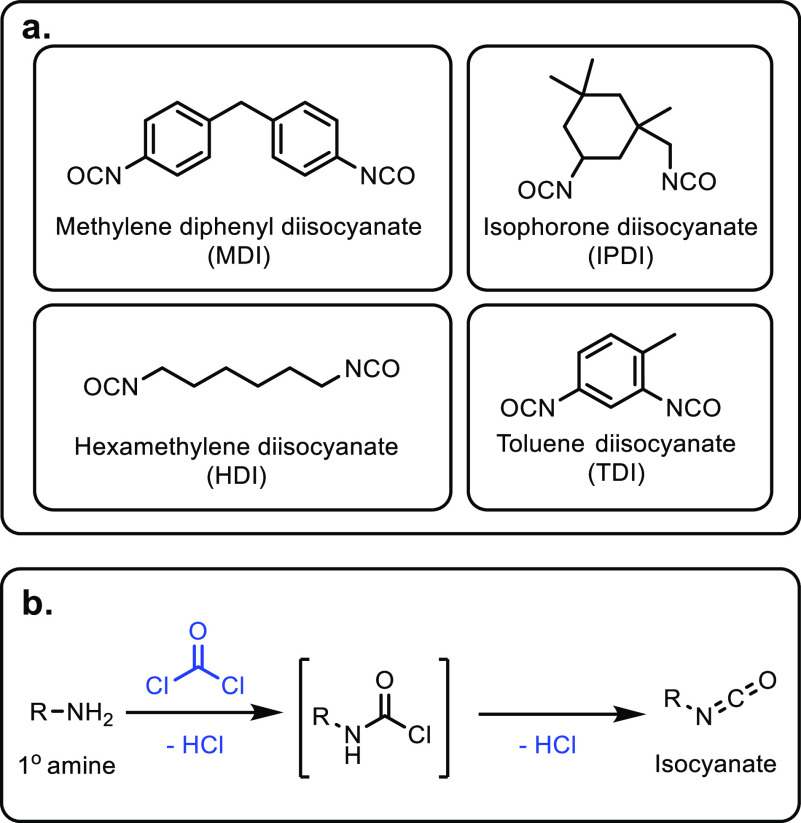
(a) Four most common diisocyanates, and (b) conventional synthetic route to isocyanates.

Diisocyanates are most commonly combined with polyols in the synthesis of polyurethanes, a prominent class of polymers discovered 70 years ago.^1253^ The most commonly used diisocyanates are the aromatic methylene diphenyl diisocyanate (MDI) and toluene diisocyanate (TDI), as well as hexamethylene diisocyanate (HDI) and isophorone diisocyanate (IPDI), constituting 61%, 34%, 3.4% and 1.2% of global demand, respectively (Figure 96a).^1254^ There are several serious concerns with conventional isocyanate synthesis, including non-renewable petrochemical feedstocks, the use of toxic reactants and the toxic nature of the isocyanates themselves.^1253^ The replacement of conventional diisocyanates with bio-based drop-in analogues presents the most likely shift toward more sustainable polyurethanes and polyureas, particularly in the short term. Non-isocyanate routes to polyurethanes and polyureas offer more sustainable long-term routes and are discussed later (sections 5.2.3 and 5.2.4, respectively).

Conventional diisocyanates are synthesized through a number of different routes, the most commonly being the reaction of primary amines with phosgene (Figure 96b).^1253^ Although efficient, avoiding the direct use of extremely toxic phosgene gas is highly desirable, and several phosgene derivatives such as trichloromethyl chloroformate and bis(trichloromethyl) carbonate are often used at a lab scale.^1253^ Other, safer, methods include the application of stoichiometric amounts of di-*tert*-butyl dicarbonate (Boc_2_O), the nucleophilic substitution of alkyl halogenides, triflates, tosylates or mesylates with metal cyanates, the thermal decomposition of carbamates, and the use of triphenylphospine, 2,3-dichloro-5,6-cyanate and tetrabutylammonium cyanate to trimethylsilyl ethers, alcohols and thiols.^1255−1258^ On a lab scale, additional frequently used methods utilize hazardous chemicals such as azides, Br_2_, acetyl chloride and ammonium persulfate.^1253,1258^

There are several routes to diisocyanates that are considered more sustainable than conventional isocyanate synthesis methods. For example, aryl isocyanates such as TDI and MDI can be obtained *via* the reductive carbonylation of nitroarenes with carbon monoxide.^1259,1260^ A range of catalysts based on metals, including Te, Se, S and a range of group 8–10 metals have been successfully used in these reactions, but catalyst residues in products commercialization *via* this method.^1253,1260^ More commonly used is the carbonylation to *N*-phenyl carbamates, followed by a thermal decomposition to isocyanates, for which many catalysts have been described, including cheap iron-based catalysts.^1261−1264^

**Figure 97:**
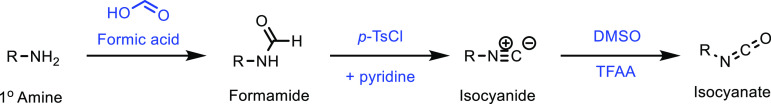
Procedure for the conversion of a primary amine to an isocyanate, *via* an isocyanide intermediate.

The conversion of isocyanides also presents a possible route to isocyanates, with a sustainable oxidation procedure applying DMSO and catalytic amounts of trifluoroacetic anhydride described by Le and Ganam, where earlier methods often utilized hazardous oxidation agents containing mercury or lead.^1265^ Isocyanides are commonly made by the dehydration of *N*-formamides, which can be readily prepared in a sustainable manner from primary amines *via* heating in recyclable formic acid or formic acid esters (Figure 97).^1266^ The dehydration step presents a barrier to sustainability, but a recent paper by Meier and colleagues developed a sustainable dehydration process using *p*-toluenesulfonyl chloride (*p*-TsCl) and pyridine.^1267^ Notably, *p*-TsCl can be obtained as an industrial waste product and is readily available, while pyridine reportedly presents the least hazardous option when compared to many other common amines.^1267^ Hence, the combination of bio-based diamines with sustainable conversion processes to *N*-formamide, isocyanide, and finally isocyanate, presents one attractive route to sustainable, bio-based diisocyanates. However, while more sustainable than the preceding routes, these processes still require solvents such as DMSO and stoichiometric amounts of base.

Carbamates, also known as urethanes, are key intermediates in isocyanate synthesis, and can also be directly converted to polyurethanes *via* transesterification with a diol, bypassing the need for diisocyanates.^1253^ Previously, silanes and boron halogenides were used as catalysts, limiting the reaction scale due to toxicity and high prices, and aliphatic carbamates were avoided due to side reactions in the thermal fragmentation.^1258,1268^ Recently, the conversion of carbamates to isocyanates *via* thermal decomposition was modelled, although this focused on the preparation of mono-isocyanates, and the authors note that many industrial patents exist concerning this process, suggesting companies have calculated the process to be commercially viable.^1269^ Carbamates have traditionally been produced from alcohols and amines with phosgene derivatives, such as chloroformates, which is suitable for lab-scale peptide chemistry but not appropriate for large-scale synthesis.^1259^ Three well-known rearrangements enable carbamate production from carboxylic acids; the Curtius rearrangement of acyl azides, the Lossen rearrangement of hydroxamic acids and the Hoffman rearrangement of carboxamides.^1253,1259^ However, these pathways suffer from toxic and often explosive reagents, which could limit the processes’ scalability and sustainability.^1270,1271^ Modern Hoffman procedures have enabled the conversion of amides to methyl carbamates with hypervalent iodine under mild conditions.^1272^ As well as from amines, alcohols, and acid derivatives, routes from nitriles, aldehydes and ketones to carbamates exist, but these also suffer from sustainability issues.^1259^

**Figure 98:**
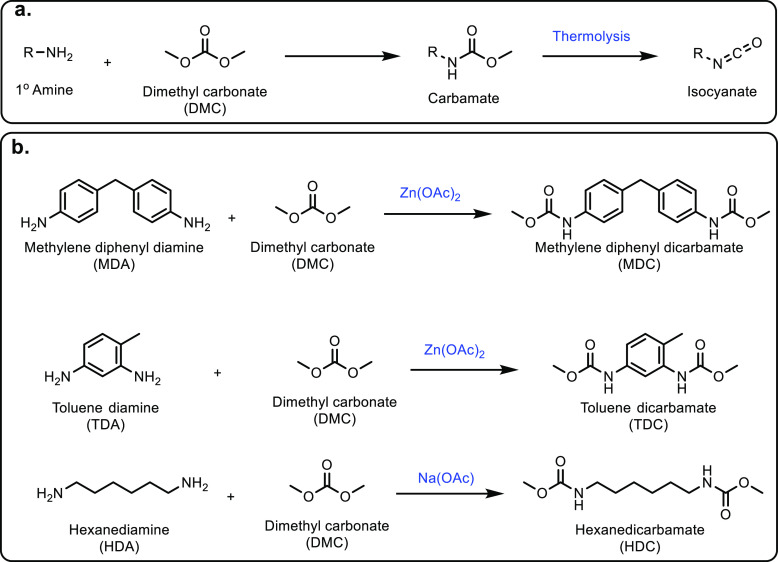
(a) General scheme for the synthesis of an isocyanate from a primary amine and dimethyl carbonate, *via* a methyl carbamate intermediate, and (b) established syntheses of the dimethyl carbamate precursors to three commonly used diisocyanates.

In recent years, more sustainable routes to carbamates have emerged, with the conversion of organic carbonates appearing the most promising.^1253^ The catalytic conversion of primary and secondary amines with dialkyl/diaryl carbonates, first reported in 1983 with *N*,*N*′-disuccinimido carbonate, has since been reported with a number of activated carbonates.^1253,1273,1274^ More recently, catalysts systems such as Zr(O*t*-Bu)_4_/1-hydroxypyridine have been reported for the conversion of amines using non-activated dialkyl carbonates.^1275^ The dimethyl carbamate precursors of TDI and MDI, the most industrially relevant diisocyanates, were prepared with almost-quantitative yields from their corresponding aniline and dimethyl carbonate (DMC) with 1.0 mol % zinc acetate as a catalyst (Figure 98b).^1276^ Similarly, sodium acetate has been shown as an effective catalyst for the conversion of 1,6-hexanediamine (HDA) and DMC *via* methoxycarbonylation.^1277^ A strong guanidine base, 1,5,7-triazobicyclo [4.4.0]dec-5-ene (TBD), catalyzes carbamate formation from DMC with primary, renewable diamines, as well as from DMC with primary, secondary and tertiary diols, although a number of other catalysts have been reported.^1278−1280^

Enzymatic alkoxycarbonylation has been used to achieve chiral carbamates, mono-substitution of a diaminonucleoside and other interesting carbamates.^1253,1281,1282^ Catalyst-free synthesis of methyl carbamates from aliphatic amines and dimethyl carbonate was achieved in 2005 in supercritical CO_2_ at 130 °C, with yields reaching 90% within 4 h.^1283^ Meier and co-workers introduced an eco-friendly synthesis of carbamates from hydroxamic acids *via* the aforementioned Lossen rearrangement with a range of dialkyl and diaryl carbonates.^1284^ Using a recyclable solvent mixture with catalytic amounts of tertiary amine bases, anilines were produced with yields of up to 83%, presenting a promising process for the synthesis of carbamates from carboxylic acids. Other routes to carbamates with potentially sustainable credentials include a three-component reaction of a primary or secondary amine with CO_2_ and an electrophile, sustainable oxidation of aromatic aldehydes with urea hydrogen peroxide, and Cu-catalyzed oxidative C–O coupling of fully substituted formamides.^1259,1285−1287^ The latter is particularly promising due to the simple access to formamides from the reaction of the corresponding amine with sustainable formic acid.^1266^

To use organic carbonates for the synthesis of sustainable carbamates, the carbonates themselves must be sustainably produced. There are well-known procedures for the synthesis of DMC that do not require phosgene or derivatives thereof.^1288^ These are the catalytic conversion of CO_2_ and MeOH, the catalytic oxidative carbonylation of MeOH, the catalytic conversion of urea, and the two step conversion of ethylene/propylene oxide with CO_2_. The first route produces water that must be removed to drive the reaction equilibrium, which has been circumvented in several ways.^1289−1291^ Catalysts such as CeO_2_ and KCl-doped-ZrO_2_ have been used, but an ionic-liquid-supported Ce_*x*_Zr_1–*x*_O_2_ catalyst proved the most promising catalyst system, although others have been described.^1292,1293^ A number of methods have been developed for the oxidative carbonylation of methanol to DMC, with an efficient and recyclable Schiff base/zeolite catalyst developed by Li and co-workers.^1294−1296^ A series of zinc based catalysts have been applied for the conversion of urea to DMC with an alcohol, and a selective batch-fed urea methanolysis system using an Fe_2_O_3_-based catalyst has also been reported.^1297−1300^ The most common method for the preparation of carbonates is a conversion of ethylene/propylene to ethylene/propylene oxide and its subsequent transesterification with MeOH to give DMC.^1253^ A range of catalysts including Mg-, Au- and phosphonium halide-based systems, a KI/K_2_CO_3_ mixed system, and an electrochemical ionic liquid system have been explored for this route.^1253,1301−1307^ As evidenced by the above examples, and the additional unmentioned routes to DMC, DMC appears to be a readily available nontoxic phosgene substitute for the synthesis of carbamates, and hence diisocyanates and their polyurethane and polyurea derivatives.

### 5.2 Step-Growth Polymers

Unlike the other sections of this review, the difunctional monomers mentioned within this section must be combined with a second difunctional monomer to give an AA+BB step growth polymer. Hence, having discussed the most important monomers within each class, it is important to frame the significance of the monomers by briefly discussing the 5 relevant polymer classes. While the overall focus of this review is on producing the monomers already commonly used, this section will also highlight areas where common polymers may be displaced by alternative polymers in the future, as well as polymers that may have great potential but are currently underutilized.

#### 5.2.1 Polyesters

Polyester materials are incredibly important polymer materials that contain an ester bond within their repeat unit and find wide-ranging applications in plastic bottles, films and textiles. Although technically comprising of a whole class of polymers, the term polyester is used ubiquitously to refer to polyethylene terephthalate (PET), which currently dominates the polyester market. Indeed, in 2020, the global PET annual production was over 57 million tons with a market size of approximately $110 billion.^1308^

**Figure 99:**
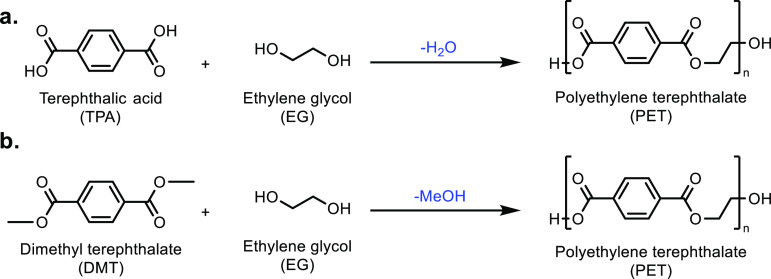
Production of polyethylene terephthalate *via* (a) polyesterification of TPA and EG, and (b) transesterification of DMT and EG.

The largest applications of PET are in fibers for textile production (60%) and bottles for water and carbonated drinks (30%). PET possesses several attractive properties for these applications, such as high hardness, stiffness and strength, a wide service range, controllable transparency and high hydrolytic resistance at room temperature. PET is produced from ethylene glycol (EG) and either terephthalic acid (TPA) or its dimethyl terephthalate (DMT) derivative in a (trans)esterification polymerization reaction (Figure 99). Conventionally, all these monomers were obtained from petrochemical sources, but in recent decades routes have emerged to produce EG, TPA and DMT from biomass sources. The bio-based production of EG is far more developed than that of TPA, due to the aromatic structure of TPA being more cumbersome to derive from readily available bio-based feedstocks. Moreover, bio-ethanol production is well-established as a main EG feedstock. As such, the earliest instances of incorporating biomass into PET production combined bio-derived EG with petrochemically sourced TPA or DMT, as demonstrated in 2009 when Coca Cola introduced a bottle containing 30% biomass.^1309^

Since then, fully bio-based PET has been demonstrated in fiber applications and commercialized in soft-drinks bottle applications *via* the inclusion of TPA derived from bio-based *p*-xylene.^986,987^ However, the scale of these operations are tiny relative to overall PET production, and is far from presenting a solution to the needs for PET supply. While mainly outside the scope of this review, it is worth mentioning that large effort in recent decades has been invested in developing the technology to recycle PET, either mechanically or chemically, with companies aiming to include 50% recycled PET in their products by 2030.^1310,1311^ Hence, any sustainable future for the use of PET would rely on a combination of continuing to move away from petrochemical feedstocks and further developing PET recycling technology, hence reducing the demand for virgin petrochemically sourced PET.

Aside from PET, a range of other polyesters have been shown to have great commercial potential, even outperforming PET in specific applications. These include poly(lactic acid) (PLA), poly(butylene succinate) (PBS) and poly(ethylene 2,5-furanoate) (PEF), among several others.^1312−1314^ PLA, produced *via* ring-opening polymerization of lactides (cf. section 4.3), is a widely used bio-based plastic with impressive physical properties but weaker thermomechanical properties than PET.^1312,1313^ PBS, an aliphatic polyester produced from 1,4-butanediol (1,4-BDO) and succinic acid (SA) in a polycondensation process similar to that of PET, exhibits promising sustainability due to its production from sustainable feedstocks and its biodegradability.^1315^ Both SA and 1,4-BDO have established production routes from biomass, with more continuing to be developed, as has been discussed in previous sections. Additionally, the degradable nature of ester linkages combined with the small metabolism-compatible aliphatic degradation products, give PBS biodegradable properties. In soil, PBS degrades significantly faster than conventional plastics, with numerous microorganisms documented to assist in its degradation.^1315^ PBS benefits from rigidity and transparency, and has found applications within compostable tableware, food packaging and agricultural mulch films, as well as various biomedical applications.^1316−1319^ However, PBS use is currently limited by its relatively high price at ∼$50 kg^–1^, being an order of magnitude greater than that of PET.^1315^ As PBS production grows, this price differential can be predicted to shrink, although the incredibly cheap nature of petrochemically sourced PET means that PBS will still remain substantially more expensive for the foreseeable future.

**Figure 100:**
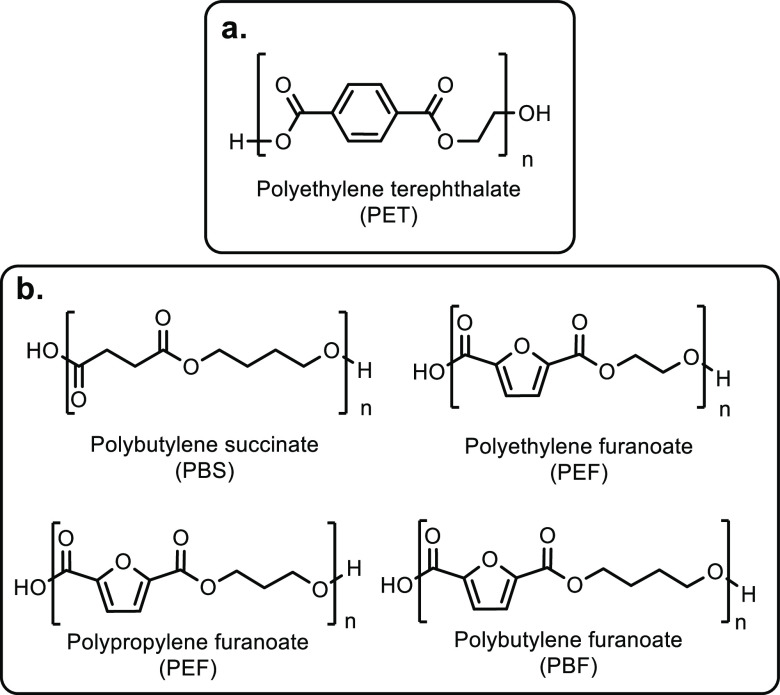
(a) Dominant polyester PET, and (b) some promising bio-based polyesters to displace PET in certain applications.

PEF is considered a top candidate for the phasing out of petrochemically sourced polyesters such as PET, in particular due to furan-2,5-dicarboxylic acid’s (FDCA) position as an aromatic bio-based platform chemical and PEF’s thermomechanical properties resembling those of PET (Figure 100).^995,1320−1322^ As with most AA+BB type polyesters, PEF synthesis follows similar routes to those described for PET production, and Fei *et al*. recently reviewed a range of polyesters derived from FDCA, including PEF.^1312^ Since the main issue with PET production is sourcing TPA, PEF provides an alternative, where the same simple diol and a similar aromatic diacid are used. Furthermore, food-contact-grade PEF has been produced with low discoloration and high transparency, and PEF has been demonstrated to possess O_2_ and CO_2_ barriers 10-20 times than those of PET, and water barriers twice as great.^1323−1325^ This has been attributed to the nonlinearity of the furan structure in FDCA inhibiting ring-flipping.^1326^ Avantium have a test plant in the Netherlands producing FDCA and then PEF using their melt-polymerization YXY® Technology, and plan to produce 5 kT annum^–1^ in a pilot plant opening in 2023.^1327,1328^ Additionally, researchers at ETH Zürich have produced bottle-grade PEF from cyclic oligomers, and are working toward adapting them for industrial scale demands.^1329,1330^ As PEF is commercialized and more thoroughly investigated, the cost of FDCA will decrease, and the range of applications in which PEF could be beneficially used will become more apparent.

Polybutylene terephthalate (PBT) and polypropylene terephthalate (PPT) are well known engineering plastics made from the reaction of terephthalic acid with 1,4-BDO or 1,3-PDO, respectively.^1312^ The FDCA analogues of these polyesters, poly(butylene 2,5-furanoate) (PBF) and poly(propylene 2,5-furanoate) (PPF), have been reported to exhibit comparable thermomechanical properties, in particular thermal stability and mechanical strength, presenting opportunities to reduce the amount of petrochemical TPA required.^1331^ Polyesters of FDCA with aliphatic diols (C3–C18) showed glass transition temperatures (*T*_g_) decreasing from 90 °C in PPF to under 0°C in poly(dodecyl 2,5-furanoate), enabling a range of control over thermal properties by diol monomer choice.^1322,1332^ Polyesters of FDCA with rigid diols have increased *T*_g_ values, as high as 180 °C in the case of poly(isosorbide 2,5-furanoate).^1320^ The inclusion of isosorbide in fully bio-based polyesters based on FDCA and short aliphatic diols for food packaging applications increased the *T*_g_ by 40 °C, and elsewhere isosorbide content has been shown to increase hydrolytic degradability.^1333,1334^

One of the great benefits of AA+BB polyester synthesis, and AA+BB polymers more generally, is the simplicity of the polymerization processes and the tuneability of the monomer composition to achieve copolymers with a range of desired properties. As the field of polyester chemistry moves away from being a monolith primarily utilizing PET for most applications and the sustainable synthesis of a range of bio-based difunctional monomers becomes more realistic, a more diverse range of polyesters, including some not described here, will be designed for usage in specific applications. An important consideration for AA+BB polymerizations, however, is the need for precise stoichiometric ratios in order to reach high molecular weights, which can pose demands on reliable bio-based monomer purity and hence could require intensive and costly purification processes.

#### 5.2.2 Polyamides

**Figure 101:**
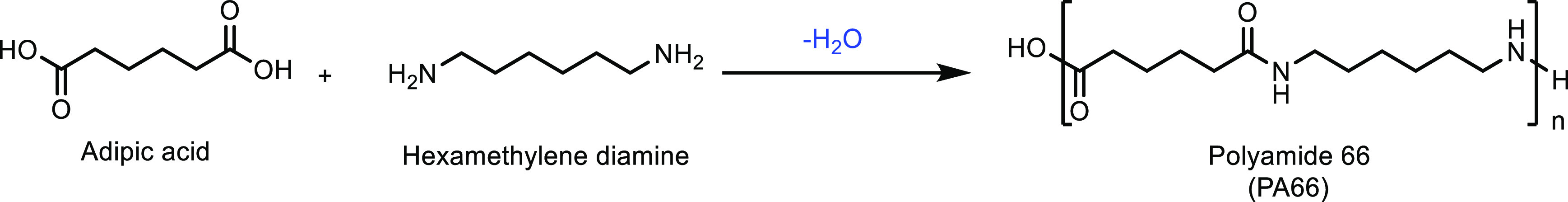
Production of polyamide 66 (PA66) from adipic acid and hexamethylene diamine.

Polyamide materials, commonly referred to as nylons, are another important class of step-growth polymers that contain an amide bond within their polymeric repeat unit. Polyamides are synthesized from a diamine and a diacid, or derivatives thereof (e.g., acid chlorides, ester). Polyamides find use in a range of applications within the automotive, food, construction, and apparel industries, as well as in many other applications.^860,1169,1335^ Nylon-66 (PA66) and nylon-6 (PA6) are the two most commonplace polyamides, with an estimated combined demand of over 1 Mt in 2019 (Figure 101).^1336^ Although PA6 is produced by the ring-opening polymerization of caprolactam (cf. section 4.2.2), PA66 can be produced *via* the polycondensation reaction of adipic acid and 1,6-hexanediamine, two monomers that have been explored in detail within this section. The impressive mechanical properties of PA66, including rigidity, high mechanical strength and robustness against heat and chemicals, have cemented its position as a high-performance commodity polymer.^1337^ In 2013, Rennovia launched a 100% bio-based PA66 product, but this was discontinued, with Rennovia ceasing operations in 2018.^1338,1339^ A recent collaboration between Genomatica and Asahi Kasei seeks to commercialize bio-based PA66.^1340^ As bio-based routes to the HDA and AA monomers continue to be developed, PA66 production will likely shift toward bio-based feedstocks. Due to their similar structures and properties, and the lower cost of PA6 over PA66, industries have somewhat prioritized employing PA6 wherever possible.^1341^ However, other polyamides with similar properties, which have previously been neglected due to more expensive petrochemical monomer syntheses, may become more commonly used if they can be produced from biomass sources more easily than the PA66 monomers.

Besides HDA, the most promising diamine for bio-based commercialization is 1,5-PeDA, which can be copolymerized with AA to give PA56. PA56 is regarded as the bio-based polyamide best-suited to displace PA66, due to their similar structures and the potential for high-volume 1,5-PeDA production.^1342^ While PA56 underperforms in some ways, such as being brittle at low-temperatures and having poor notched impact strength, blending with an elastomeric interfacial compatibilizer has had an ultra-toughening effect on PA56.^1342^ As such, PA56 may replace PA66 in some applications if and when it is commercialized. Other polyamides, such as PA610 and P612, produced from the copolymerization of HDA with sebacic acid (C10) and dodecanedioic acid (C12), respectively, can also be produced from bio-sourced monomers, and exhibit lower moisture absorption and heat resistance than PA56.^1342−1345^ However, the production of these diacid monomers from bio-sources is still under-developed.

Of the discussed aliphatic diacids, oxalic acid (C2) and succinic acid (C4) appear the most promising for large-scale bio-based production, and these can be copolymerized with 1,5-PeDa to give PA52 and PA54, respectively. PA52, first prepared by Mutua *et al.* in 2018, has the shortest possible diacid component and exhibits high heat-resistance, low water absorption, and excellent crystallizability.^1346^ With thermal stability equal to that of PA6, PA52 is very appealing as a bio-based high performance engineering plastic. PA54, first prepared by Lee *et al*. in 2020, has not been the subject of much work, although one study investigated the formation of the nylon salt of the two monomers. In another report, direct solid-state polymerization was employed in place of melt-polymerization, due to the tendency of SA to undergo intra-cyclisation at high temperatures.^1347−1349^ Although the synthesized PA54 degraded at temperatures lower than conventional polyamides (i.e., 320 °C), the melting point remains higher than that of PA66 (i.e., 275 °C).^1347,1349^ Due to its higher amide group concentration, PA54 possessed higher water absorption than PA66, and interestingly also exhibited microbial biodegradability, indicating the possible recyclability of PA54 as a bio-based nylon.^1347^

Partially or fully aromatic polyamides, termed aramids if >85% of the amide linkages are between two aromatic rings, are very high-performance polyamides, with aerospace and military applications such as Kevlar for bulletproof vests. Semi-aromatic polyamides such as PA6T and PA6I, derived from 1,6-HDA with terephthalic or isophthalic acid, respectively, are heat resistant polymers with high melting and glass-transition temperatures, high rigidity, and low water absorption.^1350^ Recent work by Shen *et al*. synthesized PA5F and PA10F, novel semi-aromatic polyamides of 2,5-FDCA with 1,5-PeDA and 1,10-decanediamine, respectively.^1351^ Both polyamides showed high thermal stability (i.e., up to 380 °C), with PA5F showing a relatively high *T*_g_ of 86 °C and PA10F exhibiting a lower *T*_g_ of 35 °C, and were identified by the authors as potentially useful materials for bio-based 3D-printing applications.

**Figure 102:**
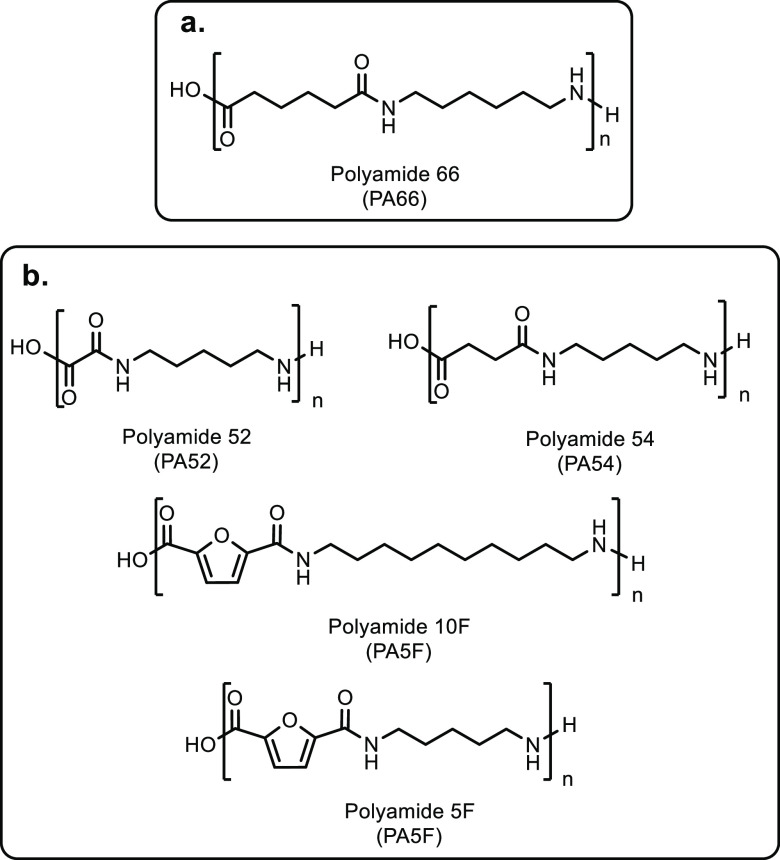
(a) One of the most common polyamides, PA66, and (b) some promising bio-based polyamide analogues for the displacement of PA66.

As the discussed polyamides only utilize the most obvious monomer choices and are fairly recent, this is an active research area, and a range of further bio-based polyamides will emerge in the coming years. Currently, the most interesting novel polyamides subject to further investigation appear to be PA56, PA52, PA54 and PA5F, although the development of additional easily accessible diamines is expected to open up a range of new polyamides to be studied (Figure 102).

#### 5.2.3 Polyurethanes

Polyurethanes (PUs) are a versatile class of polymers with excellent strength-to-weight ratios and energy-absorbing properties.^1253^ PUs find applications as elastomers, hard/flexible plastics and soft/rigid foams, within a number of fields including the automotive, industrial and medical fields, with a global market of 24 MT in 2020.^1352−1354^ The typical synthesis of PUs involves the catalyzed polyaddition reaction of a diisocyanate with a polyol. As this review mainly focusses on thermoplastic materials, the polyol discussion herein will be limited to diols. The diols chosen for PU synthesis are typically hydroxy-terminated low molecular weight polymers, such as polyethers, polyesters and polycarbonates, which can be readily produced from a range of bio-based sources.^1137^ As mentioned in section 5.1.4, conventional PU synthesis suffers from health concerns due to its reactive and toxic nature, in particular when using low molecular weight diisocyanates.^1355,1356^ The conventional diisocyanate route is currently the dominant route and would benefit from sustainably sourced diisocyanate monomers.

**Figure 103:**
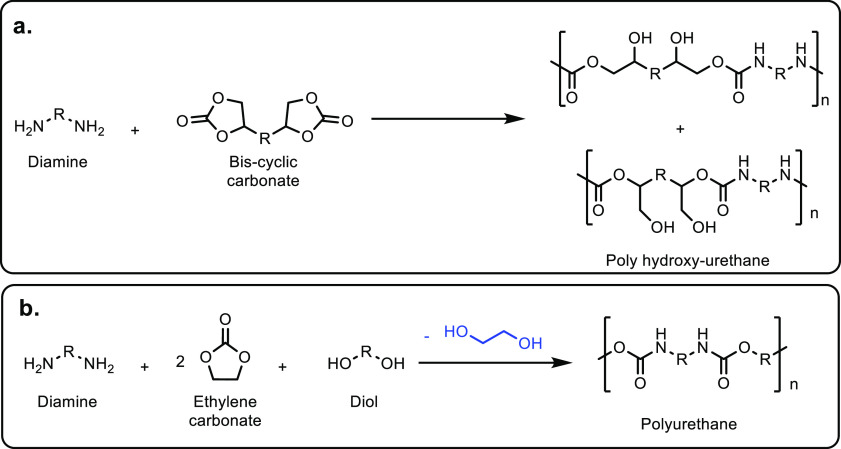
Synthesis of non-isocyanate polyurethanes (NIPUs) from (a) the polyaddition of a diamine and a bis-cyclic carbonate, and (b) the polycondensation of a diamine, a diol and ethylene carbonate.

In anticipation of bio-sourced PU production, the synthesis of so-called non-isocyanate PUs (NIPUs) has rapidly emerged and might present a worthy alternative pathway in the near-future. Indeed, NIPUs have gained tremendous attention in recent years, as was recently reviewed by Khatoon *et al*.^1357^ The preparation of cyclic carbonate precursors, discussed in section 5.1.4, and their conversion to carbamates (urethanes) in the presence of diamines can be used to give polyurethanes *via* a step-growth polyaddition reaction (Figure 103a).^1358^ The polyaddition of diamines with bifunctional 5-membered cyclic carbonates was first reported in 1957 and produces PU with pendant hydroxy groups.^1253,1359^ Notably, the asymmetric nature of the 5-membered cyclic carbonates results in a mixture of two slightly different polymer structures, with either an extra carbon in the backbone or in the alkyl chain of the pendant hydroxy groups. Cyclic carbonates have low toxicity, show biodegradability and are reactive toward amines, which make them very attractive materials to use for NIPU synthesis.^1360^ Furthermore, the absence of residual unstable chemicals commonly seen in PU enhance NIPUs with better thermal stability than conventional PU, although residuals hydroxyl groups are known to induce transcarbamoylation reactions.^1361,1362^ To avoid pendant hydroxyl groups, ethylene carbonate can be reacted with a diamine and a diol to produce linear polyurethanes and ethylene glycol (Figure 103b).^1363^

Alternatively to the above-mentioned step-growth polymerizations, the ring-opening polymerization (ROP) of aliphatic cyclic urethanes/diurethanes can occur *via* a chain-growth mechanism.^1364^ The 5- and 6-membered cyclic urethanes can be produced sustainable in a number of way, including with pressurized CO_2_ without a catalyst.^1365^ However, other cyclic urethanes are either unstable and difficult to form, or too stable for the polymerization reaction to proceed swiftly.^1253^

An additional non-isocyanate route to PU involves the replacement of phosgene with CO_2_, where aziridines (nitrogen epoxide analogues) react with supercritical CO_2_, yielding random PU copolymers with a mixture of amine and urethane units.^1253,1366,1367^ Ihata *et al*. observed changing LCST behaviour dependent on the CO_2_ pressure, which was attributed to the increased CO_2_ pressure causing an increase in urethane content.^1368^

As the most common tunable component in these systems is the diamine, the range of diamines available largely controls the range of PU structures available. A number of cross-linked NIPU systems based on terpenes and vegetable oils have been reported, while linear NIPUs are less commonly the focus of study.^1369,1370^ Cramail and co-workers conducted the polyaddition of various diamines with linear bis-carbonates derived from methyl oleate in supercritical CO_2_ to produce a range of linear NIPUs.^1371^ With a Ti-based catalyst, Deepa *et al*. conducted the melt polycondensation of sustainably prepared dicarbamates with diols to produce linear NIPUs, and Meier and co-workers synthesized terpene-based NIPUs in a similar manner.^1278,1372^ Khatoon *et al*. recently reviewed the state-of-the-art on NIPUs, albeit with a larger focus on the various applications that NIPUs find use in.^1357^

PUs from an incredibly useful and ubiquitous class of polymers due to their versatility and tunability. With advanced research, the uses of NIPUs are expected to become more widespread and perhaps capable of phasing out toxic diisocyanates and other phosgene derivatives in PU synthesis.

#### 5.2.4 Polyureas

**Figure 104:**
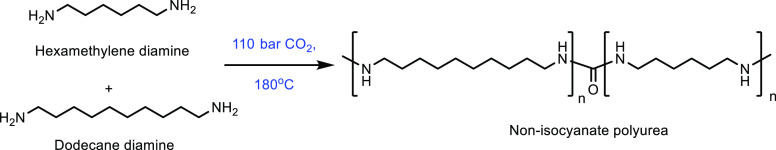
Catalyst-free synthesis of non-isocyanate polyureas (NIPUas) from the reaction of diamines in supercritical CO_2_.

Polyureas (PUas) are an important type of polymer material with structures similar to PUs. PUas are conventionally produced in a manner similar to PUs, through the polyaddition reaction of a diisocyanate with a polyamine, which is typically a diamine in the case of thermoplastic PUas. As such, conventional PUas are faced with similar concerns as conventional PUs with regard to the use of isocyanates. Additionally, there is typically a very narrow processing window between the melting and degradation of isocyanate-based PUas, such that melt processing and recycling the materials is largely ineffective.^1373,1374^ The synthesis of non-isocyanate polyureas (NIPUas) has been extensively researched, and a number of routes to thermoplastic NIPUas have been established (Figure 104). These routes typically utilize CO_2_ either as a comonomer directly or *via* some CO_2_-derived intermediate, which includes bis-carbamates and carbonates, and they often largely parallel the synthesis routes of NIPUs by using a diamine in place of a diol.^1375−1381^

The direct utilization of CO_2_ as a comonomer with a diamine has been conducted with a range of catalyst types, including ionic liquids, alkali bases, organic superbases and complex salts, as well as without a catalyst at 180 °C.^1382−1388^ However, the obtained molecular weights are typically below 4000 g mol^–1^ due to the water produced in the reaction affecting the equilibrium. Hence, underwhelming physical properties are often obtained due to the high crystallinity caused by strong regular hydrogen bonds.^1389−1391^ Recently, Shi *et al*. prepared a series of PUas formed from CO_2_, HDA and dodecanediamine (DDDA) in a two-step catalyst-free procedure (Figure 104).^1373^ While the highest molecular weight observed was still only 4500 g mol^–1^, the particular combination of diamines interrupted the crystalline packing, while maintaining less-regularly ordered hydrogen bonds. Due to this, the material’s brittleness was alleviated, and the physical properties exhibited were as good as, if not superior to, previously reported CO_2_-based PUas, isocyanate-free PUs, PA6 and conventional PUas. Additionally, the copolymers all exhibited a large processing window of ≥85 °C due to their impressive thermal stability and the depression of the two homopolymer melting points observed on the copolymer. To achieve higher molecular weight materials, indirect use of CO_2_ is required. As mentioned, one such method is the production of dicarbamates from the reaction of bifunctional cyclic carbonates, and the subsequent reaction with a diamine. Alongside making the previously mentioned linear NIPUs, Deepa *et al.* conducted the polyaddition of dicarbamates with diamines to produce linear PUas, and Koning and co-workers employed a non-metal TBD catalyst to prepare similar materials.^1372,1392^ An excellent review on the current state of polyurea research was recently published by Santana *et al*.^1393^ The work of Shi *et al*. and other similar works have highlighted the exceptional potential for the catalyst-free preparation of bio-based NIPUas directly from a diamine and CO_2_, requiring only elevated temperatures and pressurized CO_2_.^1373^ Here, the potential for bio-based NIPUas is believed to further expand as additional bio-based diamines are developed and commercialized.

#### 5.2.5 Polycarbonates

**Figure 105:**
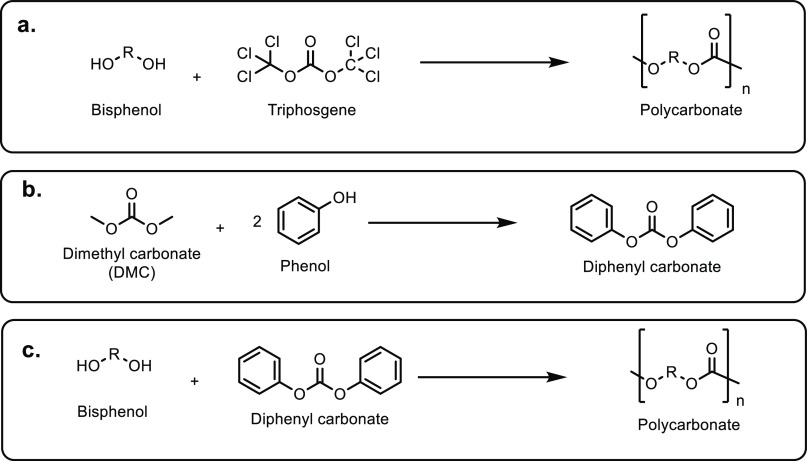
(a) Polymerization of bisphenols with toxic triphosgene to give polycarbonates, (b) production of diphenyl carbonate *via* the transesterification of DMC with phenol and (c) use of diphenyl carbonate as a comonomer with bisphenols to give more sustainable polycarbonates.

Polycarbonates (PCs) are a class of polymer with carbonate groups in their backbone. Due to their excellent properties such as ductility, creep resistance, impact resistance and optical transparency, PCs are produced in high volume as engineering plastics for use within sectors such as the construction and automotive industries.^1394,1395^ PCs are conventionally produced *via* the reaction of a diol with (tri)phosgene, with the most commonly used diol being bisphenol A (BPA, Figure 105a). Similar to PUs and PUas, the production and use of PCs has several concerns, due to the high toxicity of phosgene and the toxic and environmentally hazardous nature of BPA.^1396,1397^ Additionally, both of these components are traditionally derived from petrochemicals.

Biomass sources are seen as a potential route to both avoid the risks associated with BPA and also increase the utilization of sustainable feedstocks for chemical production. The alternative aromatic polyols produced by Garrison *et al*. (Figure 90) were then polymerized to PCs with molecular weights of up to 14 600 g mol^–1^, and *T*_g_ values in the range 51–156 °C, hence performing as good as BPA-based PCs (PCBPA) and highlighting the utility of bio-based polycarbonates as replacements for PCBPA. However, the polymerization was conducted with triphosgene, a slightly safer phosgene derivative, in order to closely follow common industrial PC synthesis procedures (Figure 105).^1146^

A number of other bio-based feedstocks have been used to synthesize PCs *via* similar routes utilizing phosgene derivatives, including sugars,^1398^ softwood lignin,^1399^ eugenol,^1400^ ferulic acid^1401^ and vanillyl alcohol.^1402^

Despite the sustainability issues of using phosgene-derivatives, bisphenols such as those mentioned present the most straightforward replacements for BPA with lower toxicity bio-based bisphenols in the short term. In the long term, a move toward more sustainable polymerization reactants will likely result in the exploitation of other methods to polycarbonate synthesis, which avoid phosgene derivatives and other petrochemically derived materials. The most common route to bio-based non-phosgene and non-BPA PCs is *via* the reaction of diols with diphenyl carbonate (DPC), which can be sustainably produced through a transesterification of bio-based dimethyl carbonate with bio-based phenol.^1140,1403−1406^ For example, novel bio-based diols with rigid cyclic ketals formed from glycerol and diketones were reacted with DPC to give PCs with glass transition and degradation temperatures above 128 and 350 °C, respectively. Similarly, a carvacrol-derived bisphenol was converted into a PC.^1404−1406^ Several other routes have been established, including the conversion of a citric acid-based diol to a bis-cyclic carbonate and subsequent step-growth ring-opening polymerization with an additional diol.^1407^ Similarly, the chain-growth ring-opening polymerization of fatty acid-based cyclic carbonates,^1408^ and the direct polymerization of a limonene oxide with CO_2_ as a green-phosgene alternative in the presence of a Zn-based catalyst have been reported.^1409^ PCs will continue to be developed as the diverse naturally occurring structures seen in bio-based feedstocks are utilized for monomer synthesis, providing great potential for phasing out the use of highly toxic phosgene-derivatives and BPA monomers in the future.

## 6 Conclusions and Outlook

Extensive research efforts in developing monomers from sustainable sources are reshaping the field of sustainable plastics. The convenience of obtaining known commodity polymer structures outweighs the additional efforts and costs related to isolation of basic chemicals from biomass and other sustainable resources. The technology for the synthesis of ethanol is already available and enables sustainable production of polyethylene and polypropylene. Similarly, various acrylics and lactones can also be obtained from carbohydrate and fatty acid derivatives. Finally, diacids and diols extracted from sustainable sources enable the synthesis of sustainable polyesters and similar step-growth polymers. The main question now is “are the consumers ready to pay the additional cost?” for the sake of obtaining the same plastics materials from sustainable sources. In 2022, following the sudden global oil and gas supply crisis, fossil fuel prices have more than doubled in less than a year, which may catalyze the methods reported in this review to become more widely available and perhaps more rewarding. Nonetheless, any extra cost associated with the shift toward (more) sustainable monomers is presumed to be compensated by introducing superior material functionality and/or properties.

While this review aims to inspire future developments toward the like-for-like substitution of petrochemicals by sustainable monomers in conventional polymerization processes, it has no intend to discourage fundamental research into creating new types of polymer materials that are derived from renewable resources. This might perhaps offer a more appropriate solution to the pressing needs for sustainable plastics in the long term. However, sustainability is a multifaceted challenge and can only truly be assessed by taking into account the entire life cycle of a plastic material, ranging from how and where monomers are sourced to its impact on the manufacturing process, material use and waste management. Hence, some renewable monomers that are generally considered to be more sustainable might still be outperformed in a life cycle analysis by their conventional petrochemical counterpart when considering one’s specific industrial process and supply chain.
